# Structural Identification of Zotarolimus (ABT-578) Metabolites Generated by Human Liver Microsomes Using Ion-Trap and High-Resolution Time-of-Flight Mass Spectrometry in Combination with the Analysis of Fragmentation Patterns

**DOI:** 10.3390/metabo13101093

**Published:** 2023-10-19

**Authors:** Touraj Shokati, Seth H. Drake, Wanzhu Zhao, Jost Klawitter, Jelena Klawitter, Uwe Christians

**Affiliations:** iC42 Clinical Research and Development, Department of Anesthesiology, School of Medicine, University of Colorado Anschutz Medical Campus, Bioscience 2, Suite 200, 12705 East Montview Boulevard, Aurora, CO 80045-7109, USA; touraj.shokati@cuanschutz.edu (T.S.); seth.drake@cuanschutz.edu (S.H.D.); wanzhu.zhao@cuanschutz.edu (W.Z.); jost.klawitter@cuanschutz.edu (J.K.); jelena.klawitter@cuanschutz.edu (J.K.)

**Keywords:** zotarolimus, human liver microsomes, human drug metabolism, metabolite structures, fragmentation patterns, high-resolution time-of-flight mass spectrometry

## Abstract

Zotarolimus (ABT-578) is a sirolimus derivative that, like sirolimus and everolimus, is an inhibitor of cell growth via inhibition of the mechanistic target of rapamycin (mTOR). Zotarolimus was developed for coating coronary stents to prevent smooth muscle cell proliferation and restenosis. Albeit zotarolimus-eluting cardiovascular devices have been on the market for years, details of zotarolimus drug metabolism in humans are still unknown. Hence, it was the goal of the present study to identify zotarolimus metabolites generated by incubation with human liver microsomes. Metabolite structures were identified using high-resolution mass spectrometry, MS/ion-trap (MS^n^), and comparison of fragmentation patterns of the metabolites with those of zotarolimus and other known sirolimus derivatives. Kinetic parameters such as incubation time, human liver microsomal protein concentrations, and drug concentrations were optimized before scaling up the metabolism experiments. Human liver microsomes mainly hydroxylated and/or demethylated zotarolimus. The structures of the following metabolites were identified: O-demethylated metabolites: 39-O-desmethyl, 16-O-desmethyl, and 27-O-desmethyl zotarolimus; hydroxylated metabolites: hydroxy piperidine zotarolimus, 11-hydroxy, 12-hydroxy, 14-hydroxy, 23-hydroxy, 24-hydroxy, 25-hydroxy, 45/46-hydroxy, and 49-hydroxy zotarolimus; demethylated-hydroxylated metabolites: 16-O-desmethyl, 23/24-hydroxy; 39-O-desmethyl, 23/24-hydroxy; 39-O-desmethyl, 25-hydroxy zotarolimus; 39-O-desmethyl, 11-hydroxy zotarolimus; 39-O-desmethyl, hydroxy-piperidine zotarolimus; 27-O-desmethyl, 45/46-hydroxy zotarolimus; didemethylated metabolites: 16,39-O-didesmethyl zotarolimus; 16,27-O-didesmethyl zotarolimus; 27,39-O-didesmethyl zotarolimus; and dihydroxylated metabolites: 11,24-dihydroxy zotarolimus, 12,24-dihydroxy zotarolimus, and 11,47/48-dihydroxy zotarolimus. It is concluded that zotarolimus is extensively metabolized by human liver microsomes. Twenty-four of these metabolites could be structurally identified using a combination of ion-trap MS^n^ and high-resolution mass spectrometry.

## 1. Introduction

**Figure 1:**
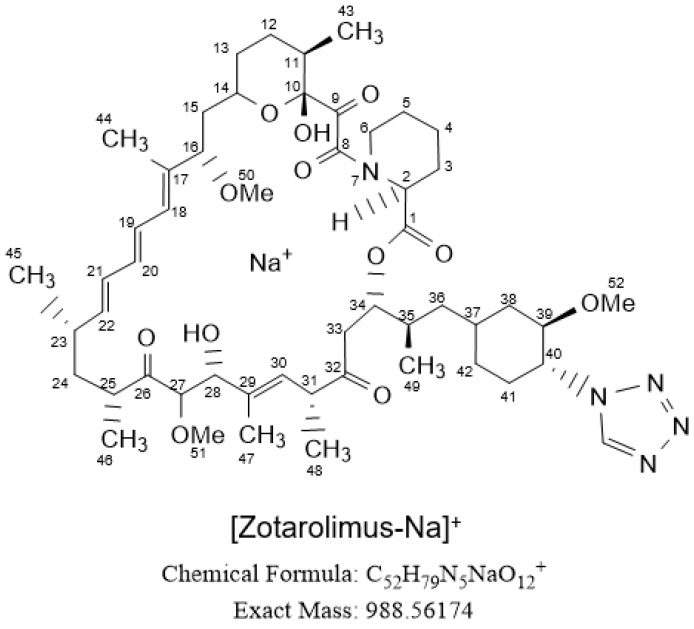
Structure of zotarolimus. The numbering follows the IUPAC (International Union of Pure and Applied Chemistry, www.iupac.org) nomenclature.

Zotarolimus (ABT-578, Abbott Pharmaceuticals, Abbott Park, IL, USA) is a semi-synthetic sirolimus (rapamycin) derivative (C_52_H_79_N_5_O_12_, (42S)-42-Deoxy-42-(1H-tetrazol-1-yl)-rapamycin, Figure 1) that, like sirolimus and everolimus, is an inhibitor of the mechanistic target of rapamycin (mTOR) [1,2,3]. As such, it arrests cell proliferation between the G1- and S-phase, including the proliferation of coronary smooth muscle cells [3]. While everolimus and sirolimus were first developed as immunosuppressants after organ transplantation [1], zotarolimus was specifically developed for coating as a proliferation inhibitor on cardiovascular devices [2]. Zotarolimus is lipophilic with a much higher octanol:water partition coefficient than sirolimus, which was expected to result in more sustained release from the stent-coating matrix and in an easier diffusion through the membrane of the target tissue [2]. Zotarolimus has been used as a proliferation inhibitor on several coronary stent systems such as ZoMaxx (Abbott Vascular, Santa Clara, CA, USA), Endeavor, and Resolute (both Medtronic CardioVascular, Santa Rosa, CA, USA). A study compared de novo patients with at least 8–9 month follow up with intravascular ultrasound analyses in the ZoMaxx I, Endeavor II, and Resolute trials and found that the Resolute coronary stent system had significantly less neointimal coverage than the other zotarolimus-eluting stents [4]. It was discussed that this was due to the unique Medtronic BioLinx C10/C19/PVP polymer used for coating the Resolute stent that enables much longer, sustained zotarolimus elution [4,5]. Of the aforementioned zotarolimus-eluting coronary stents, only the Resolute stent is on the market today with its latest version, the Resolute Onyx stent, approved by the United States Food and Drug Administration in September 2020 [6]. Clinical trials have almost consistently shown that the Resolute zotarolimus-eluting stent has 1-year [7] and 5-year outcomes comparable to everolimus-eluting coronary stents [8,9,10] and second-generation sirolimus-eluting stents [10].

Although zotarolimus-eluting cardiovascular devices have been on the market for more than 15 years, there are no publications about its drug metabolism. The only information that can be found is in the Instructions for Use for the zotarolimus coronary stent system [11]. The information provided is limited to a generalized statement that zotarolimus undergoes oxidative metabolism in the liver; that hydroxylated, demethylated, hydroxy-demethylated, and dihydroxy-demethylated metabolites are generated; and that cytochrome P4503A is responsible for its metabolism [11]. No further details regarding the metabolite structures are provided. Drug-coated balloons are increasingly used for peripheral vascular disease and due to its lipophilicity, zotarolimus is a promising drug candidate [12]. After coronary stent implantation, the systemic exposure of zotarolimus, and most likely also that of its metabolites, is very low and in the pg/mL concentration range [11]. This may be very different after deployment of peripheral zotarolimus-coated balloons, which can be expected to have a much higher total drug load than coronary stents. In the present study, as a first step to assess the potential pharmacodynamic and toxicodynamic effects of the zotarolimus metabolites, we revisited the metabolism of zotarolimus by pooled human liver microsomes to identify their structures. Due to the instability of sirolimus derivatives in pure organic solvents, often incomplete chromatographic separation and, thus, the inability to isolate pure metabolite fractions, as well as the low concentrations of most metabolites, structural identification by nuclear magnetic resonance (NMR) spectroscopy is difficult or even impossible [13]. In comparison to NMR spectroscopy, the strengths of mass spectrometry are its vastly superior sensitivity and that it allows for structural identification of drug metabolites in complex mixtures [14,15]. Therefore, we had identified the detailed fragmentation patterns of sirolimus, everolimus, and their metabolites using a combination of ion-trap (MS^n^) and high-resolution time-of-flight (TOF) mass spectrometry [13]. This strategy was previously used successfully to identify the metabolite structures of the sirolimus derivatives temsirolimus and SAR-943 after incubation with human liver microsomes [16,17] and is now used in the present study to identify the structures of twenty-four human phase-I zotarolimus metabolites.

## 2. Materials and Methods

### 2.1. Enzymes, Reagents, and Chemicals

Acetonitrile, methanol, water (all HPLC-grade), formic aid, and dichloromethane were from Fisher Scientific (Fair Lawn, NJ, USA) and zotarolimus was from Toronto Research Chemicals (North York, ON, Canada). Human liver microsomes (pooled from 200 individuals) were purchased from Xenotech (Kansas City, KS, USA). Reduced nicotinamide adenine dinucleotide phosphate (NADPH) and other chemicals for the NADPH-generating system (see below) were purchased from Sigma Aldrich (St. Louis, MO, USA).

One mg/mL zotarolimus stock solutions in 0.1% formic acid/acetonitrile 20/80 volume/volume (*v*/*v*) were prepared. Stock solutions, working solutions, and isolated metabolite fractions were kept in a −80 °C freezer.

### 2.2. Incubation of Zotarolimus with Pooled Human Liver Microsomes for the Generartion of Metabolites

The following was pre-incubated in a dry bath at 37 °C for 5 min: an NADPH-generating system in Na^+^/K^+^-phosphate buffer (pH 7.4, 0.1 mol/L), 0.5 mg microsomal protein (0.5 mg), and 20 µmol/L zotarolimus. The dry bath was a type 16,500 Dri-Bath from Barnstead Thermolyne (Dubuque, IA, USA). The NADPH-generating system contained the following: NADP (1 mmol/L), isocitric acid (25 mmol/L), isocitrate dehydrogenase (1750 U/L), MgCl_2_ (10 mmol/L), and ethylenediaminetetraacetic acid (EDTA, 5 mmol/L). To start the reaction, 0.5 mL NADPH in 0.1 mM Na^+^/K^+^-phosphate buffer pre-warmed to 37 °C was added. The incubation time was 40 min. Then, 0.5 mL ice-cold acetonitrile was added to stop the reaction via protein precipitation. Samples were centrifuged at 4 °C and 25,000× *g* for 10 min. The supernatants were further processed as described below. The following controls were run (n = 5 each): incubation without drug, incubation without human liver microsomes, and incubation without the NADPH-generating system. The aforementioned components were replaced by the corresponding volumes of 0.1 mM Na^+^/K^+^-phosphate buffer. Otherwise, the incubation conditions were as described above. Representative ion chromatograms of the extracts of the controls are shown on pages 211 and 212 of the Supplementary Materials.

### 2.3. Isolation of Zotarolimus Metabolites

**Figure 2:**
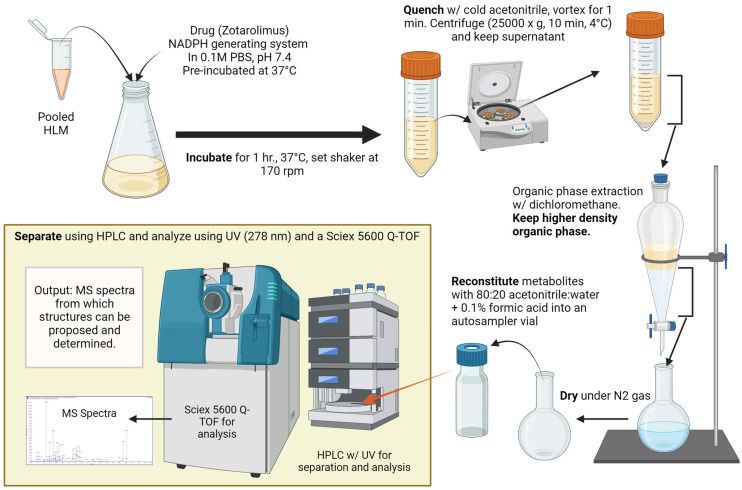
Experimental workflow.

To generate the amounts of minor metabolites required for structural identification (>5 µg), the incubations with human liver microsomes were scaled up (Figure 2). At least 1000 samples of human liver microsomes (each 0.5 mL) were prepared, incubated, and, after protein precipitation, combined. The supernatants combined after protein precipitation and centrifugation were transferred into a 1 L separating glass funnel and extracted using dichloromethane. After vigorously shaking for 5 min and phase separation, the organic phase was separated and dried down under a flow of nitrogen at 37 °C. The residue was reconstituted in 1.5 mL 0.1% formic acid/acetonitrile 1/4 *v*/*v* and was transferred into 1.8-mL glass HPLC vials (Agilent Technologies, Santa Clara, CA, USA). Fractions containing individual metabolites were isolated using semi-preparative high-performance liquid chromatography in combination with ultra-violet detection (HPLC/UV) at 278 nm. The semi-preparative HPLC/UV system consisted of the following components: G1361A LC pump, G2260A/G1330B thermostatted autosampler, G1315B diode array detector (DAD) (all Agilent 1200, Agilent Technologies), and a TS-430 column thermostat compartment (Phenomenex, Torrance, CA, USA). A total of 150 µL of the extracts was injected into the HPLC system. Zotarolimus and its metabolites were separated on 4250 × 6.4 mm analytical columns filled with Zorbax Eclipse XDB-C8 material of 5 μm particle size (Agilent Technologies) connected in series. The columns were kept at a temperature of 65 °C. Mobile phase A was water with 0.1% formic acid and mobile phase B was acetonitrile with 0.1% formic acid. The following gradient was run (flow rate: 4 mL/min): 0–1 min: 45/55 (A/B) *v*/*v*, 2.1–20 min: from 45/55 to 40/60 *v*/*v*, from 20.1–69 min: from 40/60 to 27/73 *v*/*v*, from 61.1 to 76 min: from 27/73 to 5/95 *v*/*v*. The gradient was kept at 5/95 *v*/*v* to wash the columns for 7 min. Then, the gradient returned to the starting conditions (45/55 *v*/*v*) and the columns were re-equilibrated for 7 min. The total run time was 90 min. More than 30 fractions were automatically collected using a fraction collector (Agilent Technologies). The corresponding isolated fractions were pooled and extracted using dichloromethane. After drying the extracts under nitrogen flow at 37 °C, the residues were reconstituted in 0.5 mL water with 0.1% formic acid/acetonitrile (20/80, *v*/*v*). All extracts were analyzed immediately using the HPLC-MS/TOF system as described below. The remaining extracts were kept at −70 °C and below for further analysis.

### 2.4. Instrumentation

The generated metabolites mixture as well as individual isolated metabolites of zotarolimus were measured using analytical HPLC/MS-TOF consisting of the following 3000 Ultima HPLC components (all Dionex, Thermo Fischer, Palo Alto, CA, USA): WPS-3000 (RS) autosampler, DGP-3600 Pump, and FLM-3000 Flow Manager oven. The HPLC system was controlled by Chromeleon software version 6.8.

Zotarolimus and its metabolites were analyzed using a 5600+ high-resolution triple TOF mass spectrometer (Sciex, ON, Canada) operated in the positive electrospray ionization (ESI) mode. The instrument was controlled, and data were acquired and analyzed using Sciex Analyst Software (version TF1.7.1).

### 2.5. HPLC and Mass Spectrometry Conditions

A total of 10 µL of the generated metabolite extract was injected onto three 250 × 4.5 mm analytical columns (Zorbax Eclipse XDB-C8 columns of 5 μm particle size, Agilent Technologies, Palo Alto, CA, USA) connected in series. The columns were kept at 65 °C.

Zotarolimus and its metabolites were separated using a water (A)/acetonitrile (B) gradient, both with 0.1% formic acid (flow rate: 1 mL/min). The gradient elution program was as follows: 0–2 min: 30/70 (A/B) *v*/*v*, 2.0. to 65 min: 30/70 to 14/86 *v*/*v*, 65.0 to 67 min: 14/86 to 2/98 *v*/*v*. Then, the gradient was kept at 2/98 *v*/*v* to wash the columns for 8 min and returned to the starting conditions of 30/70 *v*/*v*. The columns were re-equilibrated at this mobile phase composition for 10 min. Each run took a total of 85 min.

MS/TOF mass spectra were acquired in the scan mode over a mass range of *m*/*z =* 250–1200 as well as in product ion mode for each specific metabolite modification (hydroxy zotarolimus *m*/*z =* 1004.55, desmethyl zotarolimus *m*/*z =* 974.54, didesmethyl zotarolimus *m*/*z =* 960.53, hydroxy-desmethyl zotarolimus *m*/*z =* 990.54, dihydroxy zotarolimus *m*/*z =* 1020.55). The optimization of MS parameters was performed by direct infusion of zotarolimus stock solution (100 ng/mL) into the ESI source of the mass spectrometer via the Sciex 5600+ instrument’s internal syringe pump at a flow of 10 µL/min. The optimized ESI-MS/TOF parameters were as follows: ion spray voltage 5000 V; curtain gas 32 psi; nebulizer gas (N2) 60 psi; heater gas (N2) 50 psi; source temperature 600 °C; delustering potential (DP) 135 V; collision energy (CE) 65 eV. Ions [M+Na]^+^ were detected in the positive mode using a mass resolution of 30,000 (FWHM).

### 2.6. Identification of Zotarolimus Metabolite Structures

High-resolution TOF mass spectra in positive scan mode (scan range *m*/*z =* 250 to 1200) as well as ion product mode were recorded after HPLC separation of zotarolimus and its metabolites. The structures of the zotarolimus metabolites were identified based on the MS/MS fragmentation patterns, which were verified by (a) comparison to structurally identified derivatives (sirolimus, everolimus, temsirolimus, and SAR943, and their metabolites) [13,16,17,18], (b) exact mass determination using the ESI TOF high-resolution mass spectrometer in combination with comparison of the measured and theoretical exact mass, as well as (c) elucidation of the relationships of the fragments (ion-trap/mass spectrometry, up to MS^3^) [13].

The detailed fragmentation pattern of zotarolimus and its isolated metabolites was confirmed in scan mode (*m*/*z =* 250–1200) during infusion of a 100 ng/mL zotarolimus solution or of the isolated metabolite fractions. These solutions were infused into the Sciex 5600+’s electrospray ionization (ESI) source via the 5600+’s internal syringe pump. The infusion rate was set to 10 µL/min or as required to result in a good signal. Infusion-ESI-MS^n^ ion-trap analysis of zotarolimus showed more than 50 major fragments, the structures of which were identified. These hypothetical structures were then confirmed by high-resolution TOF mass spectrometry. Hereafter said structurally identified fragments were used to identify the structures of 24 zotarolimus metabolites generated after incubation of zotarolimus with pooled human liver microsomes as described above.

### 2.7. Data Analysis

We employed Sciex Analyst Software (version TF1.7.1) for data analysis. The structural analysis and assessment of calculated mass errors was conducted using a combination of ChemDraw (version 19.0, Perkin Elmer, Waltham, MA, USA) and Excel (version 365, Microsoft, Redmond, WA, USA).

## 3. Results

### 3.1. HPLC-MS of Zotarolimus

Like for the structurally related sirolimus and everolimus, sodium adducts [M+Na]^+^ *m*/*z =* 988.56174 were the major detected peaks of zotarolimus in the positive ionization mode, among other minor ions such as [M+H]^+^ and [M+K]^+^. Representative mass spectra are shown in the Supplementary Materials. Extraction recoveries of zotarolimus metabolites after isolation by semi-preparative HPLC were evaluated using ethylacetate and dichloromethane. The calculated extraction recoveries of zotarolimus were 92% ± 5% using dichloromethane vs. 85% ± 9% using ethylacetate. To assess the potential degradation products of zotarolimus during the extraction process, the concentration of zotarolimus before and after extraction was determined using HPLC-UV and external calibration curves of zotarolimus. No degradation of zotarolimus, especially via ester hydrolysis of the macrolide ring, was observed during sample extraction.

### 3.2. Intentification of Zotarolimus Fragments and Their Relationships

**Figure 3:**
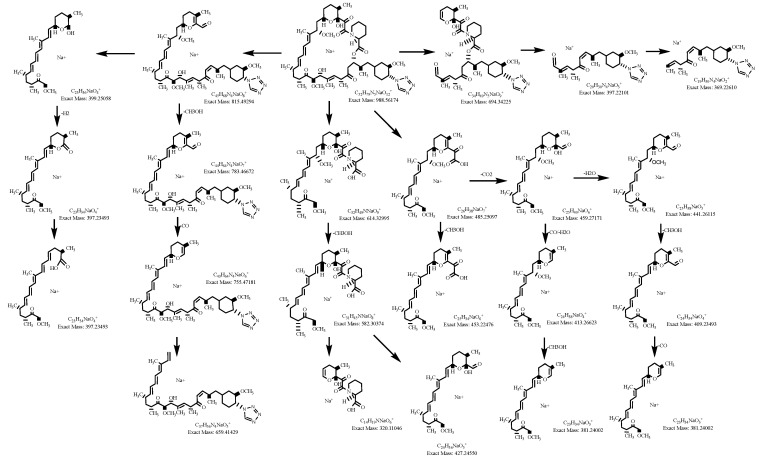
Major fragments of zotarolimus and their relationships as assessed using ion trap MS^n^. This scheme only shows fragments that were used for the elucidation of the zotarolimus metabolite structures.

**Figure 4:**
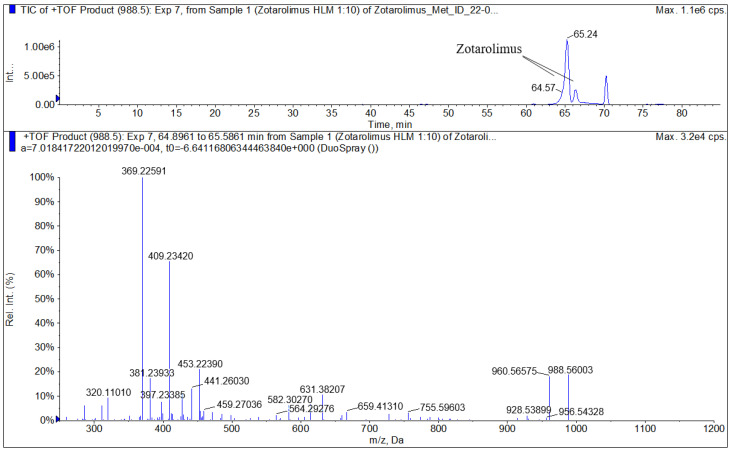
Representative highresolution MS/MS spectrum of zotarolimus recorded using a TOF mass spectrometer (5600+, Sciex, Concord, ON, Canada).

The zotarolimus fragmentation patterns were compared with the fragmentation patterns of sirolimus, everolimus, SAR-943, and temsirolimus that we had identified in previous studies [13,16,17,18]. As shown in Figure 3 and Figure 4, all zotarolimus fragments were also mainly detected as [M+Na]^+^. The Figure 4 also shows the fragmentation pathways of zotarolimus as proposed based on the ion-trap MS^n^ studies. Only those major fragments that were used to identify the structures of the zotarolimus metabolites are included. The major fragments were the result of α-cleavage. This also matched our observations with sirolimus, everolimus, SAR-943, and temsirolimus [13,16,17,18,19]. As aforementioned, after determination of the exact mass of the fragments using MS/TOF, the theoretical exact masses of the hypothetical fragment structures were compared with the corresponding exact measured masses to verify the fragment structures (Figure 4 and Table 1).

### 3.3. Metabolism of Zotarolimus by Human Liver Microsomes

In a first step, the dependency of zotarolimus metabolite formation kinetics on incubation time and the amount of microsomal protein was assessed with the goal to optimize yield. After incubation and extraction, the metabolites were quantified using the HPLC-MS/high-resolution TOF system. It was found that for incubation periods of up to 60 min, the amount of zotarolimus metabolites generated increased almost linearly. The same applied to microsome protein concentrations ranging between 0.1 and 1.5 mg/mL. Based on these results, in the next step, zotarolimus metabolite production was upscaled and zotarolimus was incubated with 0.5 mg/mL microsomal protein for 40 min.

### 3.4. Identification of the Structures of Zotarolimus Metabolites Generated by Human Liver Microsomes

Under these incubation conditions, at the end of the 40 min incubation period, 24 zotarolimus metabolites could be isolated and their structures identified (Table 2). As mentioned above, this was achieved by comparison of their fragmentation patterns with those of zotarolimus and those of structurally related, corresponding sirolimus, everolimus, temsirolimus, and SAR-943 metabolites which were available to us as reference material. These metabolites had been generated and structurally identified as previously described by us [13,16,17,18]. In several cases (please see also the Supplementary Materials), the formation of additional fragments that were unique for specific metabolites was informative [19]. Thus, the formation of additional hydroxy groups during the metabolism reactions enabled α-cleavage of adjacent C-C bonds. In the case of zotarolimus metabolites hydroxylated in the region between C(11) and C(14), the aforementioned additional fragments were critical for the identification of the hydroxylation positions. The characteristic fragments of those metabolites were proposed based on Retro-Diels-Alder cleavage. The structural identification of all 24 metabolites is described in detail in the Supplementary Materials. This includes the results of the ion-trap MS^n^ analysis of the isolated metabolites as well as the high-resolution MS data. In the following, as representative examples, the identification of the three demethylated and the two major hydroxylated zotarolimus metabolites is discussed.

#### 3.4.1. Demethylated Zotarolimus Metabolites

As also known from the structurally related sirolimus, everolimus, SAR-943, and temsirolimus [13,16,17,18], the major demethylated zotarolimus metabolites are formed by O-demethylation of the methoxy groups at C(16), C(27) and C(39).

#### 3.4.2. 16-O-Desmethyl Zotarolimus

**Figure 5:**
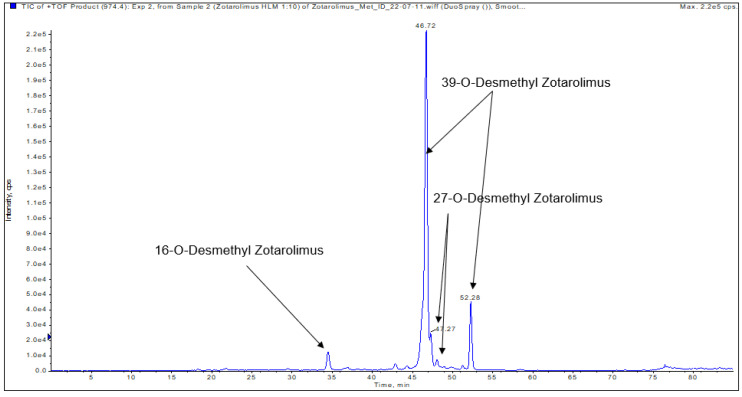
Representative extracted ion chromatogram (EIC) of demethylated zotarolimus metabolites (*m*/*z* 974.54609).

**Figure 6:**
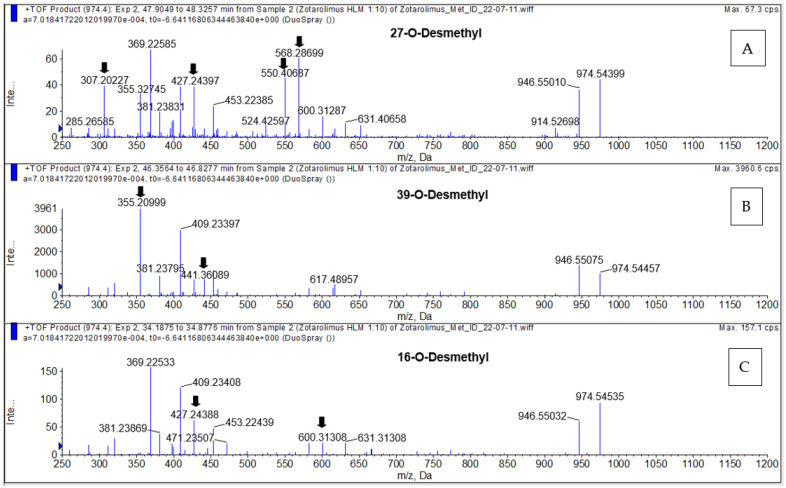
Representative MS/TOF high-resolution mass spectra of the demethylated zotarolimus metabolites (*m*/*z* 974.54609). 27-O-desmethyl zotarolimus (**A**); 39-O-desmethyl zotarolimus (**B**); 16-O-desmethyl zotarolimus (**C**). The arrows mark key fragments used for structural identification. For more details, please see the Supplementary Materials.

The compounds underlying the metabolite peaks with the main peaks having retention times of 34.85, 46.72, and 47.27 min (Figure 5) showed a molecular ion [M+Na]^+^ of *m*/*z =* 974.54609. This indicated demethylation of zotarolimus ([M+Na]^+^ *m*/*z =* 988.56174). The high-resolution mass spectrum of the metabolite with a retention time of 34.85 min is shown in Figure 6C. Comparison of the mass spectra of the metabolite underlying this peak with that of zotarolimus showed that the metabolite with the retention time of 34.85 min had fragments excluding O-demethylation at C(31): *m*/*z =* 369, 397, and 694 (the same as the corresponding zotarolimus fragments), whereas specific fragments confirming the presence of 16-O-desmethyl zotarolimus (*m*/*z =* 381, 409, 453, 928, and 755) were present in the MS/MS spectra of this metabolite (Figure 6C, for more detail please see page 104 of the Supplementary Materials). Furthermore, water elimination at the C(16) position enabled by the newly formed hydroxy group (*m*/*z =* 600 → 582, 471 → 453, and 427 → 409) confirmed 16-O-desmethyl zotarolimus. The calculated mass error (Δppm) for the observed key fragments were consistently below 3.8 ppm (please see Table 1 and the Supplementary Materials) indicating high mass accuracy of the proposed fragment structures and, thus, a high level of certainty of the structural identification of 16-O-desmethyl zotarolimus. Using a 5600+ high-resolution TOF mass spectrometer (Sciex, ON, Canada) with a resolution of 30,000 and an absolute mass error of <5 ppm allowed for confirmation of the proposed elemental composition of the fragment ions.

#### 3.4.3. 27-O-Desmethyl Zotarolimus

The isolated metabolite fractions (peak retention times: 47.27 and 47.30 min) (Figure 5) had a molecular ion *m*/*z =* 974.54609 ([M+Na]^+^). This indicated demethylation of zotarolimus ([M+Na]^+^, *m*/*z* 988.56174). Figure 6A shows the high-resolution mass spectrum of this metabolite. Comparison of the mass spectra recorded for this peak (27-O-desmethyl zotarolimus) with that of 16-O-desmethyl zotarolimus revealed that most fragments in both metabolites are identical except *m*/*z =* 307, 383, 395, 550, and 568 which indicate demethylation at C(27). The metabolite peak with retention times of 47.27 and 47.30 min also showed the five specific fragments confirming the presence of 27-O-desmethyl zotarolimus: *m*/*z =* 307, 383, 395, 550, and 568 which are the results of α-cleavage of adjacent C-C bonds enabled by the formed hydroxy group in the region between C(27) and C(28) (Figure 6A, for more detail please see the Supplementary Materials). With a highest calculated mass error (Δppm) for the aforementioned key fragments of 3.8 ppm indicating high mass accuracy of the proposed fragment structures (please see Table 1 and the Supplementary Materials), the structure of this metabolite is identified as 27-O-desmethyl zotarolimus.

#### 3.4.4. 39-O-Desmethyl Zotarolimus

The isolated metabolite fractions (peak retention times: 46.72 min and 52.28 min, Figure 5) had a molecular ion *m*/*z =* 974.54609 ([M+Na]^+^). This indicated demethylation of zotarolimus ([M+Na]^+^, *m*/*z* 988.56174). Given that the mass spectra for both peaks with the retention times of 47.62 and 52.28 min were identical, they probably correspond to the same compound. The differences in retention time are likely due the presence of two main conformers of the molecule that are chromatographically separated as also observed for zotarolimus (Figure 4).

Figure 6B shows the high-resolution mass spectrum of this metabolite. In comparison to the mass spectra of the present metabolite (39-O-desmethyl zotarolimus), 16-O-desmethyl zotarolimus and 27-O-desmethyl zotarolimus showed significant differences in fragmentation patterns. There were several fragments excluding the presence of 16-O-desmethyl and 27-O-desmethyl zotarolimus, including *m*/*z =* 485, 459, 441 and 614 (Figure 6B, for more details please see the Supplementary Materials).

#### 3.4.5. Hydroxylated Zotarolimus Metabolites

In addition to these three demethylated zotarolimus metabolites, several hydroxy metabolites, didemethylated and dihydroxylated, as well as demethylated-mono-hydroxylated metabolites were detected (Table 2 and Supplementary Materials).

In contrast to only three possible demethylation positions, there are several potential hydroxylation positions. As it was shown in our previous studies, the major sites of hydroxylation in the structurally related sirolimus, everolimus, SAR-943, and temsirolimus are C(11), C(12), C(14), C(23), C(24), C(25), C(45), C(46), and C(48) [13,16,17,18], whereas O-demethylation was limited to the methoxy groups at C(16), C(27), and C(39). As representative examples, the structural identification of the major hydroxylated zotarolimus metabolites 45-, 46-hydroxy- and 25-hydroxy- zotarolimus is discussed below; for all others, please see the Supplementary Materials.

#### 3.4.6. 45 and 46-Hydroxy Zotarolimus

**Figure 7:**
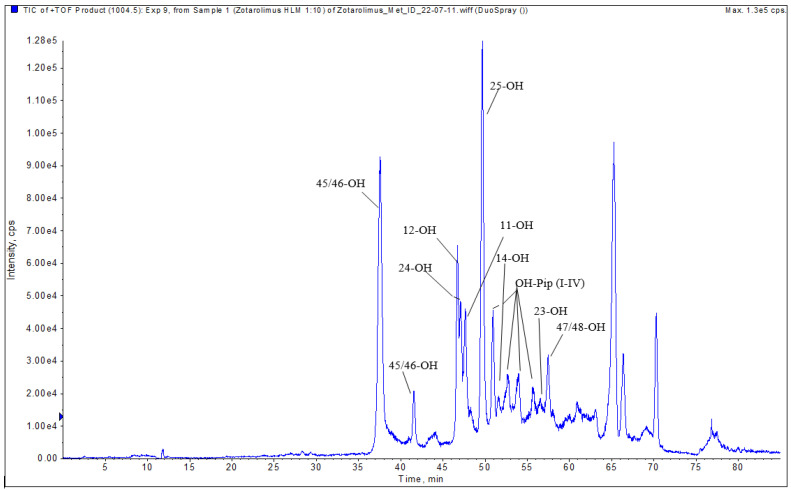
Representative extracted ion chromatogram (EIC) of hydroxylated zotarolimus metabolites (*m*/*z* 1004.55666).

**Figure 8:**
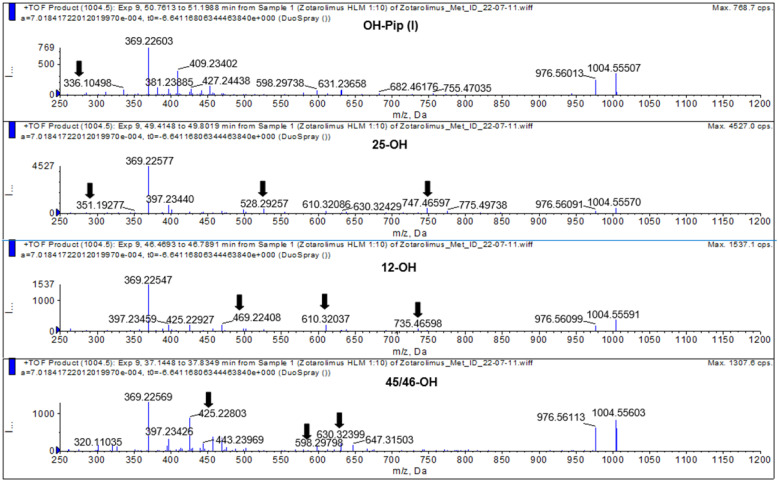
Representative mass spectra of hydroxylated zotarolimus metabolites (*m*/*z* 1004.55666), hydroxy-piperidine-, 25-hydroxy-, 12-hydroxy-, and 45/46-hydroxy-zotarolimus. The arrows mark key fragments used for structural identification. For more details, please see the Supplementary Materials.

The isolated metabolite fractions (peak retention times: 37.3 and 41.5 min, Figure 7) had a molecular ion *m*/*z =* 1004.55603 ([M+Na]^+^). This indicated hydroxylation of zotarolimus ([M+Na]^+^, *m*/*z* 988.56174). Both detected peaks showed similar fragmentation patterns. As both positions C(45) and C(46) can theoretically be hydroxylated and no difference in their fragmentation pattern could be observed, it was impossible to distinguish between 45-hydroxy- and 46-hydroxy-zotarolimus. However, the hydroxylation at C(45) or C(46) can result in the subsequent loss of methanol, which could be observed for both detected peaks at 37.3 and 41.5 min (*m*/*z* = 630 – 32 = 598, *m*/*z* = 457 – 32 = 425, *m*/*z* = 501 – 32 = 469) (Figure 8). The highest calculated mass error (Δppm) for the fragments used for the identification of these metabolites was 4.1 ppm (please see Table 1 and the Supplementary Materials) indicating high mass accuracy of the proposed structures.

#### 3.4.7. 25-Hydroxy Zotarolimus

The isolated metabolite fraction (peak retention times: 49.80 min, Figure 7) had a molecular ion *m*/*z =* 1004.55570 ([M+Na]^+^). This indicated hydroxylation of zotarolimus ([M+Na]^+^, *m*/*z* 988.56174). In comparison to zotarolimus, most metabolite fragments including the 25-hydroxy group had a +16 Da difference, indicating the addition of a hydroxy group. The key fragments supporting the structure of 25-hydroxy zotarolimus were *m*/*z =* 351, 528, 747, and 904 (Figure 7). Most importantly, as also described for 25-hydroxy sirolimus [19] and 25-hydroxy SAR 943 [17], hydroxylation facilitated α-cleavage of adjacent C-C bonds. This resulted in a fragment *m*/*z =* 351.19277 which is characteristic for 25-hydroxy zotarolimus and that did not have a corresponding zotarolimus fragment (Figure 8). For more details, please see the Supplementary Materials.

## 4. Discussion

Zotarolimus, a semi-synthetic derivative of sirolimus, is a potent antiproliferative agent used in drug-eluting stents. It differs from sirolimus by having a C(40) tetrazole substituent adjacent to the C(39) methoxy group at the cyclohexyl ring with differing stereochemistry. As a result, zotarolimus is more lipophilic, allowing for more rapid uptake into the target tissue than sirolimus, and it has a shorter half-life [2,3]. One of the major challenges in zotarolimus drug metabolism studies is the structural identification of the generated metabolites, especially in cases where many structurally closely related metabolites in low quantities are generated. Nuclear magnetic resonance (NMR) spectroscopy is considered the “gold standard” analytical method for the structural identification of unknown metabolites. It enables identification of minor modifications in different positions of a molecule (constitutional isomers) and/or of the same modification at the same position but with different special orientation (stereoisomers e.g., enantiomers or diastereomeres). However, in comparison to mass spectrometry, NMR’s sensitivity is orders of magnitude lower, and it requires high purity of the target analyte(s). As shown in the present study, metabolism of zotarolimus by human liver microsomes results in more than 24 metabolites, most of which at low concentrations, which are difficult to separate by HPLC (e.g., Figure 5 and Figure 7) and, thus, cannot be isolated with the purity required for structural NMR analysis. One of the challenges with complete chromatographic separation of the zotarolimus metabolites is that, like the parent compound, they elute in a double peak pattern due to the formation of rotamers around N(7). Another limiting factor in terms of NMR spectroscopy for identification of sirolimus, and its derivatives and metabolites, is instability in pure organic solvents [20]. This is important as due to the low amounts of most zotarolimus metabolites formed, long NMR scan times would be required. To overcome the above-mentioned limitations, the use of mass spectrometry combined with liquid chromatography has previously been reported by Boernsen et al. [13]. Based on a detailed analysis of sirolimus and everolimus MS/MS fragmentation patterns, Boernsen et al. described the sensitive, complete structural identification of their metabolites. To avoid misinterpretation of the fragmentation data such as reported by Lhoëst et al. [13,21], verification of the fragment structures by high-resolution mass spectrometry and comparison of the measured and theoretical exact masses of the hypothetical fragment structures is critical. Several previous publications [19,22,23,24,25] which postulated metabolite structures of sirolimus and its derivatives based their analysis only on a limited set of MS/MS fragments. In contrast, we relied on a comprehensive set of fragments, the structures of which had been confirmed by ion-trap MS^n^ and TOF high-resolution mass spectrometry.

The acquired MS-TOF spectra of zotarolimus and its metabolites in positive ion mode showed several adduct ions with sodium adducts having the highest intensity. This had also been observed in other studies with structurally related drugs such as sirolimus, everolimus, temsirolimus, and SAR-943 [13,16,17,18,19]. Additives, such as formic acid used in the mobile phase, may have facilitated the formation of the preferred sodium adducts. Moreover, the addition of ammonium formate or ammonium acetate without formic acid did not suppress the formation of sodium adduct ions.

The present study showed that the biotransformation of zotarolimus by pooled human liver microsomes resulted in more than 24 metabolites. The major metabolites identified were demethylated (39-O-desmethyl zotarolimus) and hydroxylated (45-, 46-hydroxy and 25-hydroxy zotarolimus), both first-generation metabolites: this means metabolites formed by metabolism of the parent compound. In addition, several minor metabolites resulting from a combination of demethylation and hydroxylation, as well as di-demethylation and di-hydroxylation, so-called second-generation metabolites, were identified. In addition to the 24 structurally identified metabolites, several other demethylated-hydroxylated and di-hydroxylated metabolites were detected. However, based on the detected fragments, it became clear that more than one metabolite was underlying the corresponding peaks and the individual structures could not be identified. The high chromatographic resolution, as well as high sensitivity achieved by high-resolution TOF mass spectrometry in combination with scale-up of metabolite generation, enabled us to identify even minor metabolites of zotarolimus.

## 5. Conclusions

The metabolite profile of zotarolimus observed after incubation with pooled human liver microsomes in vitro was similar to that of the structurally related sirolimus and other derivatives of sirolimus such as everolimus, SAR-943, and temsirolimus that had previously been studied by our group [13,16,17,18]. Our study provides a road map for the generation, isolation, and structural elucidations of the metabolites of other sirolimus derivatives, the metabolism of which has not yet been published, such as biolimus and novolimus [26,27]. The isolated, structurally identified zotarolimus metabolites will be used in future studies as reference materials for the development and validation of quantitative LC-MS/MS assays for pre-clinical and clinical pharmacokinetics and toxicokinetics studies, as well as for the assessment of their biological activity in in vitro assays.

## Figures and Tables

**Table 1:** Comparison of the calculated and measured exact masses of key zotarolimus fragments used to identify the metabolite structures. A 5600+ high-resolution TOF mass spectrometer (Sciex, Concord, ON, Canada) was used to measure the exact masses. Figure 4 shows a representative high-resolution mass spectrum.

Fragment No.	Theoretical Mass	Measured Mass	Δppm
1	320.11046	320.11010	1.1
2	369.22610	369.22591	0.5
3	381.24002	381.23933	1.8
4	397.23493	397.23385	2.7
5	409.23493	409.23420	1.8
6	427.24550	427.24491	1.4
7	441.26115	441.26030	1.9
8	453.22476	453.22390	1.9
9	582.30374	582.30270	1.8
10	614.32995	614.32890	1.7
11	659.41429	659.41310	1.8
12	694.34225	694.34140 (low intensity)	1.2
13	783.46672	783.46540 (low intensity)	1.7
14	815.49294	815.49190 (low intensity)	1.3
15	988.56174	988.56003	1.7

**Table 2:** Overview of the zotarolimus metabolites that were identified. Please see the Supplementary Materials for more detail. Four distinct hydroxy piperidine metabolites were detected. These were chromatographically separated indicating hydroxylation at different positions of the piperidine ring. However, since no fragments of the piperidine ring were detected, the exact hydroxylation positions could not be identified.

	Desmethyl Metabolites	Exact Mass
1	16-O-Desmethyl Zotarolimus	974.54609
2	27-O-Desmethyl Zotarolimus	
3	39-O-Desmethyl Zotarolimus	
	**Di-Desmethyl Metabolites**	
4	16, 39-O-Didesmethyl Zotarolimus	960.53044
5	27, 39-O-Didesmethyl Zotarolimus	
6	16, 27-O-Didesmethyl Zotarolimus	
	**Hydroxy Metabolites**	
7	45 and 46-Hydroxy Zotarolimus	1004.55666
8	24-Hydroxy Zotarolimus	
9	11-Hydroxy Zotarolimus	
10	12-HydroxyZotarolimus	
11	25-Hydroxy Zotarolimus	
12	3/4/5/6 Hydroxy-piperidine Zotarolimus	
13	23-Hydroxy Zotarolimus	
14	14-Hydroxy Zotarolimus	
15	49-Hydroxy Zotarolimus	
	**Hydroxy, Desmethyl Metabolites**	
16	23/24-hydroxy, 16-O-desmethyl Zotarolimus	990.54101
17	23/24-hydroxy, 39-O-desmethyl Zotarolimus	
18	25-hydroxy, 39-O-desmethyl Zotarolimus	
19	11-hydroxy, 39-O-desmethyl Zotarolimus	
20	Hydroxy-piperidine, 39-O-desmethyl Zotarolimus	
21	45/46-hydroxy, 27-O-desmethyl Zotarolimus	
	**Di-Hydroxy Metabolites**	
22	11,24-Dihydroxy Zotarolimus	1020.55157
23	12,24-Dihydroxy Zotarolimus	
24	11,47/48-Dihydroxy Zotarolimus	

## Data Availability

Data reported in this study is contained within the article and Supplementary Materials. The underlying raw data is available on request from the corresponding author. The raw data are not publicly available due to the complexity and amount of data which requires special software for processing.

## Supplementary Materials

The following supporting information can be downloaded at: https://www.mdpi.com/article/10.3390/metabo13101093/s1, Supplemental Digital Contents.
