# Supplementary material for: Structural Identification of Zotarolimus (ABT-578) Metabolites Generated by Human Liver Microsomes Using Ion-Trap and High-Resolution Time-of-Flight Mass Spectrometry in Combination with the Analysis of Fragmentation Patterns
Source: Metabolites. 2023 Oct 19;13(10):1093. doi: 10.3390/metabo13101093 (PMC10608723; doi:10.3390/metabo13101093)

Structural Identification of Zotarolimus (ABT-578) Metabolites Generated by Human Liver Microsomes Using Ion-Trap and High-Resolution Time-of-Flight Mass Spectrometry in Combination with the Analysis of Fragmentation Patterns

Touraj Shokati, PhD,* Seth H. Drake, BS,* Jost Klawitter, PhD,* Jelena Klawitter, PhD,* and Uwe Christians, MD, PhD*

iC42 Clinical Research and Development, Department of Anesthesiology, School of Medicine, University of Colorado Anschutz Medical Campus, Aurora, CO, USA

Supplemental Digital Contents

Identified Metabolites of Zotarolimus after Incubation with Human Liver Microsomes (HLM)

Desmethyl Metabolites, m/z = 974.54609

1) 16-O-Desmethyl Zotarolimus
2) 27-O-Desmethyl Zotarolimus
3) 39-O-Desmethyl Zotarolimus

Didesmethyl Metabolites, m/z = 960.53044

4) 16, 39-O-Didesmethyl Zotarolimus
5) 27, 39-O-Didesmethyl Zotarolimus
6) 16, 27-O-Didesmethyl Zotarolimus

Hydroxy Metabolites, m/z = 1004.55666

7) 45/46-Hydroxy Zotarolimus
8) 24-Hydroxy Zotarolimus
9) 11-Hydroxy Zotarolimus
10) 12-HydroxyZotarolimus
11) 25-Hydroxy Zotarolimus
12) 3/4/5/6,Hydroxy-piperidine Zotarolimus
13) 23-Hydroxy Zotarolimus
14) 14-Hydroxy Zotarolimus
15) 49-Hydroxy Zotarolimus

Hydroxy, desmethyl Metabolites, m/z = 990.54101

16) 23/24-hydroxy, 16-O-desmethyl Zotarolimus
17) 23/24-hydroxy, 39-O-desmethyl Zotarolimus
18) 25-hydroxy, 39-O-desmethyl Zotarolimus
19) 11-hydroxy, 39-O-desmethyl Zotarolimus
20) Hydroxy-piperidine, 39-O-desmethyl Zotarolimus
21) 45/46-hydroxy, 27-O-desmethyl Zotarolimus

Dihydroxy Metabolites, m/z = 1020.55157

22) 11,24-Dihydroxy Zotarolimus
23) 12,24-Dihydroxy Zotarolimus
24) 11,47/48-Dihydroxy Zotarolimus

Structure and MS Fragmentation of Zotarolimus

Structural Formula of Zotarolimus

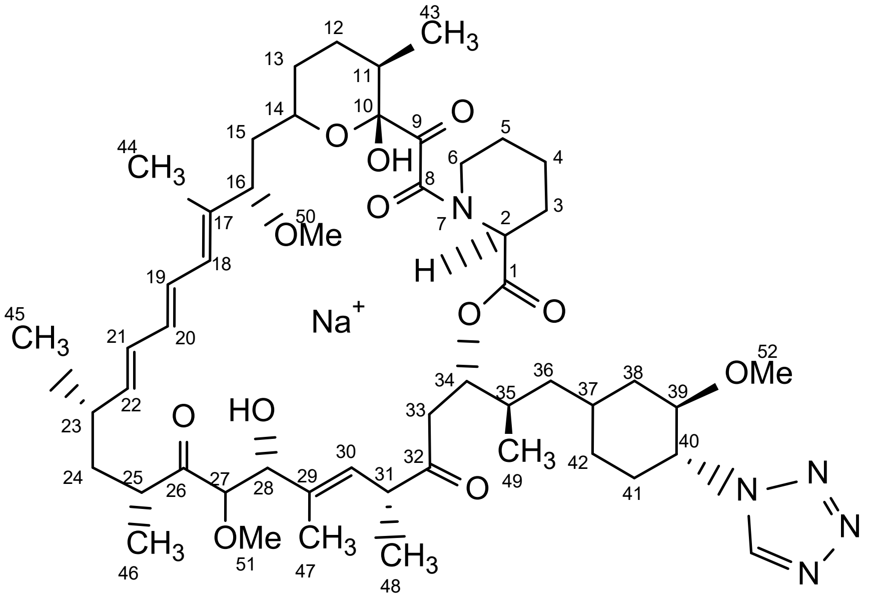

[Zotarolimus-Na]$^+$

Chemical Formula: $C_{52}H_{79}N_5NaO_{12}^+$

Exact Mass: 988.56174

Structural Formula of Zotarolimus Isomers

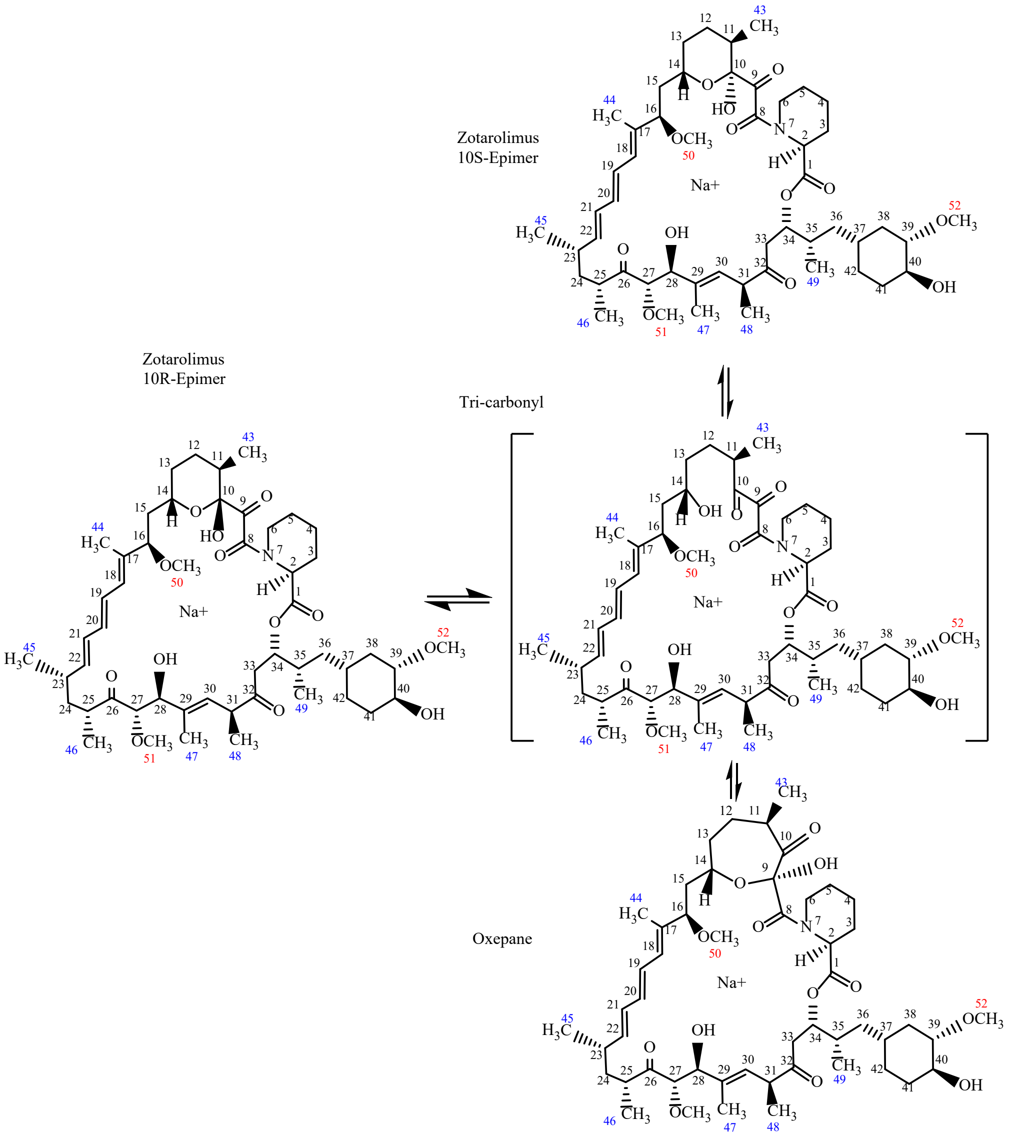

Zotarolimus 10S-Epimer

Zotarolimus 10R-Epimer

Tri-carbonyl

Oxepane

Total Ion Chromatogram of Zotarolimus Measured by TOF

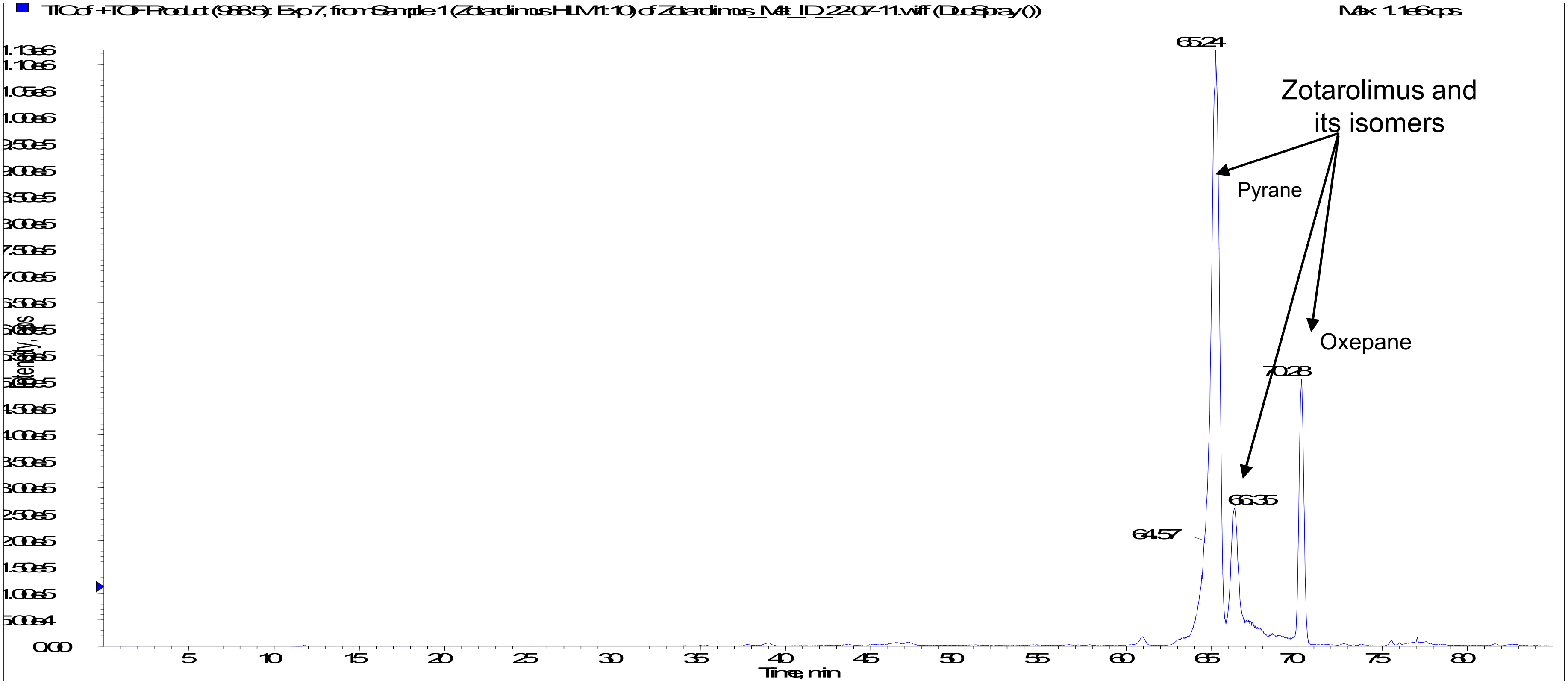

Exact Masses of Zotarolimus Adducts and Their Isotopic Pattern Measured by TOF

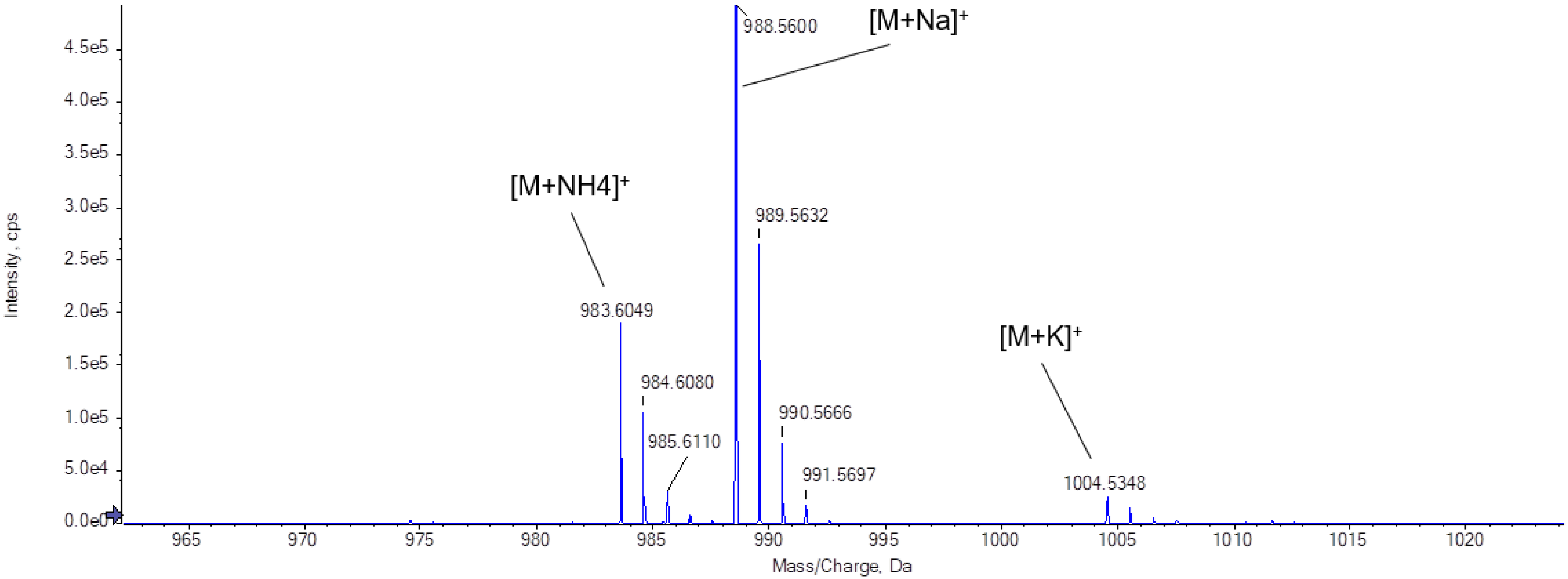

Zotarolimus	theoretical mass	measured mass	Δppm
**[M+Na]+**	988.56174	988.56000	1.8
**[M+NH4]+**	983.60635	983.60490	1.5
**[M+K]+**	1004.53568	1004.53480	0.9

MS Spectrum of Zotarolimus, CE = 75 eV, DP = 135 V

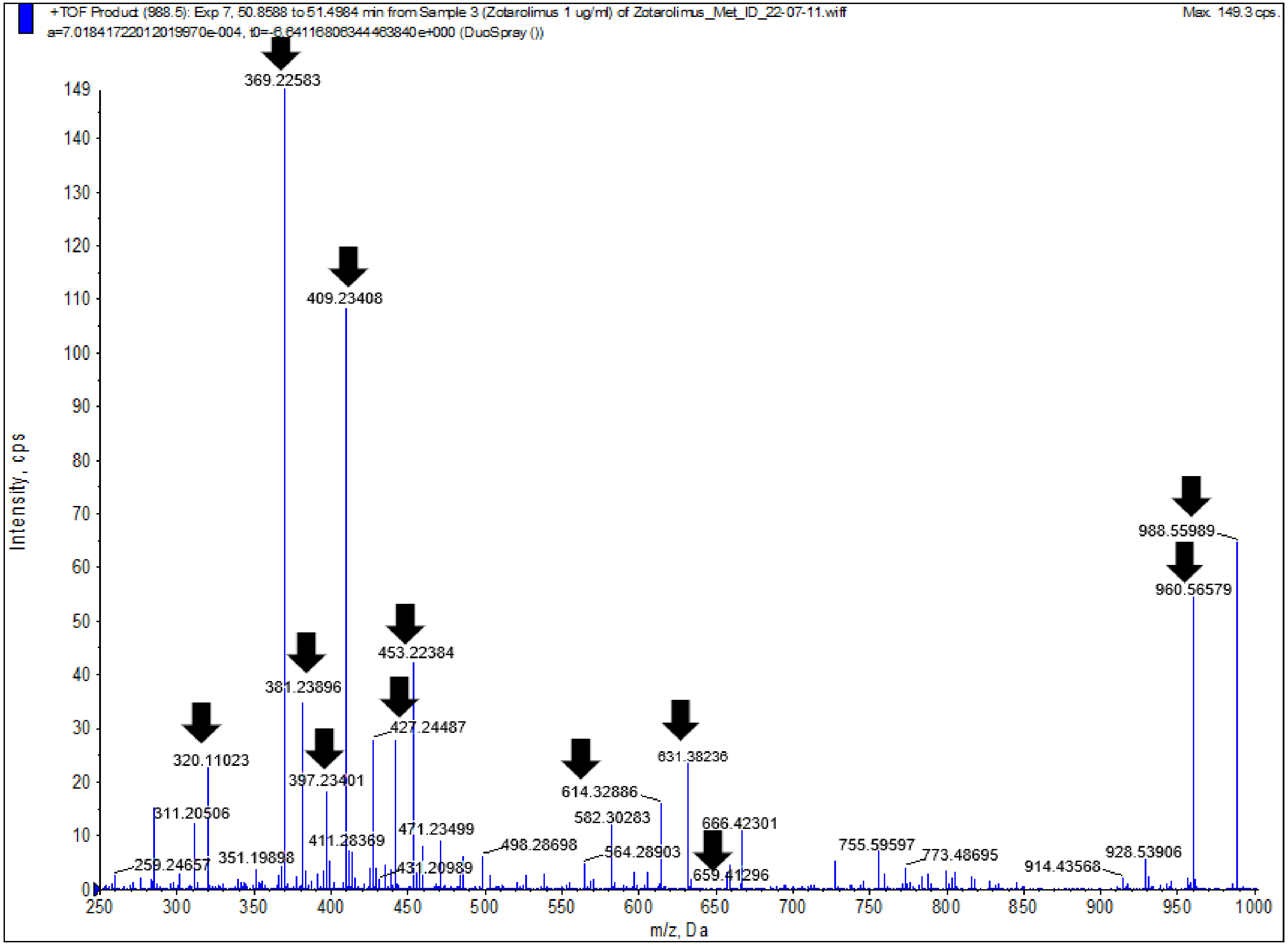

Zotarolimus Fragmentation Pathway

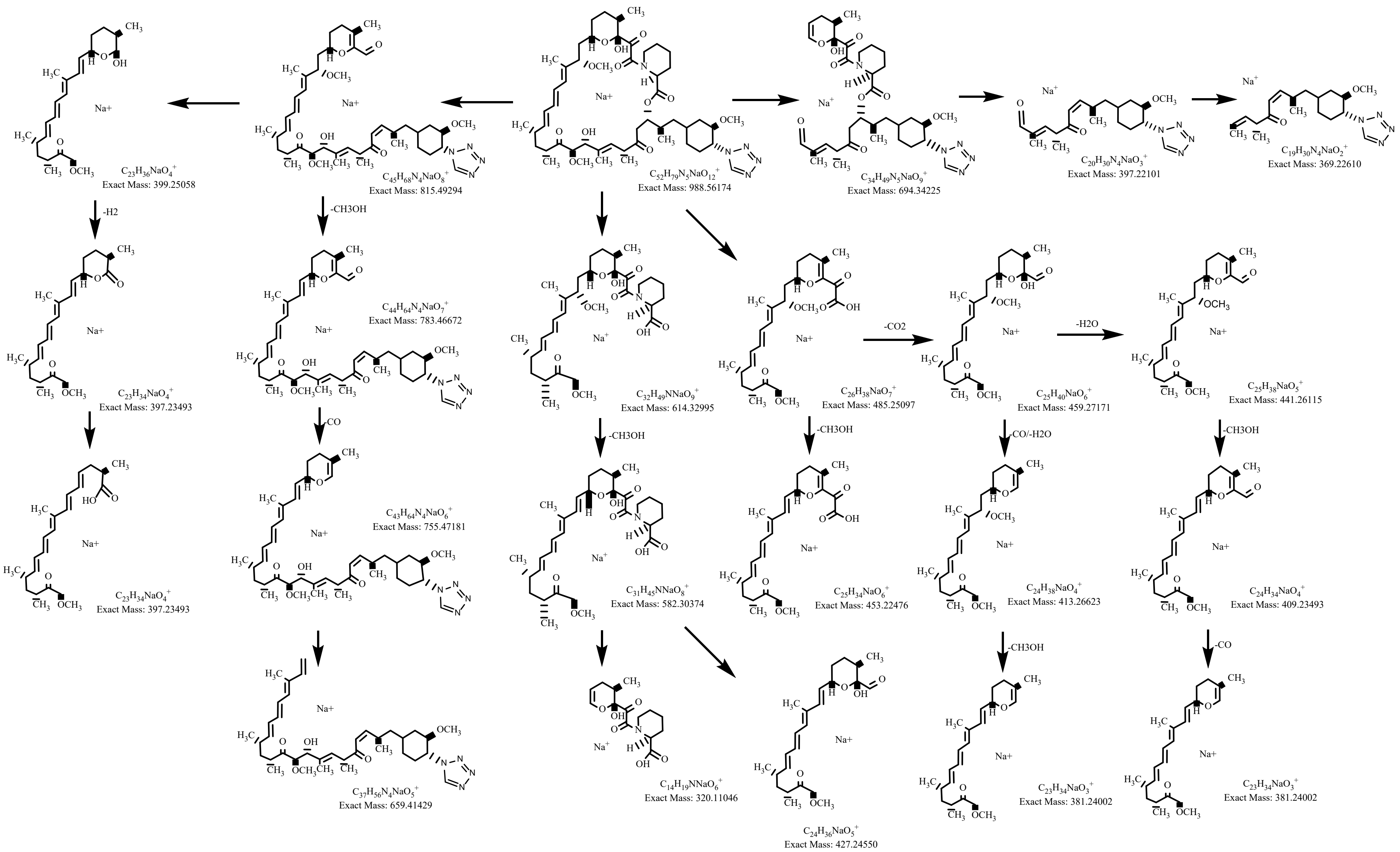

Zotarolimus Fragmentation Pathway Continued

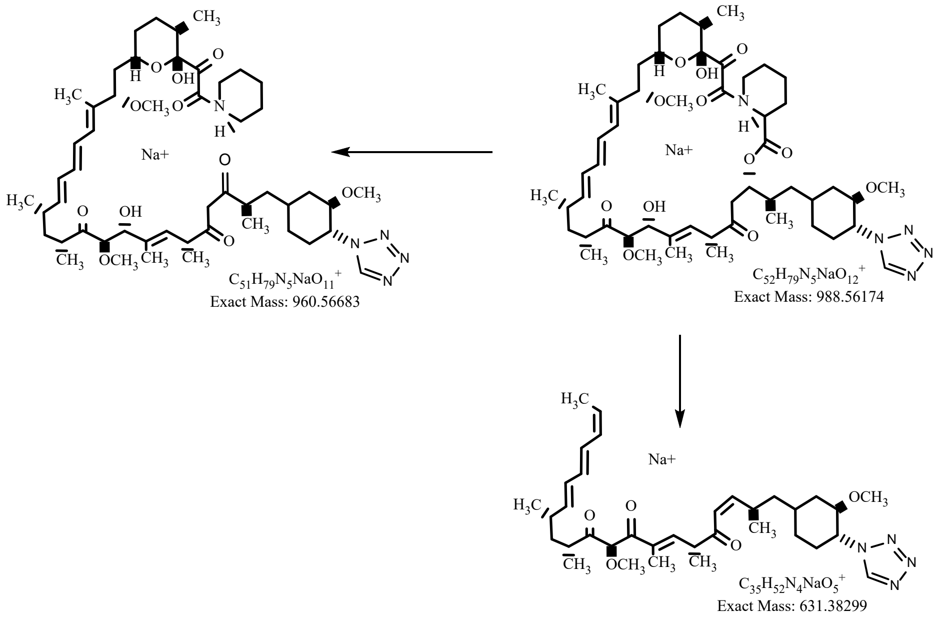

Δppm of Zotarolimus Fragments Measured by TOF

Fragment No.	theoretical mass	measured mass	Δppm
1	320.11046	320.11023	0.7
2	369.22610	369.22583	0.7
3	381.24002	381.23896	2.8
4	397.23493	397.23401	2.3
5	409.23493	409.23408	2.1
6	427.24550	427.24487	1.5
7	441.26115	441.26068	1.1
8	453.22476	453.22384	2.0
9	582.30374	582.30283	1.6
10	614.32995	614.32886	1.8
11	631.38299	631.38236	1.0
12	659.41429	659.41296	2.0
13	960.56683	960.56579	1.1
14	988.56174	988.55989	1.9

Hydroxy Zotarolimus Metabolites, m/z = 1004.55666

Hydroxy Zotarolimus Metabolites Structures, m/z = 1004.55666

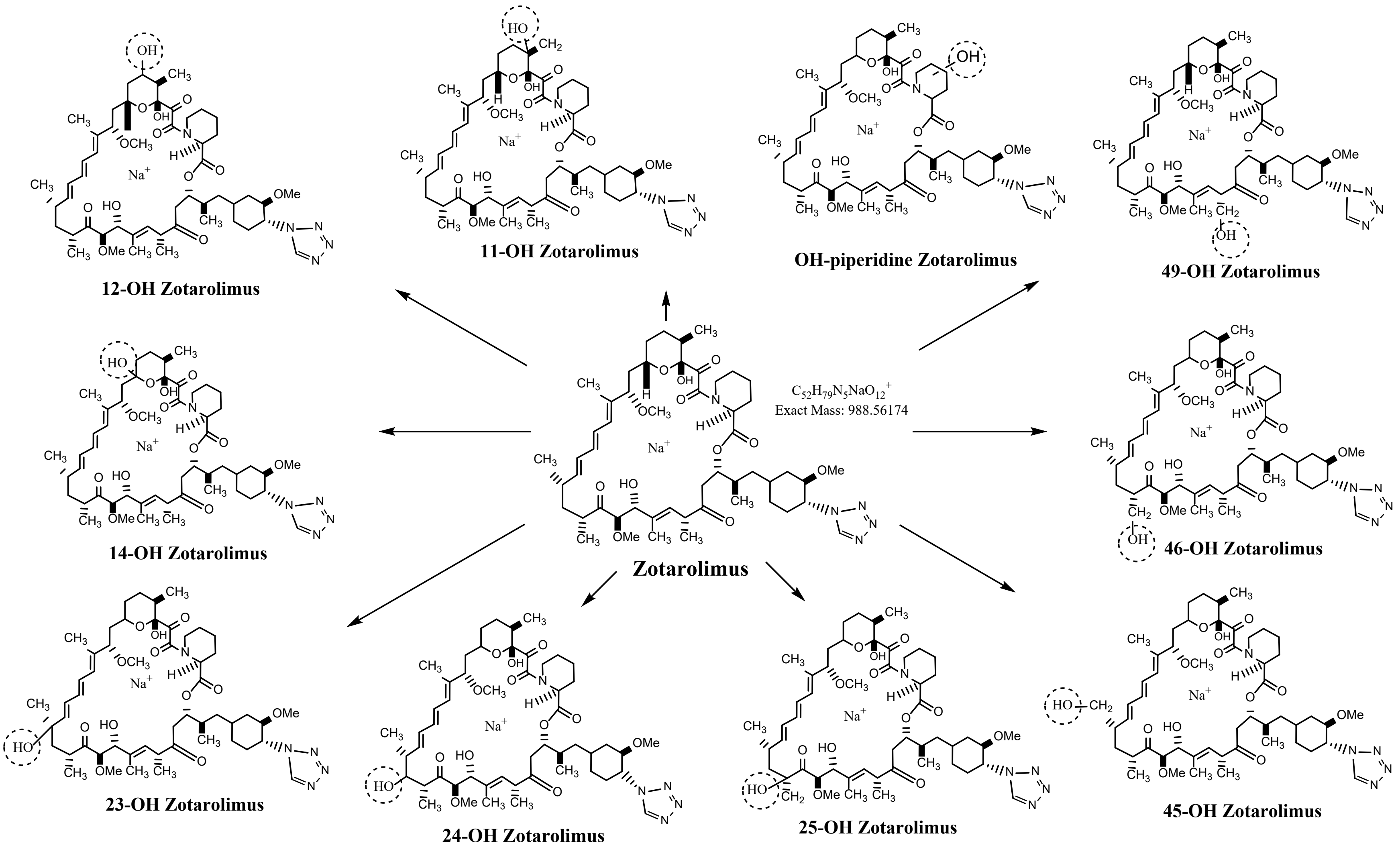

45- and 46-Hydroxy Zotarolimus, m/z = 1004.55666

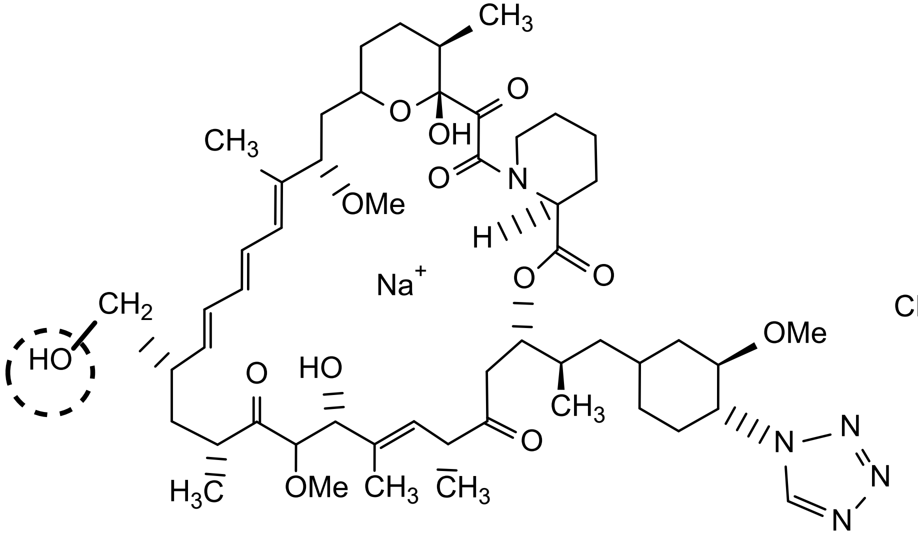

45-hydroxy zotarolimus

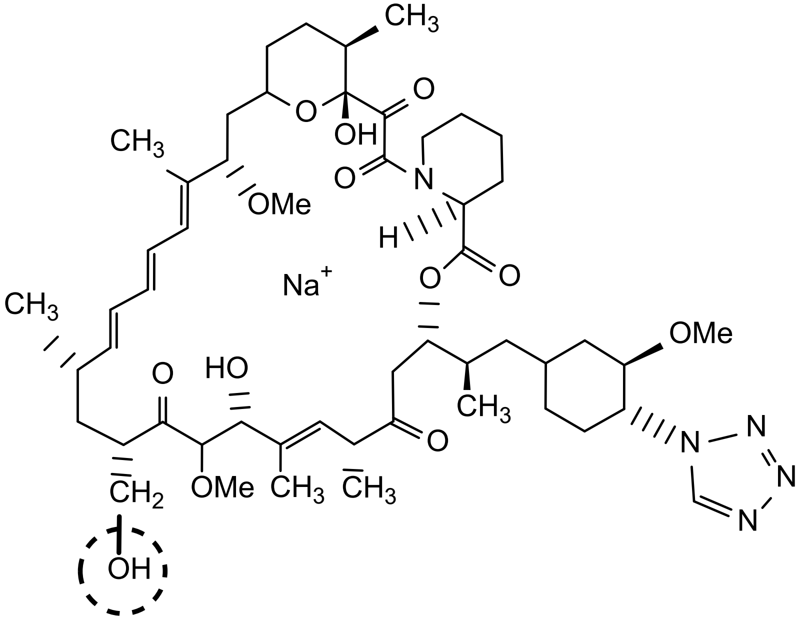

46-hydroxy zotarolimus

Chemical Formula: $C_{52}H_{79}N_5NaO_{13}{}^{+}$

Exact Mass: 1004.55666

45- and 46-Hydroxy Zotarolimus Metabolites
Extracted Ion Chromatogram (EIC), m/z = 1004.55666

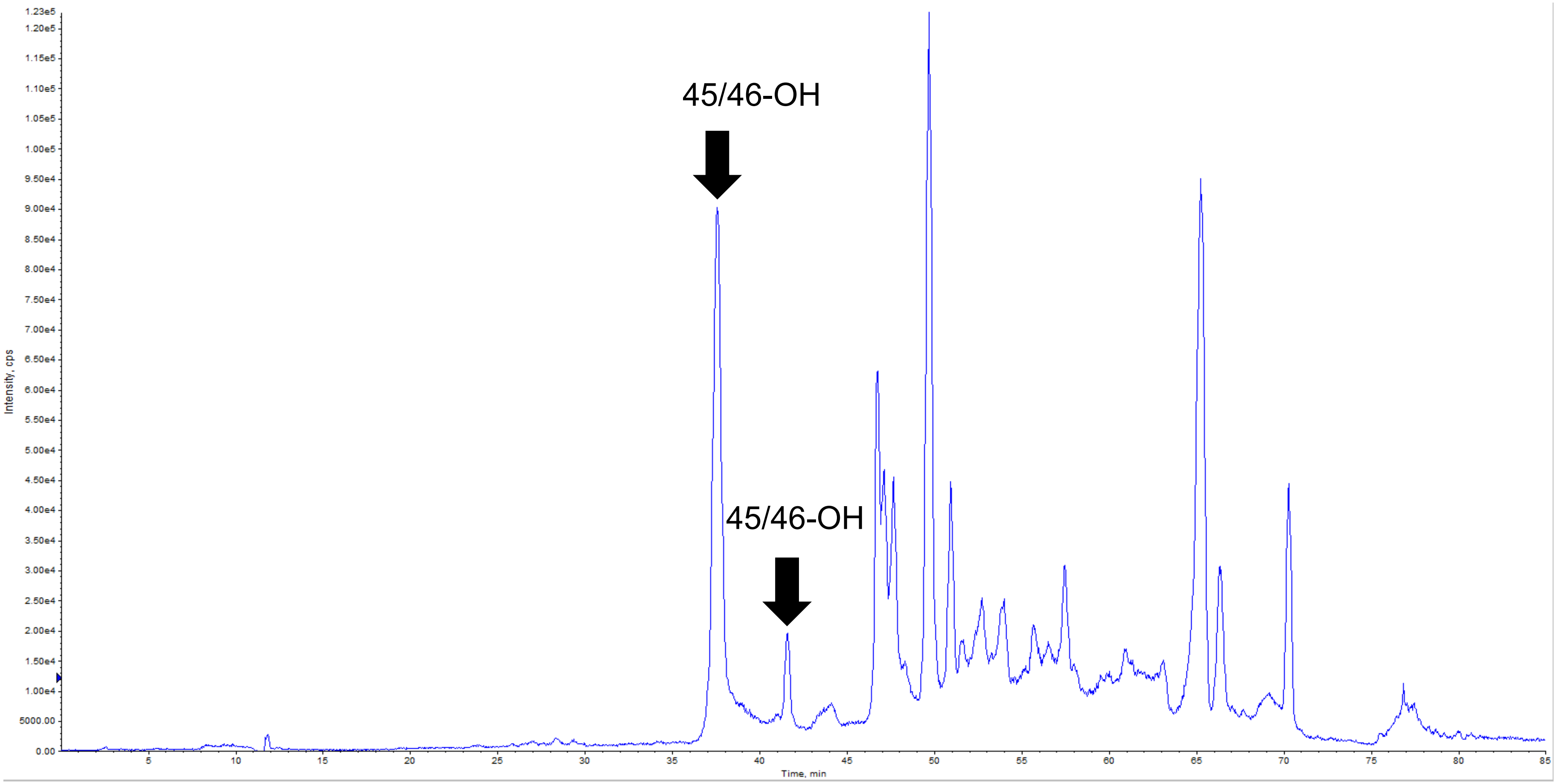

MS spectrum of 45- and 46-Hydroxy Zotarolimus
CE = 75 eV, DP = 125 V

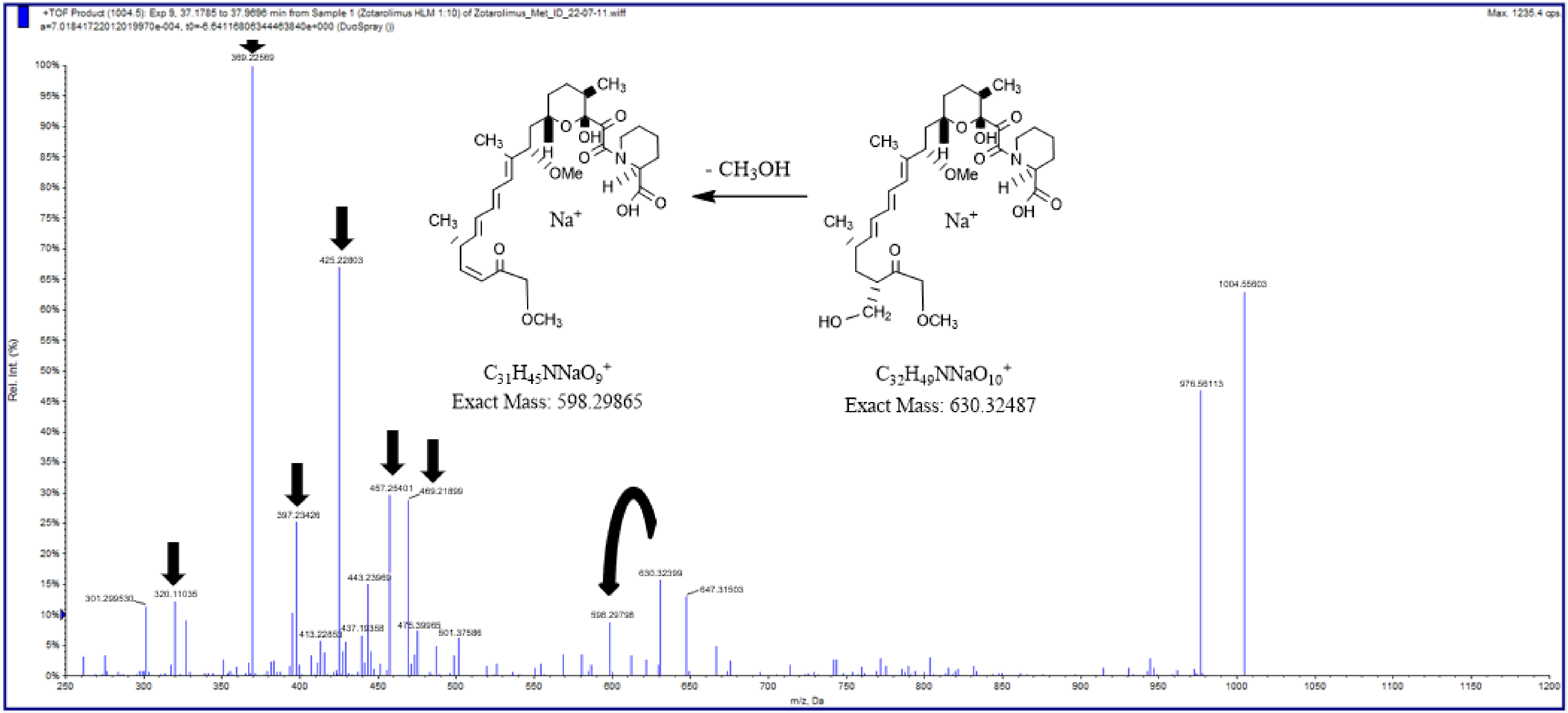

Δppm of 45- and 46-OH Zotarolimus Fragments

Fragment No.	Theoretical mass	Measured mass	Δppm
1	320.11046	320.11035	0.3
2	369.2261	369.22569	1.1
3	397.23493	397.23426	1.7
4	413.22985	413.22853	3.2
5	425.22985	425.22803	4.3
6	443.24041	443.23969	2.4
7	457.25606	457.25501	2.3
8	469.21967	469.21899	1.4
9	598.29865	598.29798	1.1
10	630.32487	630.32399	1.4
11	659.41429	659.41379 (low intensity)	0.8
12	675.40921	675.40857 (low intensity)	0.9
13	771.46672	771.46567 (low intensity)	1.4
14	831.48785	831.48693 (low intensity)	1.1
15	1004.55666	1004.55603	0.6

45-Hydroxy Zotarolimus Fragmentation Pattern

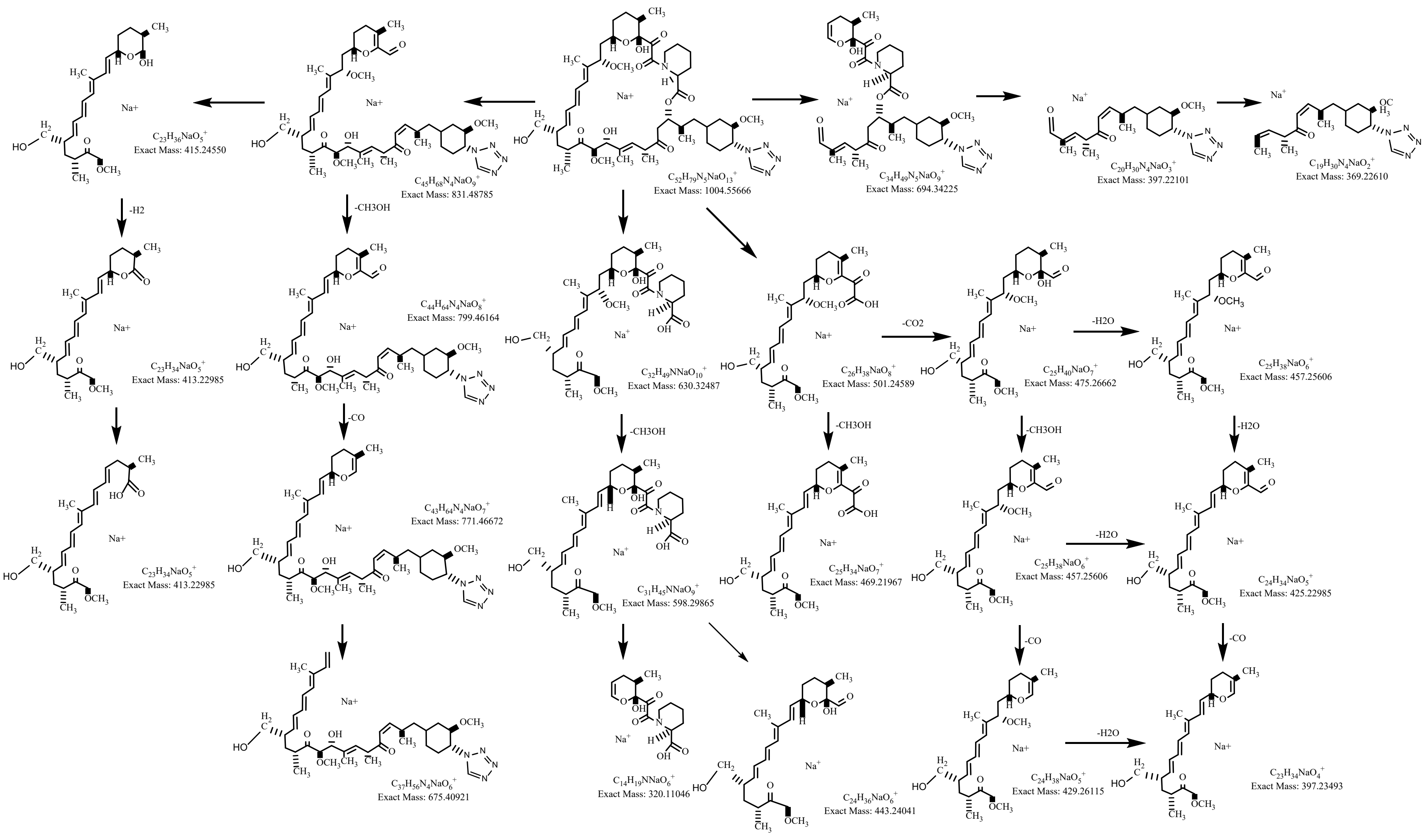

46-Hydroxy Zotarolimus Fragmentation Pattern

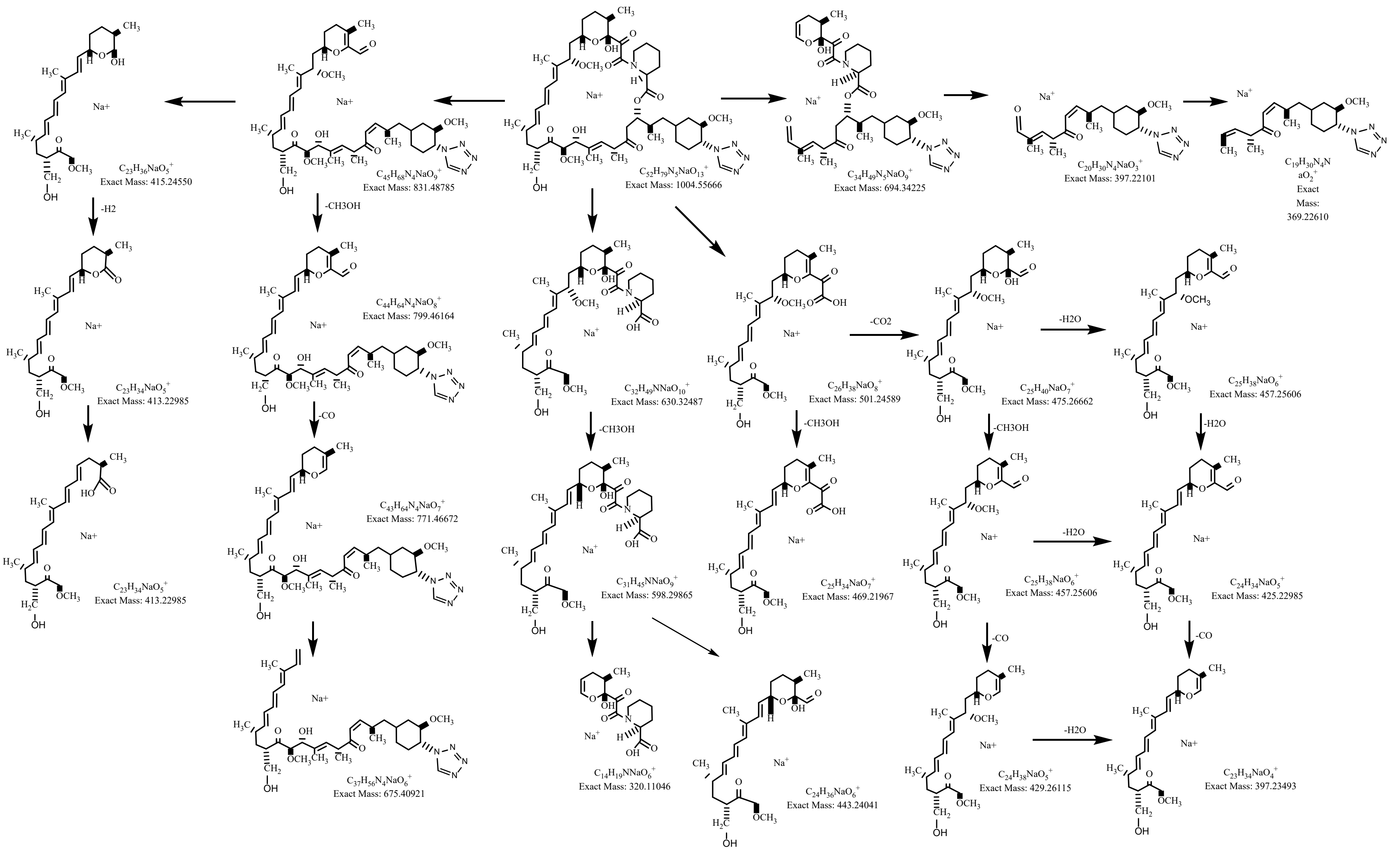

45/46-Hydroxy Zotarolimus Fragmentation Pattern (Methanol Loss)

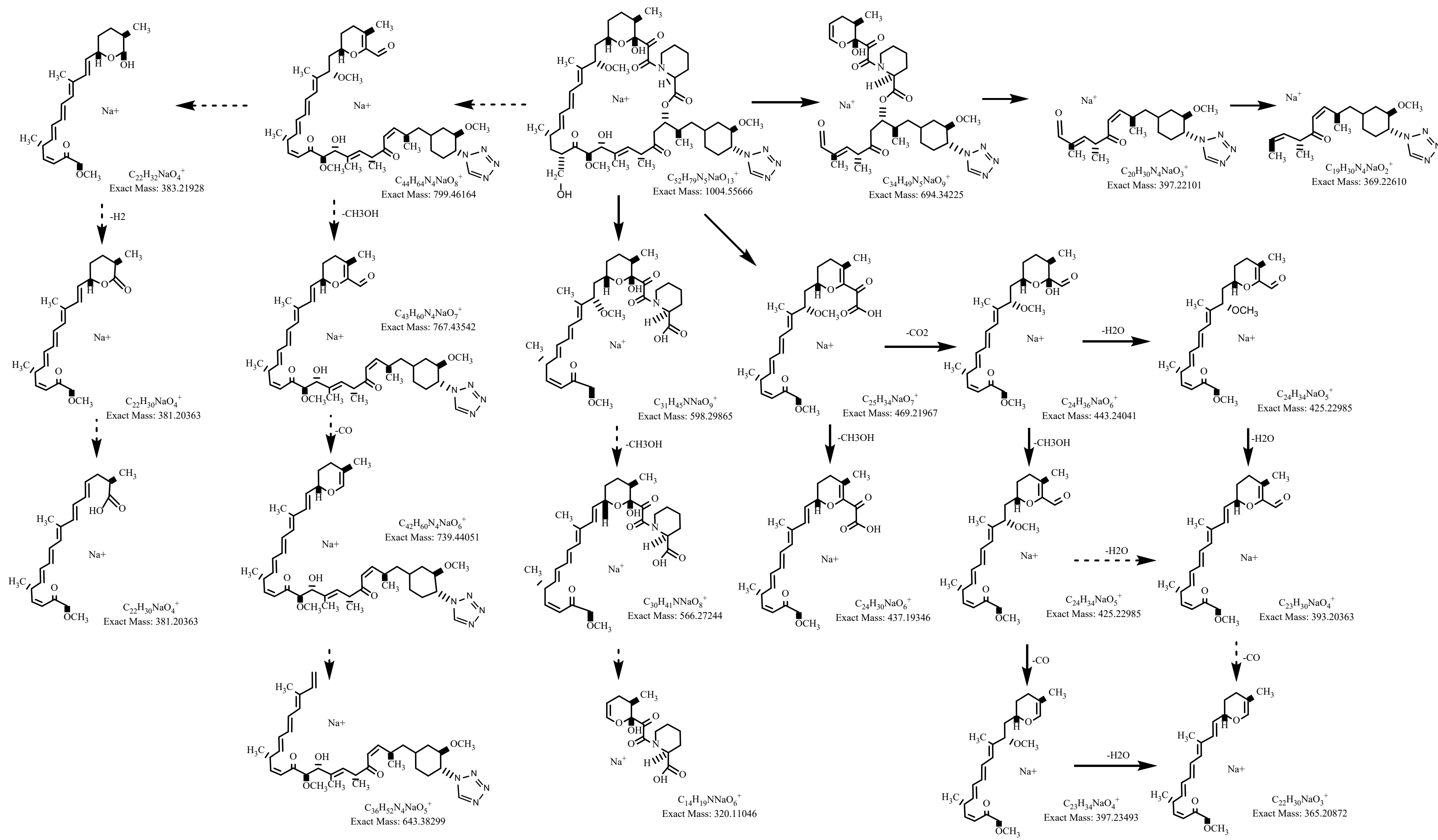

Structural Assessment of 45- and 46-Hydroxy Zotarolimus by Comparison of Fragmentation Patterns with those of Zotarolimus

Zotarolimus Fragments	45/46-Hydroxy Zotarolimus Fragments	Comments	
320.11046	320.11046	no hydroxylation	Rules out OH-piperidine, 11-, 12-, and 14-OH
369.22610	369.22610	no hydroxylation	Rules out 47-, 48-, or 49-OH
381.24002	397.24002	(+16) Hydroxylation	11-, 12-, 14-, 23-, 24-, 25-, 45-, or 46-OH present
397.23493	413.23493	(+16) Hydroxylation	11-, 12-, 14-, 23-, 24-, 25-, 45-, or 46-OH present
409.23493	425.23493	(+16) Hydroxylation	11-, 12-, 14-, 23-, 24-, 25-, 45-, or 46-OH present
427.24550	443.24550	(+16) Hydroxylation	11-, 12-, 14-, 23-, 24-, 25-, 45-, or 46-OH present
441.26115	457.26115	(+16) Hydroxylation	11-, 12-, 14-, 23-, 24-, 25-, 45-, or 46-OH present
453.22476	469.22476	(+16) Hydroxylation	11-, 12-, 14-, 23-, 24-, 25-, 45-, or 46-OH present
582.30374	598.30374	(+16) Hydroxylation	11-, 12-, 14-, 23-, 24-, 25-, 45-, 46-, or piperidine-OH present
614.32995	630.32995	(+16) Hydroxylation	11-, 12-, 14-, 23-, 24-, 25-, 45-, 46-, or piperidine-OH present
659.41429	675.41429	(+16) Hydroxylation	Rules out OH-piperidine, 11-, 12-, and 14-OH
694.34225	694.34225	no hydroxylation	Rules out OH-piperidine/ 11-, 12-, 14-, 47-, 48-, and 49-OH
783.46672	799.46672	(+16) Hydroxylation	11-, 12-, 14-, 23-, 24-, 25-, 45-, 46-, 47-, 48-, or 49-OH present
815.49294	831.49294	(+16) Hydroxylation	11-, 12-, 14-, 23-, 24-, 25-, 45-, 46-, 47-, 48-, or 49-OH present
988.56174	1004.56174	(+16) Hydroxylation	
Characteristic Fragments			630 – 32 = 598<br>457 – 32 = 425<br>501 – 32 = 469
Determinant patterns	methanol loss		45/46-OH favor the loss of Methanol in hydroxylated fragments

12-Hydroxy Zotarolimus, m/z = 1004.55666

12-Hydroxy Zotarolimus, m/z = 1004.55666

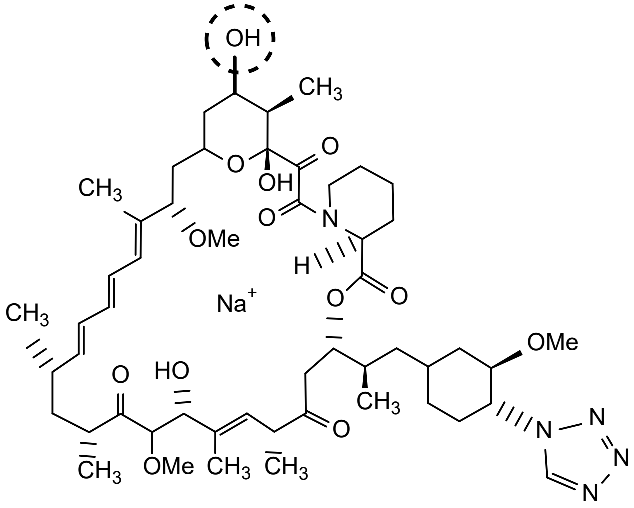

Chemical Formula: $C_{52}H_{79}N_5NaO_{13}^+$
Exact Mass: 1004.55666

12-hydroxy zotarolimus

12-Hydroxy Zotarolimus, m/z = 1004.55666
Extracted Ion Chromatogram (EIC)

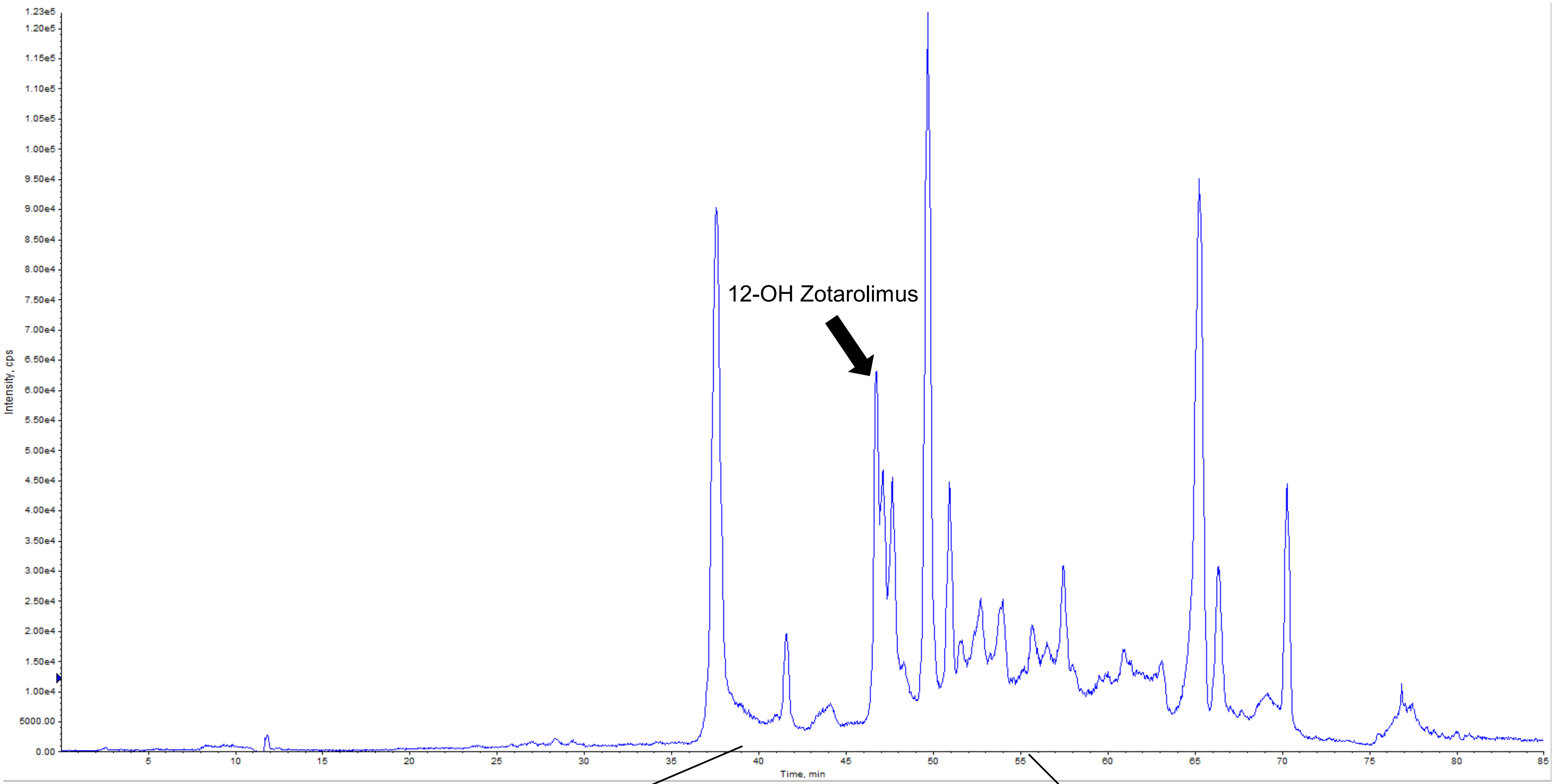

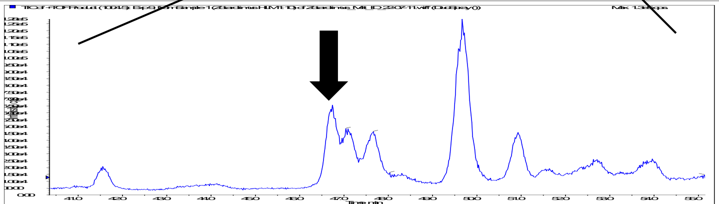

Mass Spectrum of 12-Hydroxy Zotarolimus
CE = 75 eV, DP = 125 V

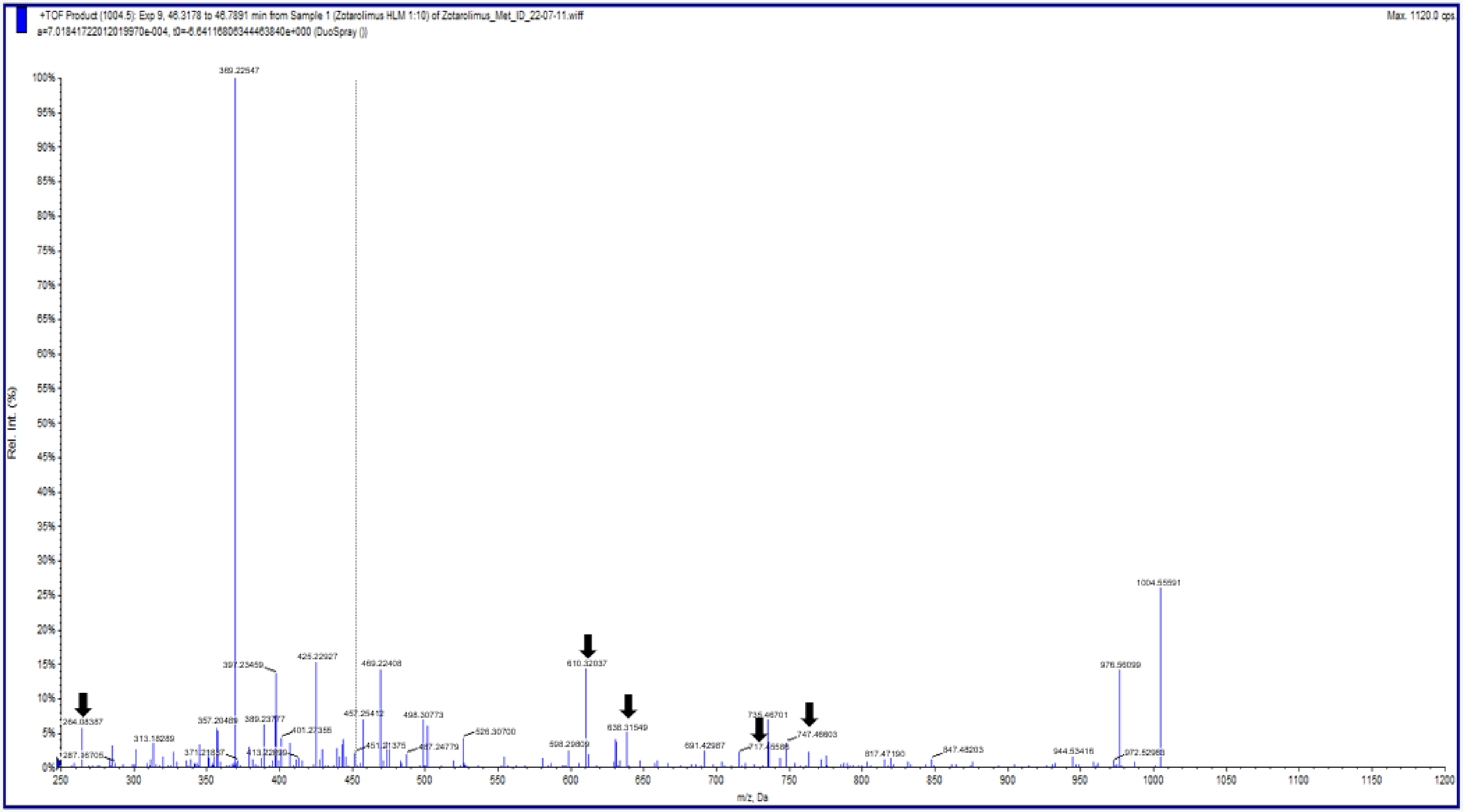

Δppm of 12-Hydroxy Zotarolimus Fragments

Fragment No.	Theoretical mass	Measured mass	Δppm
1	264.08424	264.08387	1.4
2	369.22610	369.22547	1.7
3	397.23493	397.23459	0.9
4	413.22985	413.22899	2.1
5	425.22985	425.22927	1.4
6	443.24041	443.23992	1.1
7	469.22476	469.22408	1.4
8	598.29865	598.29809	0.9
9	675.40921	675.40887	0.5
10	817.4722	817.4719	0.4
11	847.48277	847.48203	0.9
12	1004.55666	1004.55591	0.7

Characteristic Fragments

theoretical mass	measured mass	Δppm
747.46672	747.46603	0.9
717.45616	717.45586	0.4
638.31603	638.31549	0.8
610.32112	610.32037	1.2
264.08424	264.08387	1.4

12-Hydroxy Zotarolimus, m/z = 1004.55666

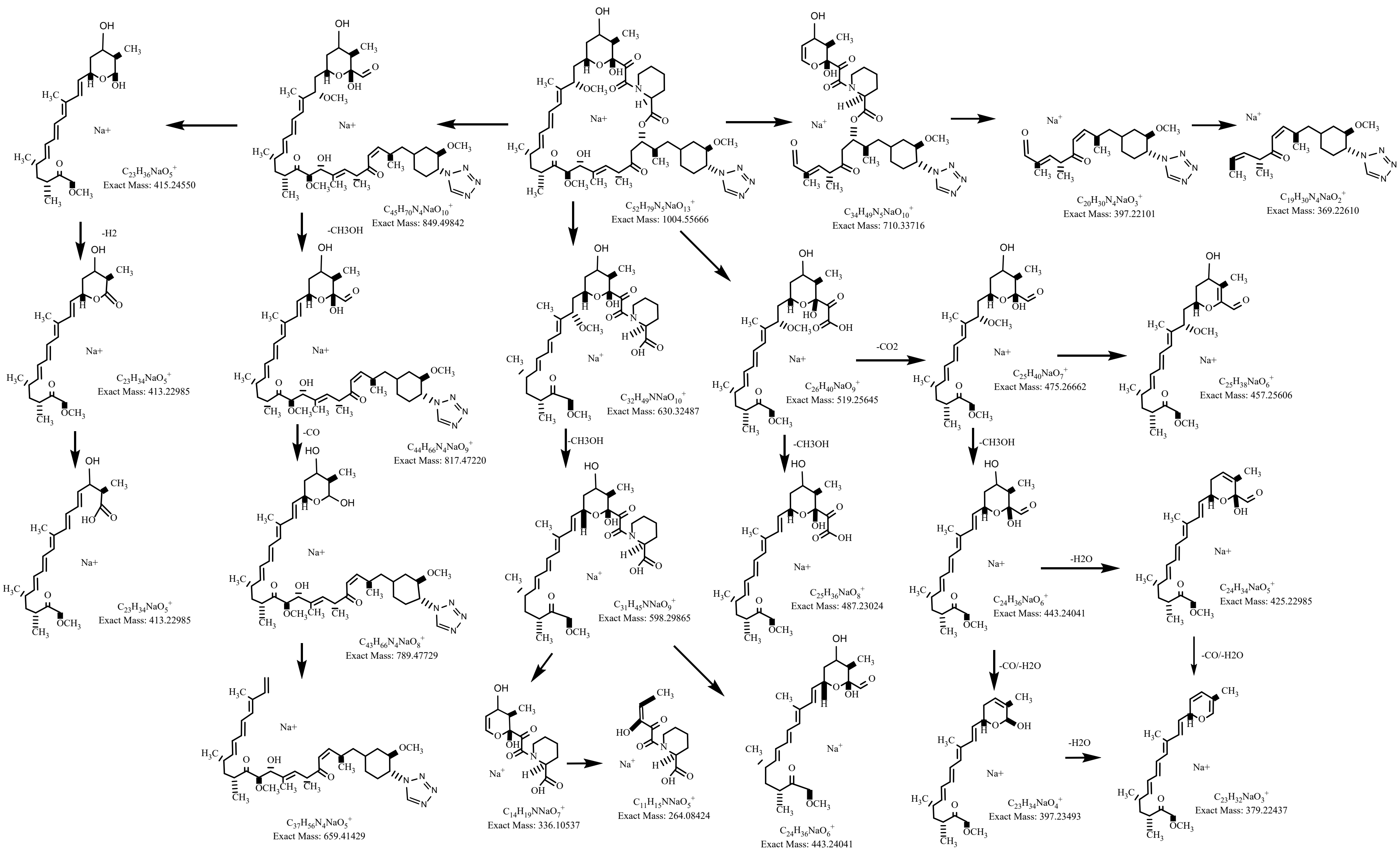

12-OH Zotarolimus, Characteristic Fragments

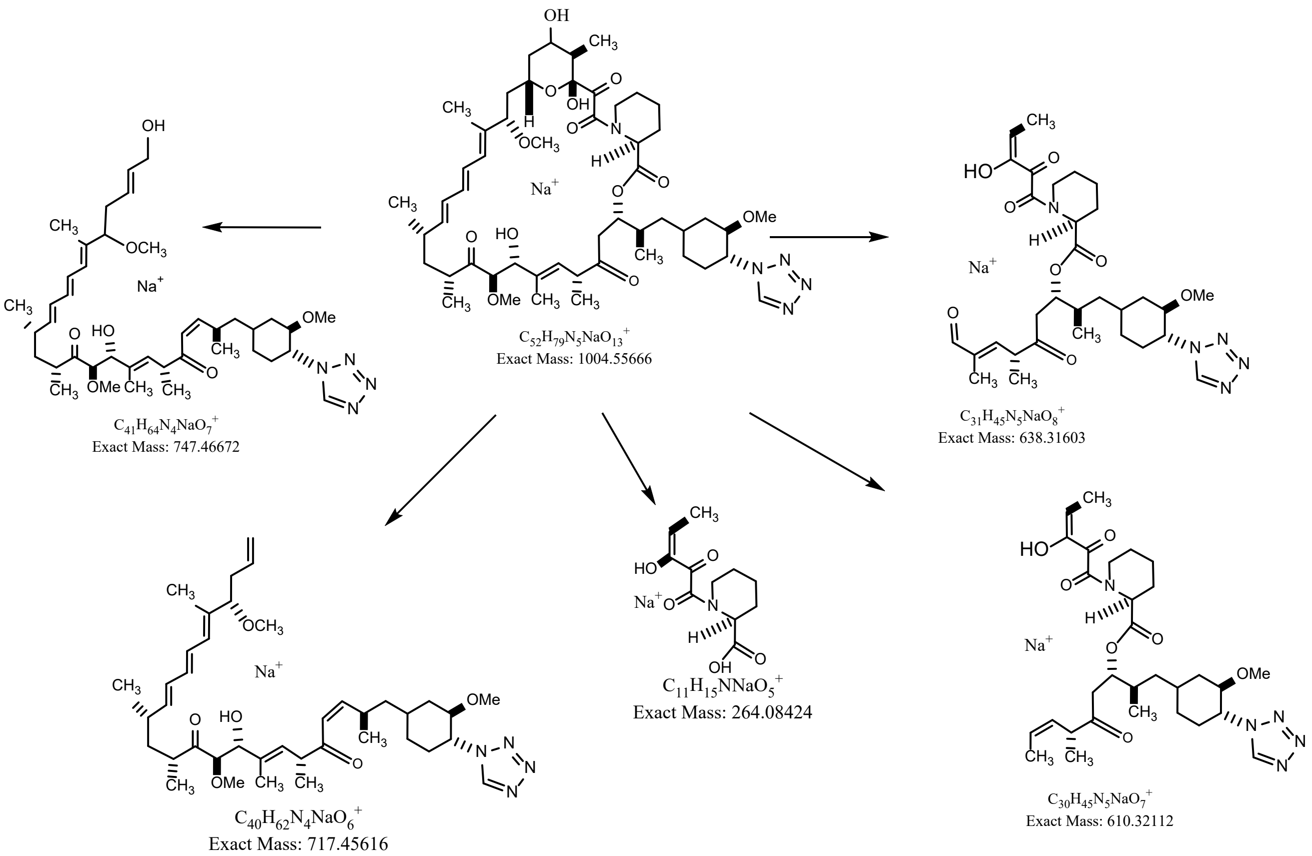

Structural Assessment of 12-Hydroxy Zotarolimus by Comparison of Fragmentation Patterns with those of Zotarolimus

Zotarolimus Fragments	12-Hydroxy Zotarolimus Fragments	Comments	
320.11046	264.08387	Hydroxylation with rearrangement	11, 12, and Piperidine-OH present
369.22610	369.22547	no hydroxylation	Rules out 47, 48, and 49-OH
381.24002	397.23459	(+16) Hydroxylation	11, 12, 14, 23, 24, 25, 45, or 46-OH present
397.23493	413.22899	(+16) Hydroxylation	11, 12, 14, 23, 24, 25, 45, or 46-OH present
409.23493	425.22927	(+16) Hydroxylation	11, 12, 14, 23, 24, 25, 45, or 46-OH present
427.24550	443.23992	(+16) Hydroxylation	11, 12, 14, 23, 24, 25, 45, or 46-OH present
453.22476	469.22408	(+16) Hydroxylation	11, 12, 14, 23, 24, 25, 45, or 46-OH present
582.30374	598.29809	(+16) Hydroxylation	11, 12, 14, 23, 24, 25, 45, 46-OH, or piperidine-OH present
614.32995	630.32403	(+16) Hydroxylation	11, 12, 14, 23, 24, 25, 45, 46-OH, or piperidine-OH present
659.41429	659.41400	no hydroxylation	Rules out 23, 24, 45, 46, 47, 48, and 49-OH
783.46672	799.46089	(+16) Hydroxylation	Rules out OH-piperidine
831.48785	847.48203	(+16) Hydroxylation	Rules out OH-piperidine
988.56174	1004.55591	(+16) Hydroxylation	
Characteristic Fragments			264, 610, 638, 717, and 747
Determinant patterns			

24-Hydroxy Zotarolimus, m/z = 1004.55666

24-Hydroxy Zotarolimus, m/z = 1004.55666

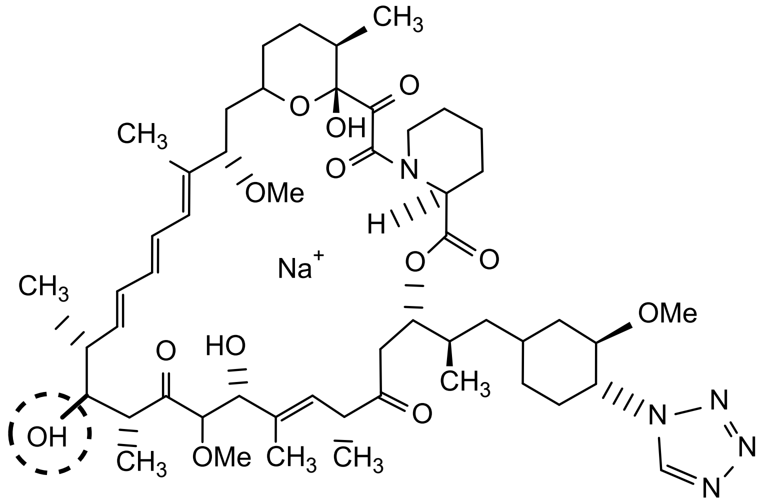

Chemical Formula: $C_{52}H_{79}N_5NaO_{13}^{+}$
Exact Mass: 1004.55666

24-hydroxy zotarolimus

24-Hydroxy Zotarolimus, m/z = 1004.55666
Extracted Ion Chromatogram (EIC)

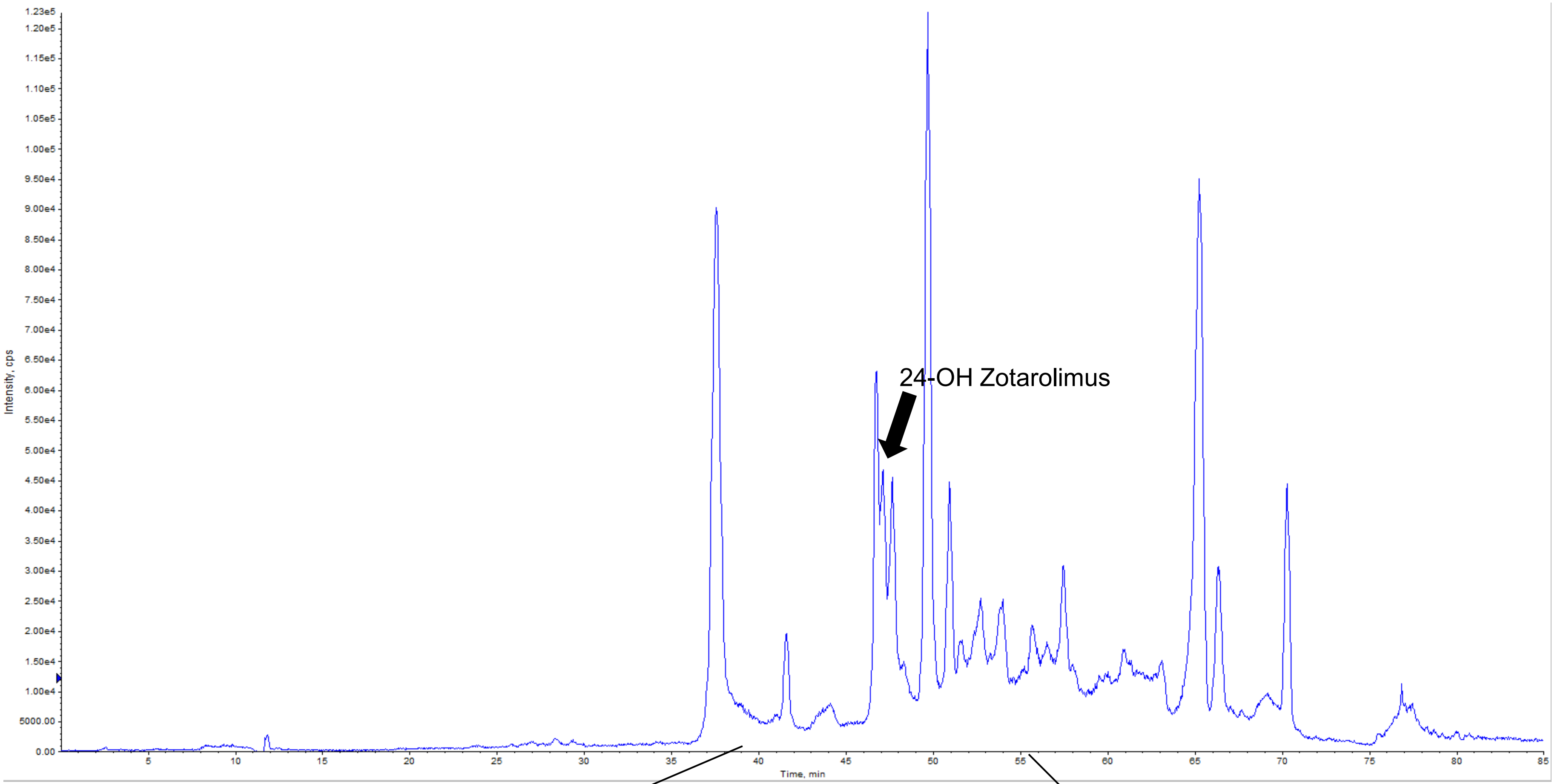

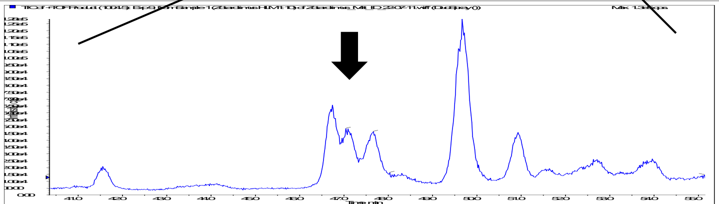

Mass Spectrum of 24-Hydroxy Zotarolimus
CE = 75 eV, DP = 125 V

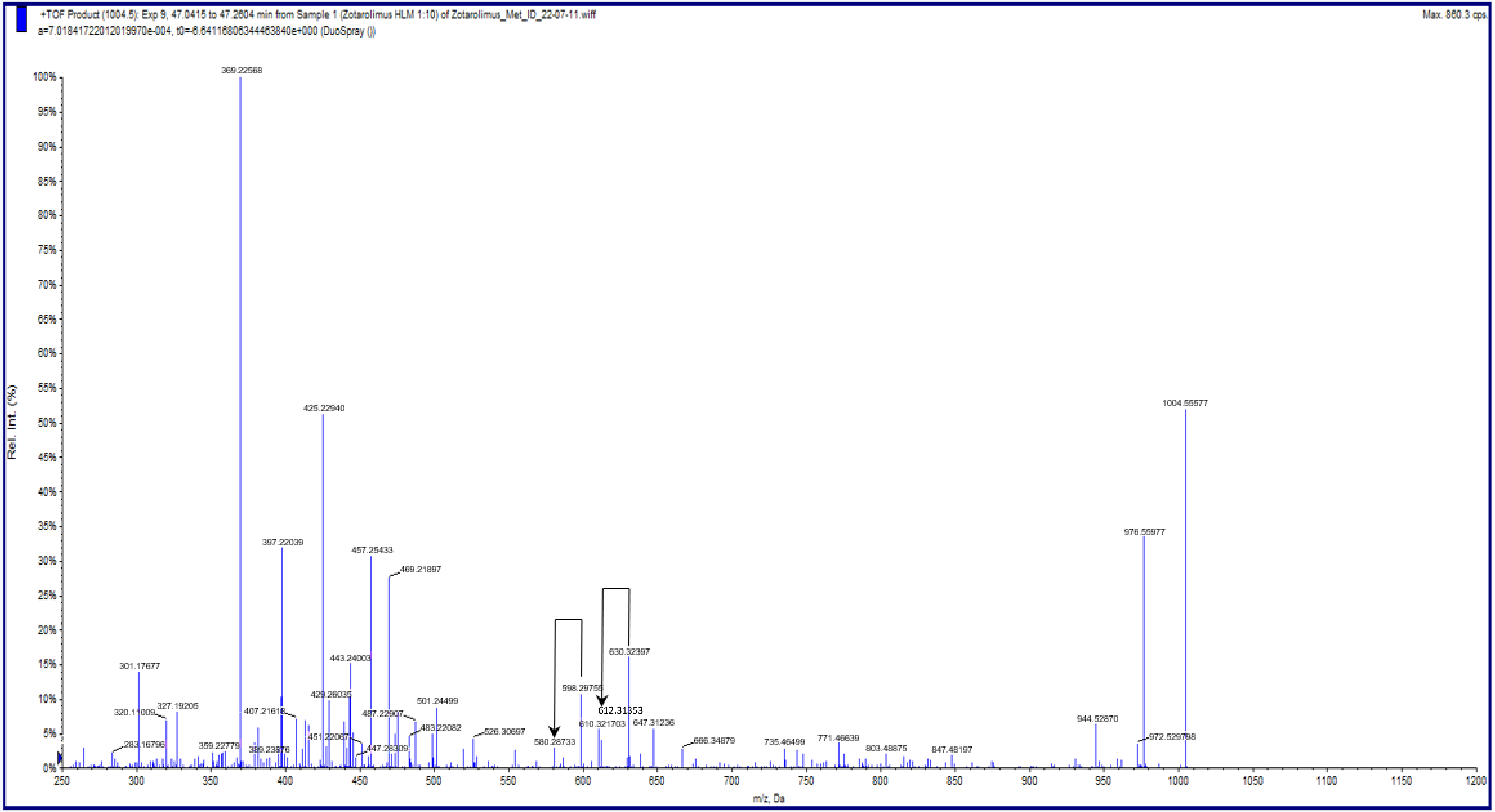

Δppm of 24-Hydroxy Zotarolimus Fragments

Fragment No.	Theoretical mass	Measured mass	Δppm
1	320.11046	320.11009	1.2
2	369.22610	369.22568	1.1
3	397.22101	397.22039	1.6
4	429.26115	429.26035	1.9
5	425.22985	425.22940	1.1
6	443.24041	443.24003	0.9
7	457.25606	457.25563	0.9
8	469.21967	469.21897	1.5
9	501.24589	501.24499	1.8
10	598.29865	598.29755	1.8
11	630.32487	630.32397	1.4
12	647.37791	647.37703	1.4
13	771.46672	771.46639	0.4
14	817.4722	817.4719 (low intensity)	0.4
15	831.48785	831.48709 (low intensity)	0.9
16	1004.55666	1004.55577	0.9

Characteristic Fragments

Theoretical mass	Measured mass	Δppm
612.31430	612.31353	1.3
580.28809	580.28733	1.3
327.19307	327.19205	3.1
301.17742	301.17677	2.2

24-Hydroxy Zotarolimus, m/z = 1004.55666

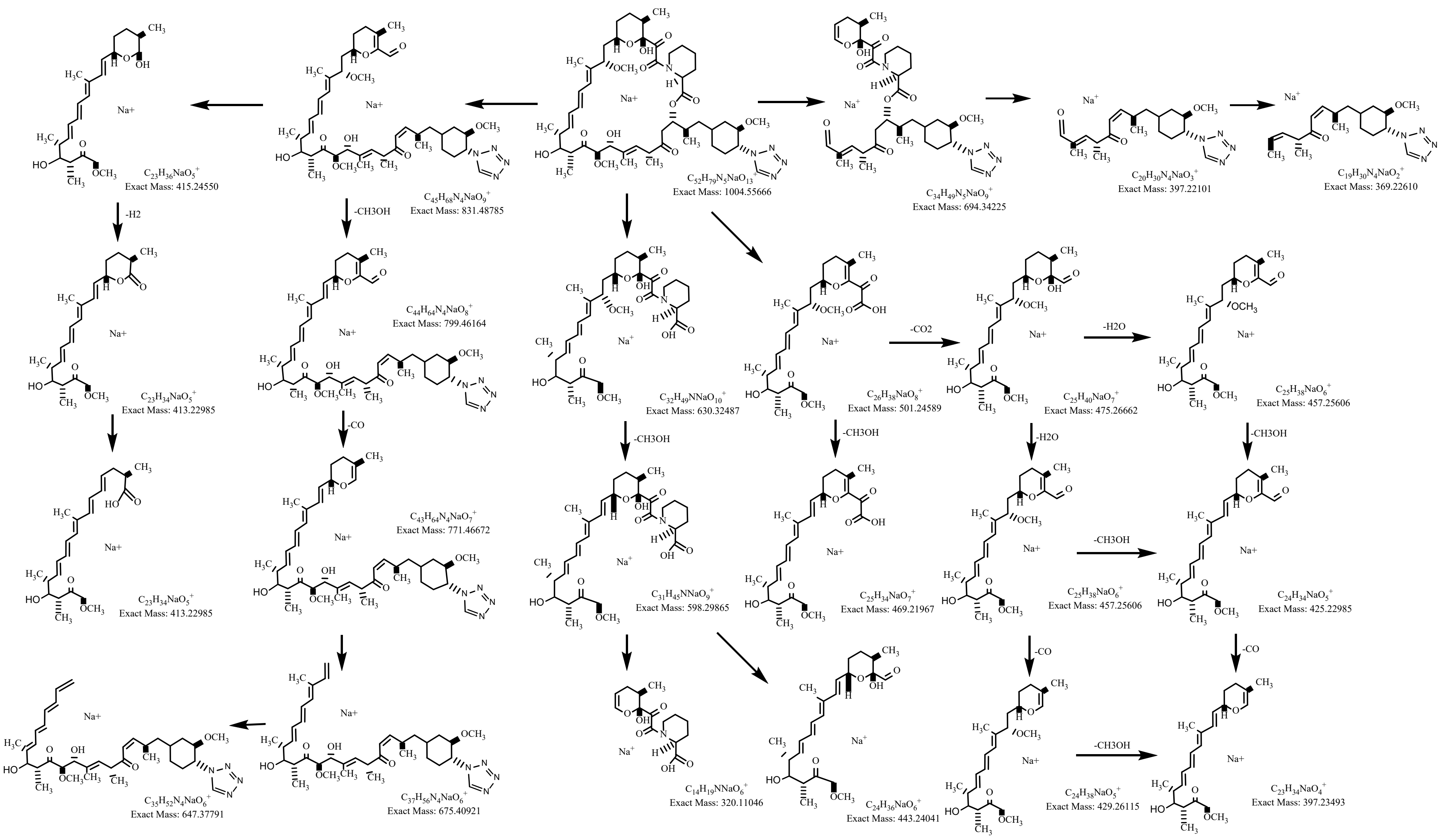

Characteristic Fragments of 24-OH Zotarolimus

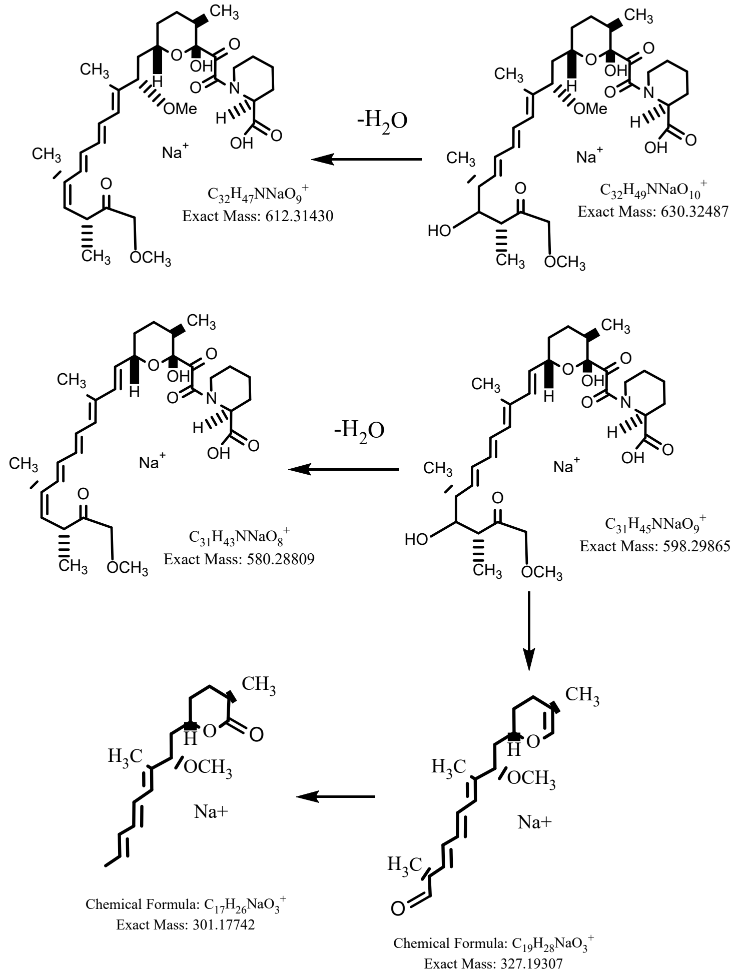

Structural Assessment of 24-Hydroxy Zotarolimus by Comparison of Fragmentation Patterns with those of Zotarolimus

Zotarolimus Fragments	24-Hydroxy Zotarolimus Fragments	Comments	
320.11046	320.11009	no hydroxylation	Rules out 11, 12, and Piperidine-OH
369.22610	369.22568	no hydroxylation	Rules out 47, 48, and -OH
381.24002	397.22039	(+16) Hydroxylation	11, 12, 14, 23, 24, 25, 45, or 46-OH present
397.23493	413.22934	(+16) Hydroxylation	11, 12, 14, 23, 24, 25, 45, or 46-OH present
409.23493	425.22940	(+16) Hydroxylation	11, 12, 14, 23, 24, 25, 45, or 46-OH present
427.24550	443.24003	(+16) Hydroxylation	11, 12, 14, 23, 24, 25, 45, or 46-OH present
413.26623	429.26035	(+16) Hydroxylation	11, 12, 14, 23, 24, 25, 45, or 46-OH present
453.22476	469.21897	(+16) Hydroxylation	11, 12, 14, 23, 24, 25, 45, or 46-OH present
582.30374	598.29755	(+16) Hydroxylation	11, 12, 14, 23, 24, 25, 45, 46-OH, or piperidine-OH present
614.32995	630.32397	(+16) Hydroxylation	11, 12, 14, 23, 24, 25, 45, 46-OH, or piperidine-OH present
659.41429	675.40832	(+16) Hydroxylation	Rules out 11, 12, and Piperidine-OH
755.47181	771.46639	(+16) Hydroxylation	Rules out OH-piperidine
988.56174	1004.55577	(+16) Hydroxylation	
Characteristic Fragments			301, 327, 580, 612
Determinant patterns			Characteristic fragment for water loss (630 -18 = 612 m/z) of 23 and 24-OH<br>Characteristic fragment for water loss (598 -18 = 580 m/z) of 23 and 24-OH

11-Hydroxy Zotarolimus, m/z = 1004.55666

11-Hydroxy Zotarolimus, m/z = 1004.55666

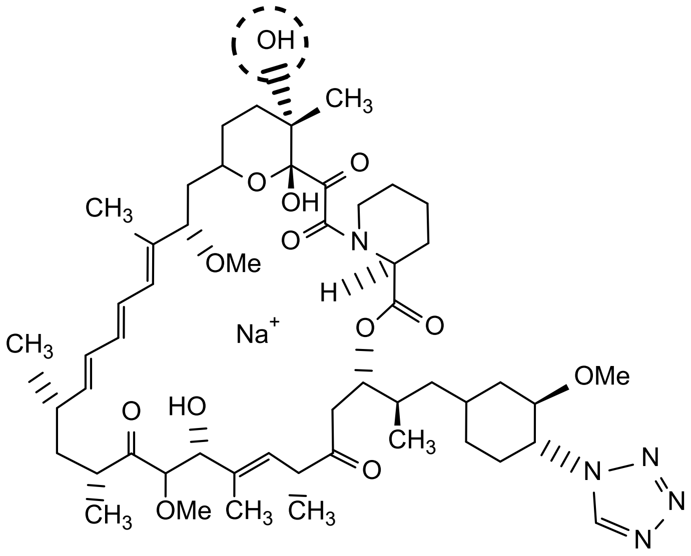

Chemical Formula: $C_{52}H_{79}N_5NaO_{13}^{+}$
Exact Mass: 1004.55666

11-hydroxy zotarolimus

11-Hydroxy Zotarolimus, m/z = 1004.55666
Extracted Ion Chromatogram (EIC)

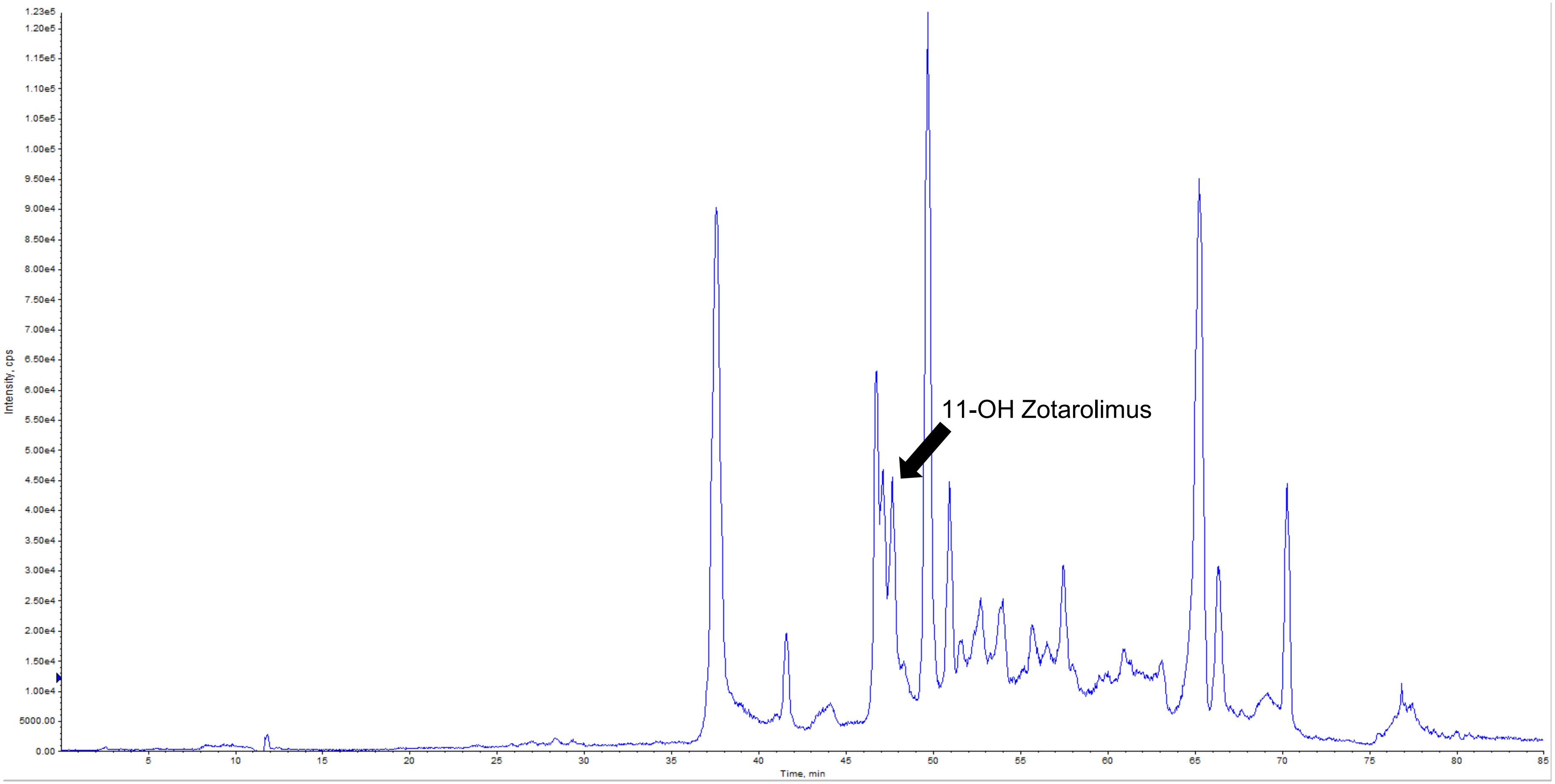

Mass Spectrum of 11-Hydroxy Zotarolimus
CE = 75 eV, DP = 125 V

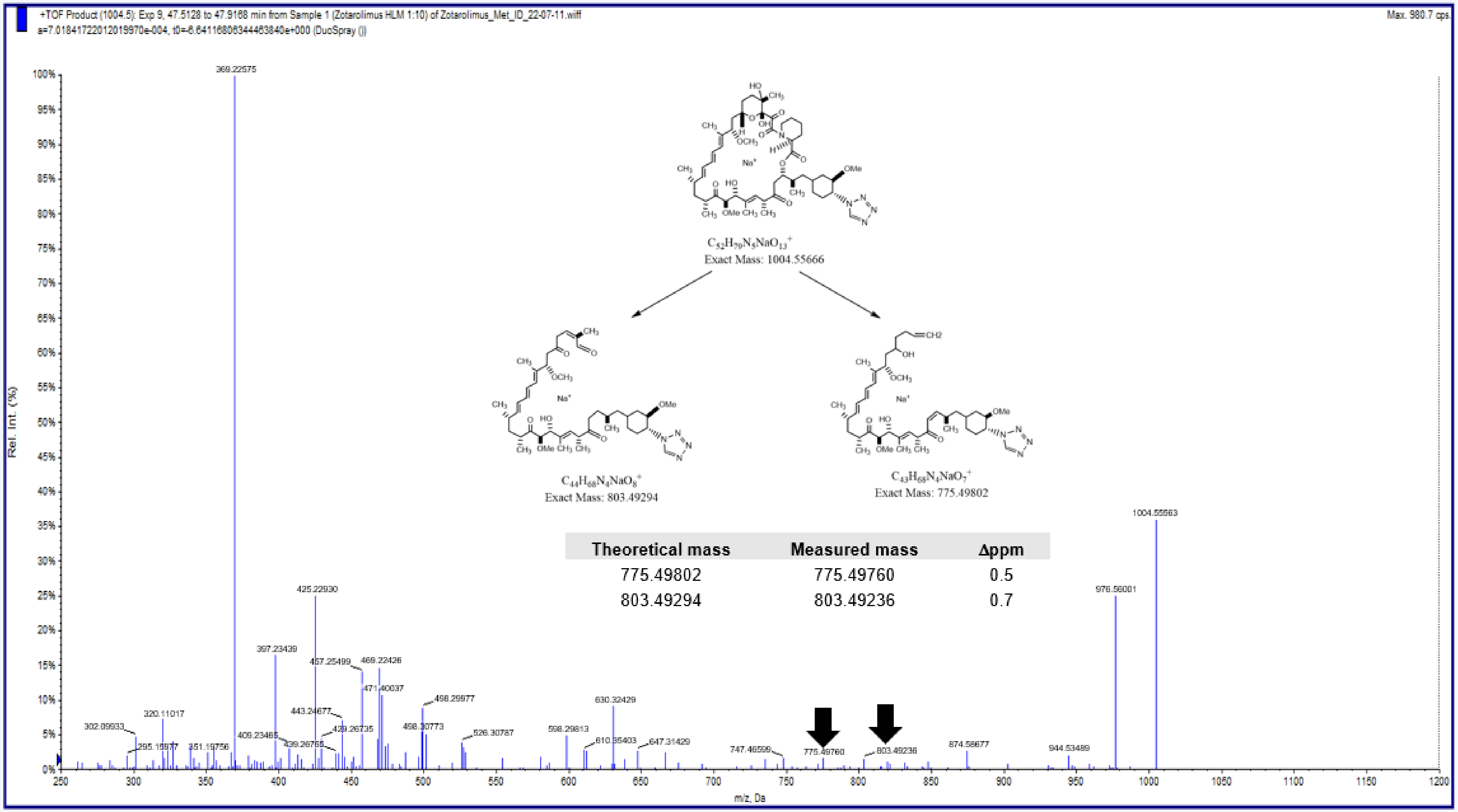

Theoretical mass	Measured mass	Δppm
775.49802	775.49760	0.5
803.49294	803.49236	0.7

Δppm of 11-Hydroxy Zotarolimus Fragments

Fragment No.	theoretical mass	measured mass	Δppm
1	302.09989	302.09933	1.9
2	369.22610	369.22575	0.9
3	397.23493	397.23439	1.4
4	413.22985	413.22967	0.4
5	409.23493	409.23465	0.7
6	457.25606	457.25499	2.3
7	469.22476	469.22426	1.1
8	598.29865	598.29813	0.9
9	630.32487	630.32429	0.9
10	677.42486	677.42409	1.1
11	710.33716	ND	NA
12	783.46672	ND	NA
13	815.49294	815.49237	0.7
14	1004.55666	1004.55630	0.4

Characteristic Fragments

Theoretical mass	Measured mass	Δppm
775.49802	775.49760	0.5
803.49297	803.49236	0.7

ND: not detected
NA: not applicable

11-Hydroxy Zotarolimus, m/z = 1004.55666

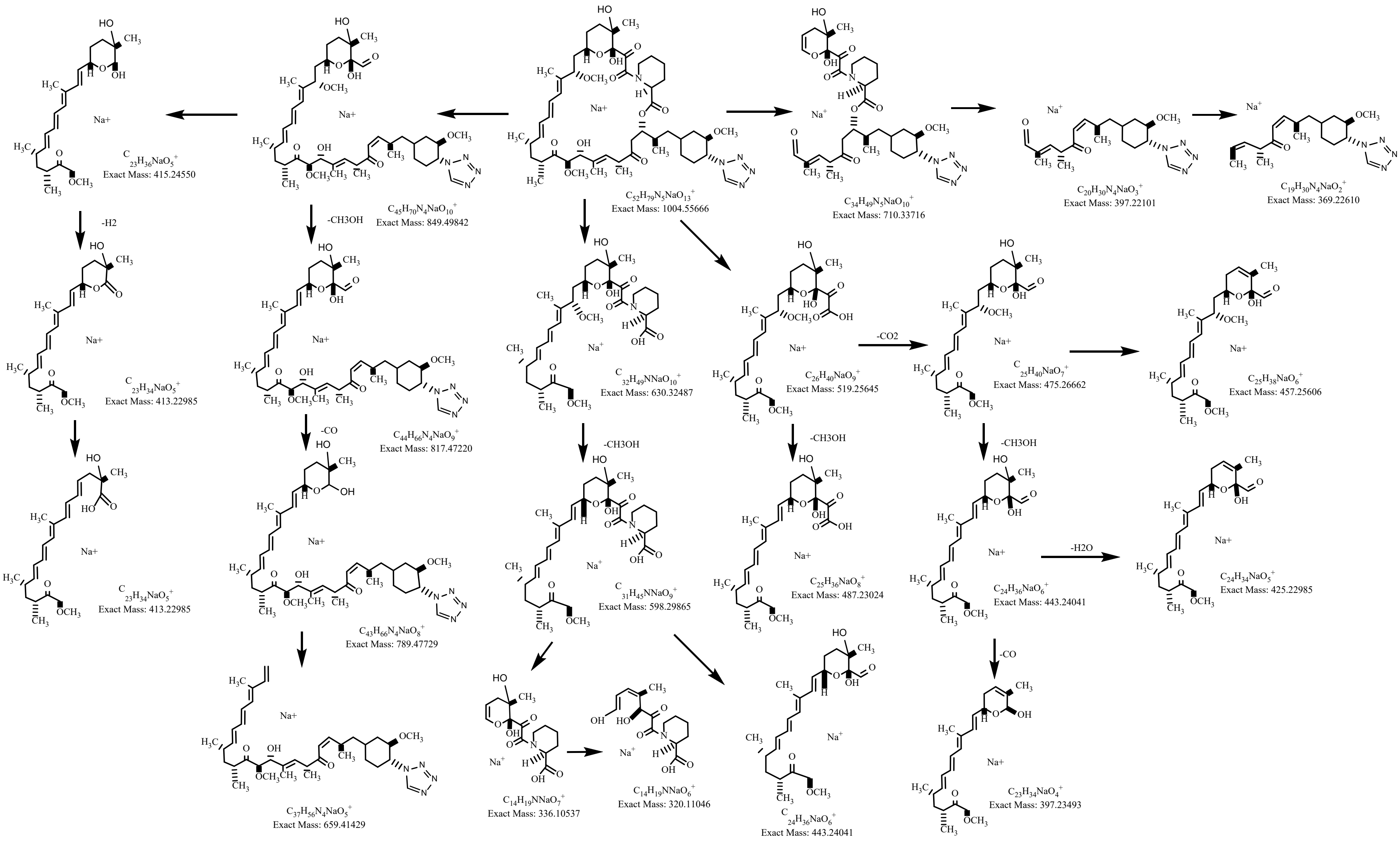

Characteristic Fragments of 11-Hydroxy Zotarolimus, m/z = 1004.55666

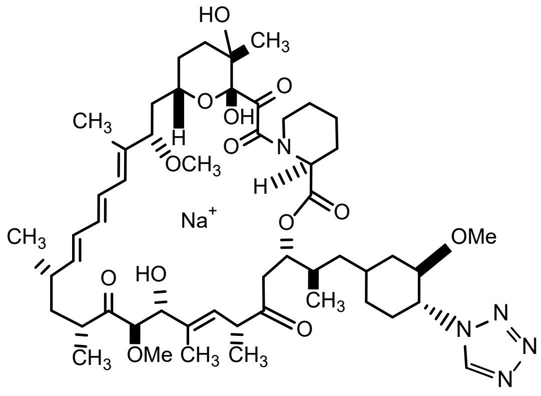

$C_{52}H_{79}N_5NaO_{13}^+$
Exact Mass: 1004.55666

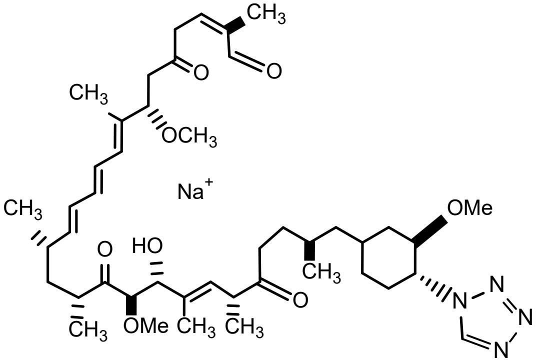

$C_{44}H_{68}N_4NaO_8^+$
Exact Mass: 803.49294

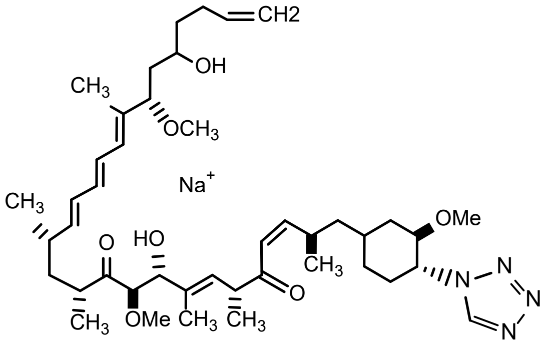

$C_{43}H_{68}N_4NaO_7^+$
Exact Mass: 775.49802

Structural Assessment of 11-Hydroxy Zotarolimus by Comparison of Fragmentation Patterns with those of Zotarolimus

Zotarolimus Fragments	11-Hydroxy Zotarolimus Fragments	Comments	
320.11046	320.11017	Hydroxylation with water elimination	11, 12, 14, and Piperidine-OH present
369.22610	369.22575	no hydroxylation	Rules out 47, 48, and 49-OH
381.24002	397.23439	(+16) Hydroxylation	11, 12, 14, 23, 24, 25, 45, or 46-OH present
397.23493	413.22967	(+16) Hydroxylation	11, 12, 14, 23, 24, 25, 45, or 46-OH present
409.23493	425.22930	(+16) Hydroxylation	11, 12, 14, 23, 24, 25, 45, or 46-OH present
427.24550	441.26103	(+16) Hydroxylation	11, 12, 14, 23, 24, 25, 45, or 46-OH present
453.22476	469.22426	(+16) Hydroxylation	11, 12, 14, 23, 24, 25, 45, or 46-OH present
582.30374	598.29813	(+16) Hydroxylation	11, 12, 14, 23, 24, 25, 45, 46-OH, or piperidine-OH present
614.32995	630.32429	(+16) Hydroxylation	11, 12, 14, 23, 24, 25, 45, 46-OH, or piperidine-OH present
659.41429	659.41397	no hydroxylation	Rules out 23, 24, 25, 45, 46, 47, 48 and 49-OH
694.34225	ND		NA
783.46672	ND		NA
988.56174	1004.55630	(+16) Hydroxylation	
Characteristic Fragments			775, 803
Determinant patterns			

ND: not detected
NA: not applicable

25-Hydroxy Zotarolimus, m/z = 1004.55666

25-Hydroxy Zotarolimus, m/z = 1004.55666

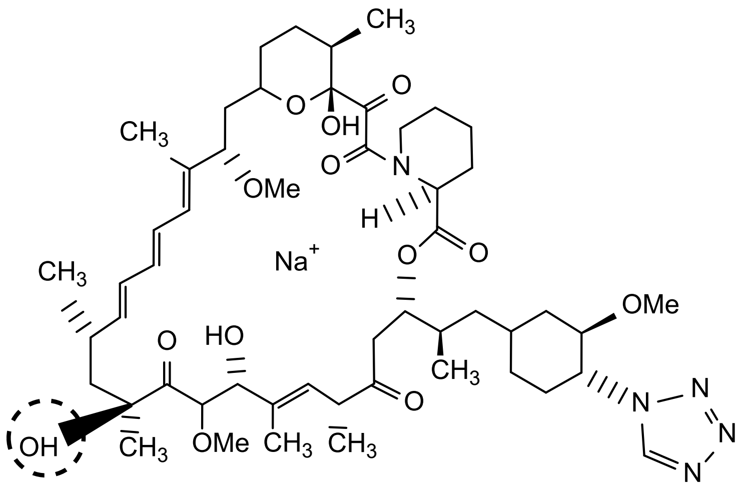

Chemical Formula: $C_{52}H_{79}N_5NaO_{13}^+$
Exact Mass: 1004.55666

25-hydroxy zotarolimus

25-Hydroxy Zotarolimus, m/z = 1004.55666
Extracted Ion Chromatogram (EIC)

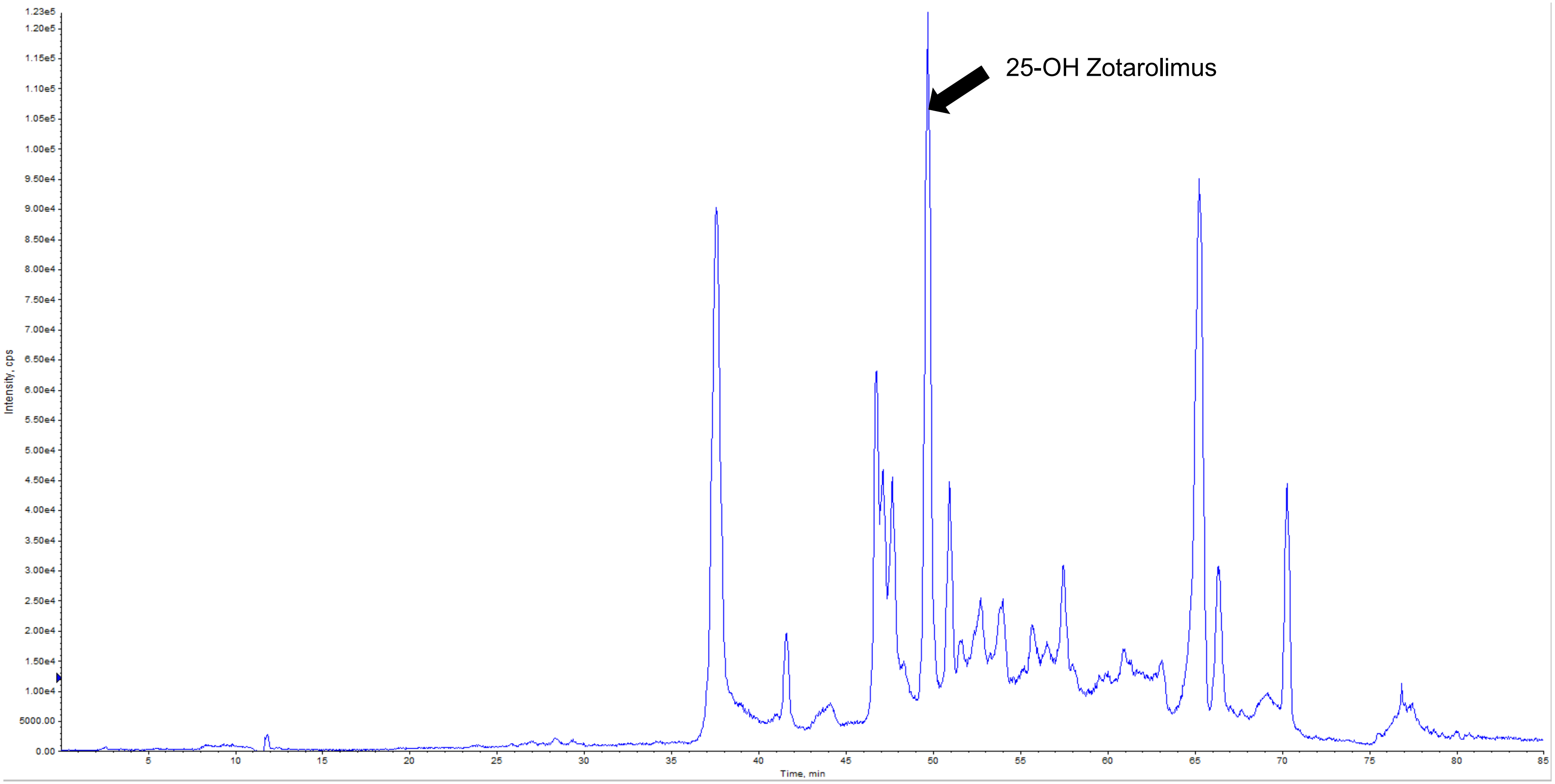

Mass Spectrum of 25-Hydroxy Zotarolimus
CE = 75 eV, DP = 125 V

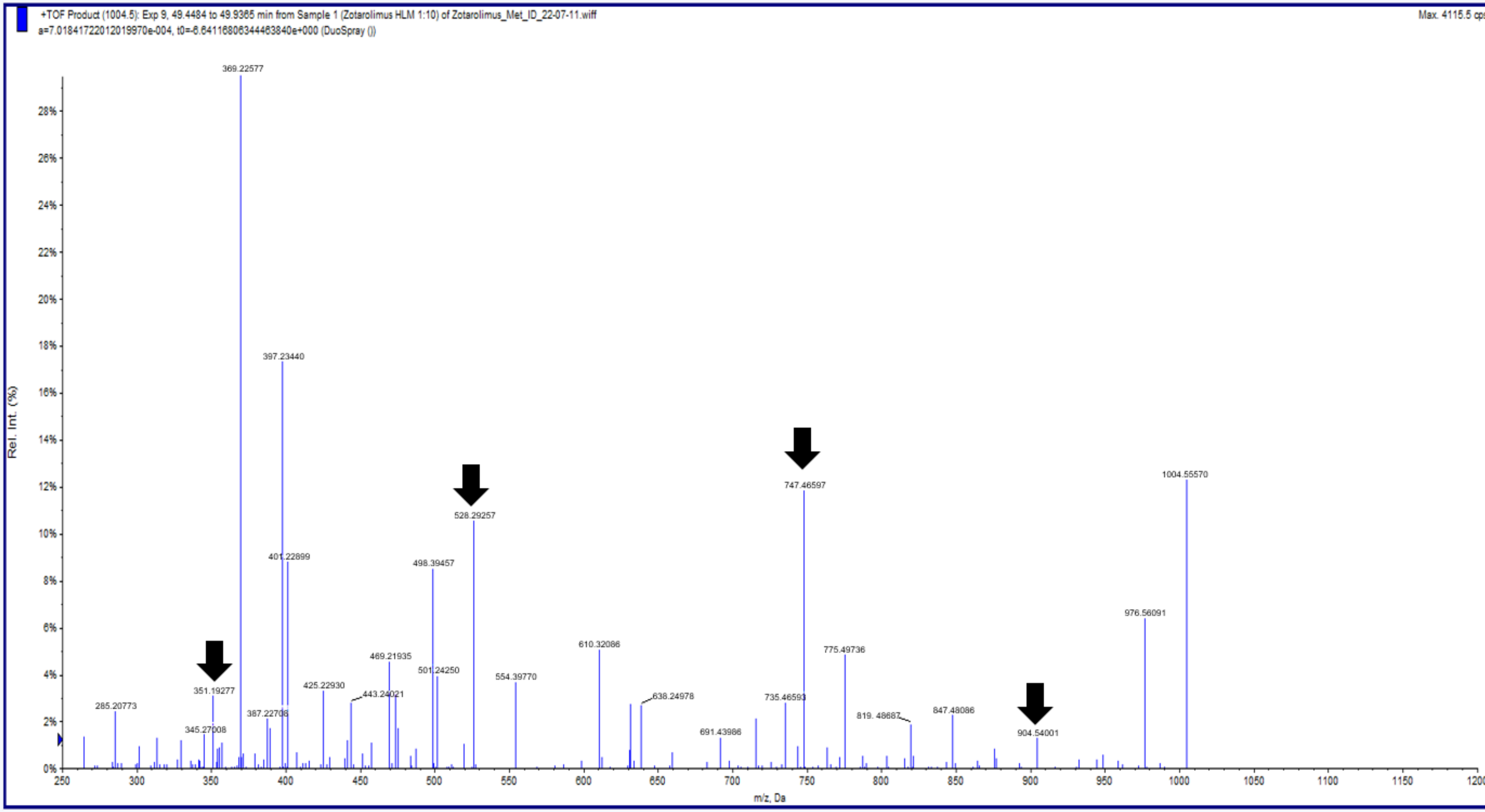

Δppm of 25-Hydroxy Zotarolimus Fragments

Fragment No.	Theoretical mass	Measured mass	Δppm
1	320.11046	Very low	NA
2	369.22610	369.22577	0.9
3	397.23493	397.23440	1.3
4	413.22985	413.22955	0.7
5	425.22985	425.22930	1.3
6	443.24041	443.24021	0.3
7	457.25606	457.25583	0.5
8	469.21967	469.21935	0.7
9	598.29865	598.29799	1.1
10	630.32487	630.32429	0.9
11	675.40921	ND	NA
12	694.34225	ND	NA
13	771.46672	ND	NA
14	904.54061	904.54001	0.7
15	1004.55666	1004.55570	1.0

	Theoretical mass	Measured mass	Δppm
Characteristic Fragments	351.19307	351.19277	0.9
	528.29317	528.29257	1.1
	904.54061	904.54001	0.7
	747.46672	747.46597	1.0

ND: not detected
NA: not applicable

25-Hydroxy Zotarolimus, m/z = 1004.55666

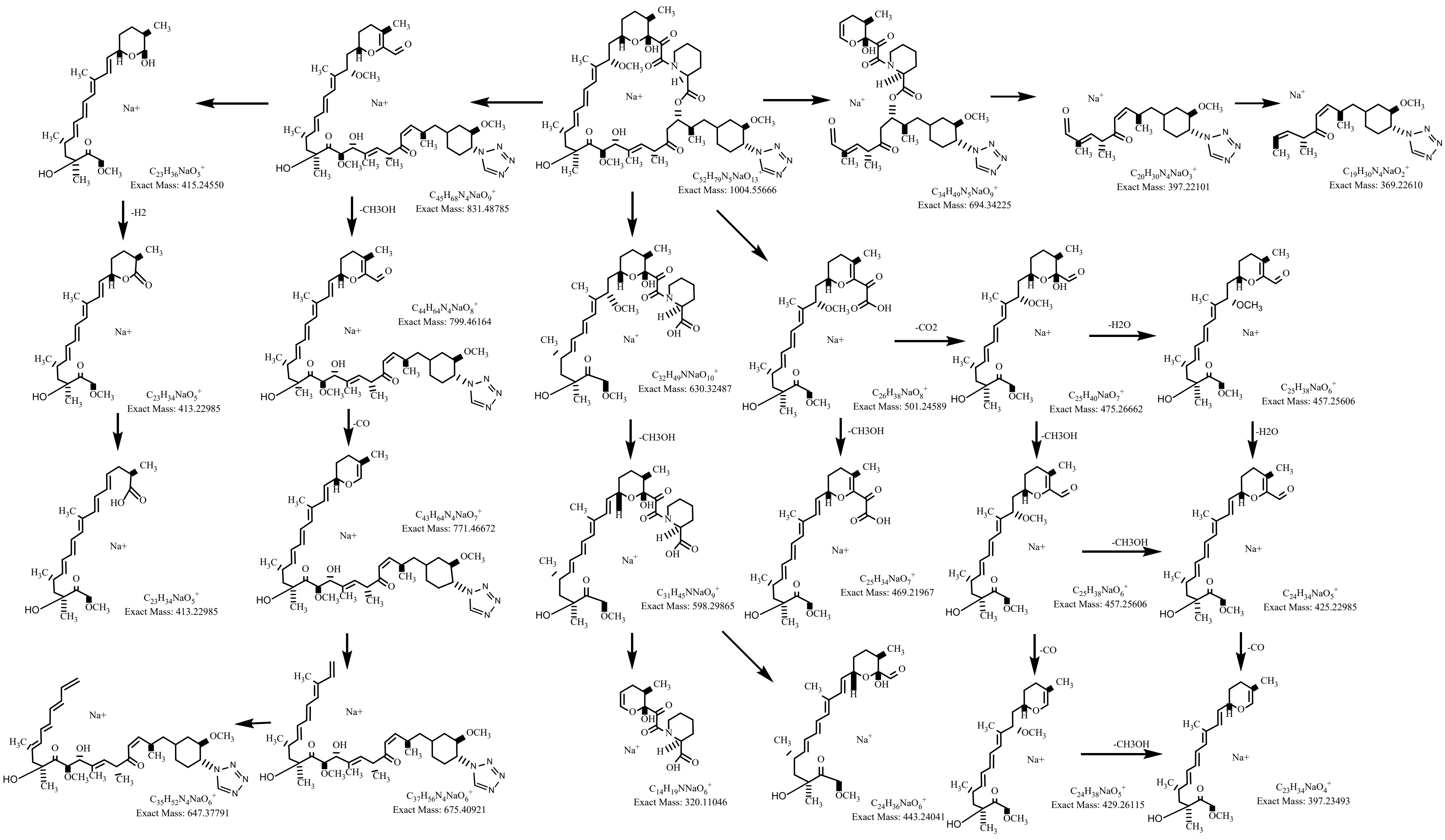

Characteristic Fragments of 25-Hydroxy Zotarolimus, m/z = 1004.55666

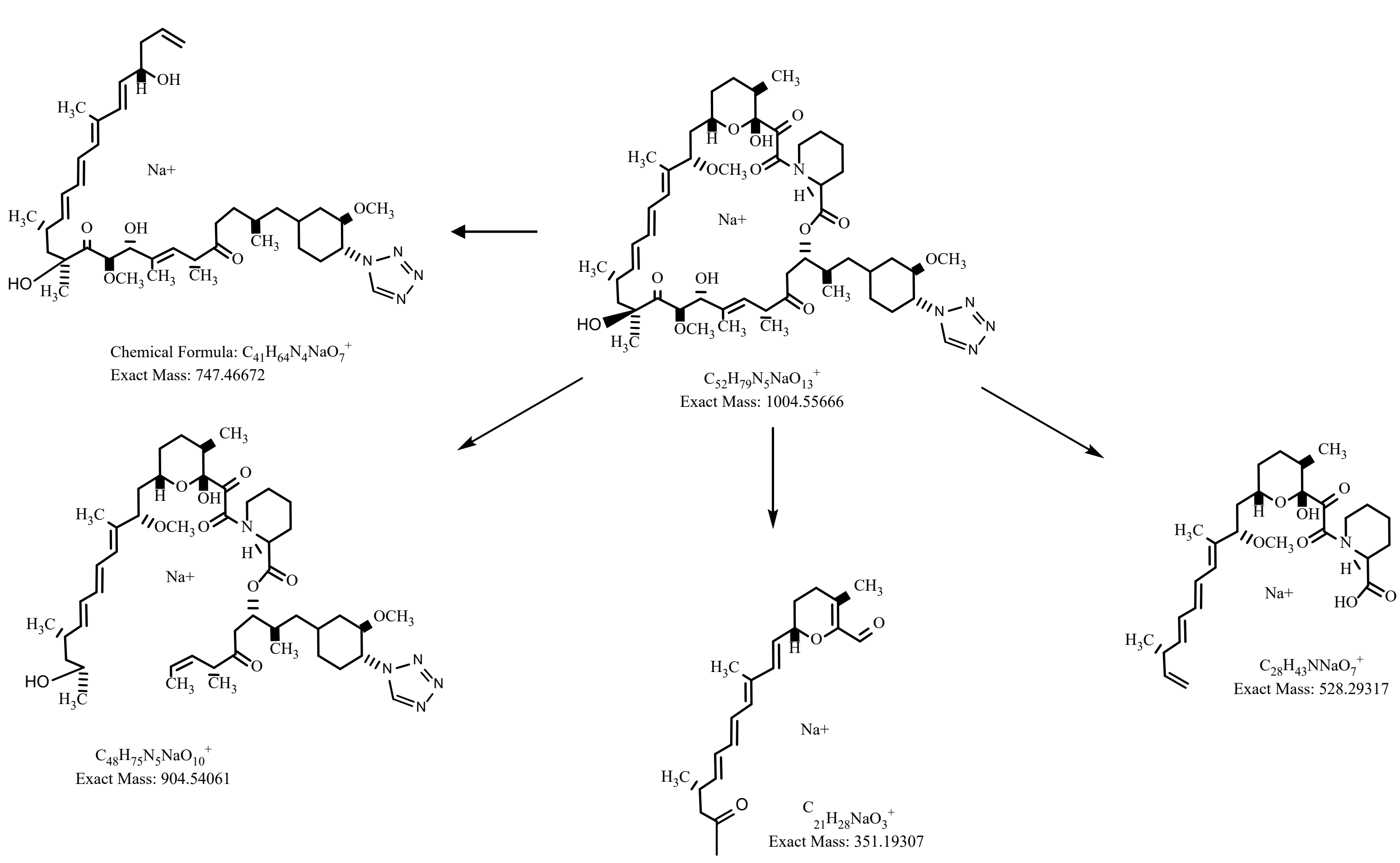

Structural Assessment of 25-Hydroxy Zotarolimus by Comparison of Fragmentation Patterns with those of Zotarolimus

Zotarolimus Fragments	25-Hydroxy Zotarolimus Fragments	Comments	
320.11046	320.11020	no hydroxylation	Rules out OH-piperidine, 11, 12, and 14-OH
369.22610	369.22577	no hydroxylation	Rules out 47, 48, and 49-OH
381.24002	397.23440	(+16) Hyroxylation	11, 12, 14, 23, 24, 25, 45, or 46-OH present
397.23493	397.23440	(+16) Hyroxylation	Rules out 47, 48, and 49-OH
409.23493	425.22930	(+16) Hyroxylation	11, 12, 14, 23, 24, 25, 45, or 46-OH present
427.24550	443.24021	(+16) Hyroxylation	11, 12, 14, 23, 24, 25, 45, or 46-OH present
441.26115	ND	(+16) Hyroxylation	NA
453.22476	469.21935	(+16) Hyroxylation	11, 12, 14, 23, 24, 25, 45, or 46-OH present
582.30374	598.29799	(+16) Hyroxylation	11, 12, 14, 23, 24, 25, 45, 46-OH, or piperidine-OH present
614.32995	630.32429	(+16) Hyroxylation	11, 12, 14, 23, 24, 25, 45, 46-OH, or piperidine-OH present
659.41429	ND	(+16) Hyroxylation	NA
694.34225	ND	no hydroxylation	NA
783.46672	799.46031	(+16) Hyroxylation	Rules out OH-piperidine
815.49294	ND	(+16) Hyroxylation	NA
988.56174	1004.5559	(+16) Hyroxylation	
Characteristic Fragments			351, 528, 904
Determinant patterns			

ND: not detected
NA: not applicable

Hydroxy-Piperidine Zotarolimus (I), m/z =1004.55666

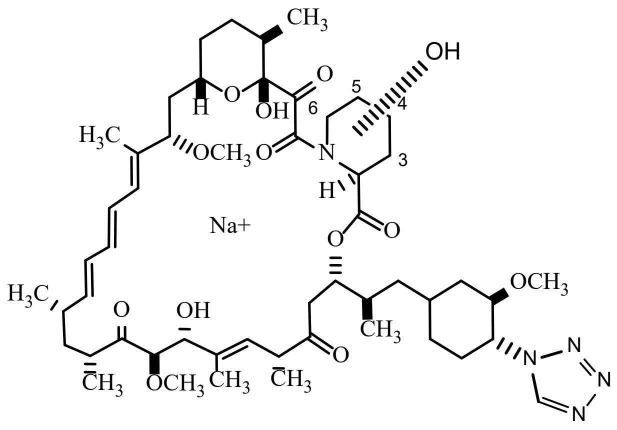

3/4/5/6-Hydroxy Piperidine Zotarolimus (I), m/z = 1004.55666
Extracted Ion Chromatogram (EIC)

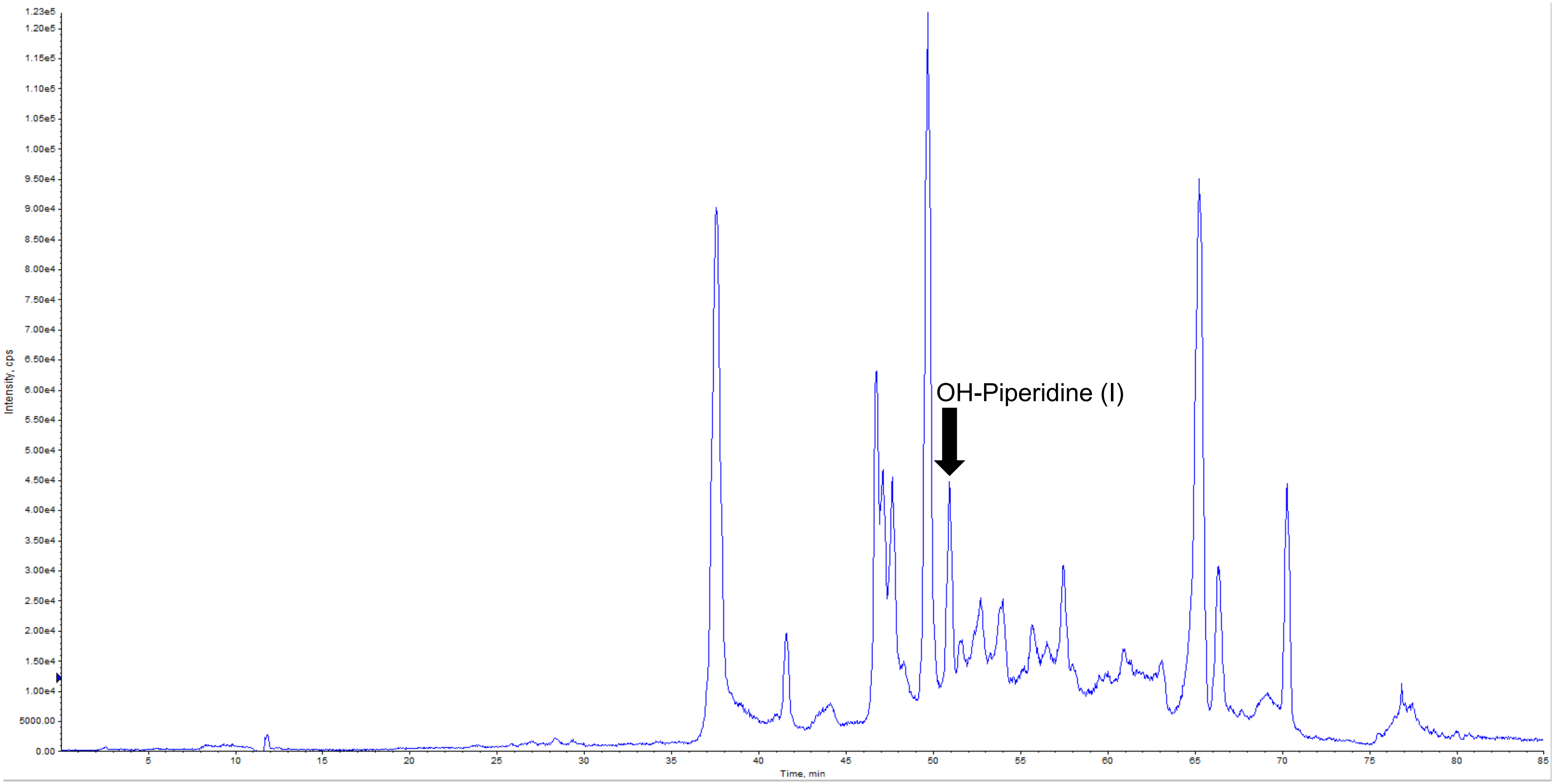

# Mass Spectrum of 3/4/5/6-Hydroxy Piperidine Zotarolimus (I)
CE = 75 eV, DP = 125 V

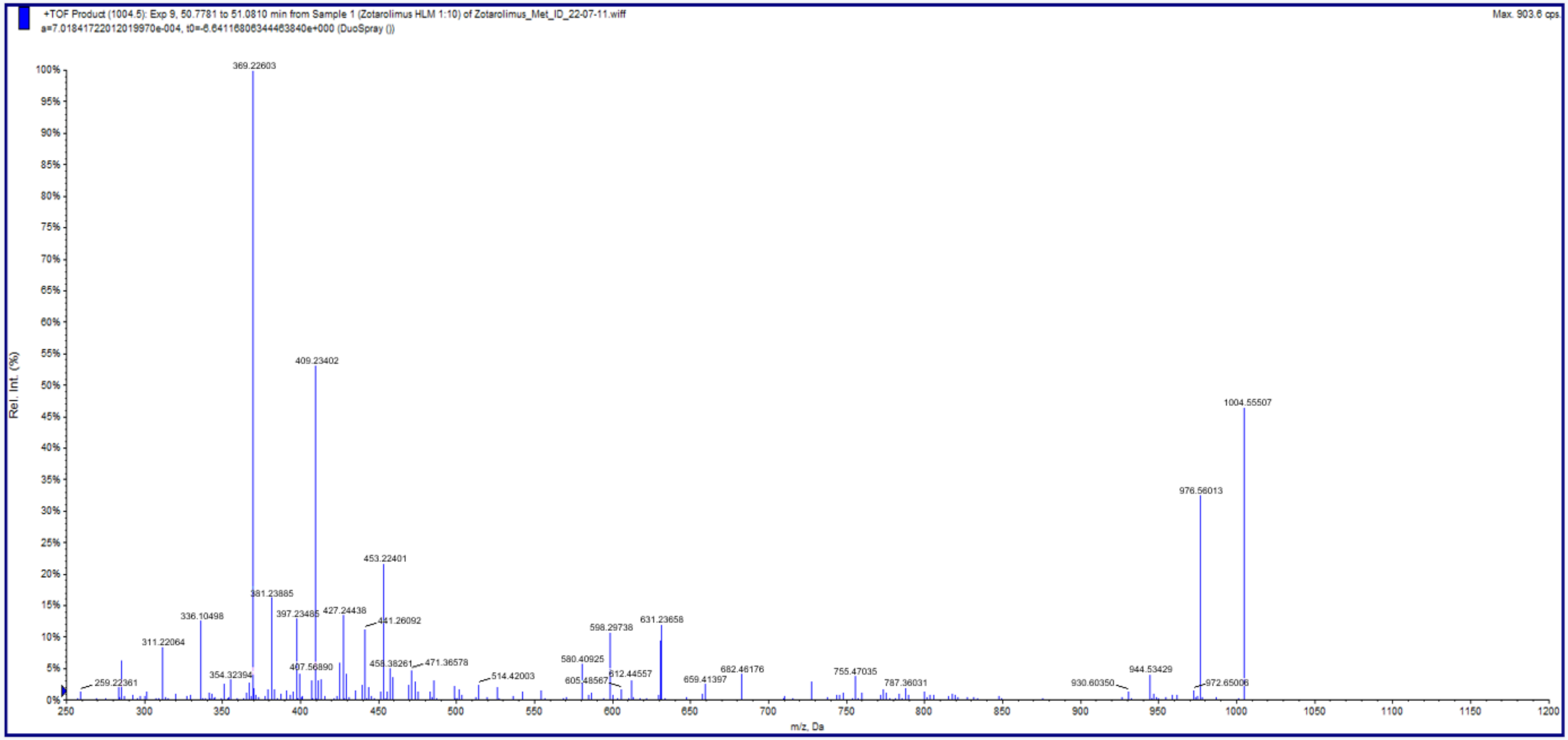

Δppm of Hydroxy-Piperidine Zotarolimus (I) Fragments

Fragment No.	Theoretical mass	Measured mass	Δppm
1	336.10537	336.10498	1.2
2	369.22610	369.22603	0.2
3	381.24002	381.23885	3.1
4	397.23493	397.23485	0.2
5	409.23493	409.23402	2.2
6	427.24550	427.24438	2.6
7	441.26115	441.26092	0.5
8	453.22476	453.22401	1.7
9	598.29865	598.29738	2.1
10	630.32487	630.32397	1.4
11	659.41429	659.41397	0.5
12	710.33716	710.33681	0.5
13	783.46672	783.46537	1.7
14	815.49294	815.49203	1.1
15	1004.55666	1004.55507	1.6

Fragmentation Pattern of 3/4/5/6-Hydroxy Piperidine Zotarolimus, m/z = 1004.55666

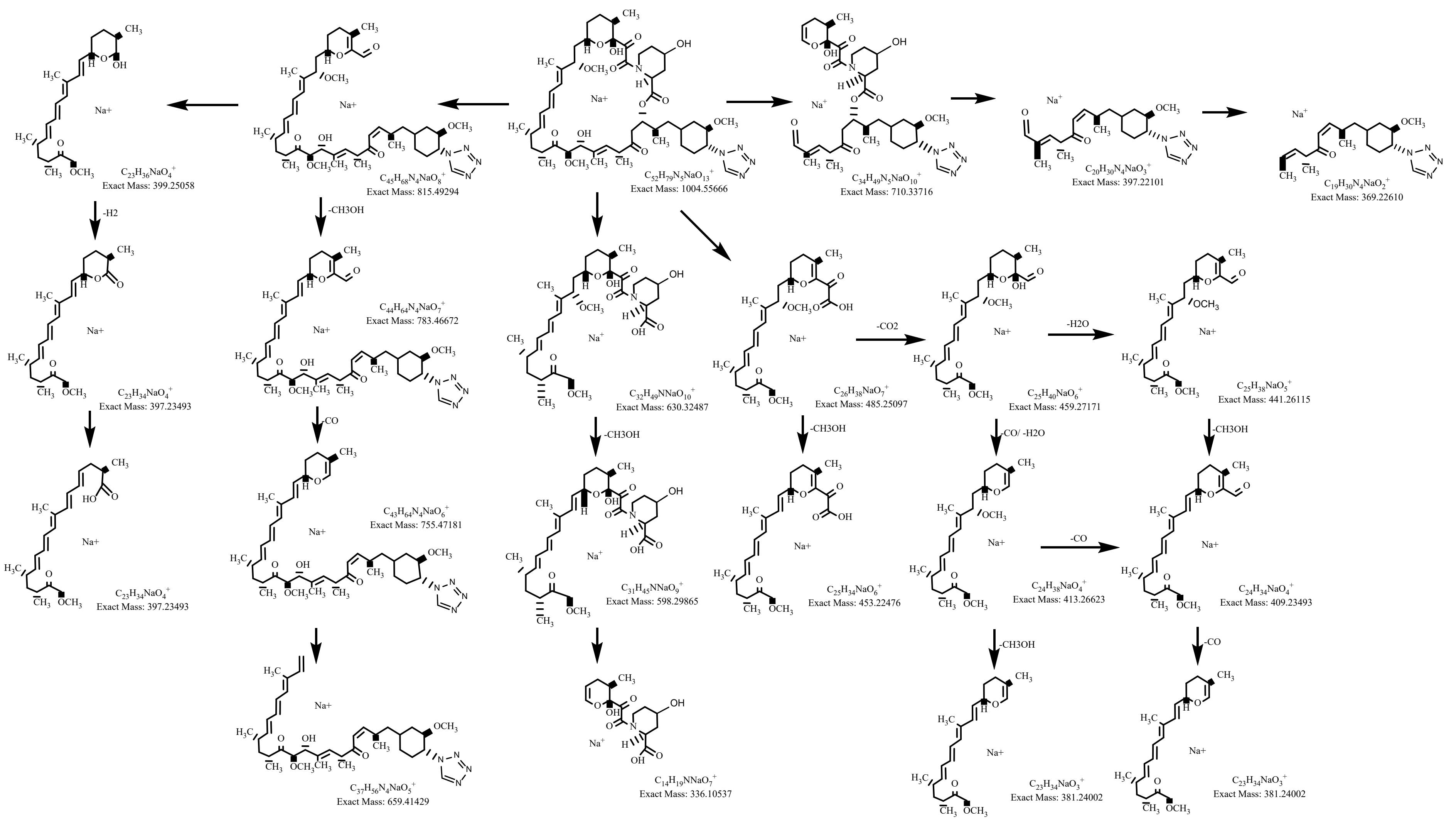

Structural Confirmation of Hydroxy Piperidine (I) Zotarolimus, m/z = 1004.55666

Zotarolimus Fragments	Pip-OH Zotarolimus (I) Fragments	Comments	
320.11046	336.10498	(+16) Hyroxylation	11, 12, or OH-piperidine
369.22610	369.22603	no hydroxylation	Rules out 47, 48, and 49-OH
381.24002	381.23885	no hydroxylation	Rules out 11, 12, 14, 23, 24, 25, 45, or 46-OH present
397.23493	397.23485	no hydroxylation	Rules out 11, 12, 14, 23, 24, 25, 45, or 46-OH present
409.23493	409.23402	no hydroxylation	Rules out 11, 12, 14, 23, 24, 25, 45, or 46-OH present
441.26115	441.26092	no hydroxylation	Rules out 11, 12, 14, 23, 24, 25, 45, or 46-OH present
453.22476	453.22401	no hydroxylation	Rules out 11, 12, 14, 23, 24, 25, 45, or 46-OH
582.30374	598.29738	(+16) Hyroxylation	11, 12, 14, 23, 24, 25, 45, 46-OH, or piperidine-OH present
614.32995	630.32397	(+16) Hyroxylation	11, 12, 14, 23, 24, 25, 45, 46-OH, or piperidine-OH present
659.41429	659.41397	no hydroxylation	Rules out 23, 24, 25, 45, 46, 47, 48, or 49-OH
694.34225	710.33681	(+16) Hyroxylation	11, 12, 14, 48, 49, 50, or Pip-OH
783.46672	783.46537	no hydroxylation	Rules out 11, 12, 14, 23, 24, 25, 45, 46, 47, 48, or 49-OH present
815.49294	815.49203	no hydroxylation	Rules out 11, 12, 14, 23, 24, 25, 45, 46, 47, 48, or 49-OH present
988.56174	1004.55507	(+16) Hyroxylation	
Characteristic Fragments			336
Determinant patterns			

14-Hydroxy Zotarolimus, m/z = 1004.55666

14-Hydroxy Zotarolimus, m/z = 1004.55666

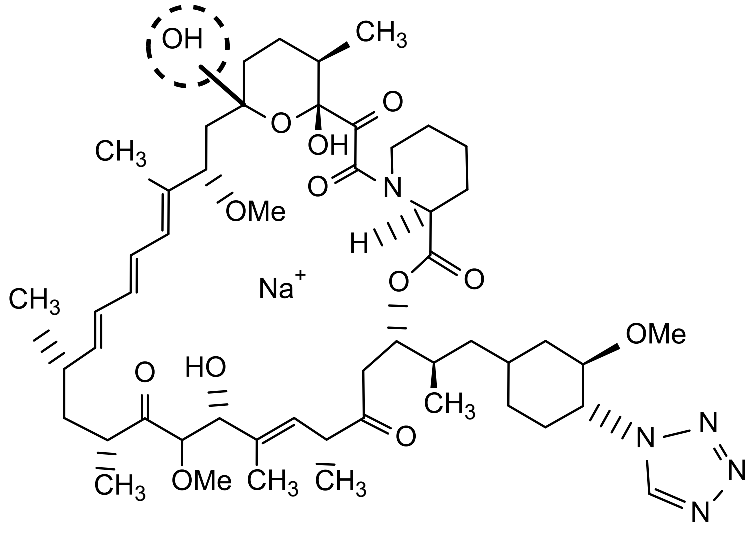

Chemical Formula: $C_{52}H_{79}N_5NaO_{13}^+$
Exact Mass: 1004.55666

14-hydroxy zotarolimus

14-Hydroxy Zotarolimus, m/z = 1004.55666
Extracted Ion Chromatogram (EIC)

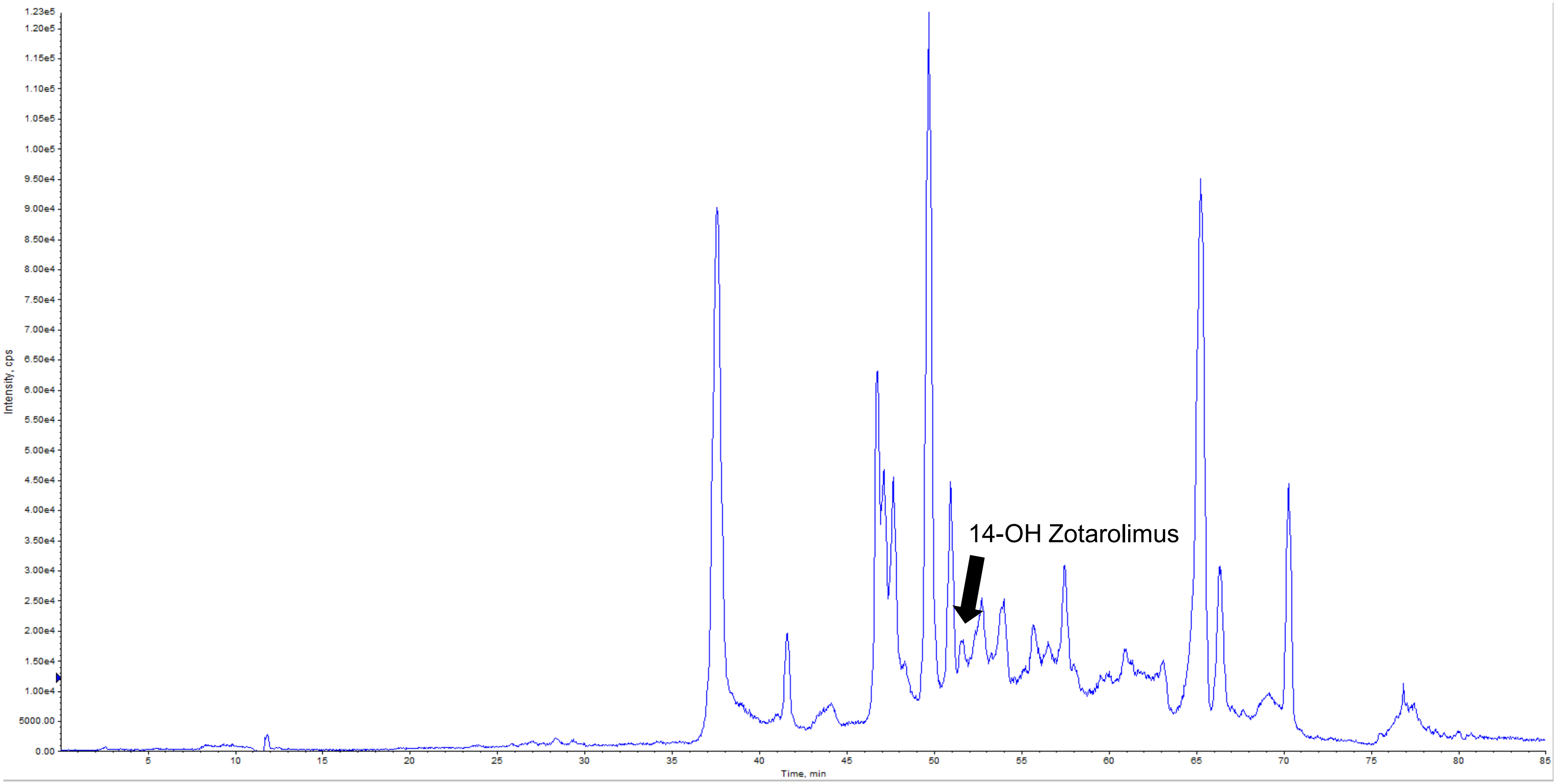

Mass Spectrum of 14-Hydroxy Zotarolimus
CE = 75 eV, DP = 125 V

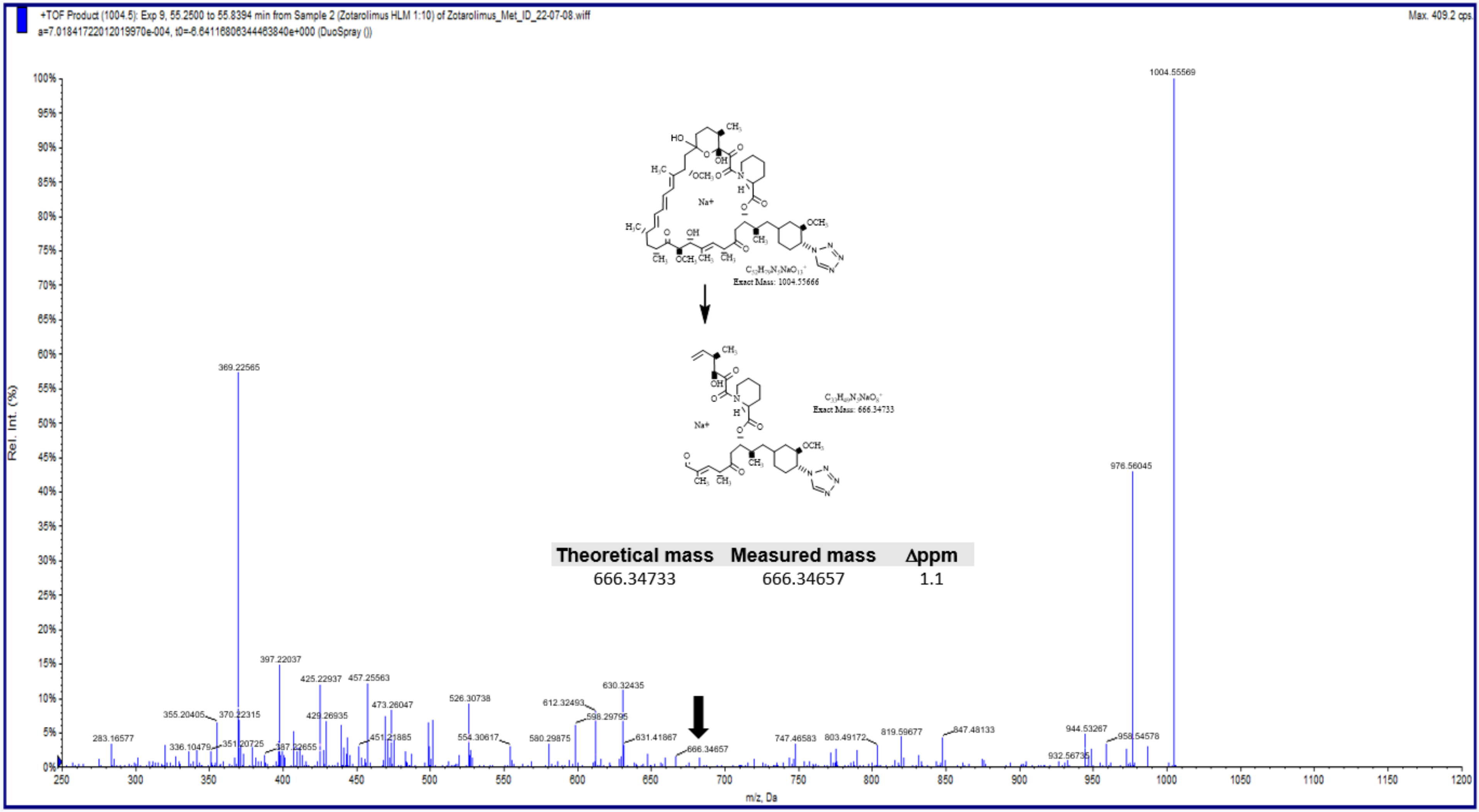

Theoretical mass	Measured mass	Δppm
666.34733	666.34657	1.1

Δppm of 14-Hydroxy Zotarolimus Fragments

Fragment No.	Theoretical mass	Measured mass	Δppm
1	320.11046	320.11009	1.2
2	336.10537	336.10479	1.7
3	369.2261	369.22565	1.2
4	397.22101	397.22037	1.6
5	413.22985	413.22935	1.2
6	425.22985	425.22937	1.1
7	457.25606	457.25563	0.9
8	475.26662	475.26579	1.7
9	598.29865	598.29795	1.2
10	630.32487	630.32435	0.8
11	659.41429	659.41377	0.8
12	803.49294	803.49172	1.5
13	1004.55666	1004.55569	1.0

	theoretical mass	measured mass	Δppm
Characteristic Fragment	666.34733	666.34657	1.1

Fragmentation Pattern of 14-Hydroxy Zotarolimus, m/z = 1004.55666

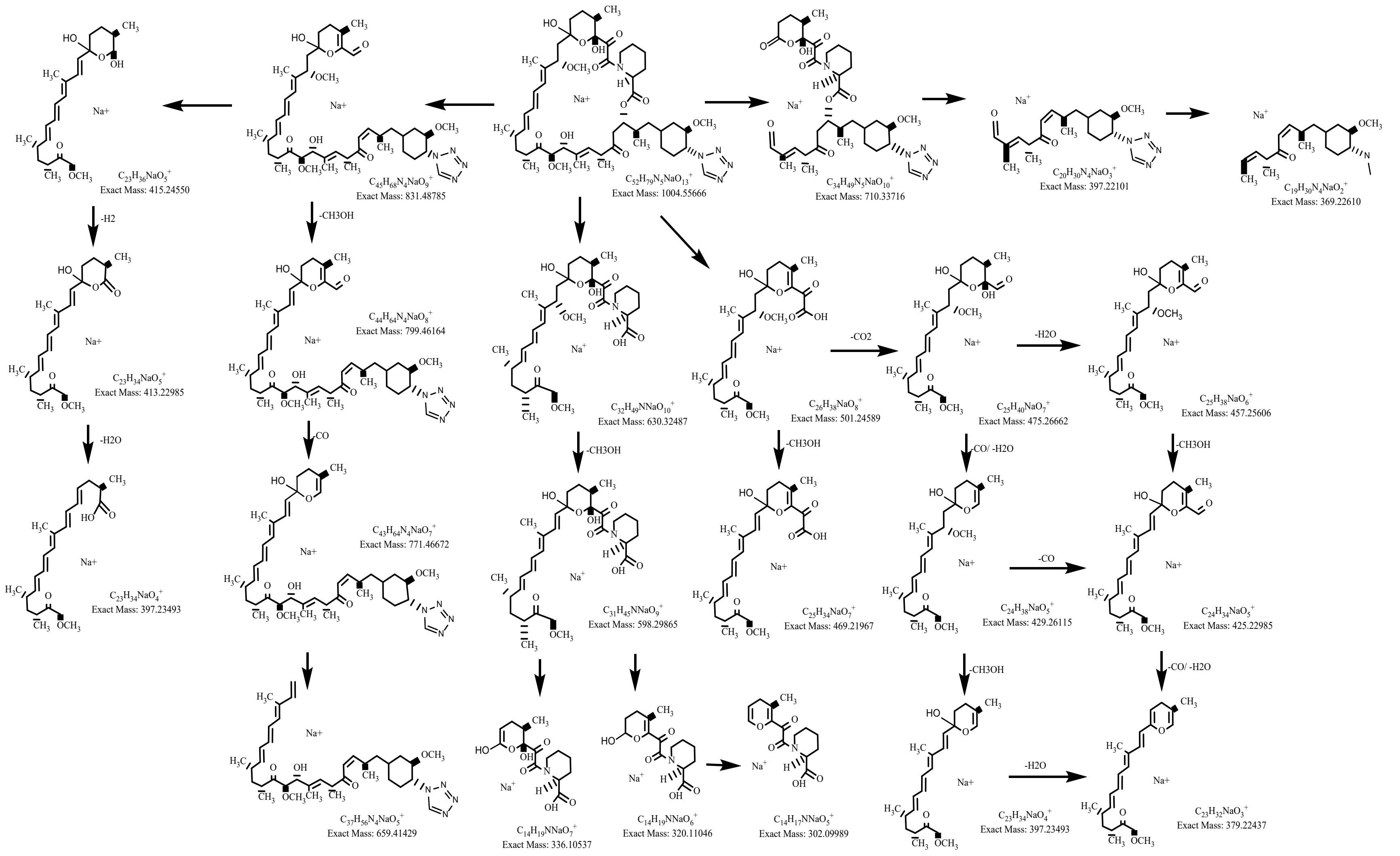

Characteristic Fragment of 14-Hydroxy Zotarolimus, m/z = 1004.55666

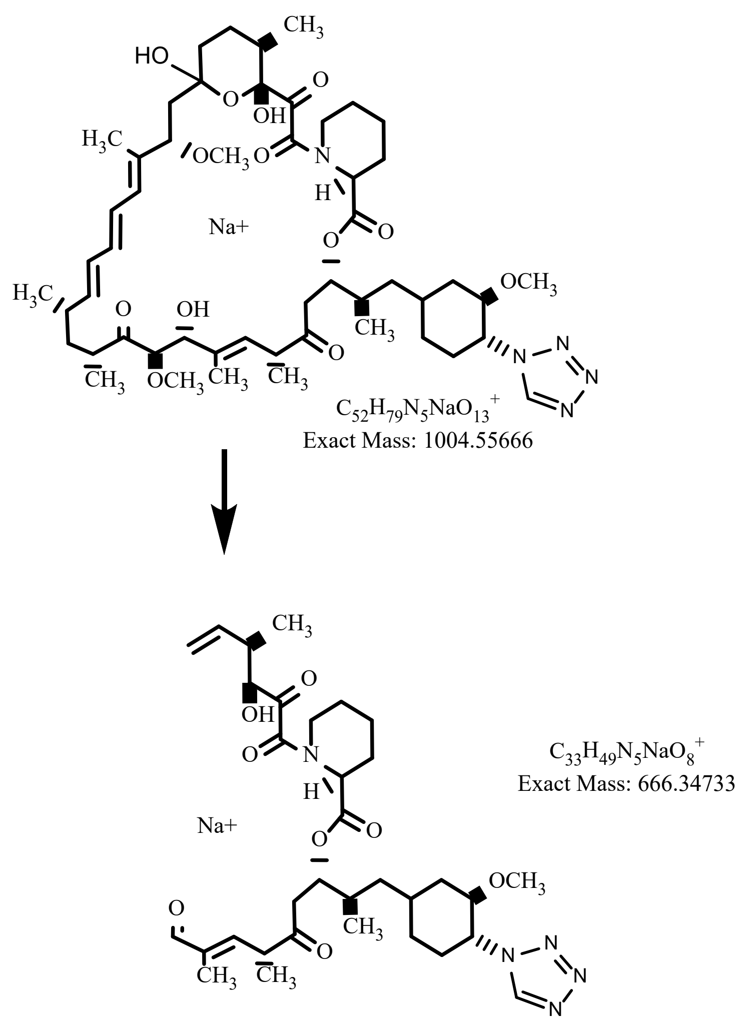

Structural Confirmation of 14-Hydroxy Zotarolimus, m/z = 1004.55666

Zotarolimus Fragments	14-Hydroxy Zotarolimus Fragments	Comments	
320.11046	336.10479	(+16) Hydroxylation	11, 12, 14, and Piperidine-OH present
369.22610	369.22565	No Hydroxylation	Rules out 47, 48, and 49-OH
381.24002	397.22037	(+16) Hydroxylation	11, 12, 14, 23, 24, 25, 45, or 46-OH present
397.23493	397.22037	No Hydroxylation	Rules out 47, 48, and 49-OH
441.26115	457.25563	(+16) Hydroxylation	11, 12, 14, 23, 24, 25, 45, or 46-OH present
459.27171	475.26579	(+16) Hydroxylation	11, 12, 14, 23, 24, 25, 45, or 46-OH present
582.30374	598.29795	(+16) Hydroxylation	11, 12, 14, 23, 24, 25, 45, 46-OH, or piperidine-OH present
614.32995	630.32435	(+16) Hydroxylation	11, 12, 14, 23, 24, 25, 45, 46-OH, or piperidine-OH present
659.41429	659.41377	No Hydroxylation	Rules out 23, 24, 25, 45, 46, 47, 48, and 49-OH
988.56174	1004.55569	(+16) Hydroxylation	
Characteristic Fragments			666
Determinant patterns			

Hydroxy-Piperidine Zotarolimus (II), m/z =1004.55666

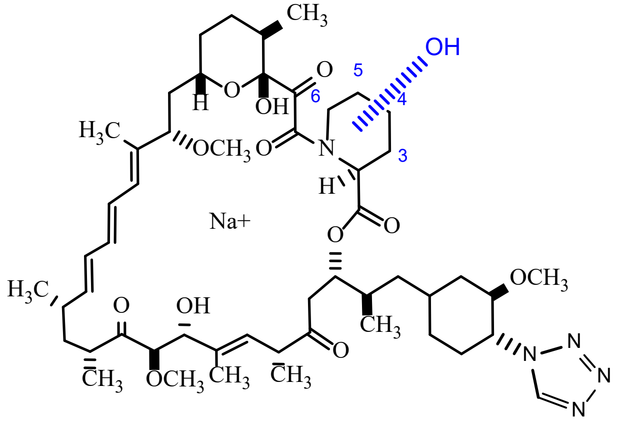

3/4/5/6-Hydroxy Piperidine Zotarolimus (II), m/z = 1004.55666
Extracted Ion Chromatogram (EIC)

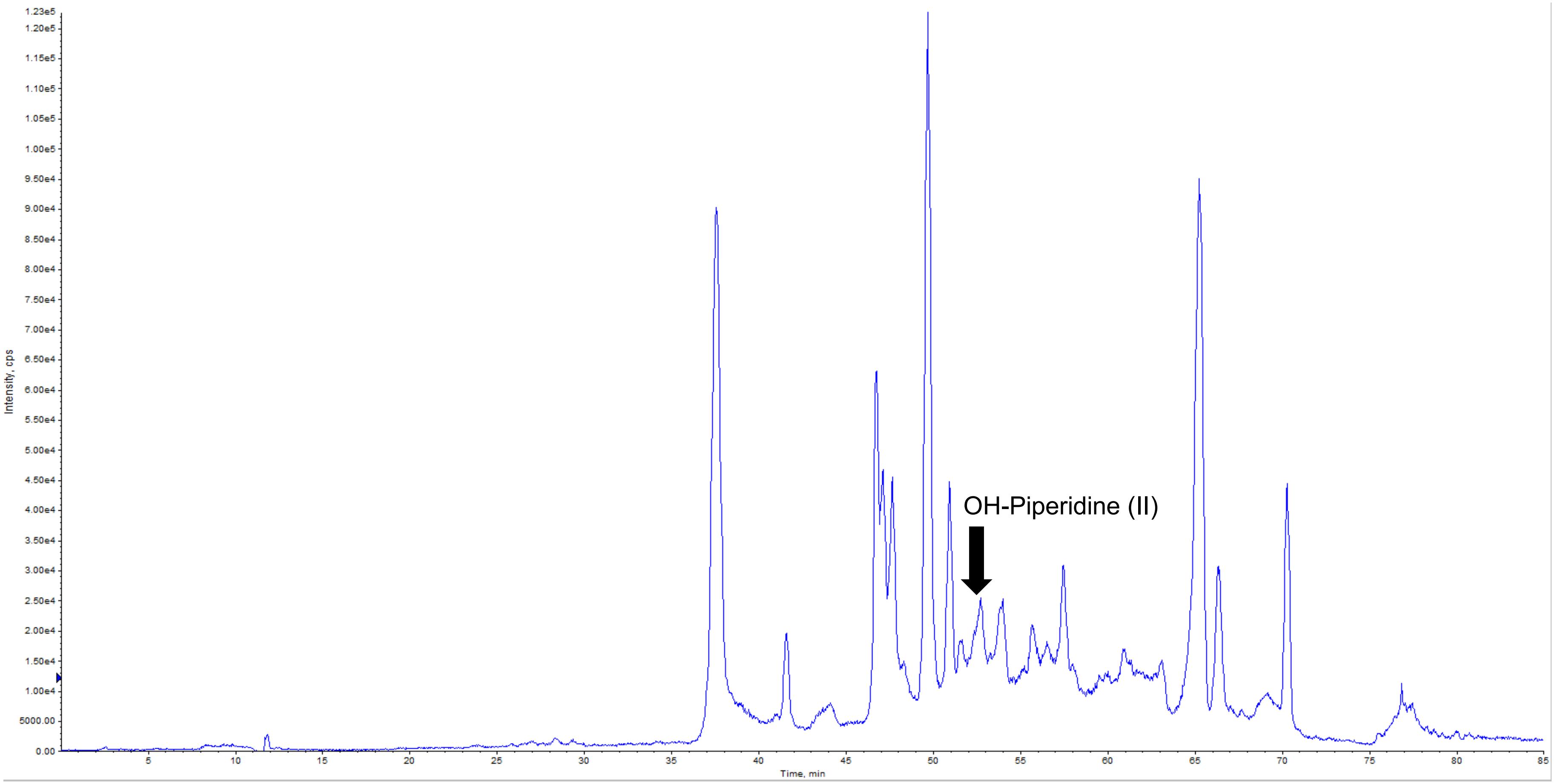

Mass Spectrum of 3/4/5/6-Hydroxy Piperidine Zotarolimus (II)

CE = 75 eV, DP = 125 V

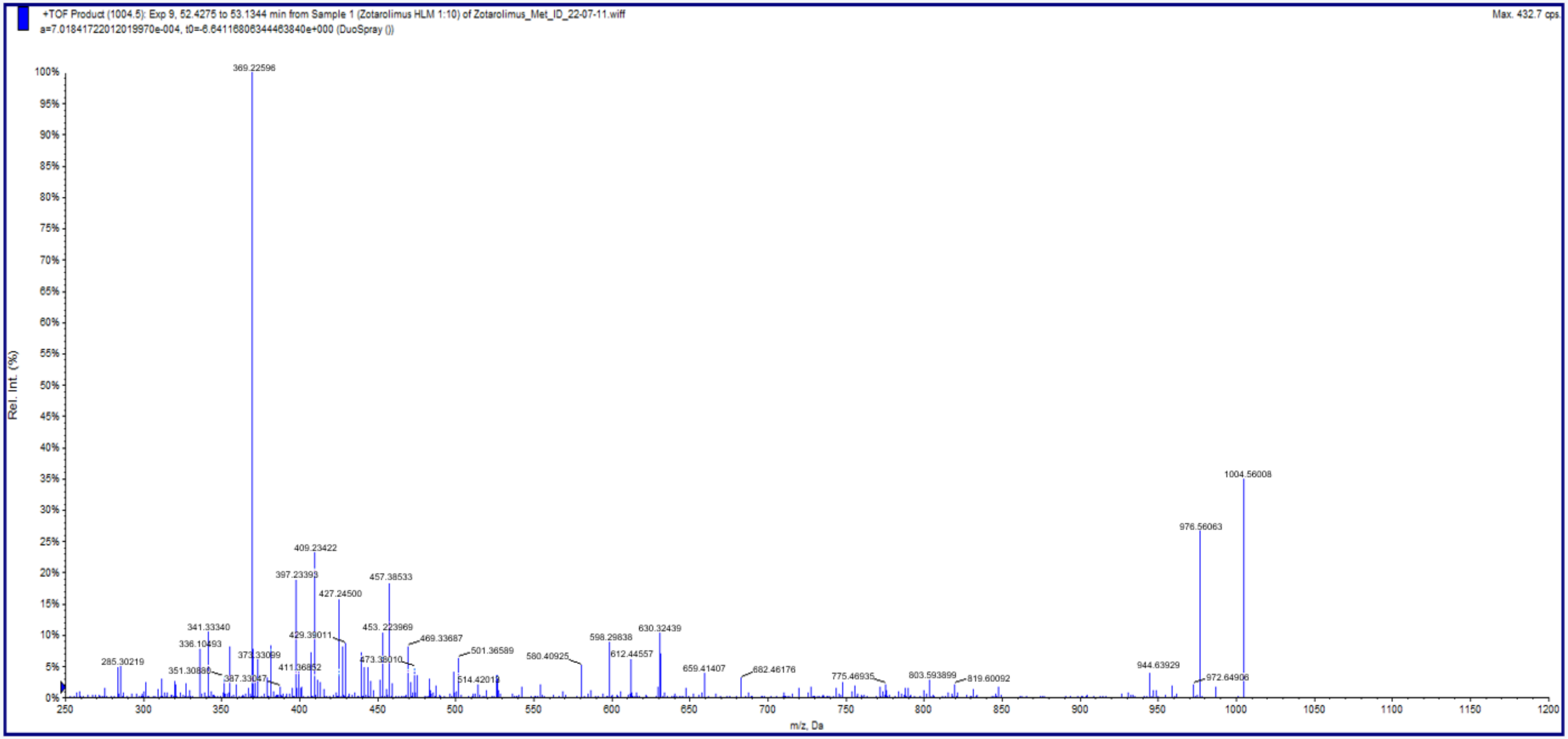

Δppm of Hydroxy-Piperidine (II) Zotarolimus Fragments

Fragment No.	Theoretical mass	Measured mass	Δppm
1	336.10537	336.10493	1.3
2	369.22610	369.22596	0.4
3	381.24002	381.23885	3.1
4	397.23493	397.23393	2.5
5	409.23493	409.23422	1.7
6	427.24550	427.24500	1.2
7	441.26115	441.26092	0.5
8	453.22476	453.22369	2.4
9	598.29865	598.29838	0.5
10	630.32487	630.32439	0.8
11	659.41429	659.41407	0.3
12	710.33716	710.33699	0.2
13	783.46672	783.46637	0.4
14	815.49294	815.49103	2.3
15	1004.55666	1004.56008	-3.4

3/4/5/6-Hydroxy Piperidine (II) Zotarolimus, m/z = 1004.55666

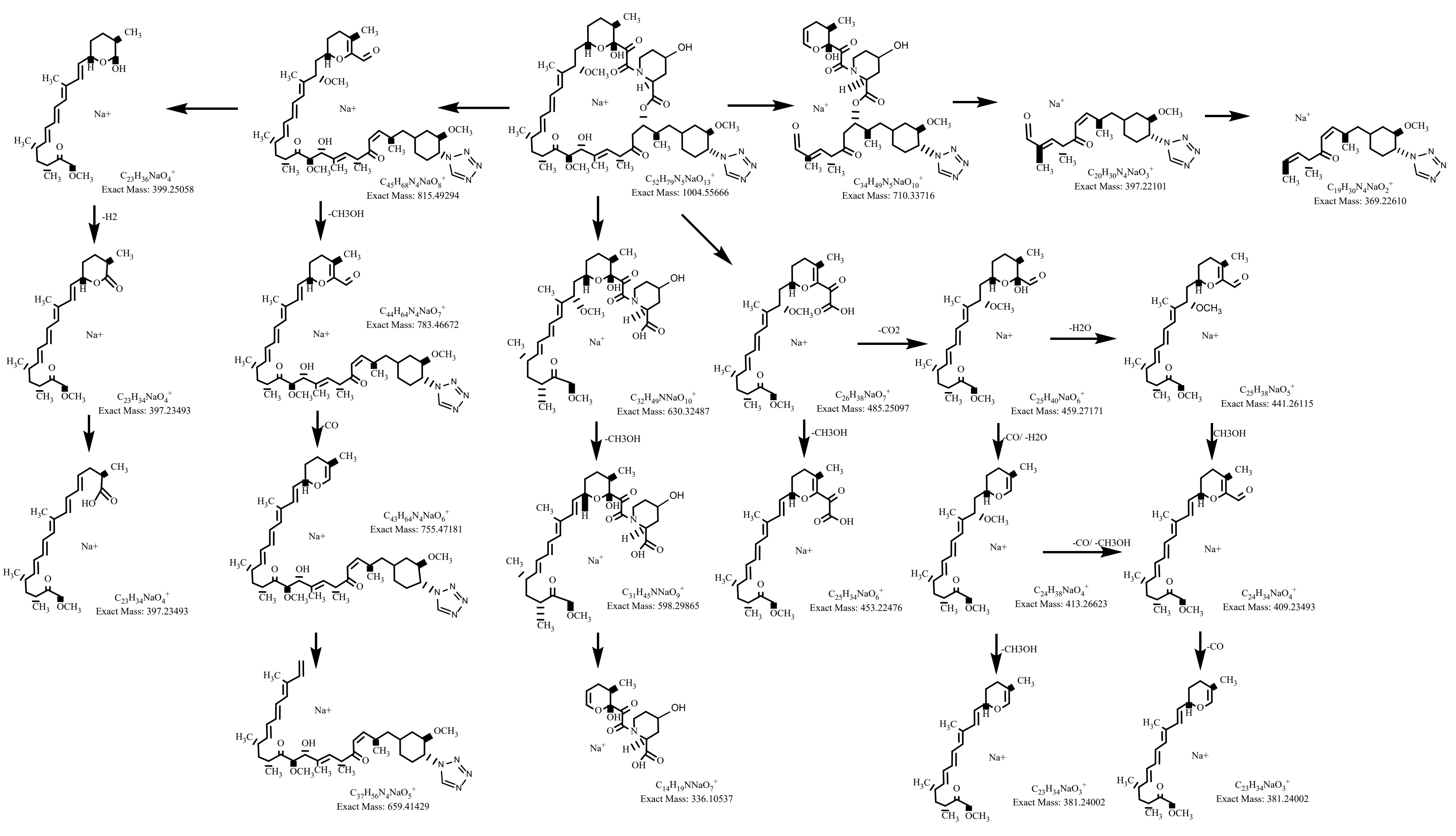

Structural Confirmation of Hydroxy Piperidine (II) Zotarolimus, m/z = 1004.55666

Zotarolimus Fragments	Pip-OH (II) Zotarolimus Fragments	Comments	
320.11046	336.10493	+16) Hydroxylation	11-, 12-, 14-OH, or OH-piperidine present
369.22610	369.22596	no hydroxylation	Rules out 47, 48, and 49-OH
381.24002	381.23885	no hydroxylation	Rules out 11, 12, 14, 23, 24, 25, 45, or 46-OH
397.23493	397.23393	no hydroxylation	Rules out 11, 12, 14, 23, 24, 25, 45, or 46-OH
409.23493	409.23422	no hydroxylation	Rules out 11, 12, 14, 23, 24, 25, 45, or 46-OH
441.26115	441.26092	no hydroxylation	Rules out 11, 12, 14, 23, 24, 25, 45, or 46-OH
453.22476	453.22369	no hydroxylation	Rules out 11, 12, 14, 23, 24, 25, 45, or 46-OH
582.30374	598.29838	+16) Hydroxylation	11, 12, 14, 23, 24, 25, 45, 46-OH, or piperidine-OH present
614.32995	630.32439	+16) Hydroxylation	11, 12, 14, 23, 24, 25, 45, 46-OH, or piperidine-OH present
659.41429	659.41407	no hydroxylation	Rules out 23, 24, 25, 45, 46, 47, 48, or 49-OH
694.34225	710.33699	(+16) Hydroxylation	11, 12, 14, 47, 48, 49, or Pip-OH present
783.46672	783.46637	no hydroxylation	Rules out 11, 12, 14, 23, 24, 25, 45, 46, 47, 48, or 49-OH
815.49294	815.49103	no hydroxylation	Rules out 11, 12, 14, 23, 24, 25, 45, 46, 47, 48, or 49-OH
988.56174	1004.56008	+16) Hydroxylation	
Characteristic Fragments			336
Determinant patterns			

Hydroxy-Piperidine Zotarolimus (III), m/z =1004.55666

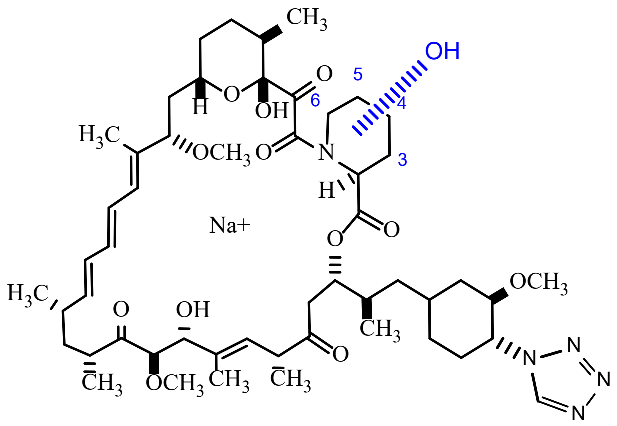

3/4/5/6-Hydroxy Piperidine Zotarolimus (III), m/z = 1004.55666
Extracted Ion Chromatogram (EIC)

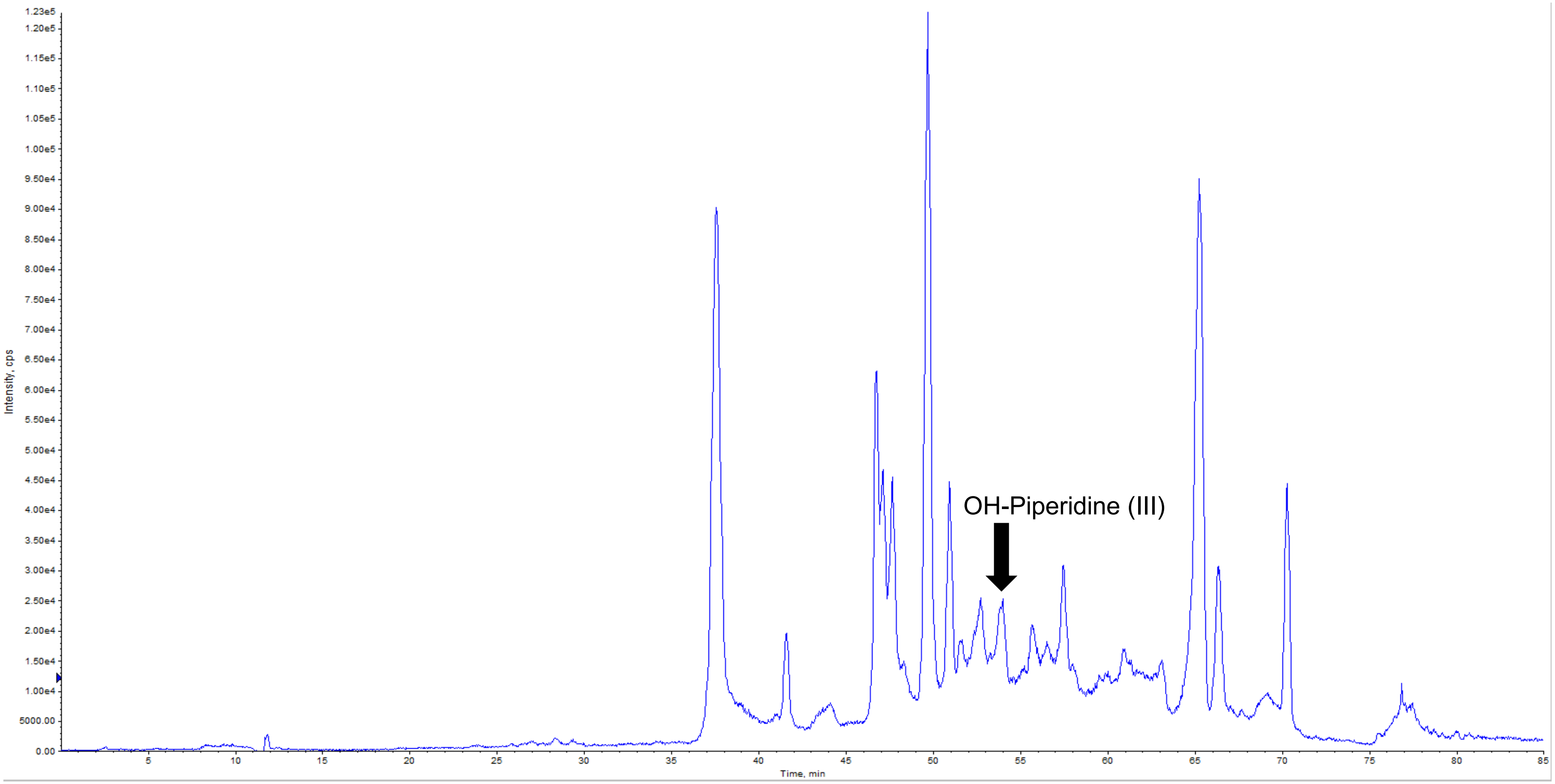

# Mass Spectrum of 3/4/5/6-Hydroxy Piperidine Zotarolimus (III)
CE = 75 eV, DP = 125 V

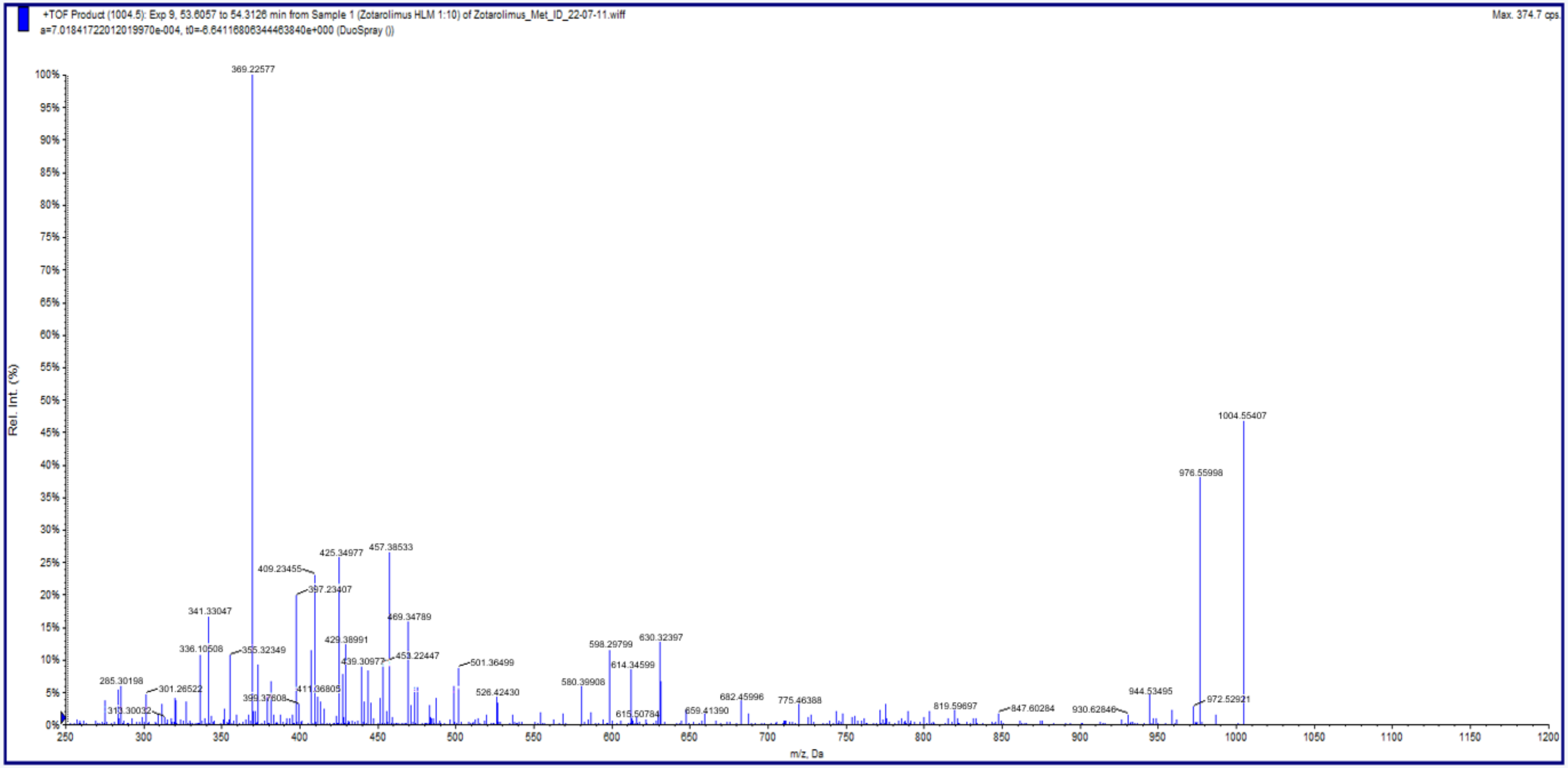

Δppm of Hydroxy-Piperidine (III) Zotarolimus Fragments

Fragment No.	theoretical mass	measured mass	Δppm
1	336.10537	336.10508	0.9
2	369.22610	369.22577	0.9
3	381.24002	381.23907	2.5
4	397.23493	397.23407	2.2
5	409.23493	409.23455	0.9
6	441.26115	441.26089	0.6
7	453.22476	453.22447	0.6
8	598.29865	598.29799	1.1
9	630.32487	630.32397	1.4
10	659.41429	659.41390	0.6
11	710.33716	710.33469	3.5
12	783.46672	783.46598	0.9
13	815.49294	815.49193	1.2
14	1004.55666	1004.55407	2.6

3/4/5/6-Hydroxy Piperidine Zotarolimus, m/z = 1004.55666

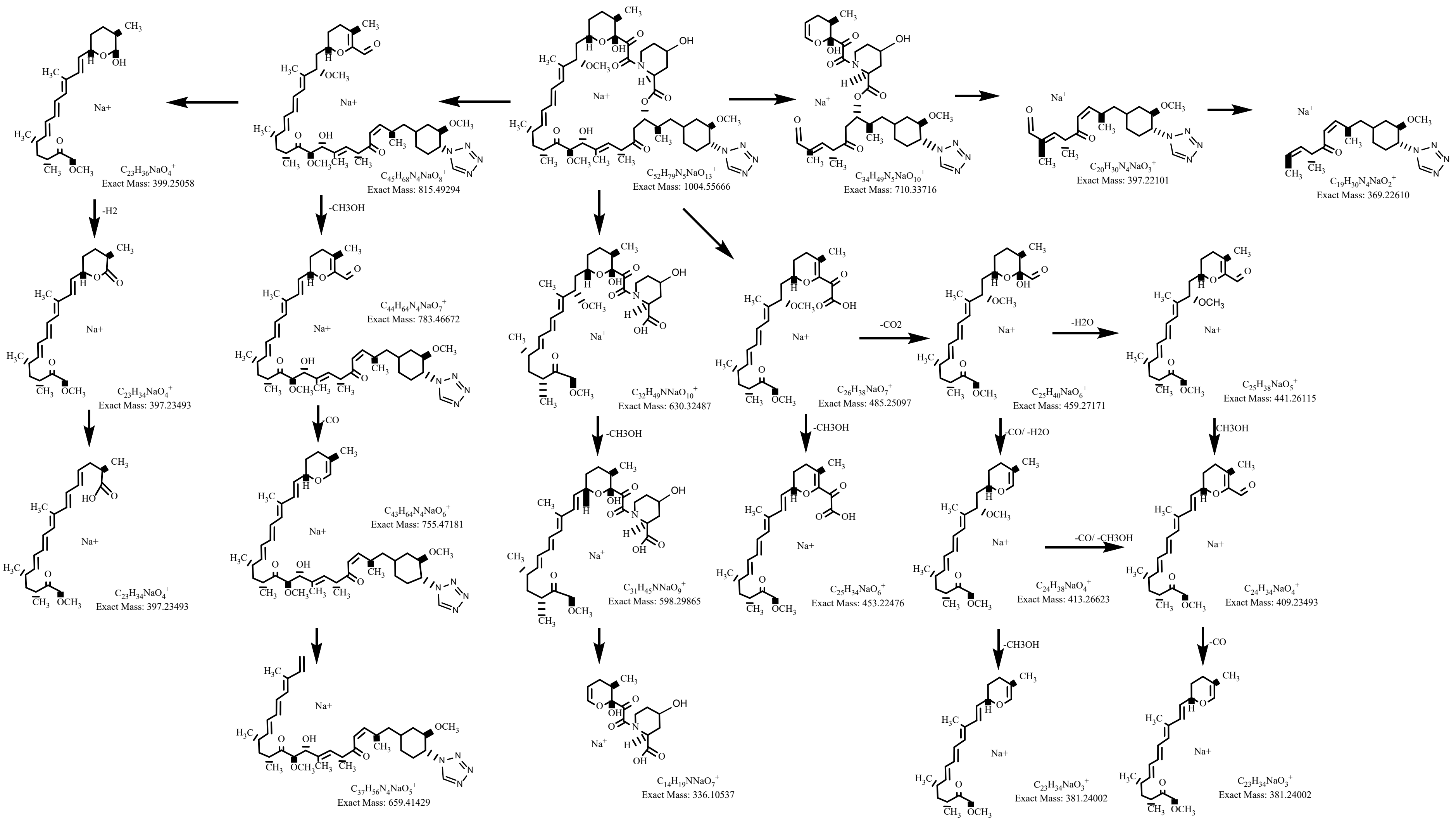

Structural Confirmation of Hydroxy Piperidine (III) Zotarolimus, m/z = 1004.55666

Zotarolimus Fragments	Pip-OH (III) Zotarolimus Fragments	Comments	
320.11046	336.10508	+16) Hydroxylation	11-, 12-, 14-OH, or OH-piperidine present
369.22610	369.22577	no hydroxylation	Rules out 47, 48, and 49-OH
381.24002	381.23907	no hydroxylation	Rules out 11, 12, 14, 23, 24, 25, 45, or 46-OH
397.23493	397.23407	no hydroxylation	Rules out 11, 12, 14, 23, 24, 25, 45, or 46-OH
409.23493	409.23455	no hydroxylation	Rules out 11, 12, 14, 23, 24, 25, 45, or 46-OH
441.26115	441.26089	no hydroxylation	Rules out 11, 12, 14, 23, 24, 25, 45, or 46-OH
453.22476	453.22447	no hydroxylation	Rules out 11, 12, 14, 23, 24, 25, 45, or 46-OH
582.30374	598.29799	+16) Hydroxylation	11, 12, 14, 23, 24, 25, 45, 46-OH, or piperidine-OH present
614.32995	630.32397	+16) Hydroxylation	11, 12, 14, 23, 24, 25, 45, 46-OH, or piperidine-OH present
659.41429	659.41390	no hydroxylation	Rules out 23, 24, 25, 45, 46, 47, 48, or 49-OH
694.34225	710.33469	(+16) Hydroxylation	11, 12, 14, 47, 48, 49, or Pip-OH present
783.46672	783.46598	no hydroxylation	Rules out 11, 12, 14, 23, 24, 25, 45, 46, 47, 48, or 49-OH
815.49294	815.49193	no hydroxylation	Rules out 11, 12, 14, 23, 24, 25, 45, 46, 47, 48, or 49-OH
988.56174	1004.55407	+16) Hydroxylation	
Characteristic Fragments			336
Determinant patterns			

Hydroxy-Piperidine Zotarolimus (IV), m/z =1004.55666

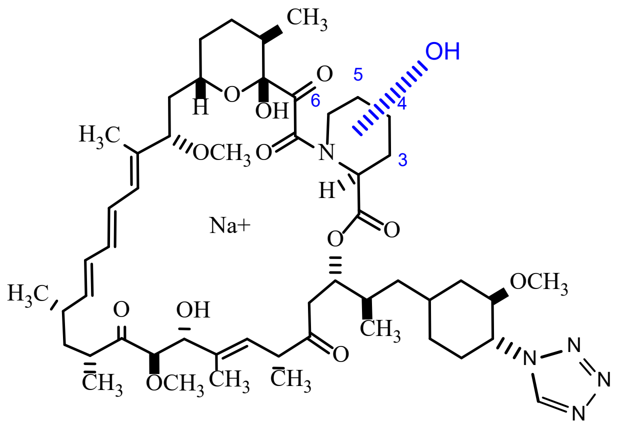

3/4/5/6-Hydroxy Piperidine Zotarolimus (IV), m/z = 1004.55666
Extracted Ion Chromatogram (EIC)

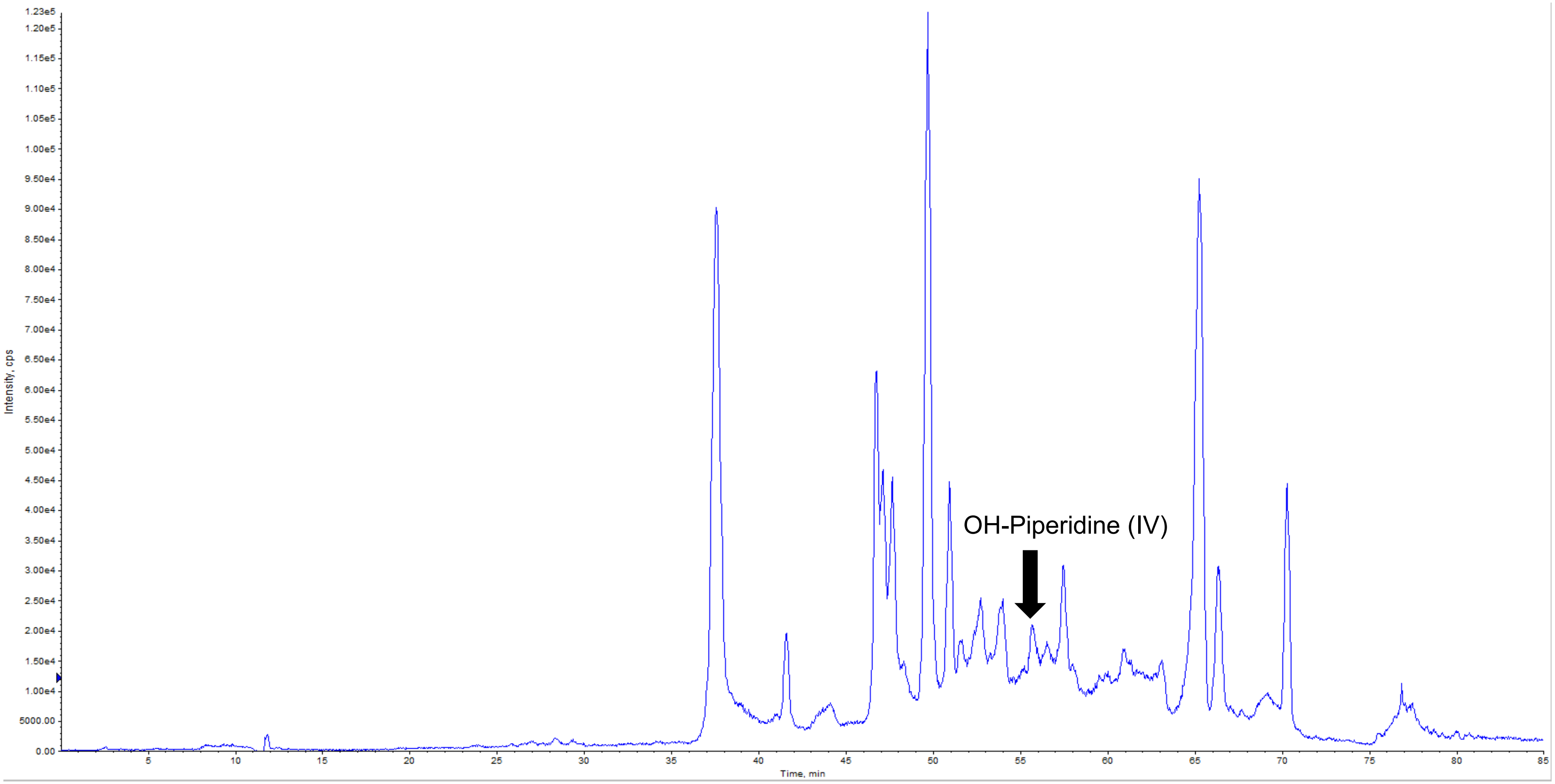

Mass Spectrum of 3/4/5/6-Hydroxy Piperidine Zotarolimus (IV)
CE = 75 eV, DP = 125 V

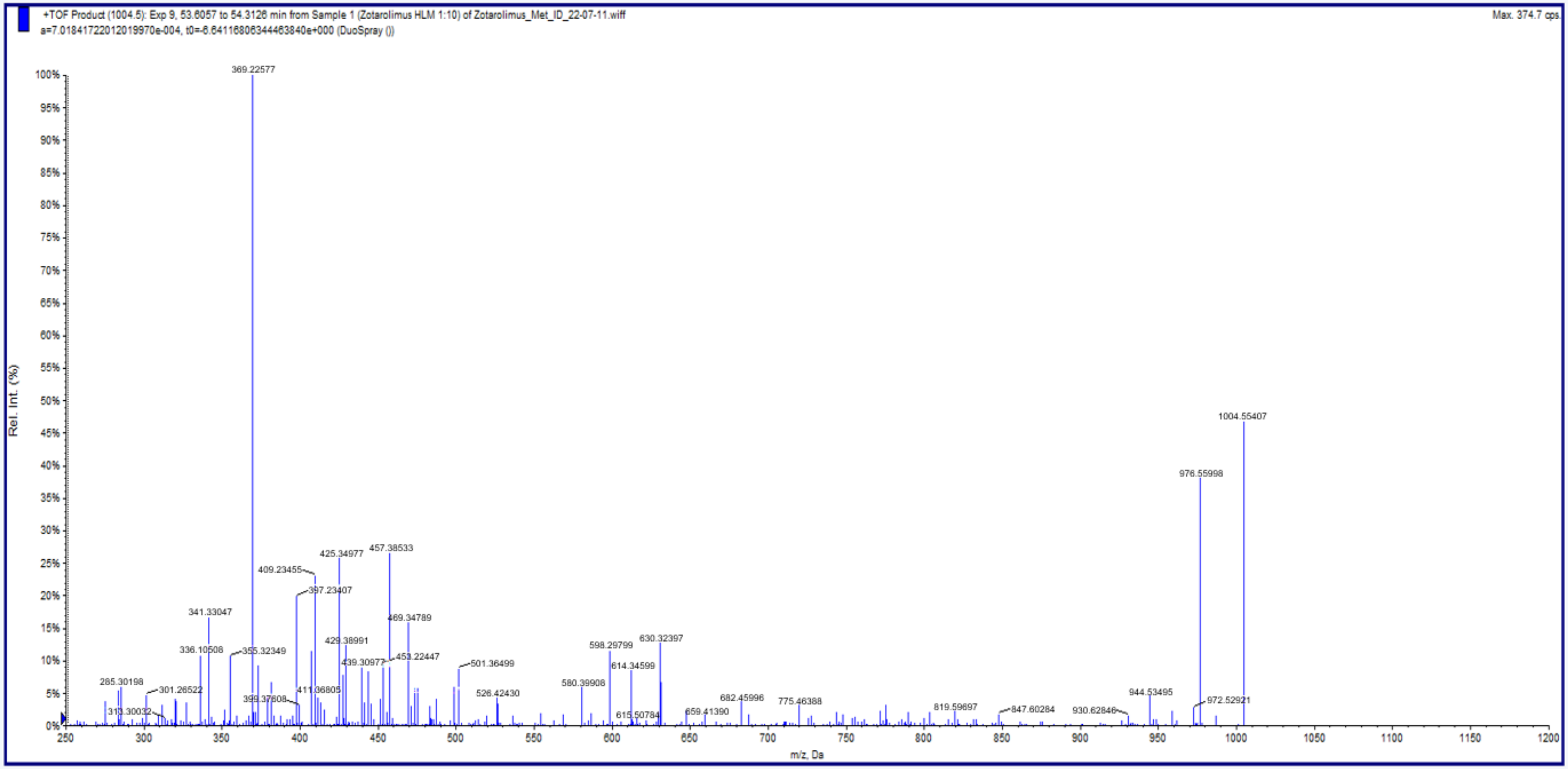

Δppm of Hydroxy-Piperidine (IV) Zotarolimus Fragments

Fragment No.	theoretical mass	measured mass	Δppm
1	336.10537	336.10499	1.1
2	369.22610	369.22599	0.3
3	381.24002	381.23990	0.3
4	397.23493	397.23463	0.8
5	409.23493	409.23487	0.1
6	441.26115	441.26068	1.1
7	453.22476	453.22403	1.6
8	598.29865	598.29809	0.9
9	630.32487	630.32470	0.3
10	659.41429	659.41400	0.4
11	710.33716	710.33670	0.6
12	783.46672	783.46603	0.9
13	815.49294	815.49218	0.9
14	1004.55666	1004.55599	0.7

3/4/5/6-Hydroxy Piperidine Zotarolimus, m/z = 1004.55666

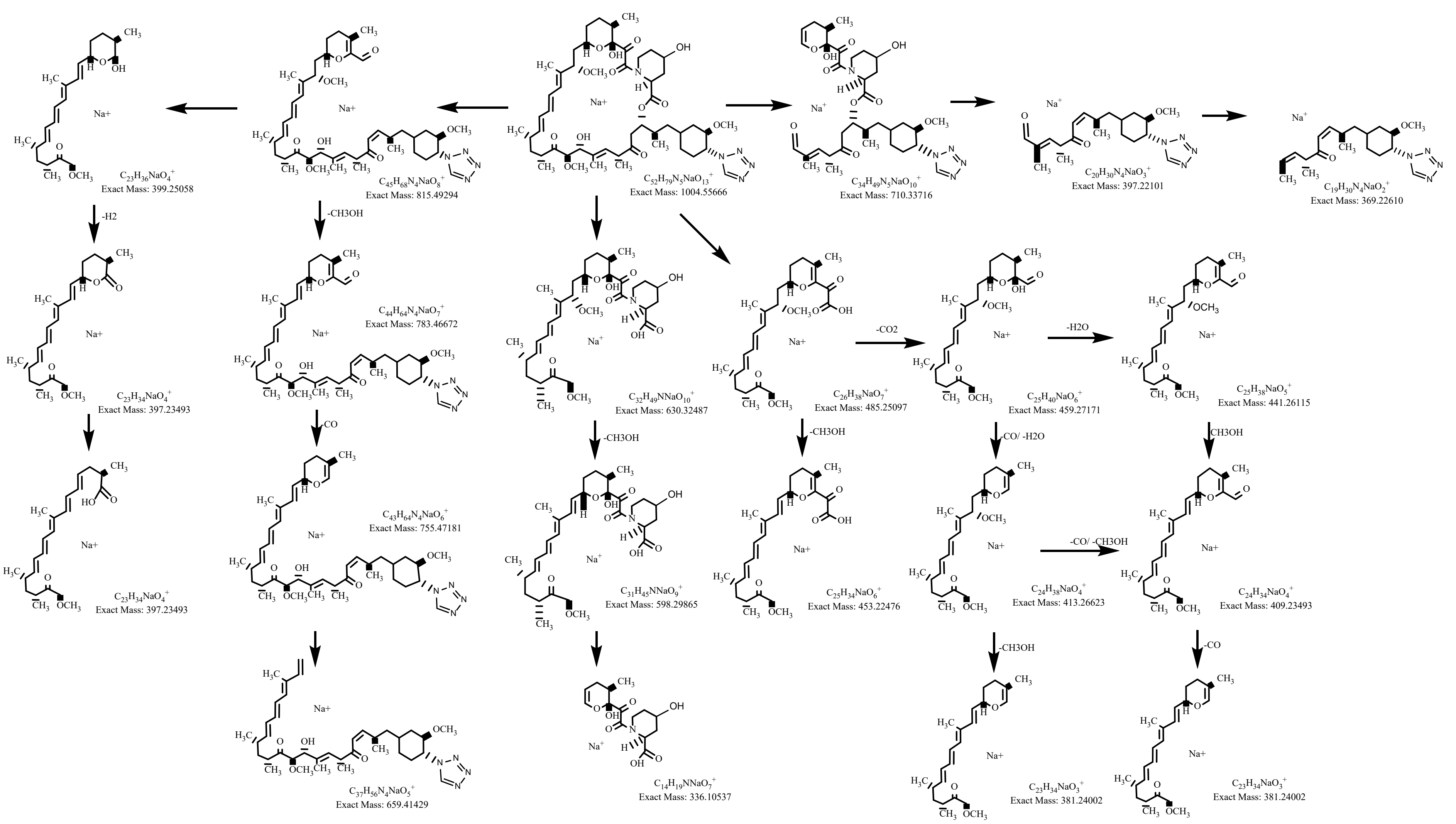

Structural Confirmation of Hydroxy Piperidine (IV) Zotarolimus, m/z = 1004.55666

Zotarolimus Fragments	Pip-OH (IV) Zotarolimus Fragments	Comments	
369.22610	336.10499	+16) Hydroxylation	11-, 12-, 14-OH, or OH-piperidine present
381.24002	369.22599	no hydroxylation	Rules out 47, 48, and 49-OH
397.23493	381.23990	no hydroxylation	Rules out 11, 12, 14, 23, 24, 25, 45, or 46-OH
409.23493	397.23463	no hydroxylation	Rules out 11, 12, 14, 23, 24, 25, 45, or 46-OH
441.26115	409.23487	no hydroxylation	Rules out 11, 12, 14, 23, 24, 25, 45, or 46-OH
453.22476	441.26068	no hydroxylation	Rules out 11, 12, 14, 23, 24, 25, 45, or 46-OH
582.30374	453.22403	no hydroxylation	Rules out 11, 12, 14, 23, 24, 25, 45, or 46-OH
614.32995	598.29809	+16) Hydroxylation	11, 12, 14, 23, 24, 25, 45, 46-OH, or piperidine-OH present
659.41429	630.32470	+16) Hydroxylation	11, 12, 14, 23, 24, 25, 45, 46-OH, or piperidine-OH present
694.34225	659.41400	no hydroxylation	Rules out 23, 24, 25, 45, 46, 47, 48, or 49-OH
783.46672	710.33670	(+16) Hydroxylation	11, 12, 14, 47, 48, 49, or Pip-OH present
815.49294	783.46603	no hydroxylation	Rules out 11, 12, 14, 23, 24, 25, 45, 46, 47, 48, or 49-OH
988.56174	815.49218	no hydroxylation	Rules out 11, 12, 14, 23, 24, 25, 45, 46, 47, 48, or 49-OH
Characteristic Fragments			336.10499
Determinant patterns			

23-Hydroxy Zotarolimus, m/z = 1004.55666

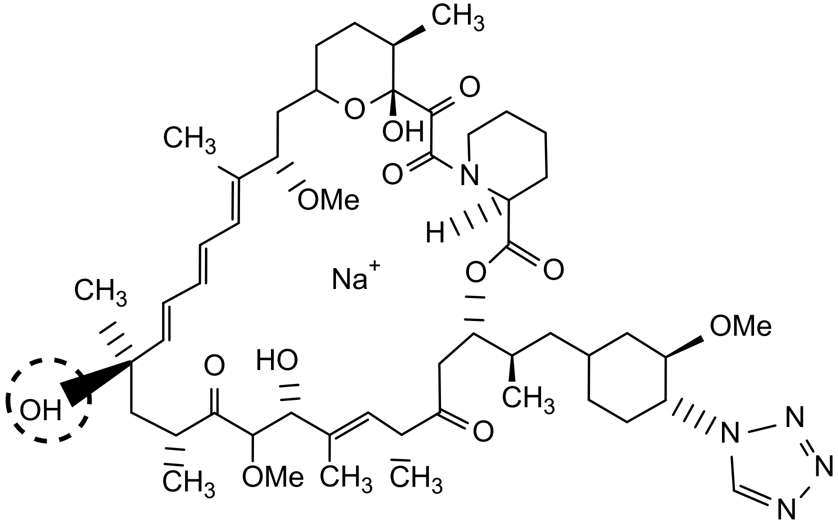

Chemical Formula: $C_{52}H_{79}N_5NaO_{13}^{+}$
Exact Mass: 1004.55666

23-hydroxy zotarolimus

23-Hydroxy Zotarolimus, m/z = 1004.55666
Extracted Ion Chromatogram (EIC)

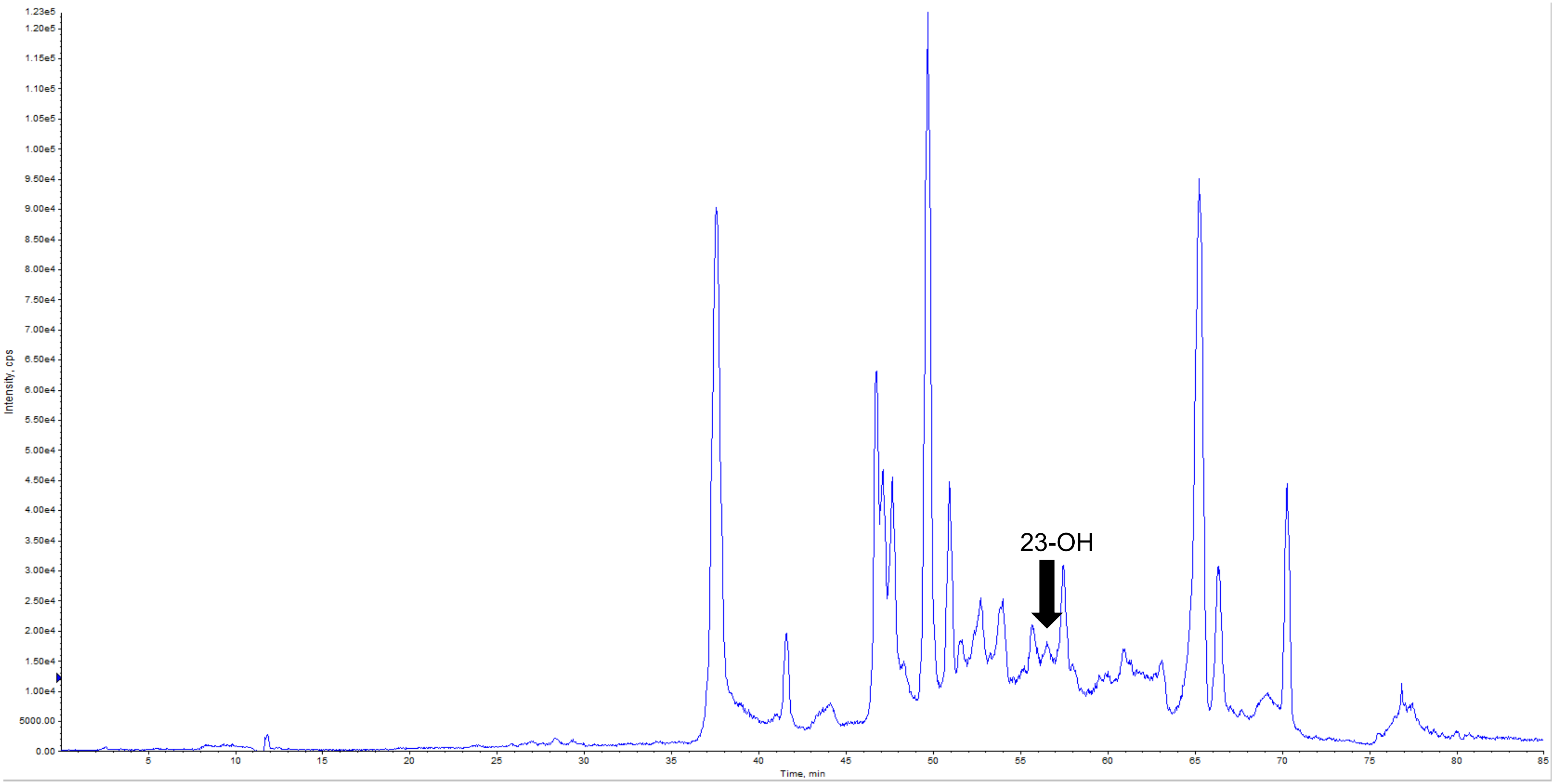

Mass Spectrum of 23-Hydroxy Zotarolimus

CE = 75 eV, DP = 125 V

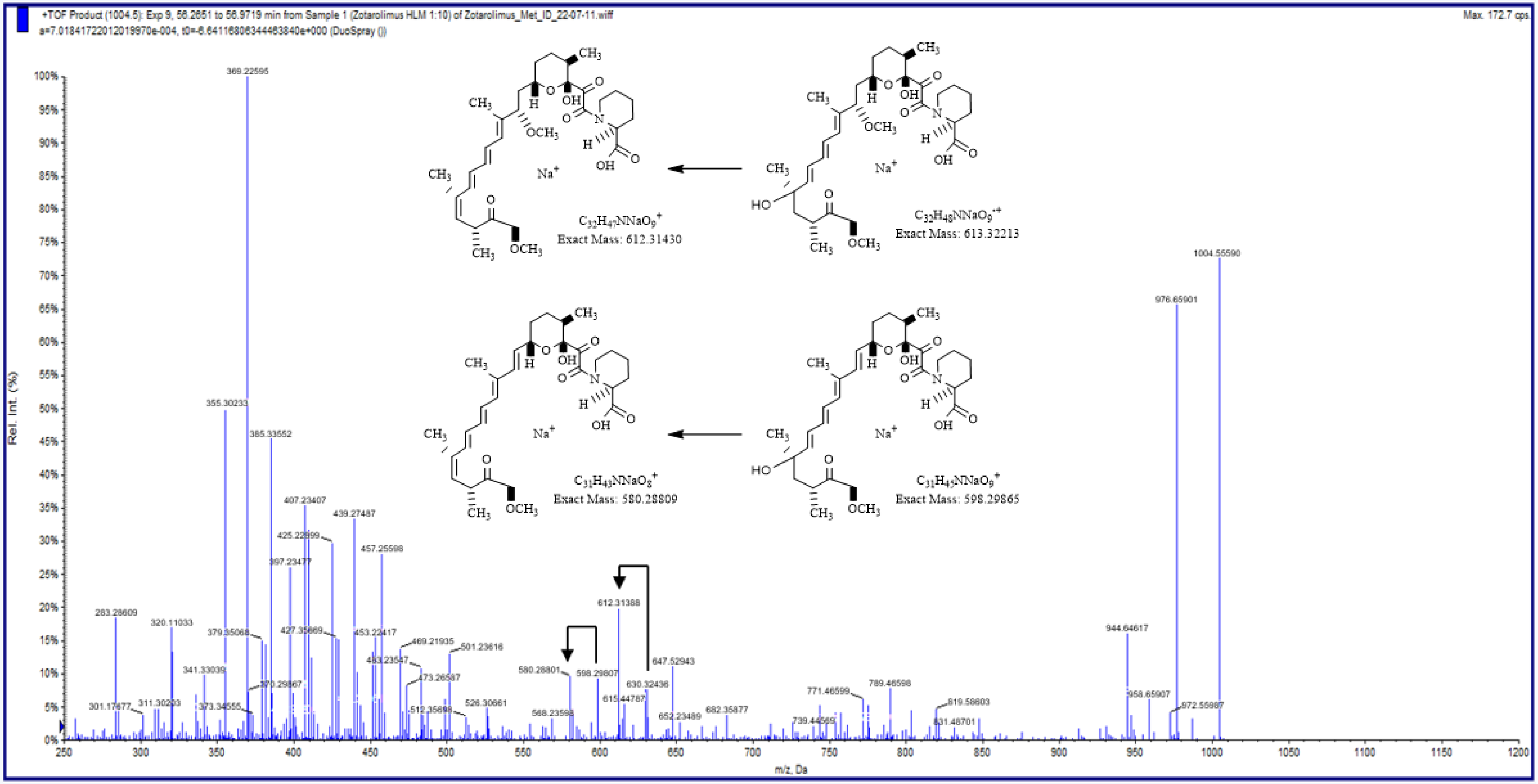

Δppm of 23-Hydroxy Zotarolimus Fragments

Fragment No.	theoretical mass	measured mass	Δppm
1	320.11046	320.11033	0.4
2	369.22610	369.22595	0.4
3	397.23493	397.23477	0.4
4	413.22985	413.22937	1.2
5	425.22985	425.22899	2.0
6	441.26115	441.26103	0.3
7	457.25606	457.25598	0.2
8	469.21967	469.21935	0.7
9	598.29865	598.29807	1.0
10	630.32487	630.32436	0.8
11	675.40921	675.40899	0.3
12	694.34225	ND	ND
13	771.46672	771.46599	0.9
14	831.48785	831.48701	1.0
15	1004.55666	1004.5559	0.8

23-Hydroxy Zotarolimus, m/z = 1004.55666

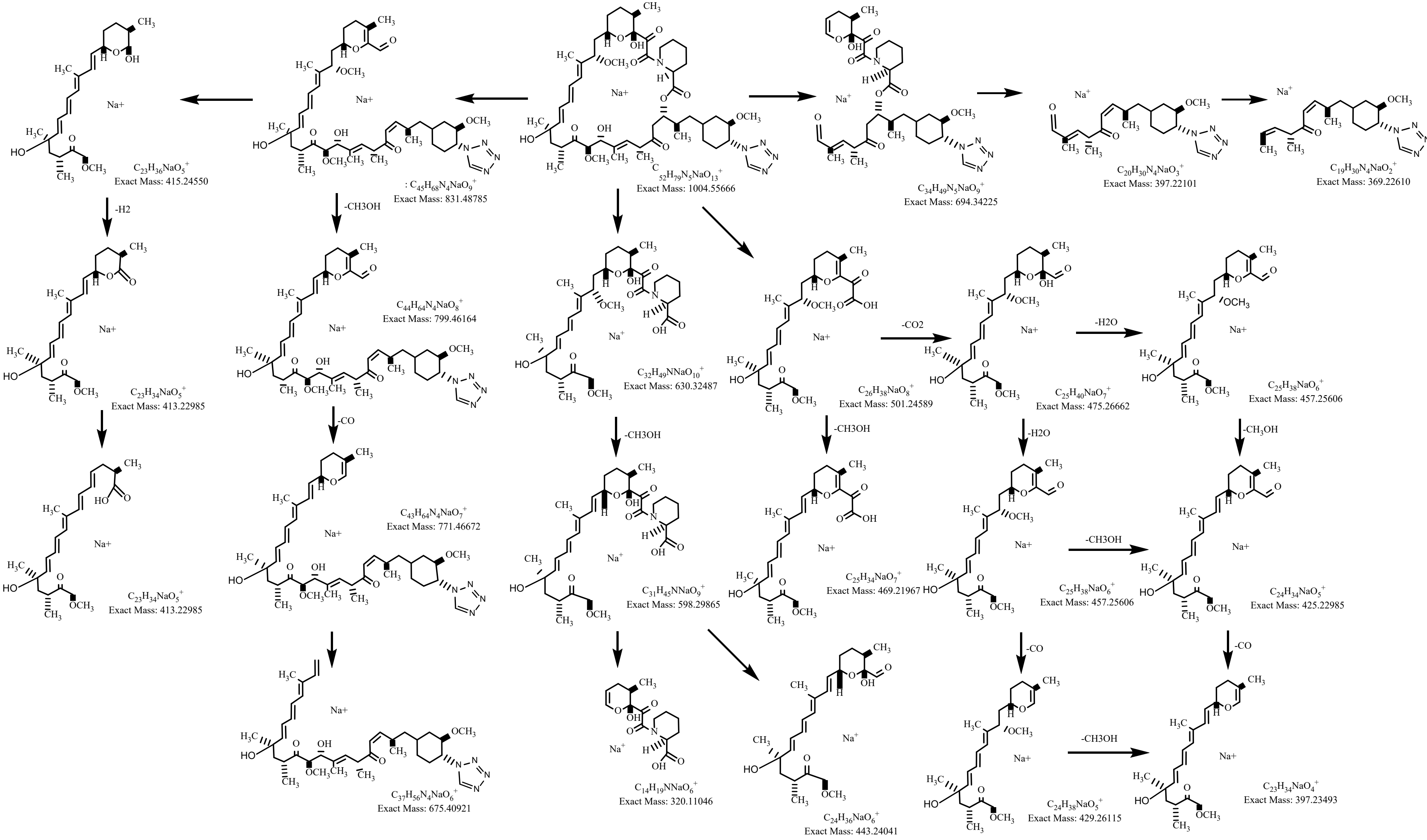

Structural Confirmation of 23-Hydroxy Zotarolimus, m/z = 1004.55666

Zotarolimus Fragments	23-Hydroxy Zotarolimus Fragments	Comments	
320.11046	320.11033	no hydroxylation	Rules out OH-piperidine, 11 and 12, and 14-OH
369.22610	369.22595	no hydroxylation	Rules out 47, 48, and 49-OH
381.24002	397.23477	(+16) Hyroxylation	11, 12, 14, 23, 24, 25, 45, or 46-OH present
397.23493	397.23477	no hydroxylation	Rules out 47, 48, and 49-OH
409.23493	425.22899	(+16) Hyroxylation	11, 12, 14, 23, 24, 25, 45, or 46-OH present
427.24550	443.24041	(+16) Hyroxylation	11, 12, 14, 23, 24, 25, 45, or 46-OH present
441.26115	457.25598	(+16) Hyroxylation	11, 12, 14, 23, 24, 25, 45, or 46-OH present
453.22476	469.21935	(+16) Hyroxylation	11, 12, 14, 23, 24, 25, 45, or 46-OH present
582.30374	598.29807	(+16) Hyroxylation	11, 12, 14, 23, 24, 25, 45, 46-OH, or piperidine-OH present
614.32995	630.32436	(+16) Hyroxylation	11, 12, 14, 23, 24, 25, 45, 46-OH, or piperidine-OH present
659.41429	675.40899	(+16) Hyroxylation	Rules out OH-piperidine, 11, 12 and 14-OH
694.34225	ND	no hydroxylation	Rules out OH-piperidine, 11, 12, 14, 47, 48, and 49-OH
783.46672	799.46035	(+16) Hyroxylation	11, 12, 14, 23, 24, 25, 45, 46, 47, 48, or 49-OH present
815.49294	831.48701	(+16) Hyroxylation	11, 12, 14, 23, 24, 25, 45, 46, 47, 48, or 49-OH present
988.56174	1004.5559	(+16) Hyroxylation	
Characteristic Fragments	significant 598		Characteristic fragment for water loss (630 -18 = 598 m/z) of 23 and 24-OH<br>Characteristic fragment for water loss (612-18 = 594 m/z) of 23 and 24-OH
Determinant patterns	Water loss		23-OH metabolite elutes at same relative retention time as 23-OH identified from sirolimus and temsirolimus

49-Hydroxy Zotarolimus, m/z = 1004.55666

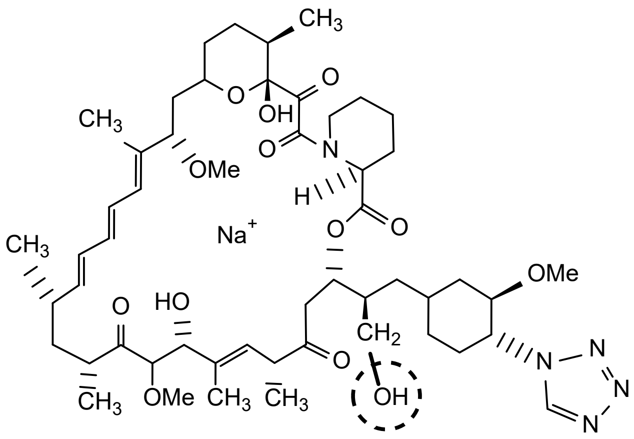

Chemical Formula: $C_{52}H_{79}N_5NaO_{13}^+$
Exact Mass: 1004.55666

49-hydroxy zotarolimus

49-Hydroxy Zotarolimus, m/z = 1004.55666
Extracted Ion Chromatogram (EIC)

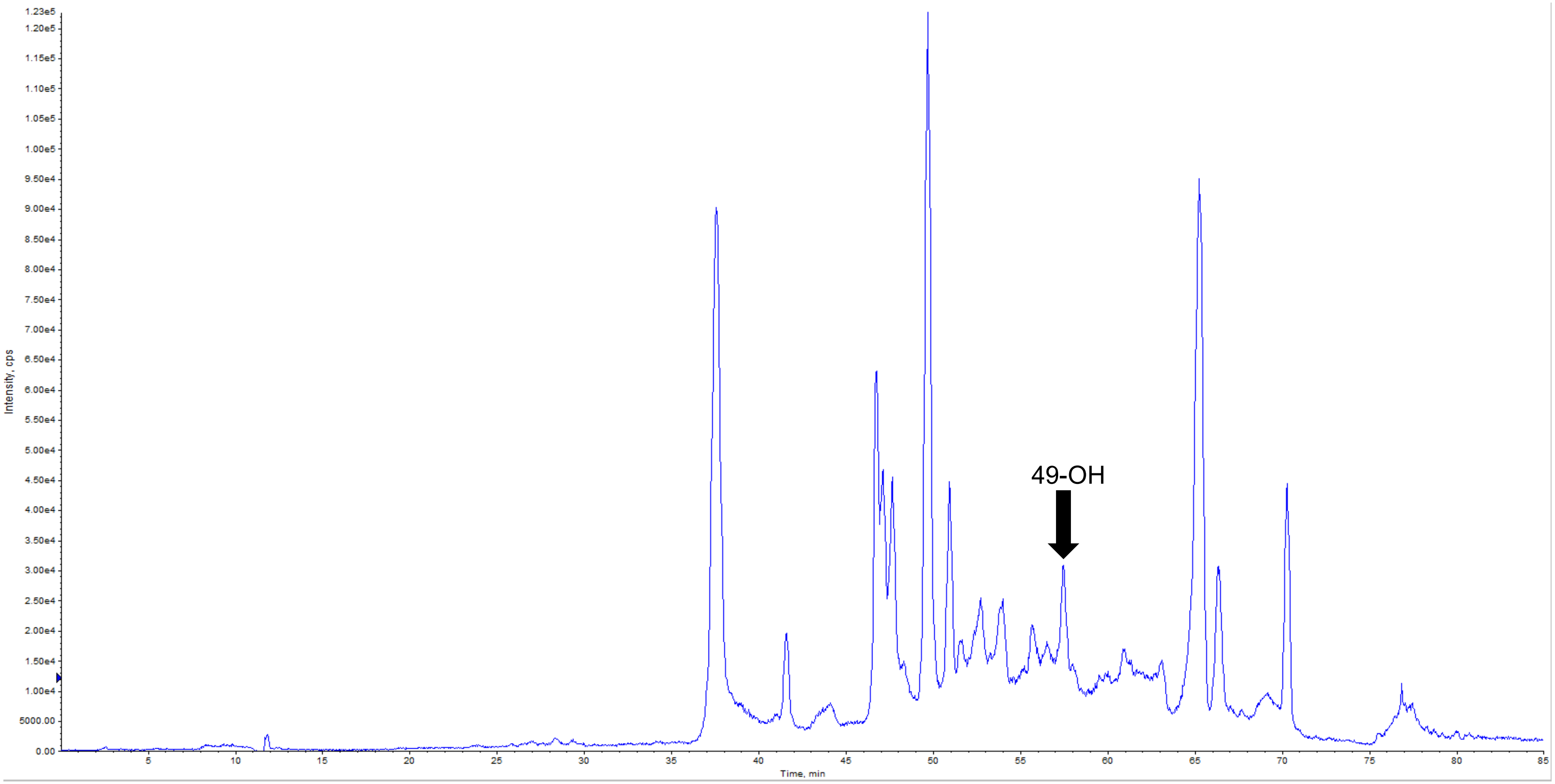

Mass Spectrum of 49-Hydroxy Zotarolimus

CE = 75 eV, DP = 125 V

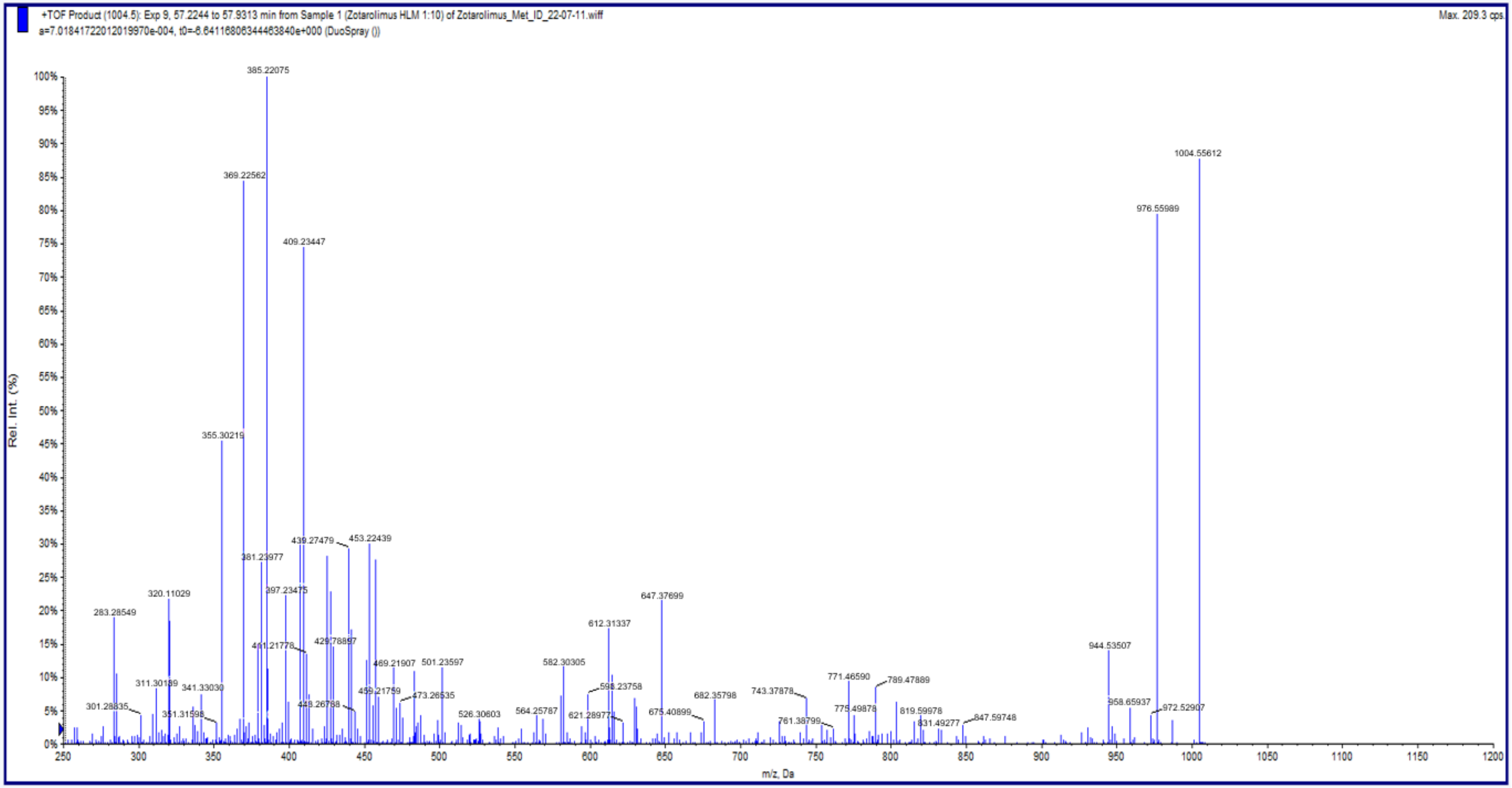

∆ppm of 49-Hydroxy Zotarolimus Fragments

Fragment No.	theoretical mass	measured mass	∆ppm
1	320.11046	320.11029	0.5
2	381.24002	381.23977	0.7
3	397.23493	397.23475	0.5
4	409.23493	409.23447	1.1
5	441.26115	441.26073	1.0
6	453.22476	453.22439	0.8
7	582.30374	582.30305	1.2
8	614.32995	614.32937	0.9
9	647.37791	647.37699	1.4
10	675.40921	675.40899	0.3
11	771.46672	771.4659	1.1
12	831.48785	831.48727	0.7
13	1004.55666	1004.55612	0.5

	theoretical mass	measured mass	∆ppm
Characteristic Fragment	385.22101	385.22075	0.7
	413.21593	413.21579	0.3

49-Hydroxy Zotarolimus, m/z = 1004.55666

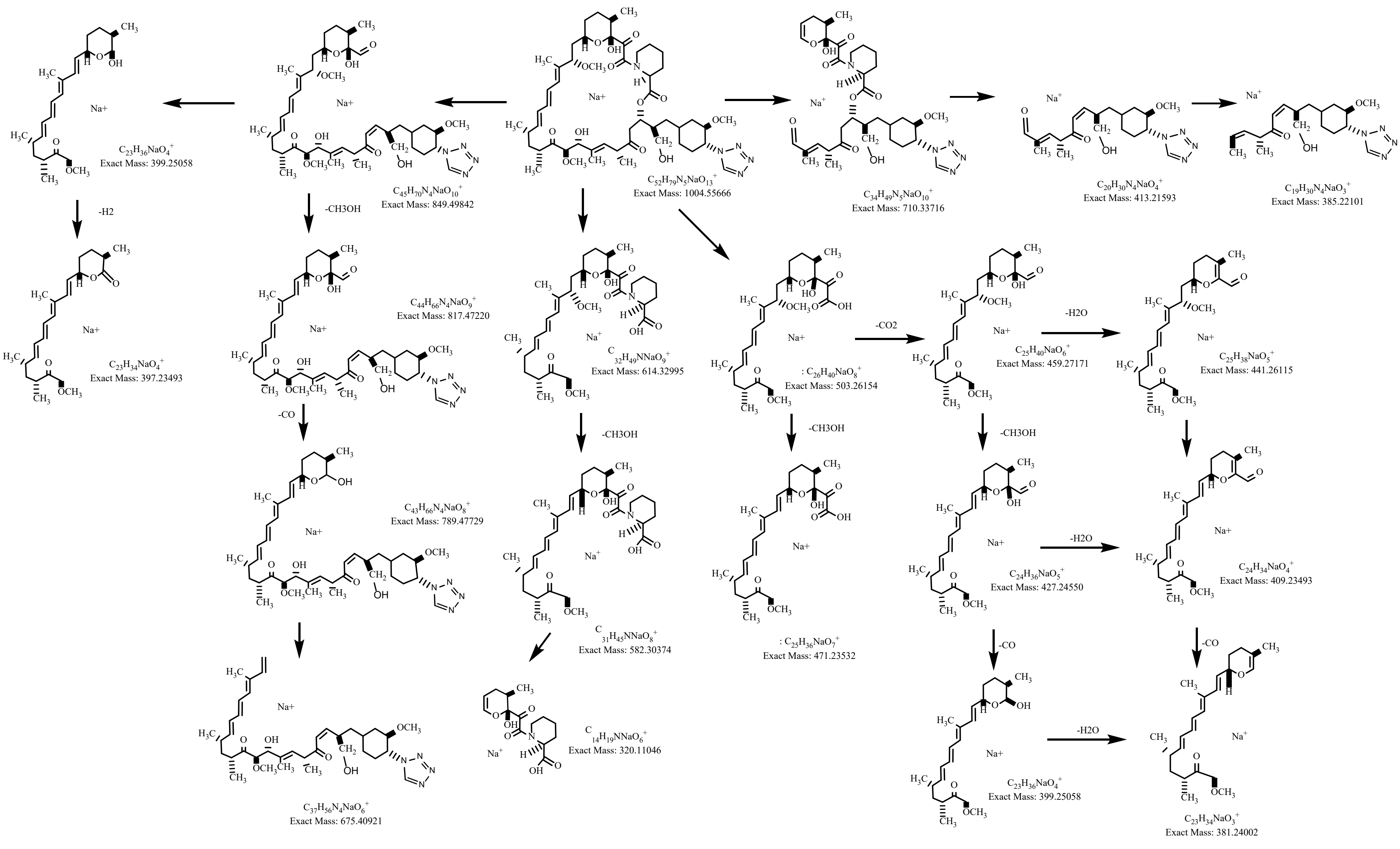

Characteristic Fragments of 49-Hydroxy Zotarolimus, m/z = 1004.55666

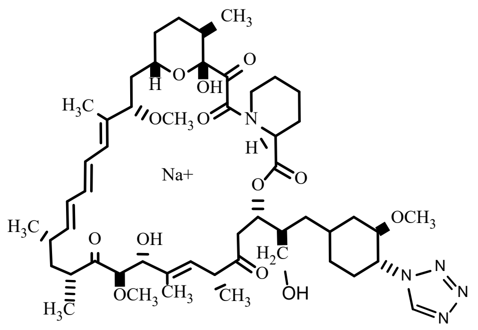

$C_{52}H_{79}N_5NaO_{13}^+$
Exact Mass: 1004.55666

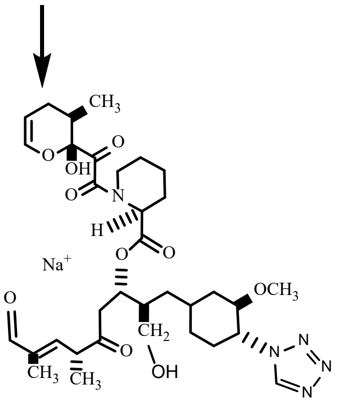

$C_{34}H_{49}N_5NaO_{10}^+$
Exact Mass: 710.33716

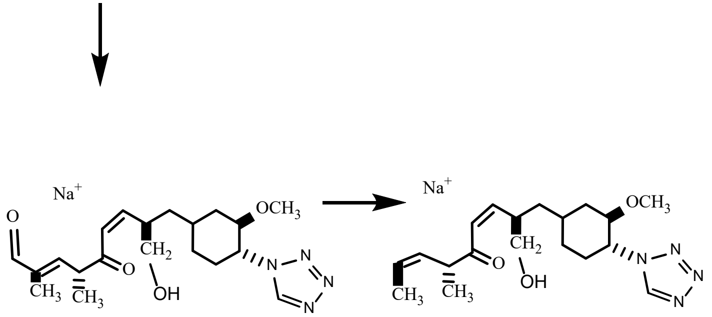

$C_{20}H_{30}N_4NaO_4^+$
Exact Mass: 413.21593

$C_{19}H_{30}N_4NaO_3^+$
Exact Mass: 385.22101

Structural Confirmation of 49-Hydroxy Zotarolimus, m/z = 1004.55666

Zotarolimus Fragments	49-Hydroxy Zotarolimus Fragments	Comments	
320.11046	320.11046	no hydroxylation	Rules out OH-piperidine, 11, 12, and 14-OH
369.22610	385.22070	(+16) Hydroxylation	confirms 48, or 49-OH
381.2396	381.23977	no hydroxylation	Rules out OH-piperidine, 11, 12, 14, 23, 24, 25, 45, and 46-OH
397.23493	397.23475	no hydroxylation	Rules out 11, 12, 14, 23, 24, 25, 45, and 46-OH
409.23493	409.23447	no hydroxylation	Rules out 11, 12, 14, 23, 24, 25, 45, and 46-OH
427.24550	427.24489	no hydroxylation	Rules out 11, 12, 14, 23, 24, 25, 45, and 46-OH
441.26115	441.26073	no hydroxylation	Rules out 11, 12, 14, 23, 24, 25, 45, and 46-OH
453.22476	453.22439	no hydroxylation	Rules out 11, 12, 14, 23, 24, 25, 45, and 46-OH
582.30374	582.30305	no hydroxylation	Rules out 11, 12, 14, 23, 24, 25, 45, and 46-OH piperidine-OH present
614.32995	614.32937	no hydroxylation	11, 12, 14, 23, 24, 25, 45, 46-OH, and piperidine-OH
659.41429	675.40899	(+16) Hydroxylation	23, 24, 25, 45, 46, 47, 48, or 49-OH present
694.34225	710.33689 (very low)	(+16) Hydroxylation	Rules out 23, 24, 25, 45, and 46-OH
783.46672	799.46672 (low)	(+16) Hydroxylation	Rules out OH-piperidine
815.49294	831.49277	(+16) Hydroxylation	Rules out OH-piperidine
988.56174	1004.55612	(+16) Hydroxylation	
Characteristic Fragments			413 and 385
Determinant patterns			No methanol loss possible

Desmethyl Zotarolimus Metabolites, (m/z = 974.54609)

Zotarolimus Demethylated Metabolites

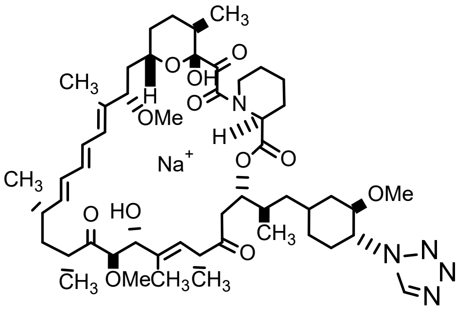

$C_{52}H_{79}N_5NaO_{12}^{+}$

Exact Mass: 988.56174

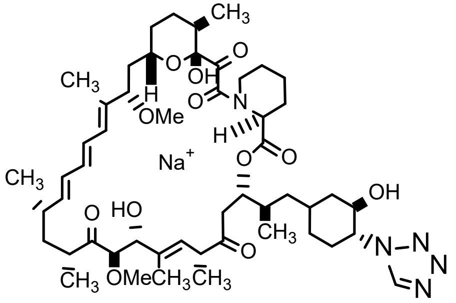

39-O-desmethyl zotarolimus

$C_{51}H_{77}N_5NaO_{12}^{+}$

Exact Mass: 974.54609

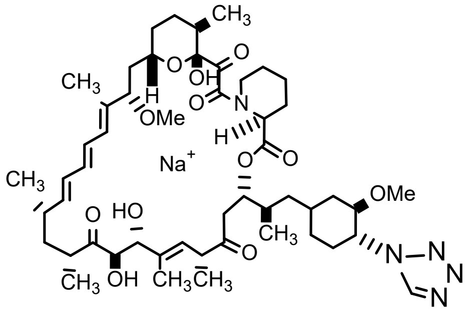

27-O-desmethyl zotarolimus

$C_{51}H_{77}N_5NaO_{12}^{+}$

Exact Mass: 974.54609

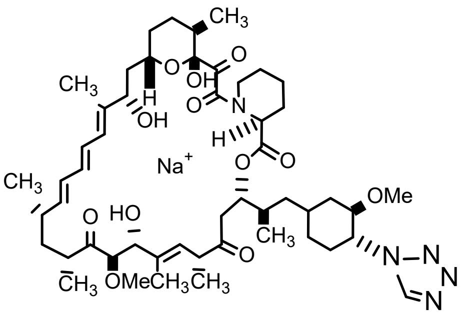

16-O-desmethyl zotarolimus

$C_{51}H_{77}N_5NaO_{12}^{+}$

Exact Mass: 974.54609

Desmethyl Zotarolimus Metabolites
Extracted Ion Chromatogram (EIC), m/z = 974.54609

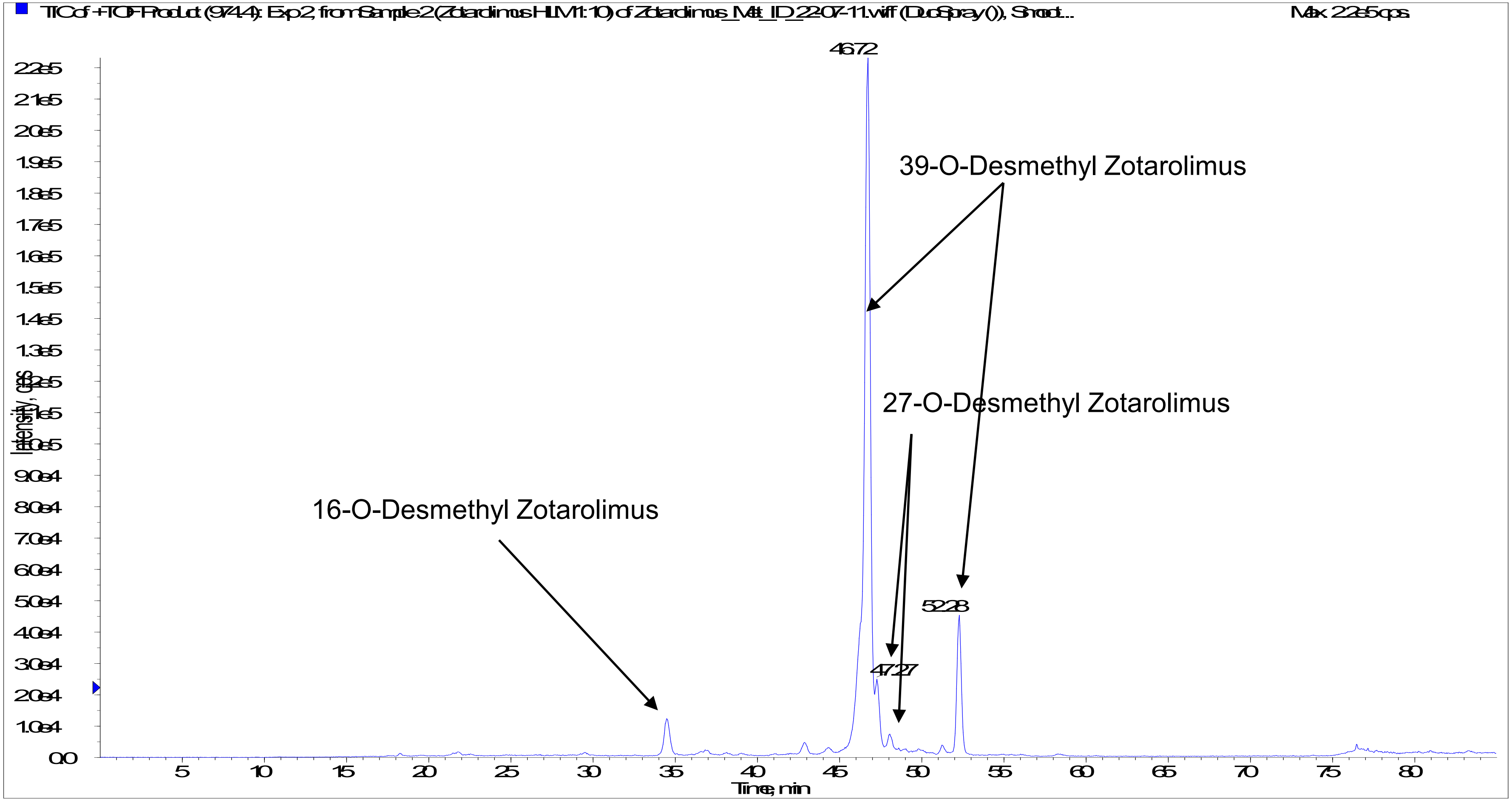

16-O-Desmethyl Zotarolimus (m/z = 974.54609)

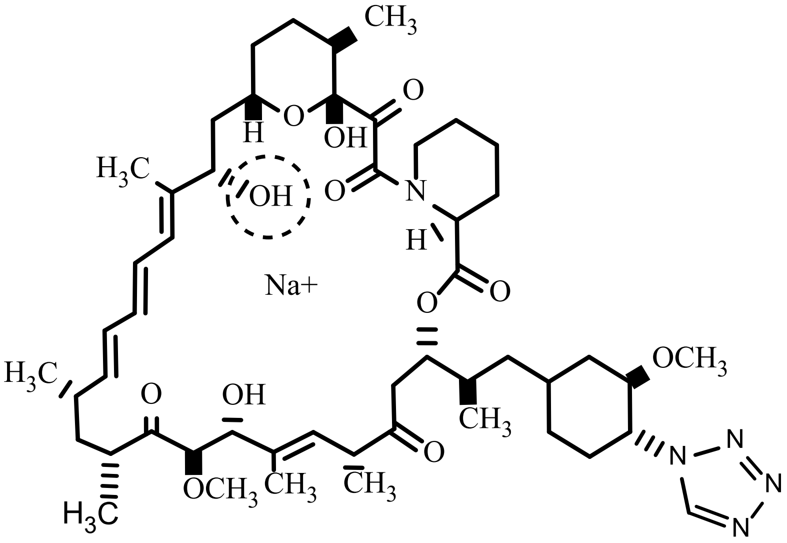

$C_{51}H_{77}N_5NaO_{12}^+$
Exact Mass: 974.54609

16-O-desmethyl zotarolimus

16-O-Desmethyl Zotarolimus Metabolite
Extracted Ion Chromatogram (EIC), m/z = 974.54609

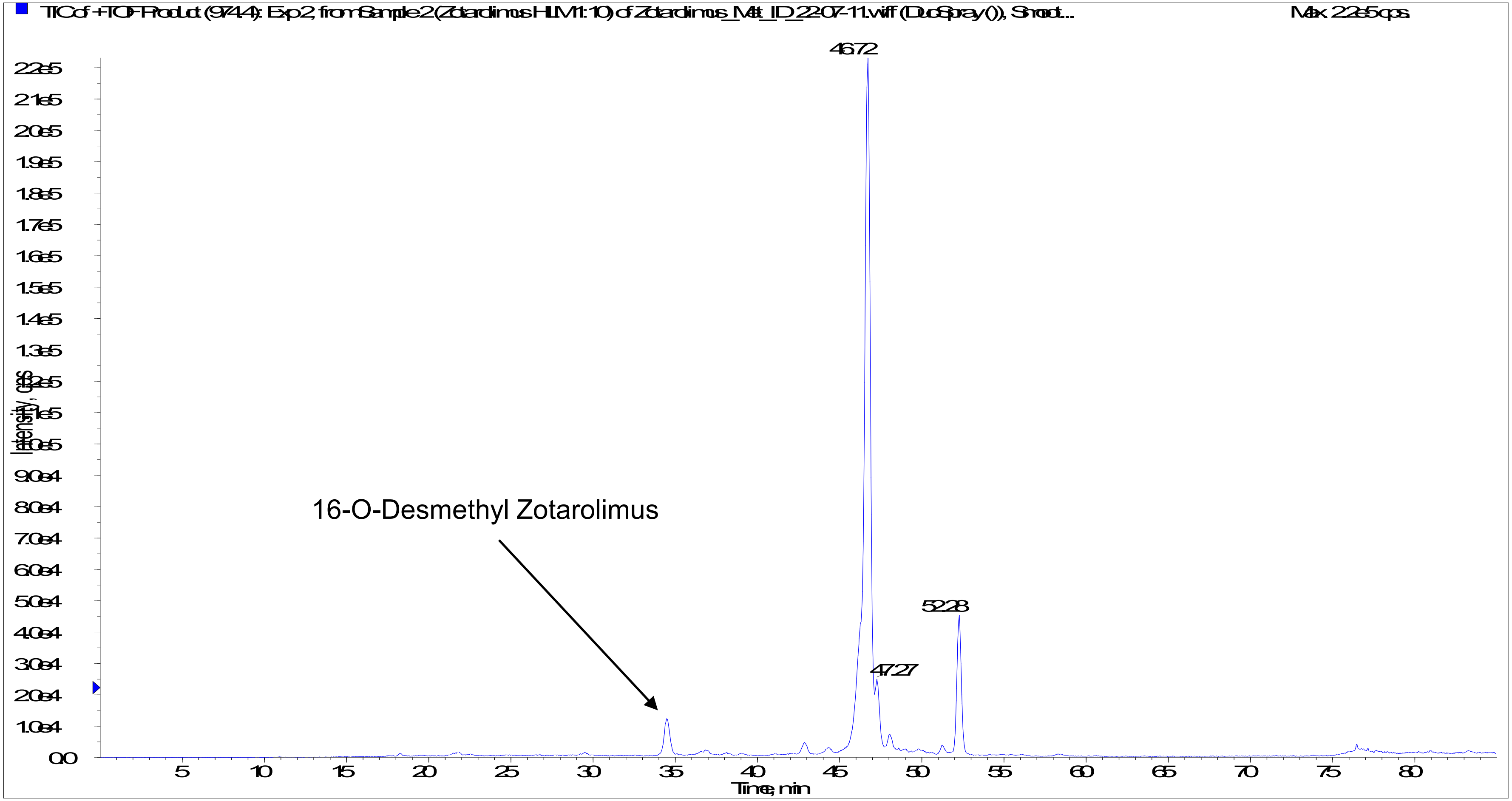

Mass Spectrum of 16-O-desmethyl Zotarolimus

CE = 75 eV, DP = 125 V

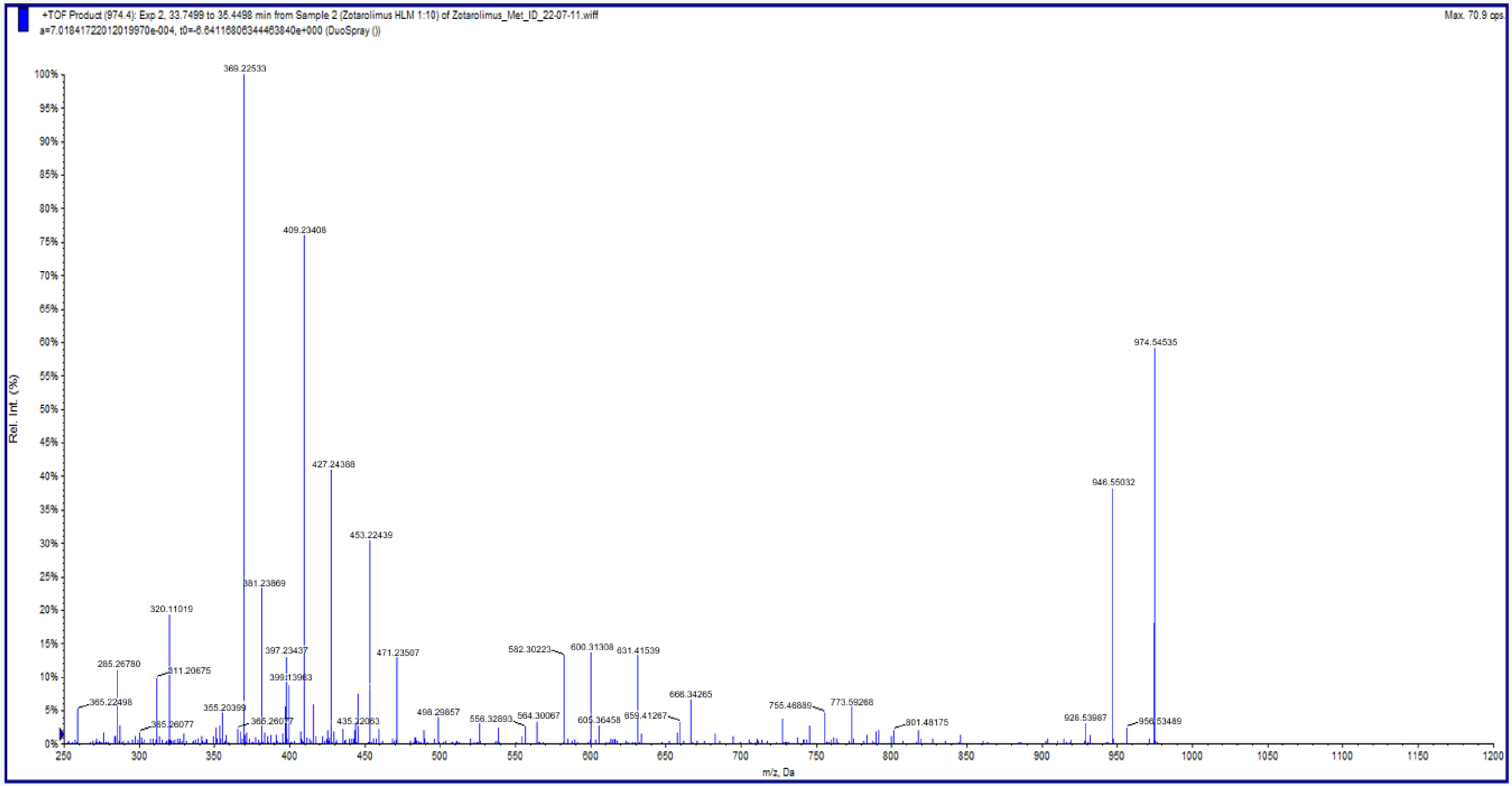

16-O-Desmethyl Zotarolimus Fragmentation Pattern

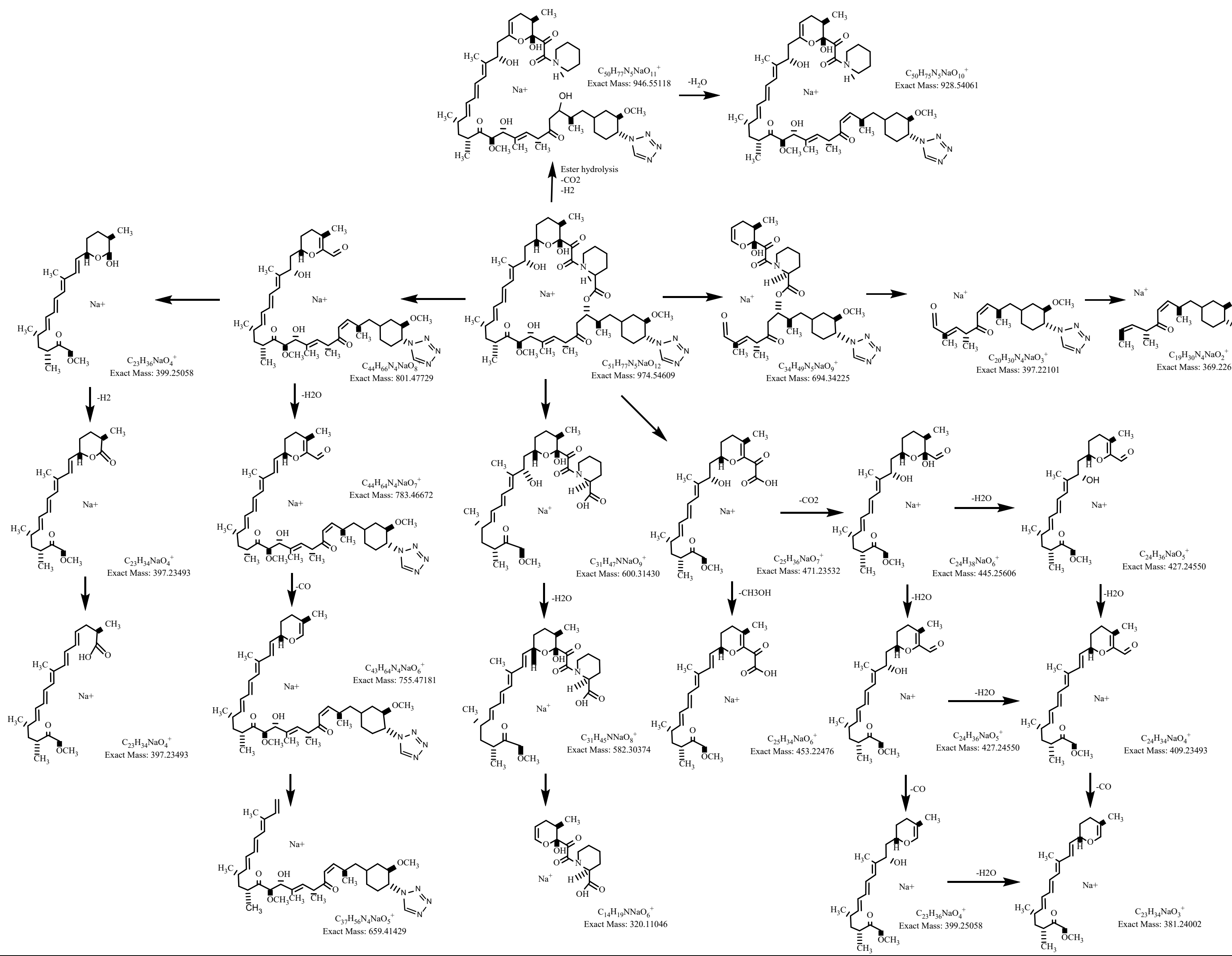

Characteristic Fragments of 16-O-Desmethyl Zotarolimus

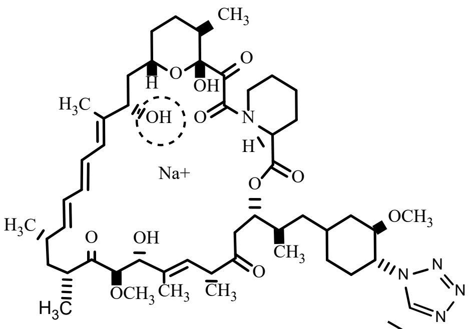

$C_{51}H_{77}N_5NaO_{12}^+$
Exact Mass: 974.54609

16-O-desmethyl zotarolimus

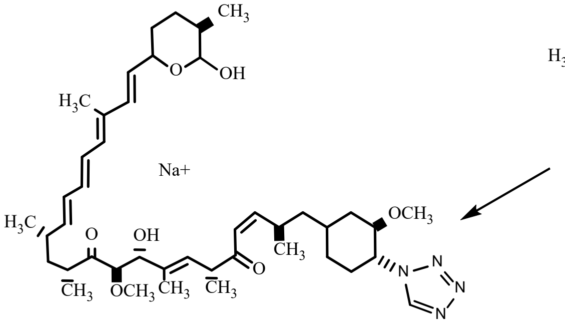

$C_{43}H_{66}N_4NaO_7^+$
Exact Mass: 773.48237

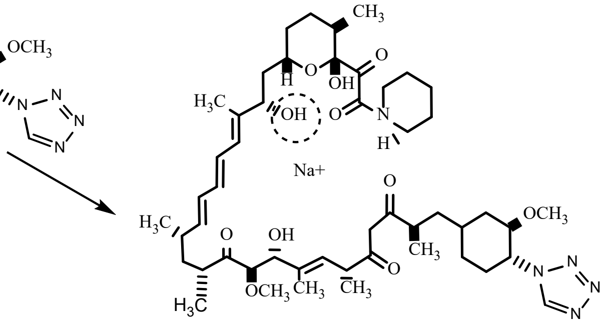

$C_{50}H_{77}N_5NaO_{11}^+$
Exact Mass: 946.55118

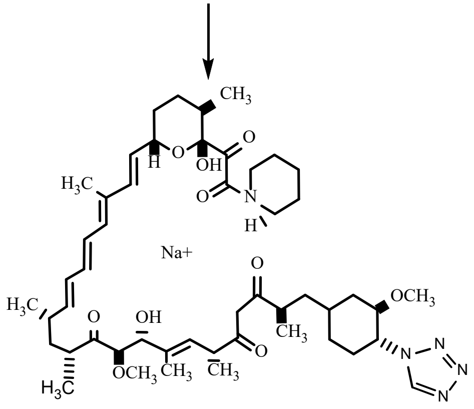

$C_{50}H_{75}N_5NaO_{10}^+$
Exact Mass: 928.54061

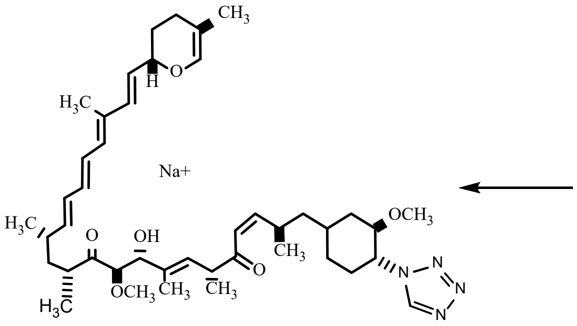

$C_{43}H_{64}N_4NaO_6^+$
Exact Mass: 755.47181

Δppm of 16-O-Desmethyl Zotarolimus Fragments

Fragment No.	Theoretical mass	Measured mass	Δppm
1	320.11046	320.11009	1.2
2	369.22610	369.22533	2.1
3	397.23493	397.23437	1.4
4	409.23493	409.23408	2.1
5	427.24550	427.24388	3.8
6	453.22476	453.22439	0.8
7	471.23532	471.23507	0.5
8	582.30374	582.30223	2.6
9	600.31430	600.31308	2.0
10	659.41429	659.41267	2.5
11	694.34225	694.34099	1.8
12	755.47181	755.46889	3.9
13	974.54609	974.54535	0.8

Structural Confirmation of 16-O-Desmethyl Zotarolimus, m/z = 1004.55666

Zotarolimus Fragments	16-O-desmethyl Zotarolimus Fragments	Comments	
320.11046	320.11009	no demethylation	Not conclusive
369.22610	369.22533	no demethylation	Rules out 39-O-desmethyl
397.23493	397.23437	no demethylation	Rules out 39-O-desmethyl
409.23493	409.23408	no demethylation	confirms 16-O-desmethyl after water elimination
427.24550	427.26090	Demethylation	confirms 16-O-desmethyl after water elimination
453.22476	453.22439	no demethylation	confirms 16-O-desmethyl after water elimination
485.25097	471.23507	Demethylation	16, or 27-O-desmethyl present
582.30374	582.30223	no demethylation	confirms 16-O-desmethyl after water elimination
614.32995	600.31308	Demethylation	16, or 27-O-desmethyl present
659.41429	659.41267	no demethylation	16, 27, 39-O-desmethyl present
694.34225	694.34099	no demethylation	Rules out 39-O-desmethyl
783.46672	783.46603	no demethylation	16, 27, 39-O-desmethyl present
988.56174	974.54535	Demethylation	16, 27, 39-O-desmethyl present
Characteristic Fragments	928 and 755		
Determinant patterns			Characteristic fragment for water loss (600 -18 = 582 m/z) of 16-O-desmethyl<br>Characteristic fragment for water loss (946 -18 = 928 m/z) of 16-O-desmethyl

39-O-Desmethyl Zotarolimus (m/z = 974.54609)

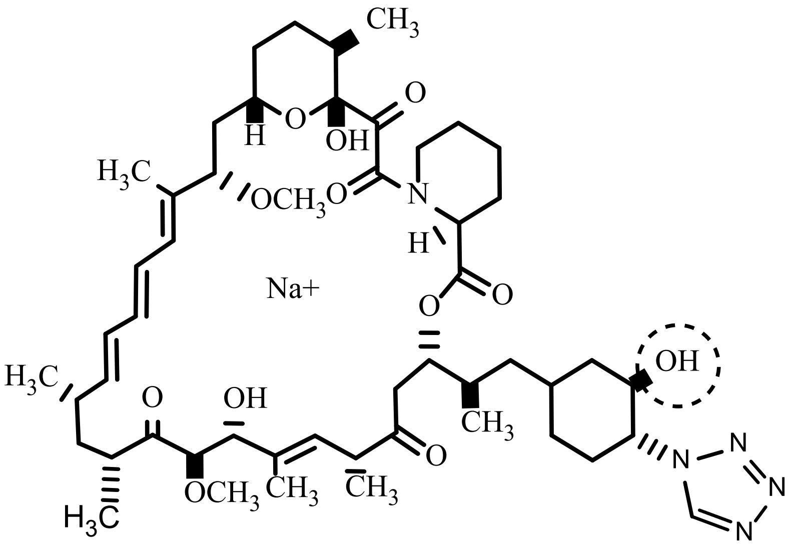

$C_{51}H_{77}N_5NaO_{12}^{+}$
Exact Mass: 974.54609

39-O-desmethyl zotarolimus

39-O-Desmethyl Zotarolimus Metabolite
Extracted Ion Chromatogram (EIC), m/z = 974.54609

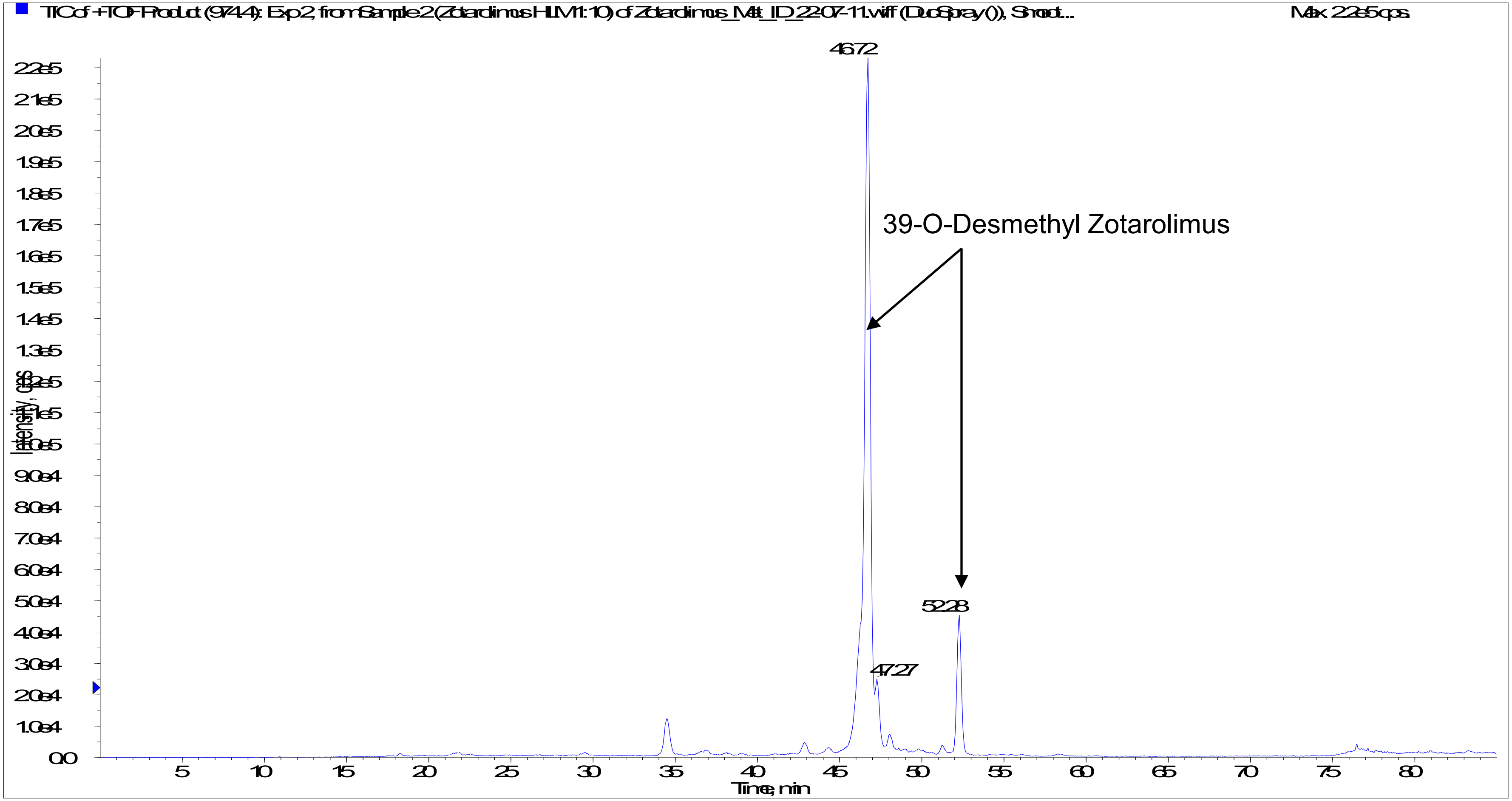

39-O-Desmethyl Zotarolimus Metabolite
Extracted Ion Chromatogram (EIC), m/z = 974.54609
RT = 46.72 min

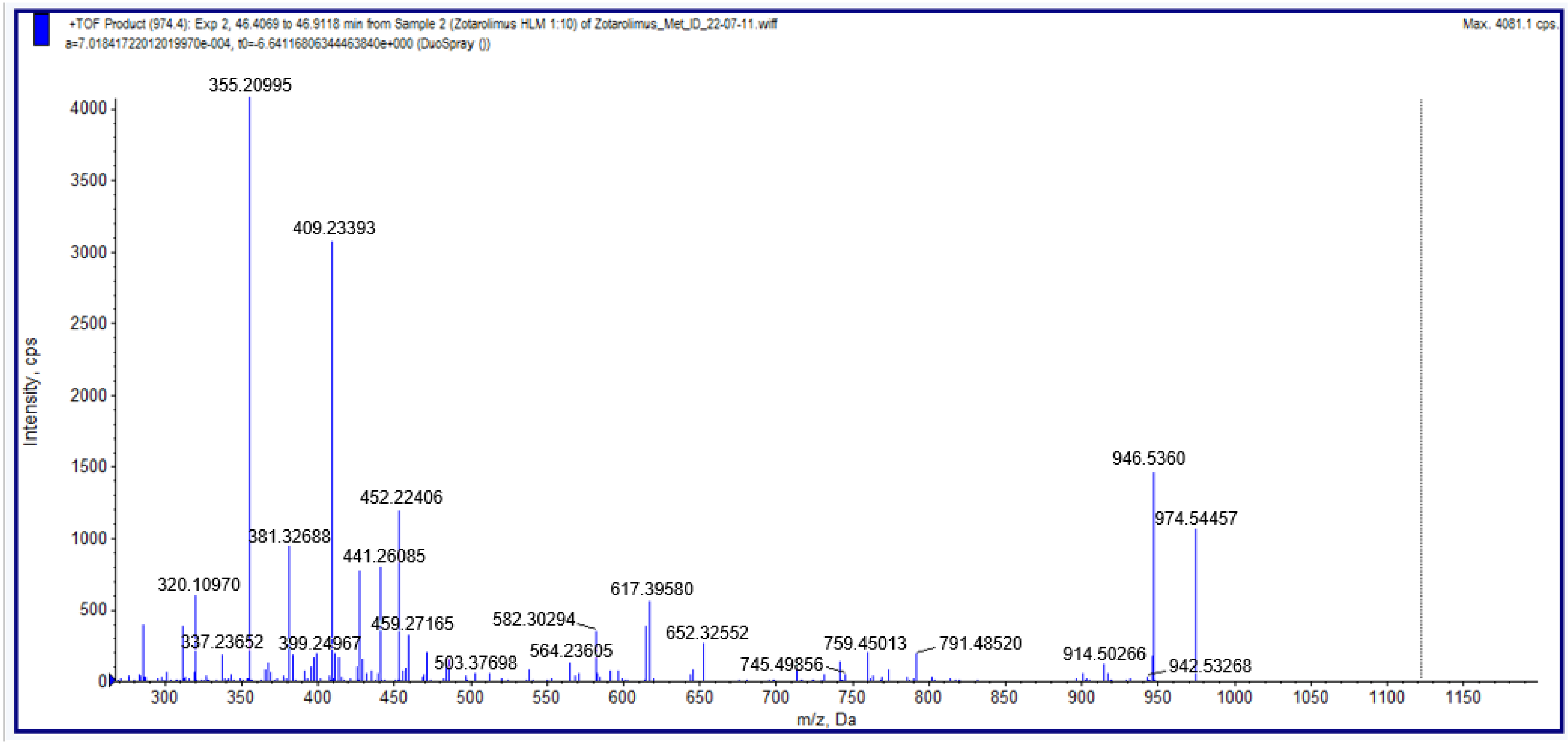

39-O-Desmethyl Zotarolimus Metabolite
Extracted Ion Chromatogram (EIC), m/z = 974.54609
RT = 52.28 min

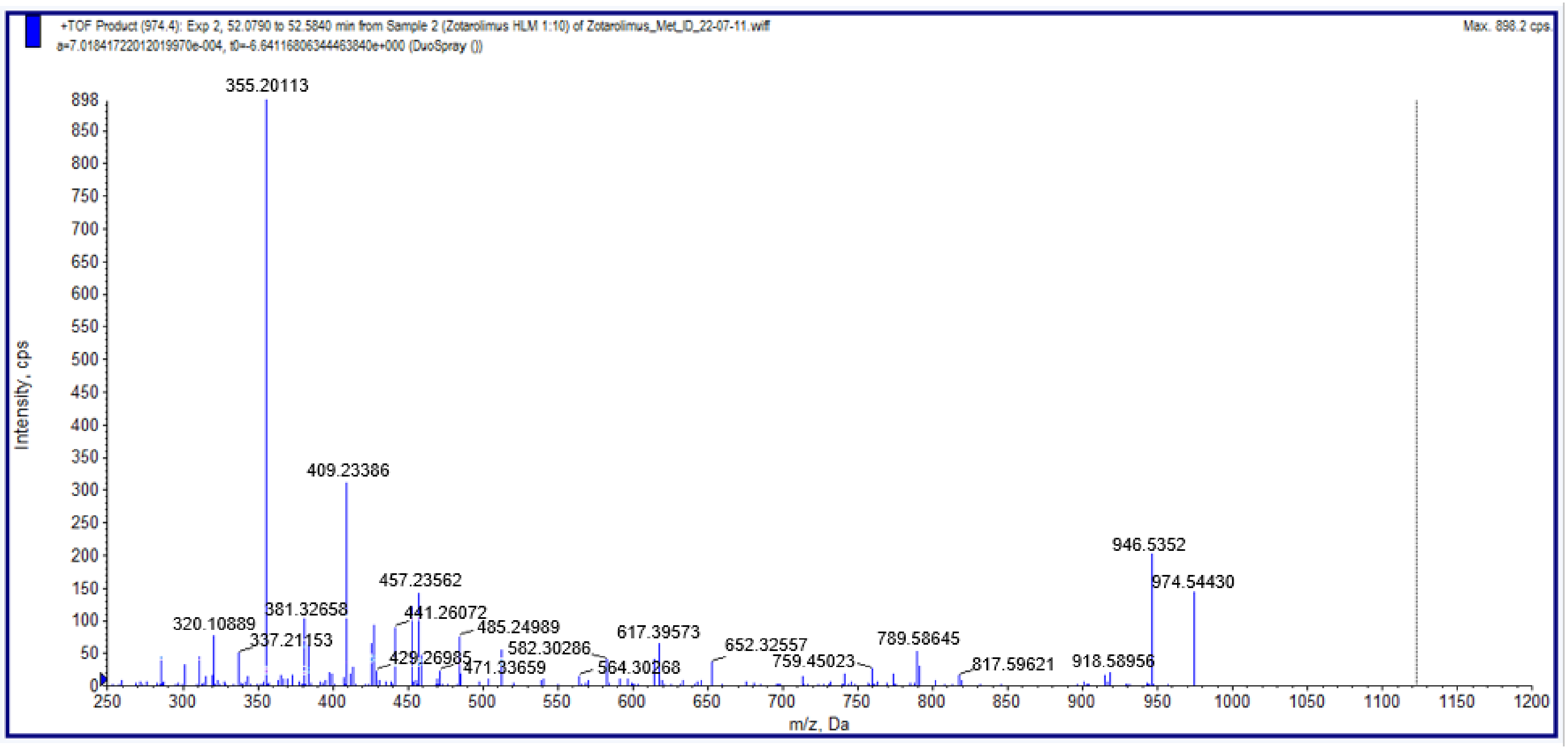

∆ppm of 39-O-Desmethyl Zotarolimus Fragments

Fragment No.	Theoretical mass	Measured mass	∆ppm
1	320.11046	320.1097	2.4
2	383.20536	383.20486	1.3
3	355.21045	355.20995	1.4
4	399.25058	399.24967	2.3
5	409.23493	409.23393	2.4
6	441.26115	441.26085	0.7
7	453.22476	453.22412	1.4
8	485.25097	485.25012	1.8
9	582.30374	582.30294	1.4
10	614.32995	614.32845	2.4
11	974.54609	974.54457	1.6

39-O-Desmethyl Zotarolimus Fragmentation Pattern

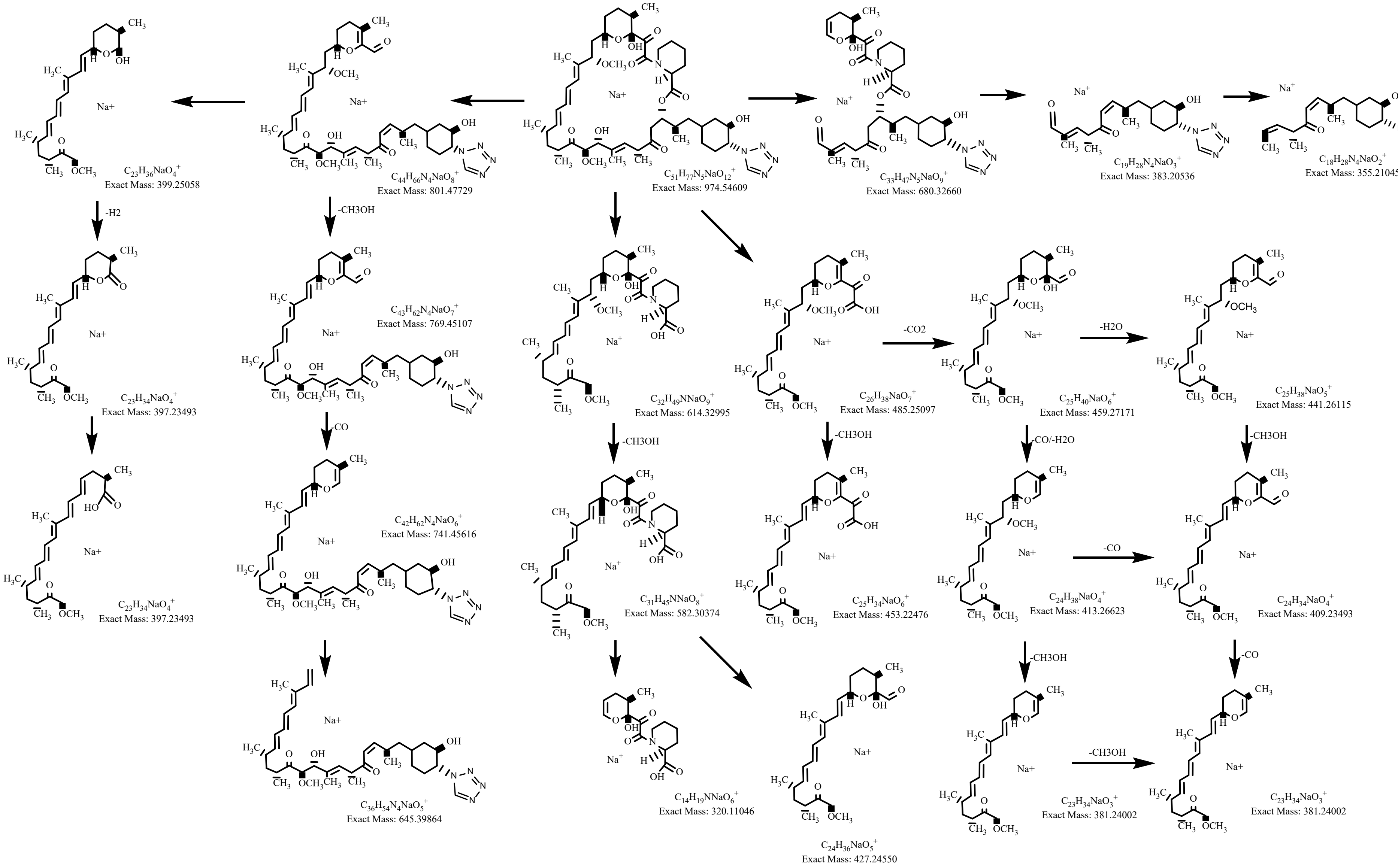

Structural Confirmation of 39-O-Desmethyl Zotarolimus, m/z = 1004.55666

Zotarolimus Fragments	39-O-desmethyl Zotarolimus Fragments	Comments	
320.11046	320.10975	no demethylation	Not conclusive
369.22610	355.20999	Demethylation	confirms 39-O-desmethyl
397.23493	ND	ND	ND
409.23493	409.23397	no demethylation	16, or 27-O-desmethyl present
441.26115	441.26089	no demethylation	rules out 16, and 27-O-desmethyl
453.22476	453.22439	no demethylation	16, or 27-O-desmethyl present
485.25097	485.25002	no demethylation	rules out 16, or 27-O-desmethyl
582.30374	582.30297	no demethylation	16, or 27-O-desmethyl present
614.32995	614.32857	no demethylation	rules out 16, and 27-O-desmethyl
659.41429	645.39864	Demethylation (very low)	16, 27, 39-O-desmethyl present
974.54609	974.54457	no demethylation	
Characteristic Fragments	355, 441, 614, and 741		No 369 confirms demethylation at C-39
Determinant patterns			

27-O-Desmethyl Zotarolimus (m/z = 974.54609)

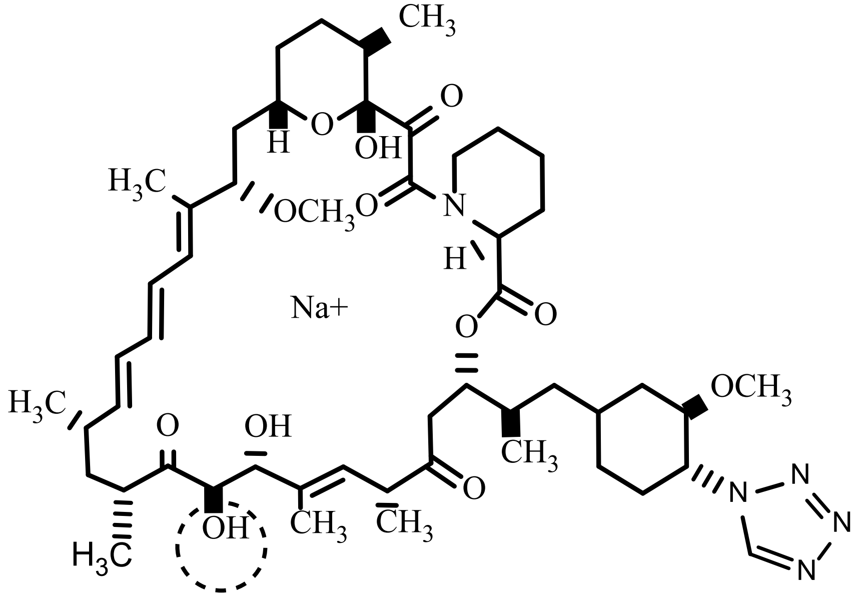

$C_{51}H_{77}N_5NaO_{12}^+$
Exact Mass: 974.54609

27-O-desmethyl zotarolimus

27-O-Desmethyl Zotarolimus Metabolite
Extracted Ion Chromatogram (EIC), m/z = 974.54609

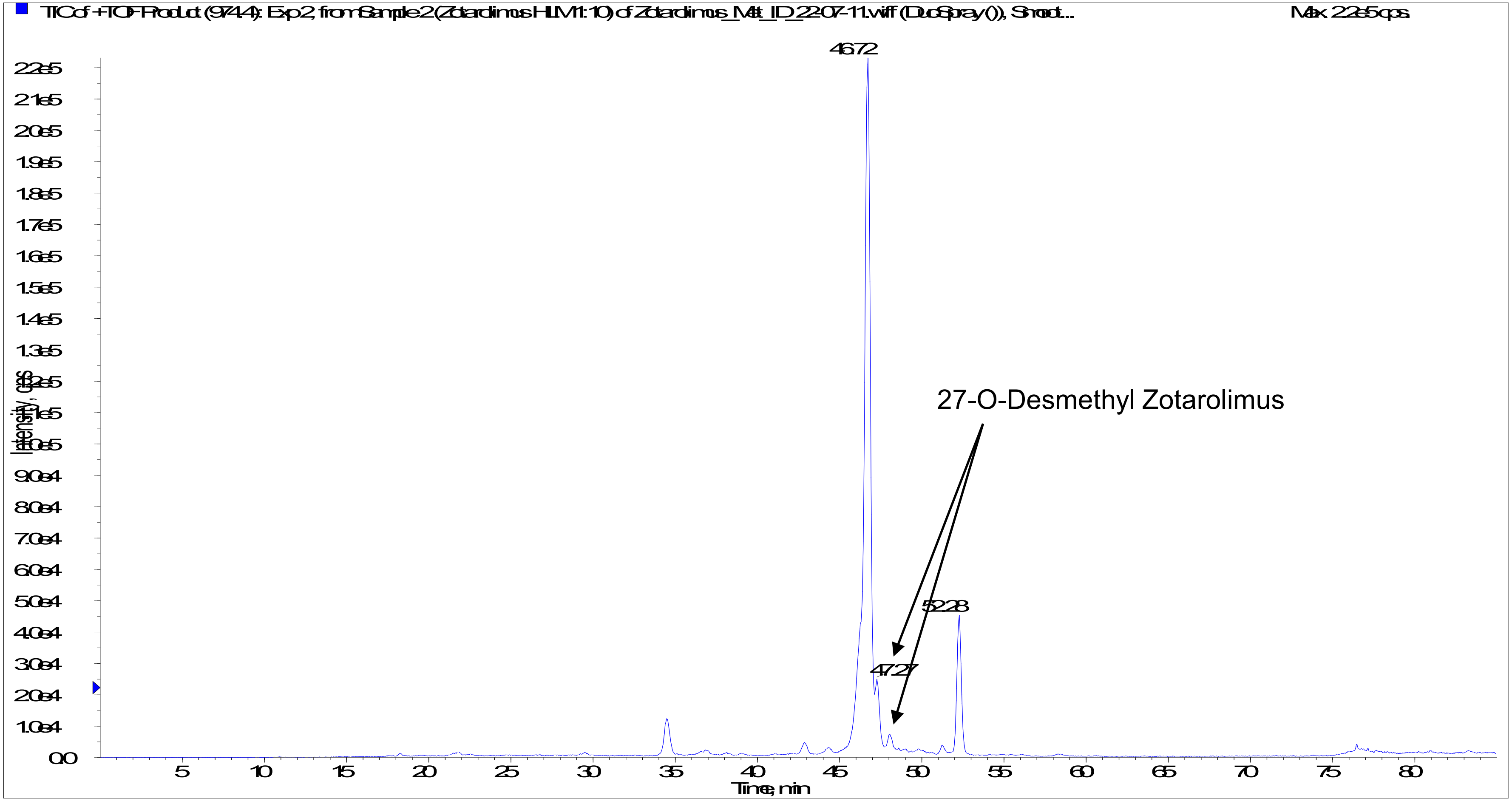

27-O-Desmethyl Zotarolimus Metabolite
Extracted Ion Chromatogram (EIC), m/z = 974.54609

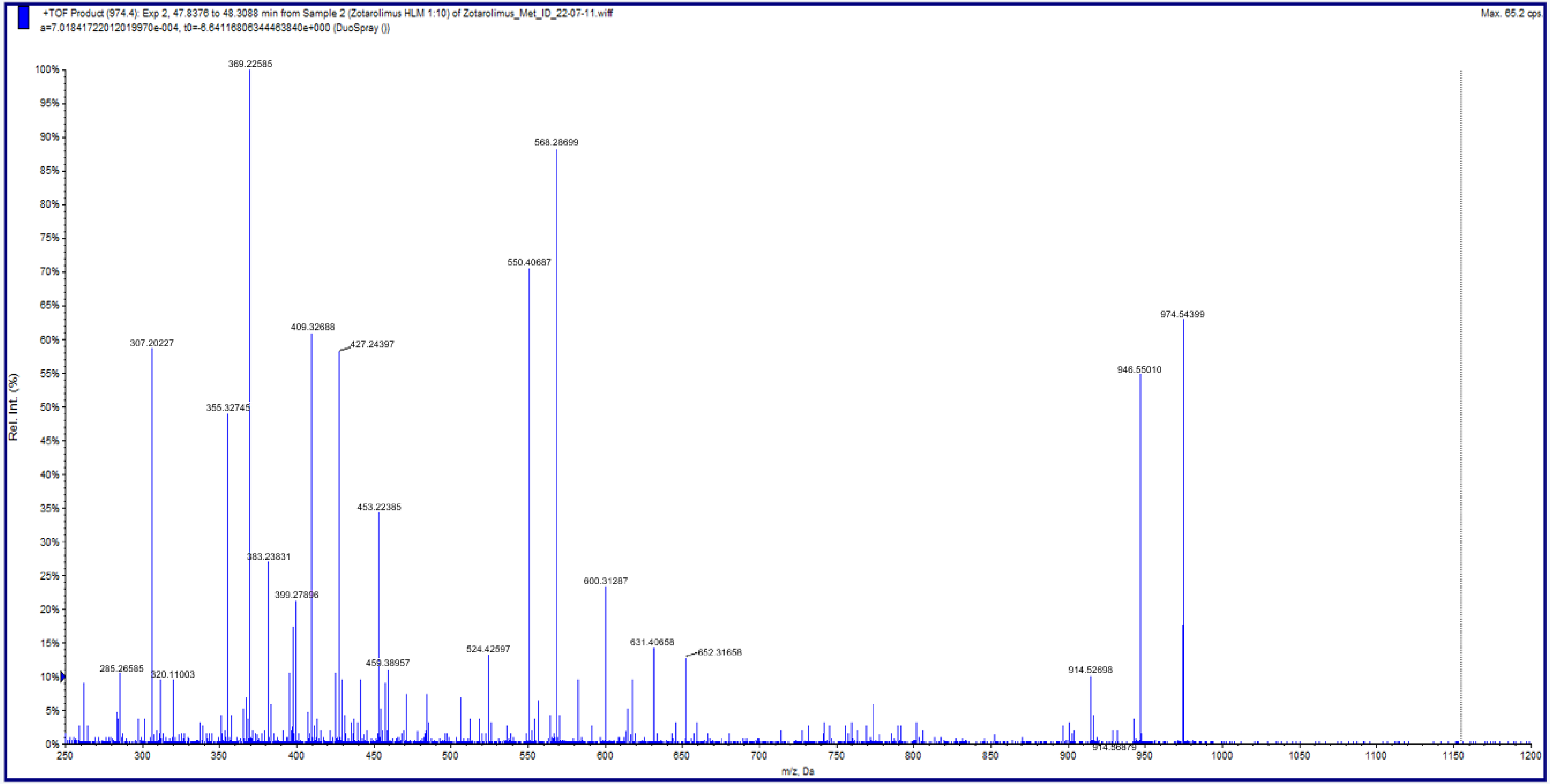

Δppm of 27-O-Desmethyl Zotarolimus Fragments

Fragment No.	Theoretical mass	Measured mass	Δppm
1	320.11046	320.11003	1.3
2	307.20324	307.20277	1.5
3	369.2261	369.22585	0.7
4	397.22101	397.21997	2.6
5	427.24550	427.24397	3.6
6	445.25606	445.25538	1.5
7	471.23532	471.23469	1.3
8	568.28809	568.28699	1.9
9	600.3143	600.31287	2.4
10	974.54609	974.54399	2.2

27-O-Desmethyl Zotarolimus Fragmentation Pattern

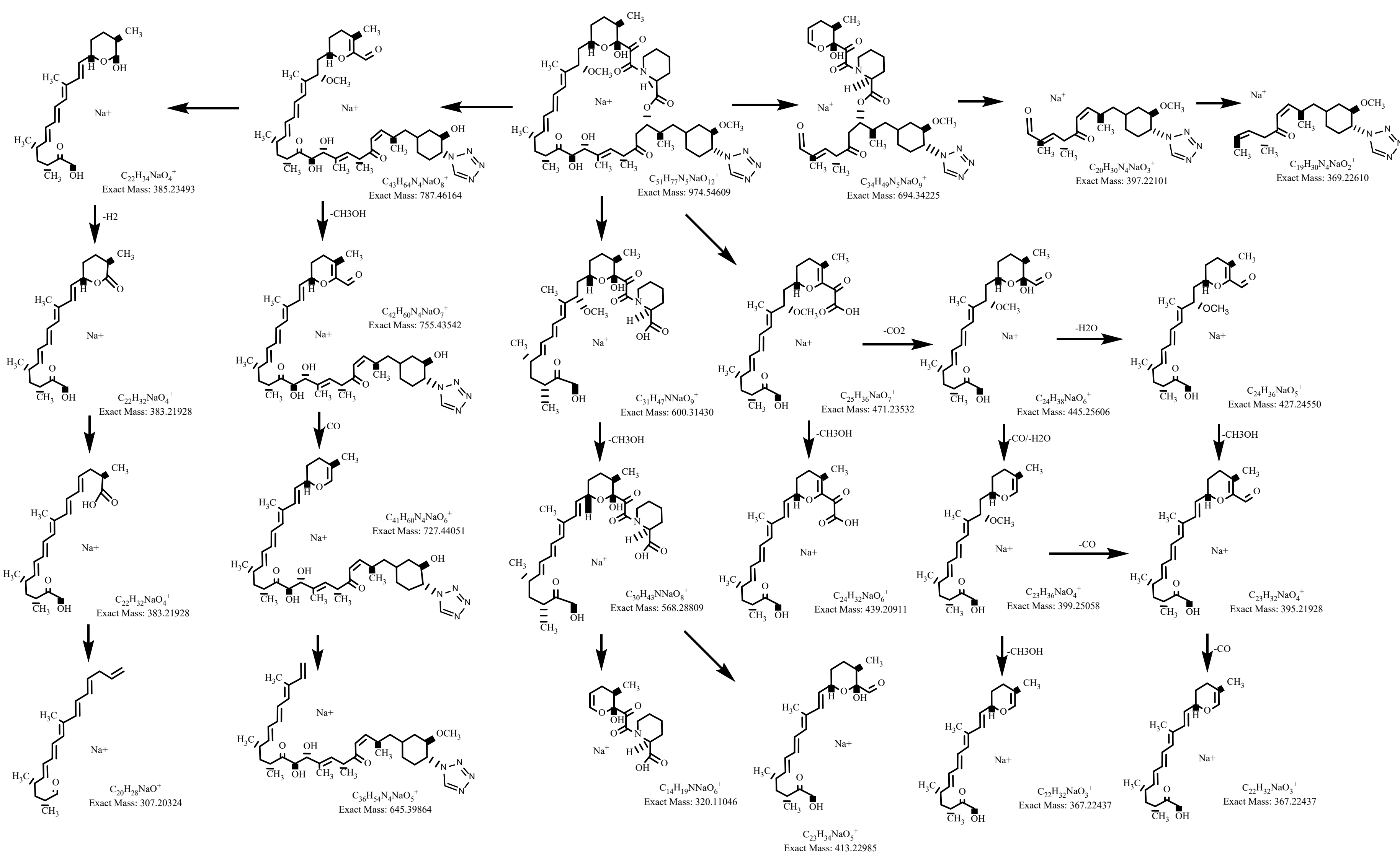

Structural Confirmation of 27-O-Desmethyl Zotarolimus, m/z = 1004.55666

Zotarolimus Fragments	27-O-desmethyl Zotarolimus Fragments	Comments	
320.11046	320.11003	no demethylation	Not conclusive
369.22610	369.22585	no demethylation	Rules out 39-O-desmethyl
397.23493	397.21997	no demethylation	Rules out 39-O-desmethyl
409.23493	395.21928	Demethylation	confirms 27-O-desmethyl
441.26115	427.24397	Demethylation	16, and 27-O-desmethyl present
453.22476	439.20911	nd	n/a
485.25097	471.23469	Demethylation	16, and 27-O-desmethyl present
582.30374	568.28699	Demethylation	confirms 27-O-desmethyl
614.32995	600.31287	Demethylation	16, and 27-O-desmethyl present
659.41429	645.39864	nd	27, and 39-O-desmethyl present
974.54609	974.54399	no demethylation	
Characteristic Fragments	307, 383, 395, 550, and 568		
Determinant patterns			No water loss at C-27 compared to the corresponding 16-ODM fragments<br>water loss at C-10 by fragment 568 – 18 = 550

Desmethyl, Hydroxy Zotarolimus Metabolites, m/z = 990.54101

Desmethyl, Hydroxy Zotarolimus Metabolites, m/z = 990.54101

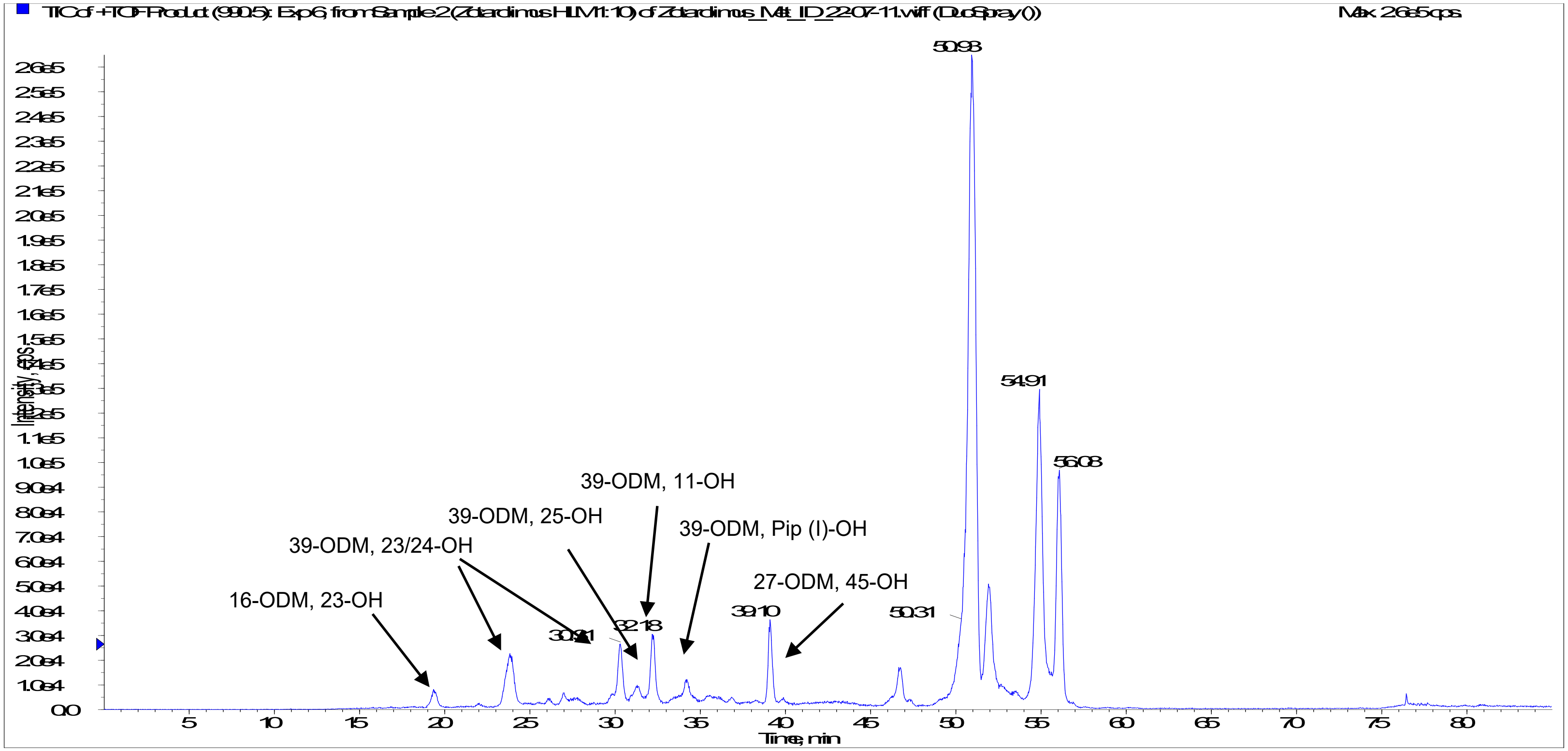

16-O-Desmethyl, 23-Hydroxy Zotarolimus, m/z = 990.54101

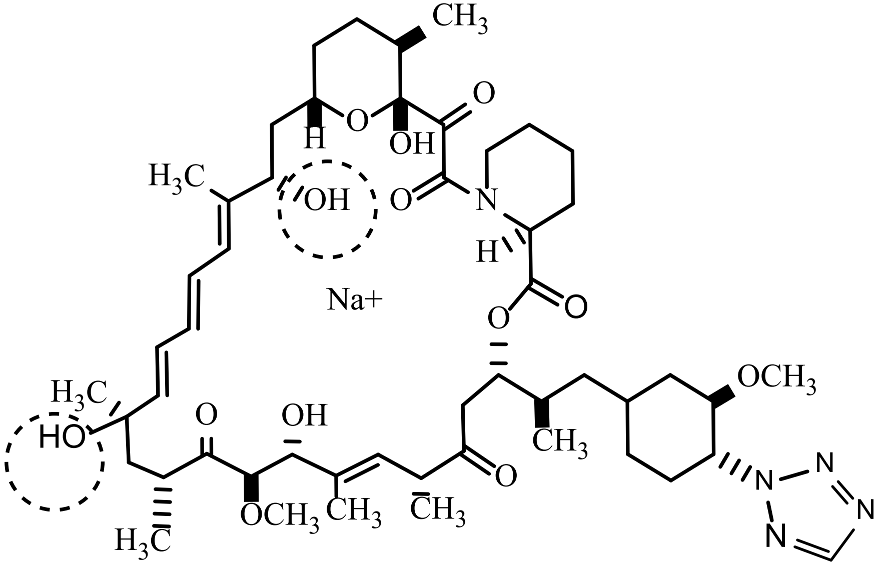

$C_{51}H_{77}N_5NaO_{13}^+$
Exact Mass: 990.54101

16-O-Desmethyl, 23-Hydroxy Zotarolimus m/z = 990.54101
Extracted Ion Chromatogram (EIC)

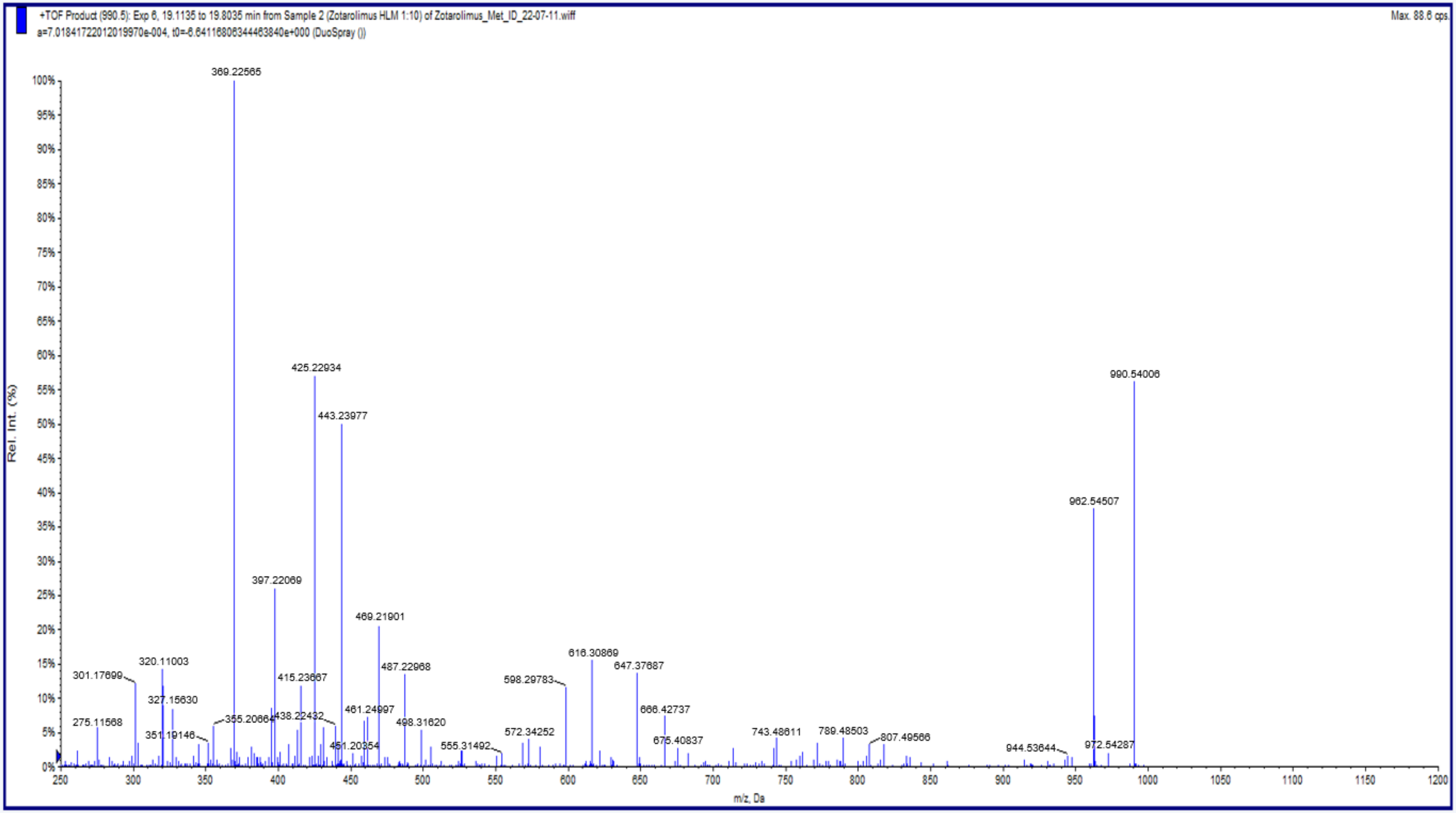

16-O-Desmethyl, 23-Hydroxy Zotarolimus Fragmentation Pattern

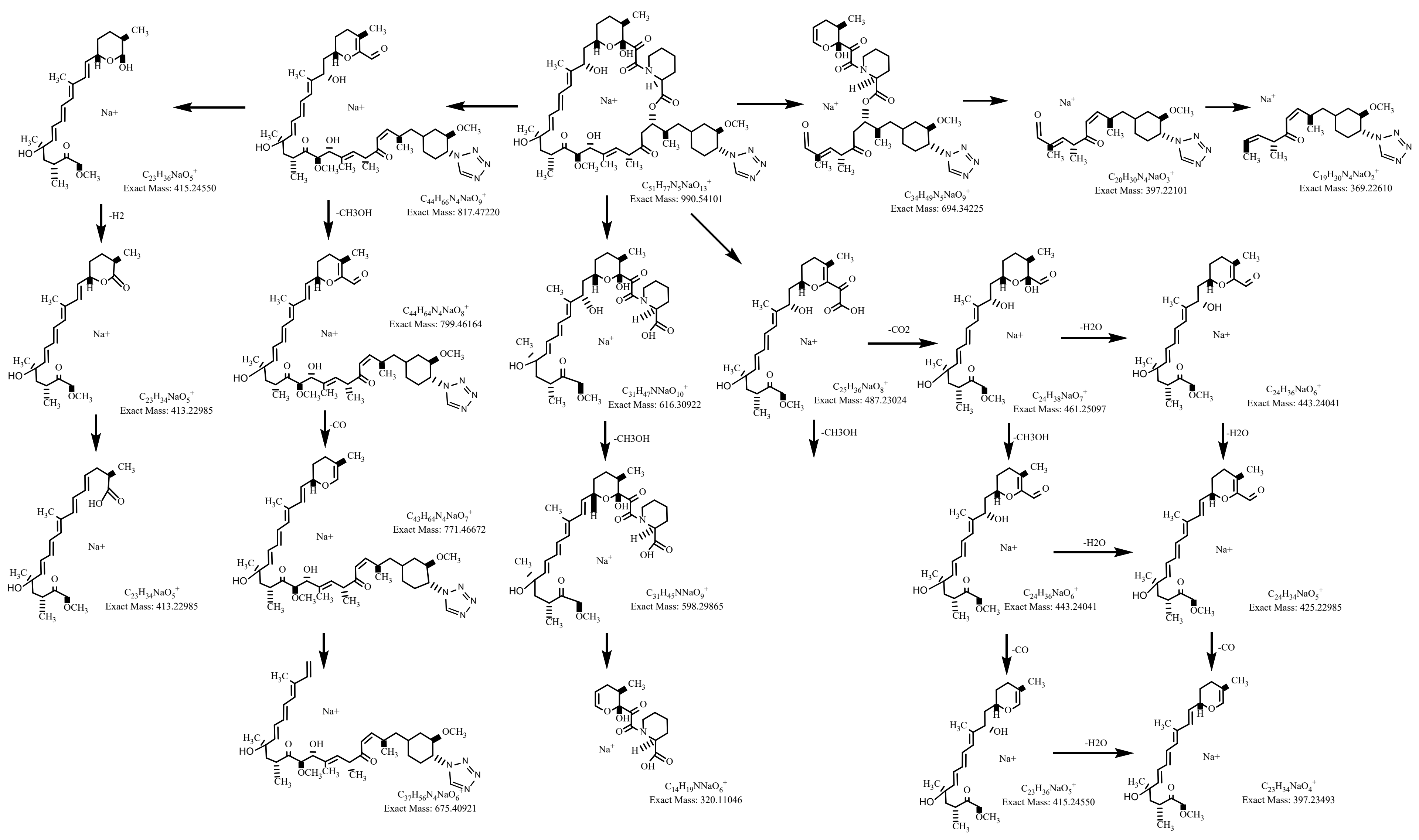

Δppm of 16-O-Desmethyl, 23-Hydroxy Zotarolimus

Fragment No.	Theoretical mass	Measured mass	Δppm
1	320.11046	320.11003	1.3
2	369.22610	369.22565	1.2
3	397.22101	397.22069	0.8
4	425.22985	425.22934	1.2
5	443.24041	443.23977	1.4
6	461.25097	461.24997	2.2
7	487.23024	487.22968	1.1
8	598.29865	598.29783	1.4
9	616.30922	616.30869	0.9
10	647.37791	647.37687	1.6
11	675.40921	675.40837	1.2
12	817.47220	817.47129	1.1
13	962.54609	962.54507	1.1
14	990.54101	990.54006	1.0

Characteristic Fragments of 16-O-desmethyl, 23-OH Zotarolimus (Water Loss)

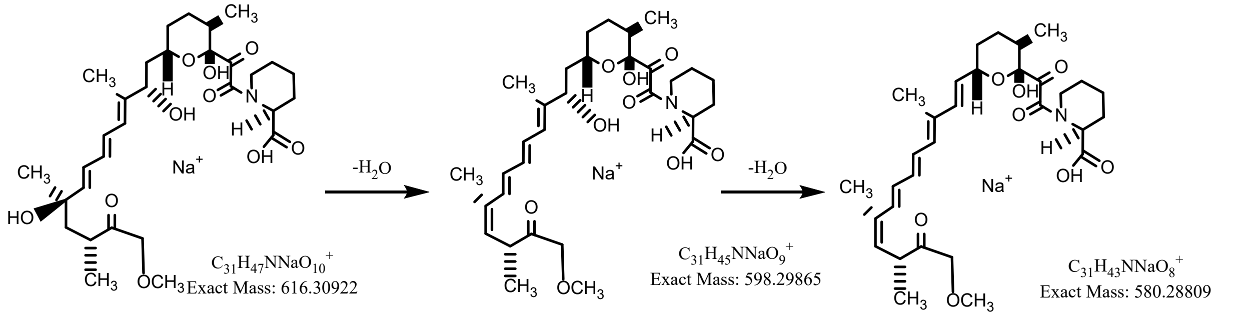

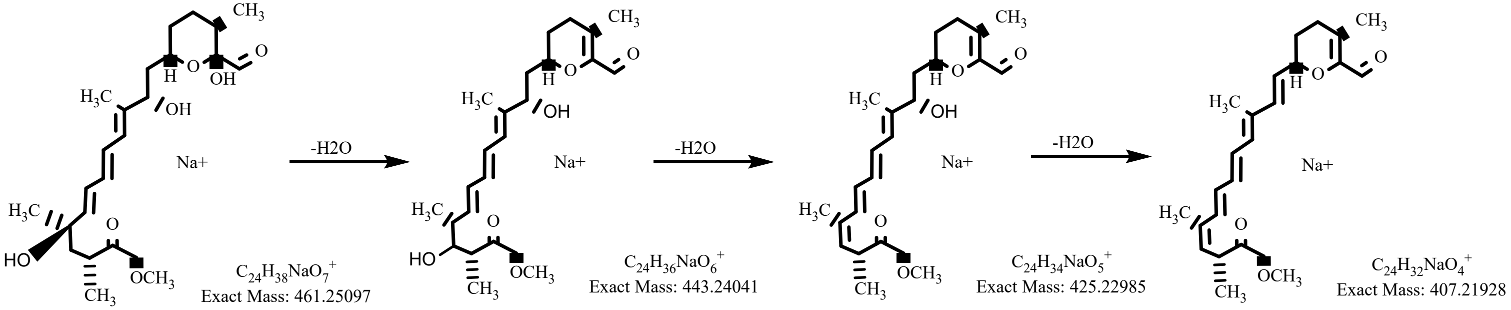

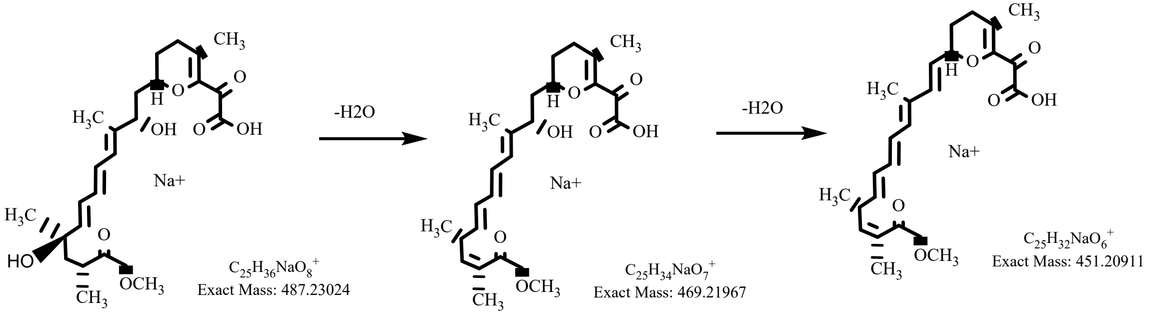

Characteristic Fragments of 39-O-Desmethyl, 23-Hydroxy Zotarolimus Fragmentation Pattern

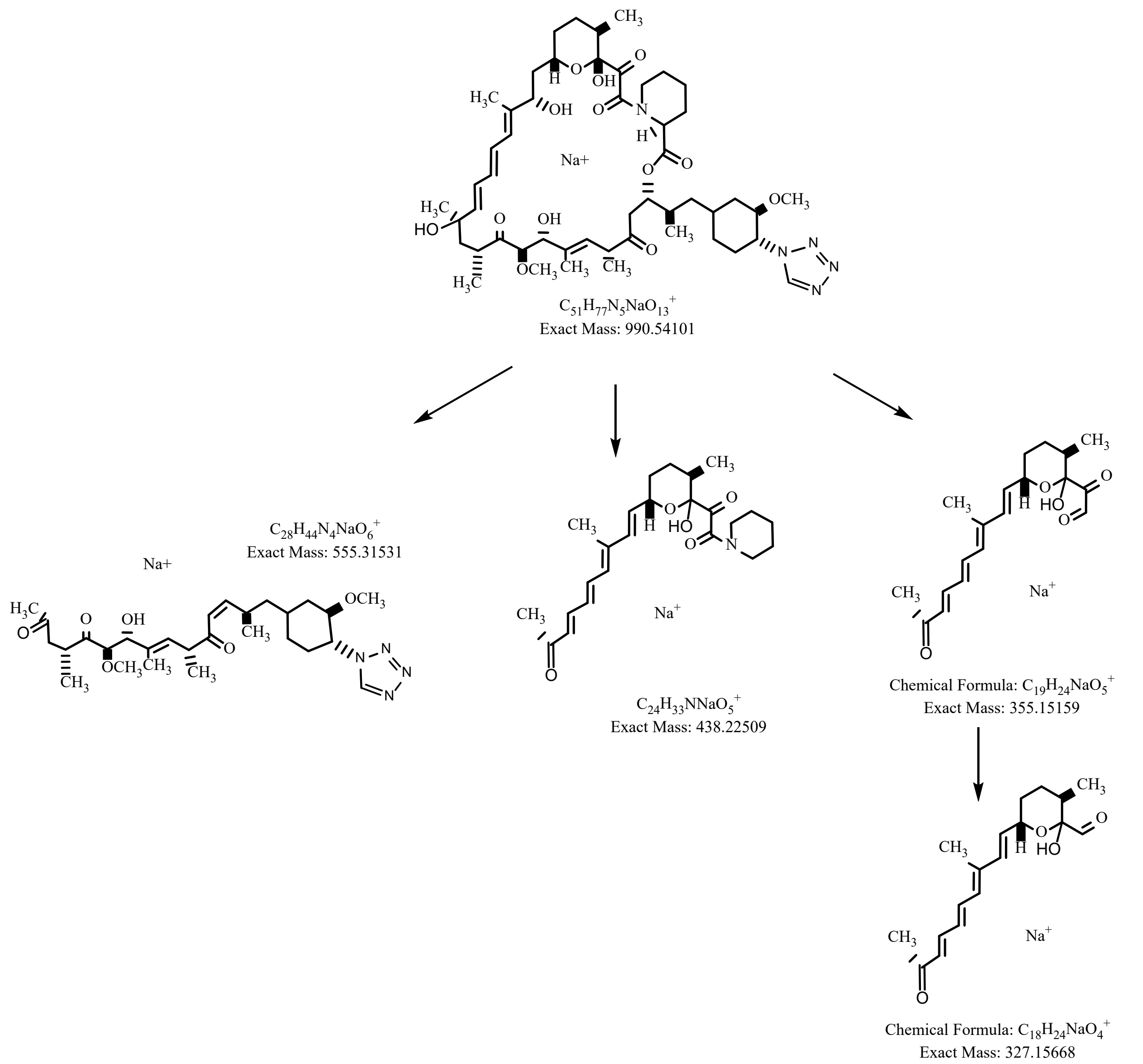

Structural Confirmation of 16-O-Desmethyl, 24-Hydroxy Zotarolimus

Zotarolimus Fragments	16-O-desmethyl, 23/24-hydroxy Zotarolimus Fragments	Comments	
320.11046	320.11003	no hydroxylation, no desmethylation	Rules out OH-pipiredine, 11, 12, and 14-OH
369.22610	369.22586	no hydroxylation, no desmethylation	Rules out 47, 48 or 49-OH and 39-O-desmethyl
397.22101	397.22069	no hydroxylation, no desmethylation	Rules out 47, 48 or 49-OH and 39-O-desmethyl
409.23493	425.22934	hydroxylation	11, 12, 14, 23, 24,45, or 46-OH present/ 16- or 27-O-desmethyl present
427.2455	443.23977	hydroxylation	11, 12, 14, 23, 24,45, or 46-OH present/ 16- or 27-O-desmethyl present
459.27171	461.24997	hydroxylation & demethylation	11, 12, 14, 23, 24,45, or 46-OH present/ 16- or 27-O-desmethyl present
485.25097	487.22968	hydroxylation & demethylation	11, 12, 14, 23, 24,45, or 46-OH present/ 16- or 27-O-desmethyl present
582.30374	598.29783	hydroxylation	11, 12, 14, 23, 24,45, or 45/46-OH, OH-Piperidine present/ 16- or 27-O-desmethyl present
614.32995	616.30869	hydroxylation & demethylation	11, 12, 14, 23, 24,45, or 45/46-OH, OH-Piperidine present/ 16- or 27-O-desmethyl present
659.41429	675.40837	hydroxylation	Rules out 11, 12, 14-OH, and OH-piperidine/ Rules out 16-O-desmethyl
815.49294	817.47129	hydroxylation & demethylation	11, 12, 14, 23, 23, 24,45, 46, 47, 48 or 49-OH present/ 16- or 27-O-desmethyl
988.56174	990.54006	hydroxylation & demethylation	
Characteristic Fragments		327, 355, 438, 451, 555, 598, 616	451 due to water loss of 469 confirms 27-O-desmethyl zotarolimus (no water loss would be possible if 27-O-desmethyl presents). Characteristic fragment for water loss (616 -18 = 598 m/z) of 23 and 24-OH
Determinant patterns		Water Loss	The hydroxylation of 23 and 24-OH located near the conjugated triene energetically favor the loss of water.

39-O-Desmethyl, 23/24-Hydroxy Zotarolimus, m/z = 990.54101

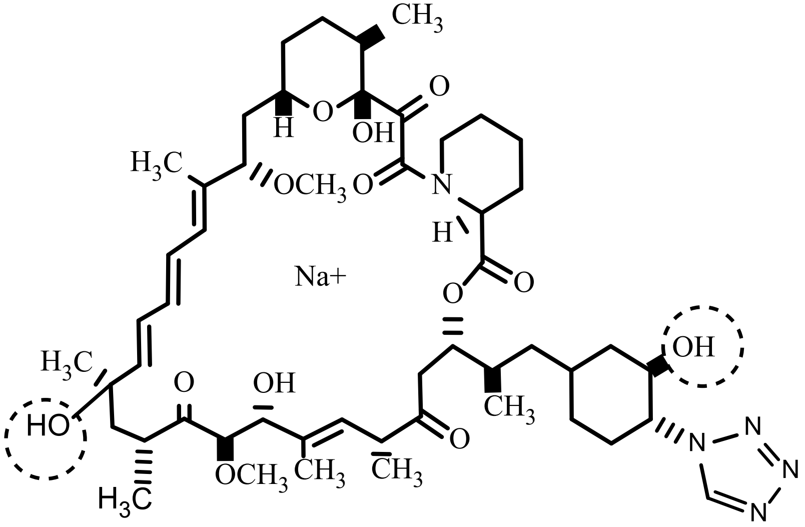

$C_{51}H_{77}N_5NaO_{13}^+$
Exact Mass: 990.54101

39-O-Desmethyl, 23-Hydroxy Zotarolimus

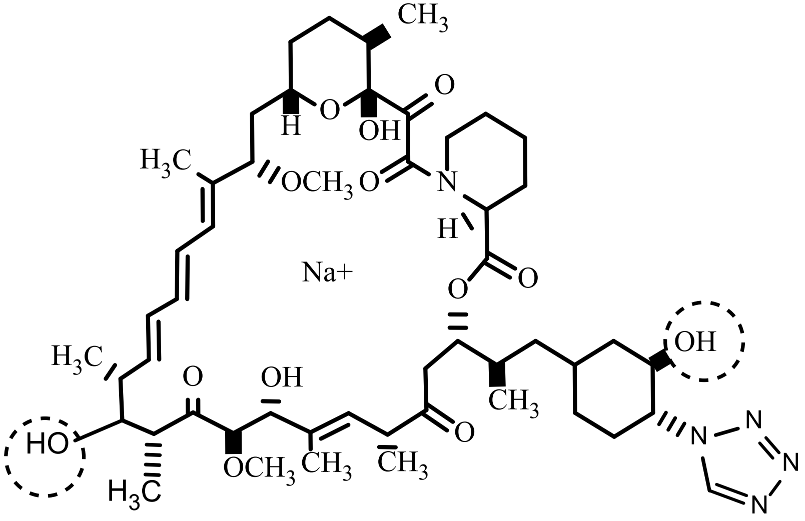

$C_{51}H_{77}N_5NaO_{13}^+$
Exact Mass: 990.54101

39-O-Desmethyl, 24-Hydroxy Zotarolimus

39-O-Desmethyl, 23/24-Hydroxy Zotarolimus, m/z = 990.54101

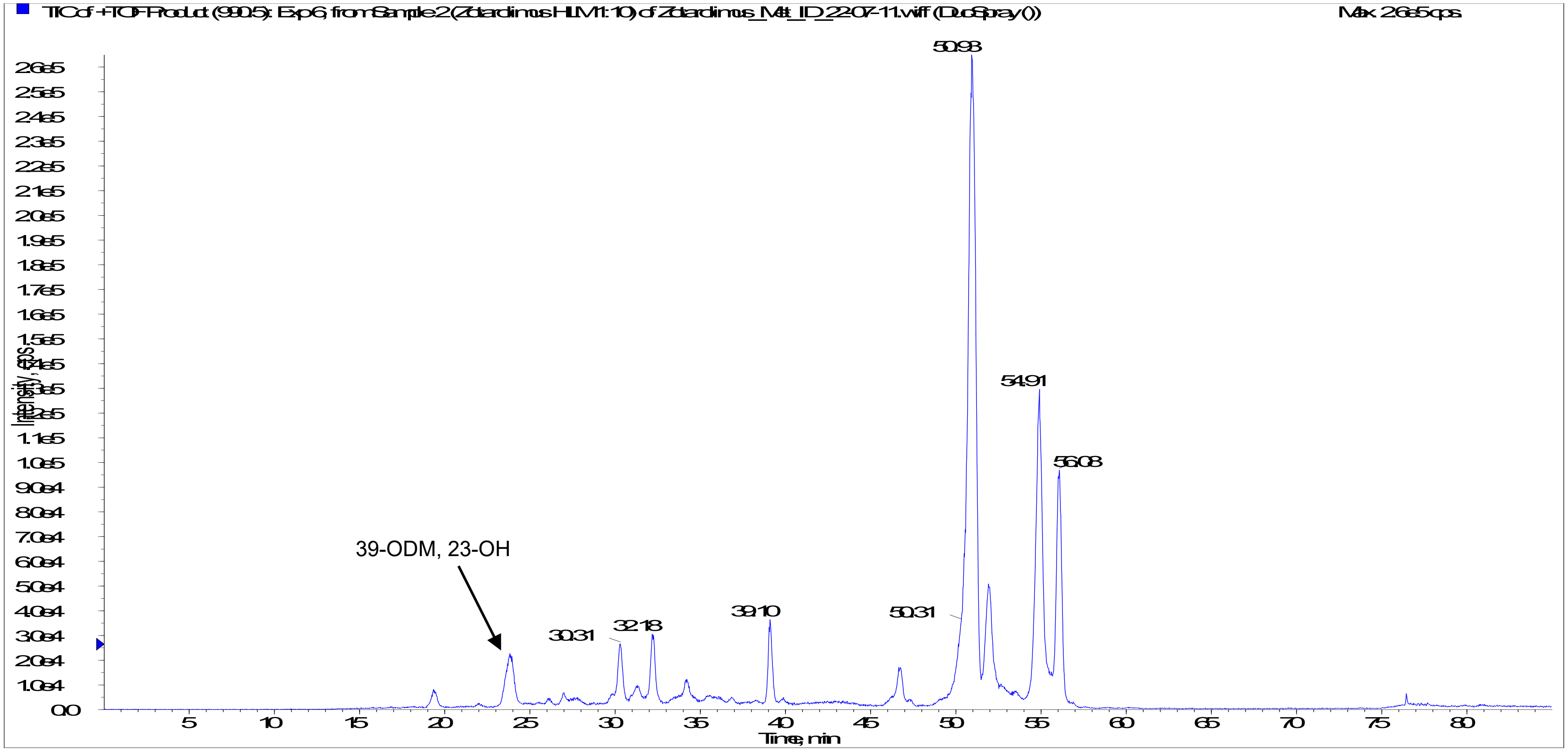

39-O-Desmethyl, 23/24-Hydroxy Zotarolimus , m/z = 990.54101

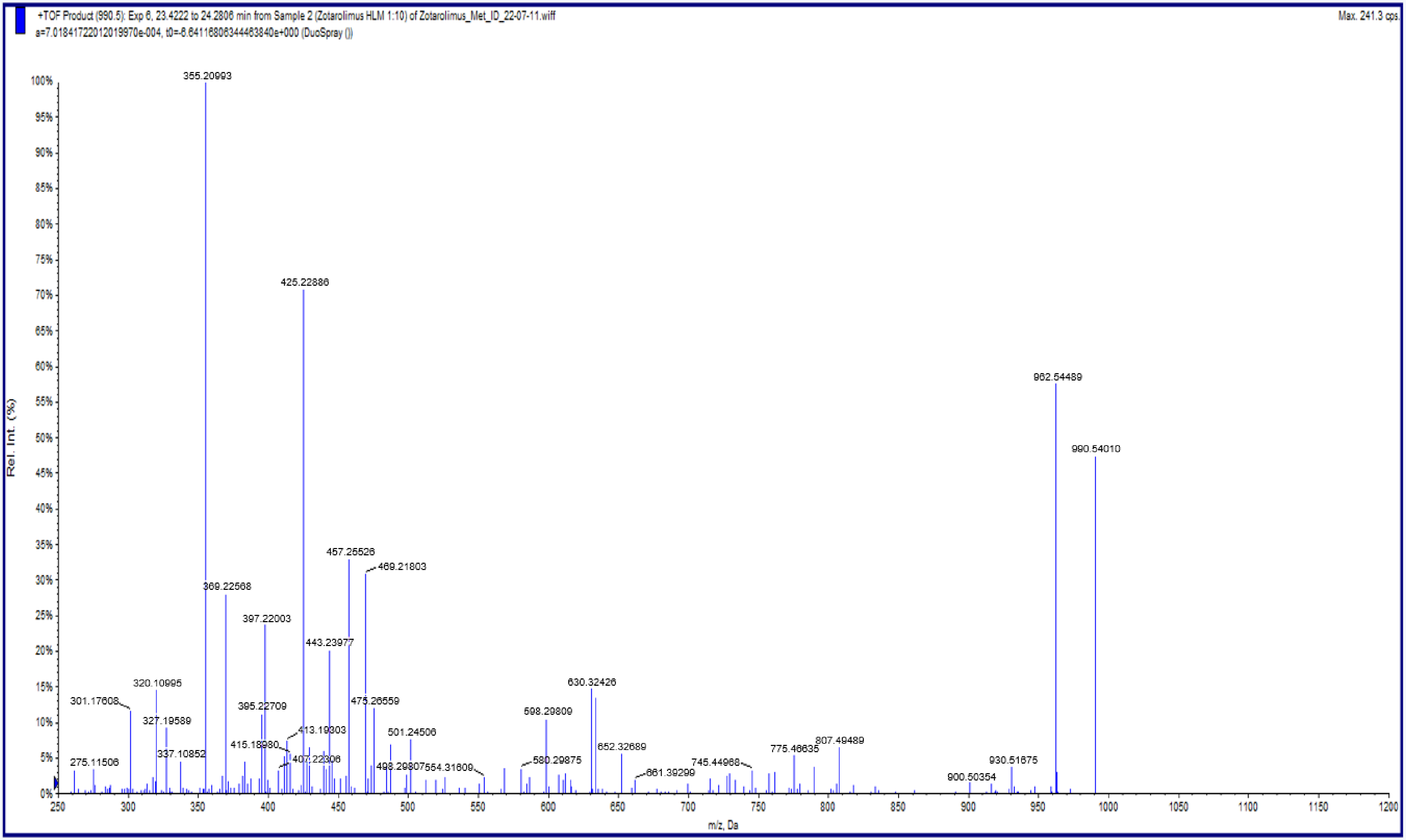

39-O-Desmethyl, 23-Hydroxy Zotarolimus Fragmentation Pattern

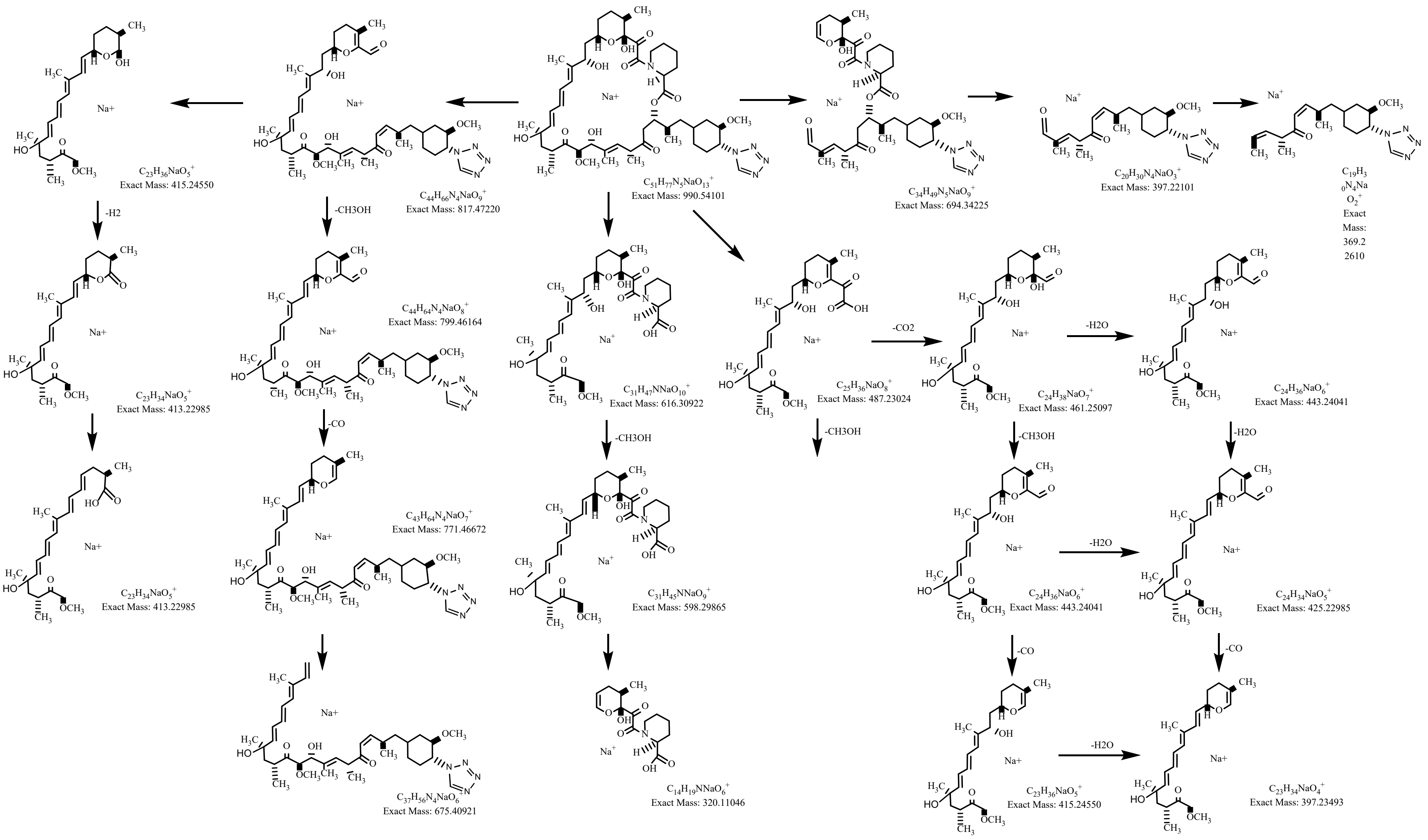

Δppm of 39-O-Desmethyl, 23/24-Hydroxy Zotarolimus

Fragment No.	Theoretical mass	Measured mass	Δppm
1	320.11046	320.10995	1.6
2	355.21045	355.20993	1.5
3	369.22610	369.22568	1.1
4	397.22101	397.22003	2.5
5	425.22985	425.22886	2.3
6	443.24041	443.23977	1.4
7	457.25606	457.25526	1.7
8	469.21967	469.21803	3.5
9	475.26662	475.26559	2.2
10	501.24589	501.24506	1.7
11	598.29865	598.29809	0.9
12	630.32487	630.32426	1.0
13	661.39356	661.39299	0.9
14	757.45107	757.45008	1.3
15	990.54101	990.54010	0.9

Characteristic Fragments of 39-O-desmethyl, 23-OH Zotarolimus (Water Loss)

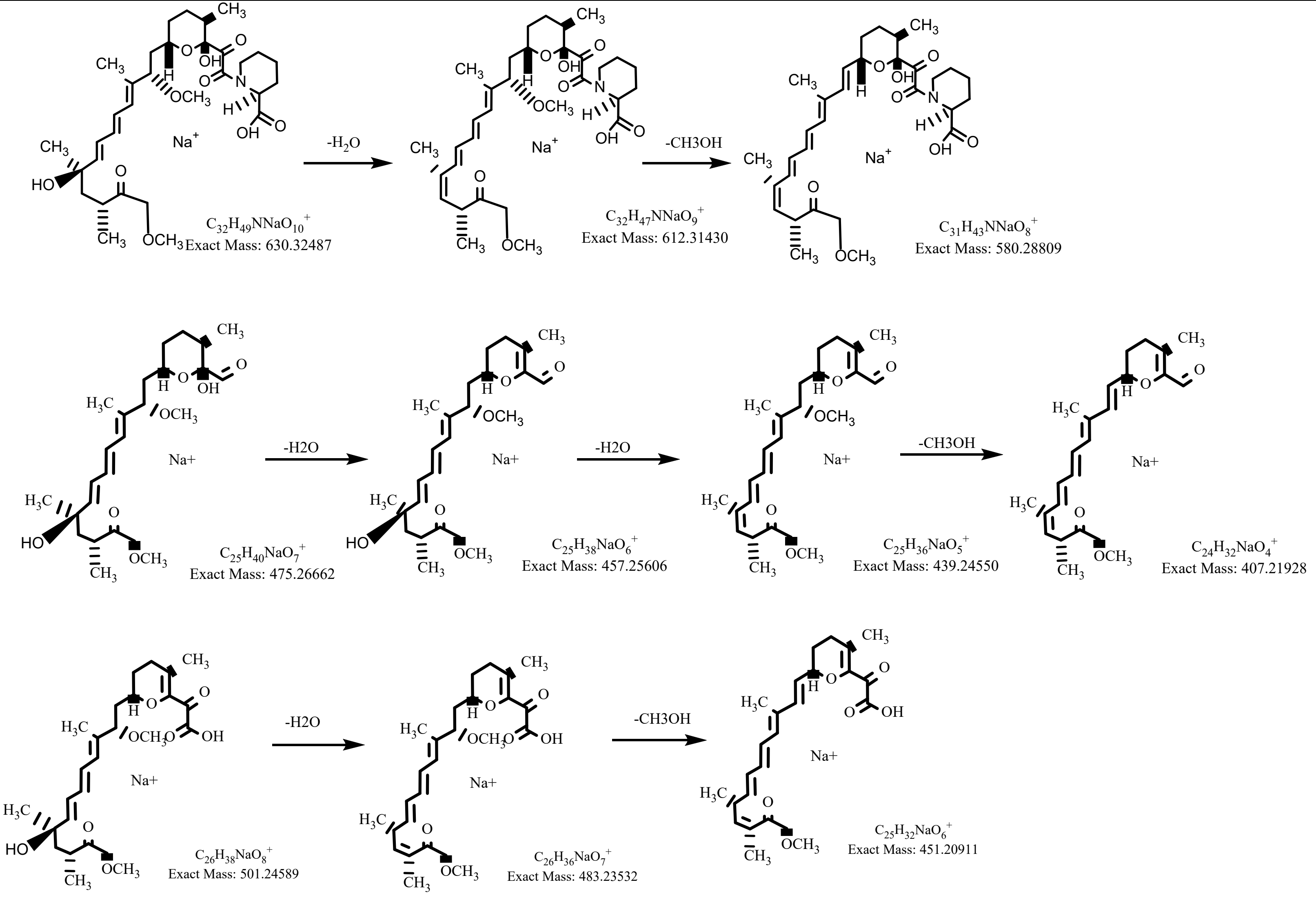

Structures of 23-OH was only shown

Characteristic Fragments of 39-O-desmethyl, 23-OH vs 24-OH Zotarolimus

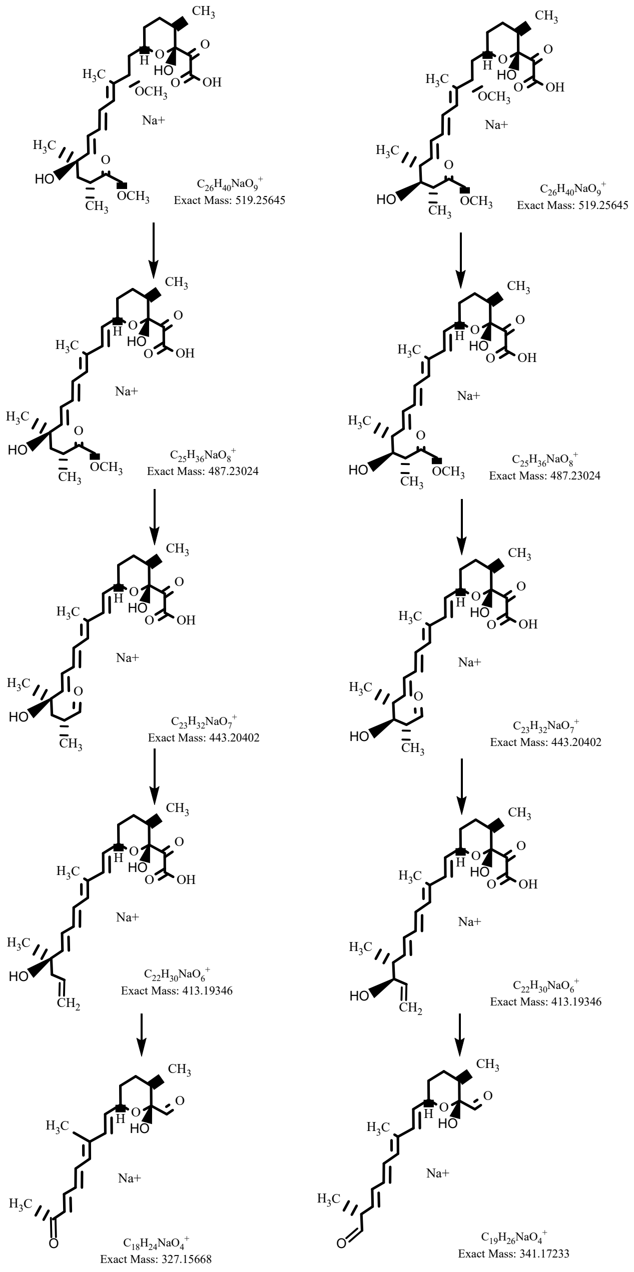

Structural Confirmation of 39-O-Desmethyl, 23-Hydroxy Zotarolimus

Zotarolimus Fragments	39-O-desmethyl, 23/24-hydroxy Zotarolimus Fragments	Comments	
320.11046	320.10995	no hydroxylation, no desmethylation	Rules out OH-pipiredine, 11, 12, and 14-OH
369.22610	355.20993	desmethylation	Confirms 39-O-desmethyl
381.24002	397.22003	hydroxylation	11, 12, 14, 23, 24,45, or 46-OH present
409.23493	425.22886	hydroxylation	11, 12, 14, 23, 24,45, or 46-OH present
427.2455	443.23977	hydroxylation	11, 12, 14, 23, 24,45, or 46-OH present
459.27171	475.26559	hydroxylation	11, 12, 14, 23, 24, 45, or 45/46-OH present/ Rules out 16- and 27-O-desmethyl
485.25097	501.24506	hydroxylation	11, 12, 14, 23, 24, 45, or 45/46-OH present/ Rules out 16- and 27-O-desmethyl
582.30374	598.29809	hydroxylation	11, 12, 14, 23, 24, 45, or 45/46-OH present/ Rules out 27-O-desmethyl
614.32995	630.32426	hydroxylation	11, 12, 14, 23, 24, 45, or 45/46-OH present/ Rules out 16- and 27-O-desmethyl
659.41429	661.39299	hydroxylation	Rules out 11, 12, 14-OH, and OH-piperidine
755.47181	757.45008	hydroxylation, desmethylation	11, 12, 14, 23, 24,45, or 45/46-OH present/ 16- 27-. or 39-O-desmethyl
988.56174	990.54010	demethylation	
Characteristic Fragments		327.15668 fragments confirms 23-OH	451 due to methanol loss of 501 Rules out 16-O-desmethyl zotarolimus<br>425 due to methanol loss of 457 Rules out 16-O-desmethyl zotarolimus
Determinant patterns			Additional water loss for 23/24-OH

39-O-Desmethyl, 24-Hydroxy Zotarolimus, m/z = 990.54101

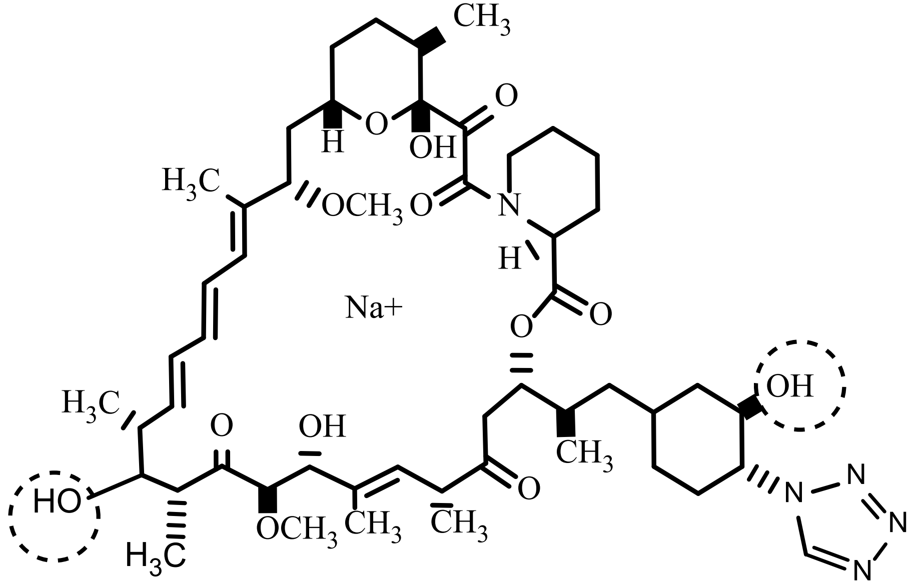

$C_{51}H_{77}N_5NaO_{13}^+$
Exact Mass: 990.54101

39-O-Desmethyl, 24-Hydroxy Zotarolimus Metabolites, m/z = 990.54101

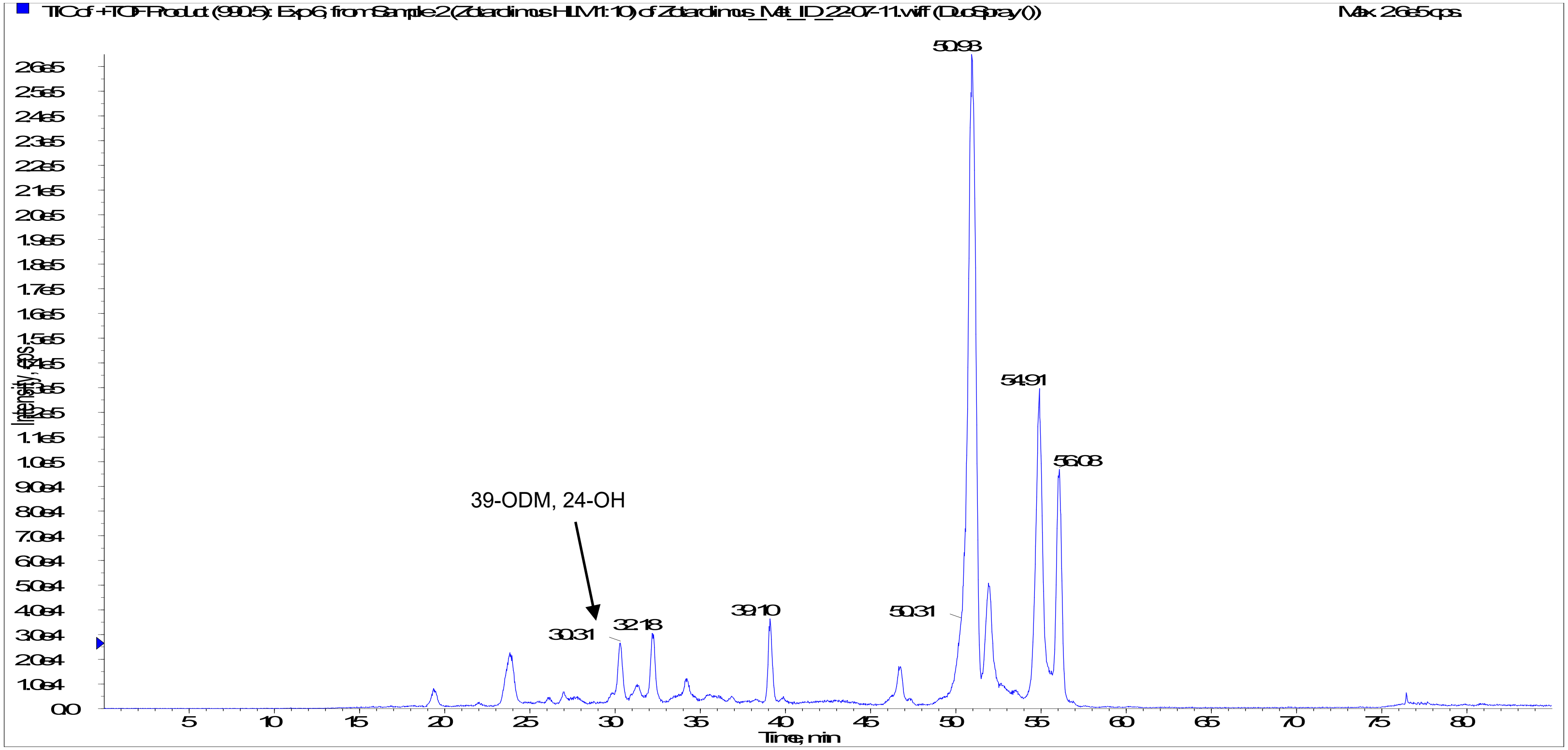

39-O-Desmethyl, 24-Hydroxy Zotarolimus m/z = 990.54101
Extracted Ion Chromatogram (EIC)

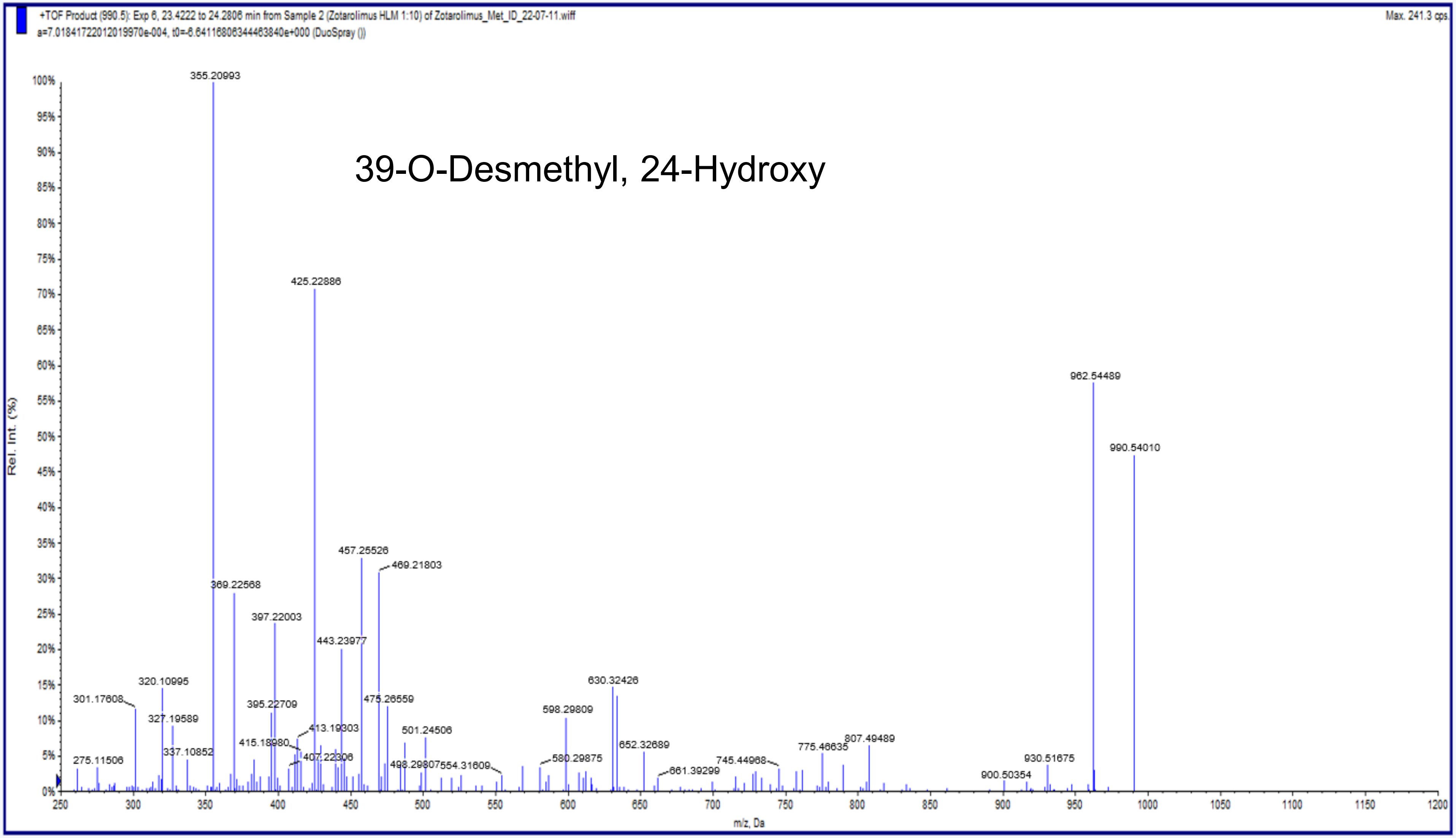

39-O-Desmethyl, 24-Hydroxy Zotarolimus Fragmentation Pattern

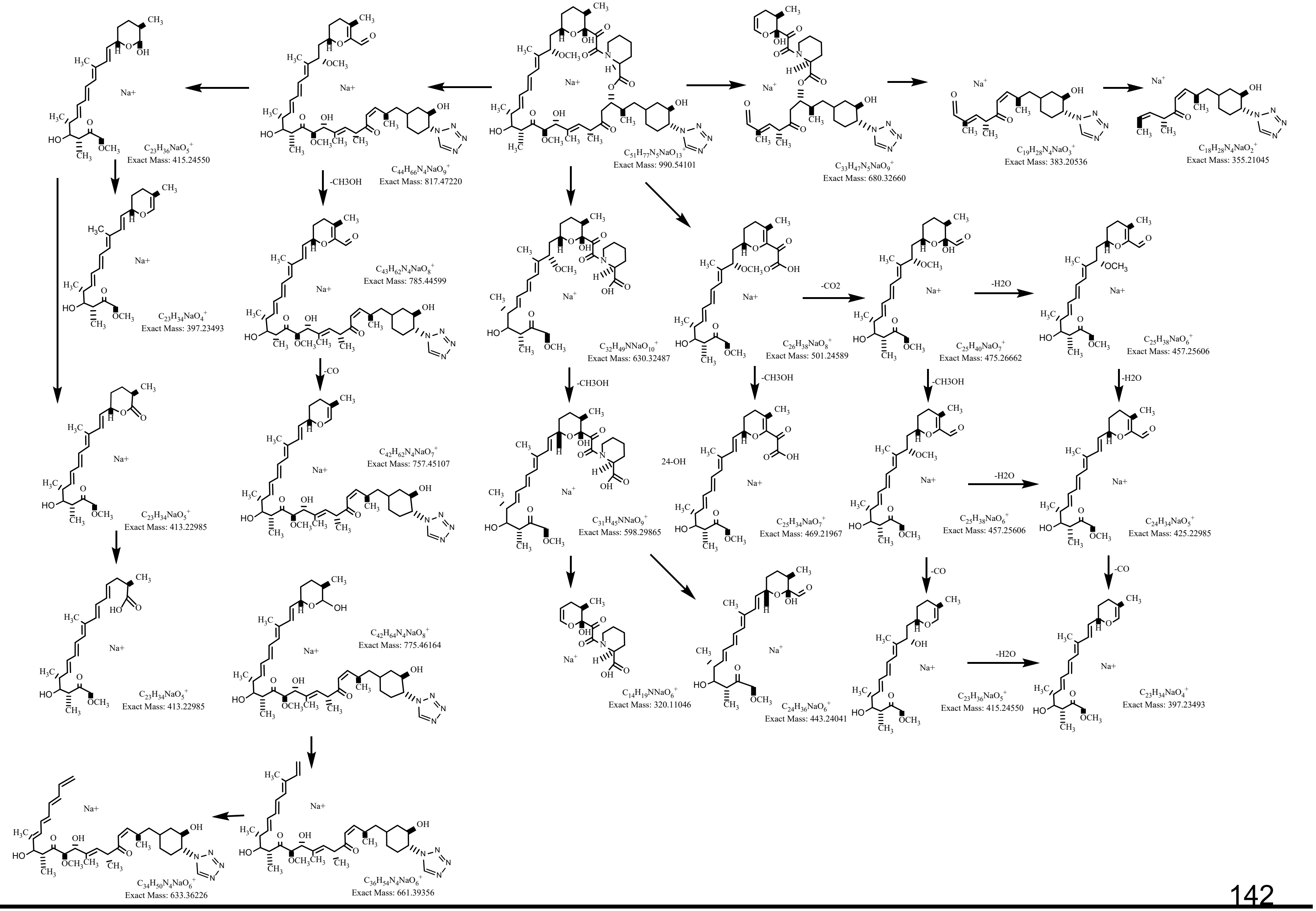

Characteristic Fragments of 39-O-desmethyl, 24-OH Zotarolimus

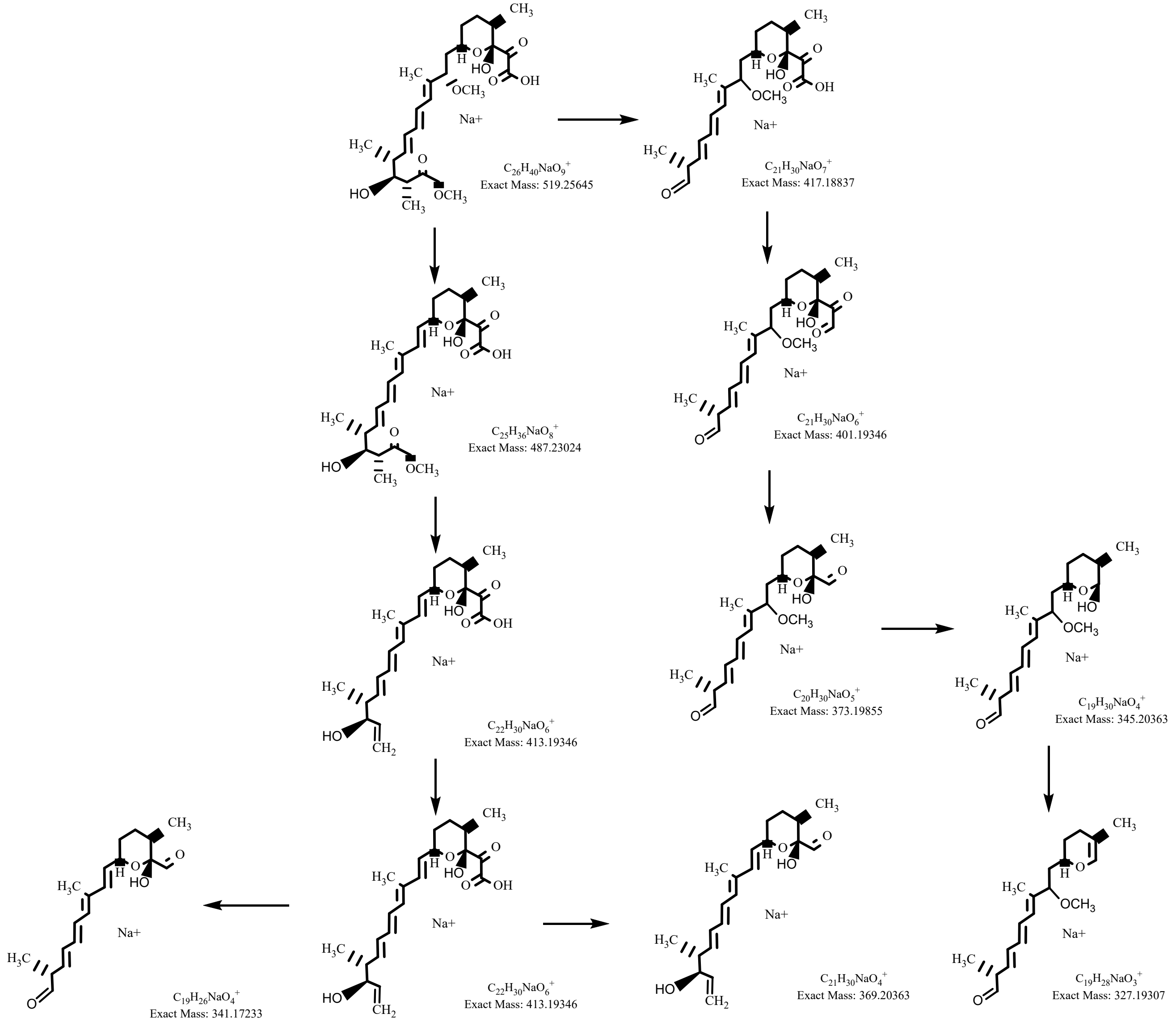

Δppm of 39-O-Desmethyl, 24-Hydroxy Zotarolimus

Theoretical mass	Measured mass	Δppm
320.11046	320.10999	1.5
355.21045	355.21005	1.1
369.22610	369.22577	0.9
397.22101	397.22069	0.8
425.22985	425.22899	2.0
443.24041	443.23993	1.1
457.25606	457.25544	1.4
469.21967	469.21838	2.7
475.26662	475.26577	1.8
501.24589	501.24520	1.4
598.29865	598.29811	0.9
630.32487	630.32469	0.3
661.39356	661.39303	0.8
757.45107	757.45044	0.8
990.54101	990.54036	0.7

Characteristic Fragments of 39-O-desmethyl, 24-OH Zotarolimus (Water Loss)

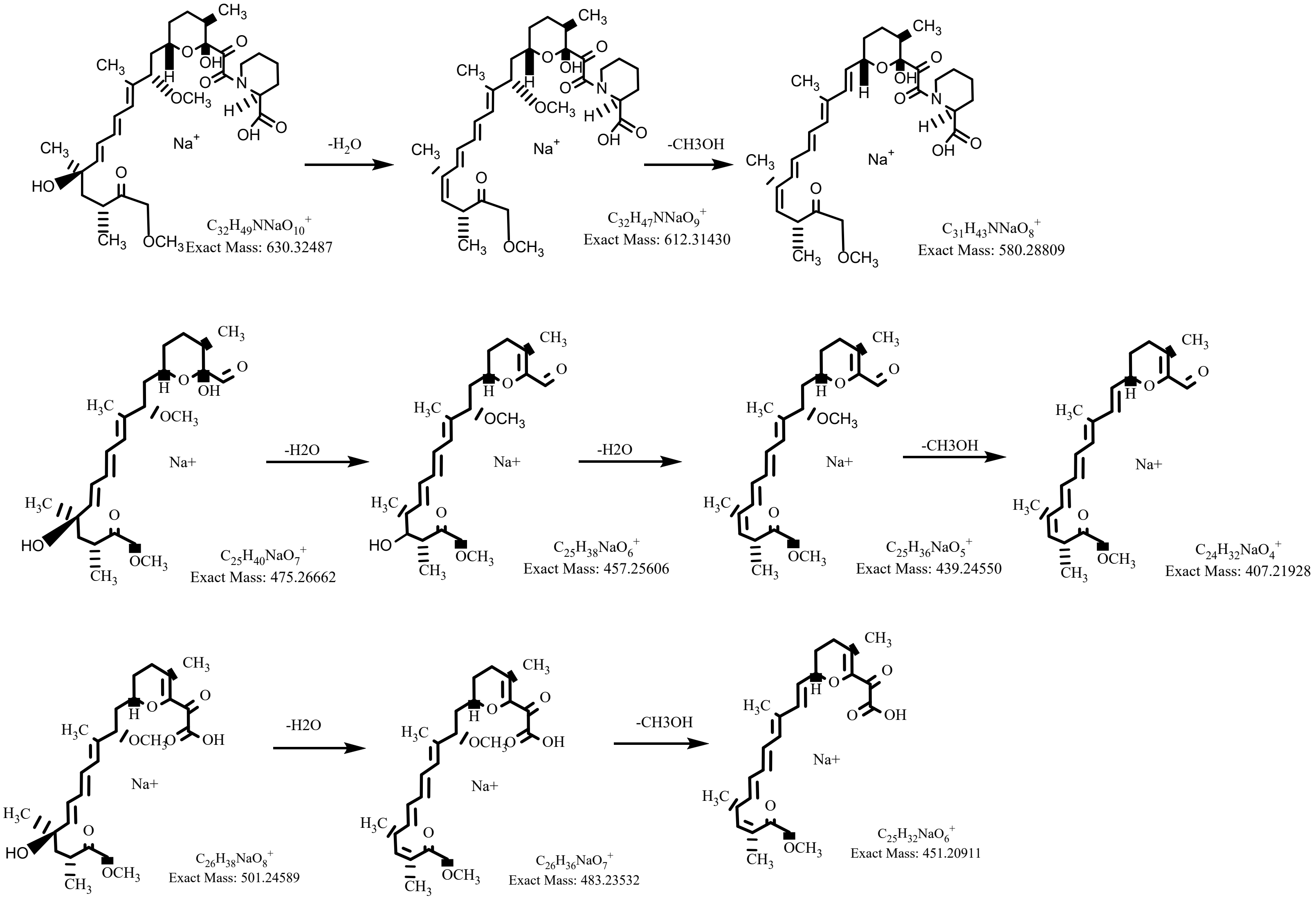

Structural Confirmation of 39-O-Desmethyl, 24-Hydroxy Zotarolimus

Zotarolimus Fragments	39-O-desmethyl, 23/24-hydroxy Zotarolimus Fragments	Comments	
320.11046	320.10999	no hydroxylation, no desmethylation	Rules out OH-pipiredine, 11, 12, and 14-OH
369.22610	355.21005	desmethylation	Confirms 39-O-desmethyl
381.24002	397.22045	hydroxylation	11, 12, 14, 23, 24,45, or 46-OH present
409.23493	425.22899	hydroxylation	11, 12, 14, 23, 24,45, or 46-OH present
427.2455	443.23993	hydroxylation	11, 12, 14, 23, 24,45, or 46-OH present
459.27171	475.26577	hydroxylation	11, 12, 14, 23, 24, 45, or 45/46-OH present/ Rules out 16- and 27-O-desmethyl
485.25097	501.24520	hydroxylation	11, 12, 14, 23, 24, 45, or 45/46-OH present/ Rules out 16- and 27-O-desmethyl
582.30374	598.29811	hydroxylation	11, 12, 14, 23, 24, 45, or 45/46-OH present/ Rules out 27-O-desmethyl
614.32995	630.32469	hydroxylation	11, 12, 14, 23, 24, 45, or 45/46-OH present/ Rules out 16- and 27-O-desmethyl
659.41429	661.39303	hydroxylation	Rules out 11, 12, 14-OH, and OH-piperidine
755.47181	757.45044	hydroxylation, desmethylation	11, 12, 14, 23, 24,45, or 45/46-OH present/ 16- 27-. or 39-O-desmethyl
988.56174	990.54036	demethylation	
Characteristic Fragments		327.19307/ 345.20363/ 401.19346/ 417.18837	451 due to methanol loss of 501 Rules out 16-O-desmethyl zotarolimus<br>425 due to methanol loss of 457 Rules out 16-O-desmethyl zotarolimus
Determinant patterns			Additional water loss for 23/24-OH

39-O-Desmethyl, 25-Hydroxy Zotarolimus, m/z = 990.54101

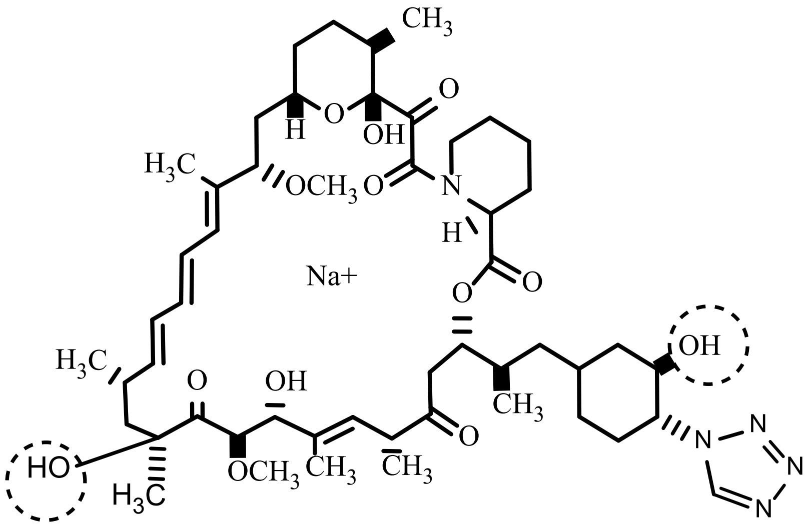

$C_{51}H_{77}N_5NaO_{13}^{+}$
Exact Mass: 990.54101

39-O-desmethyl, 25-hydroxy zotarolimus

39-O-Desmethyl, 25-Hydroxy Zotarolimus Metabolites, m/z = 990.54101

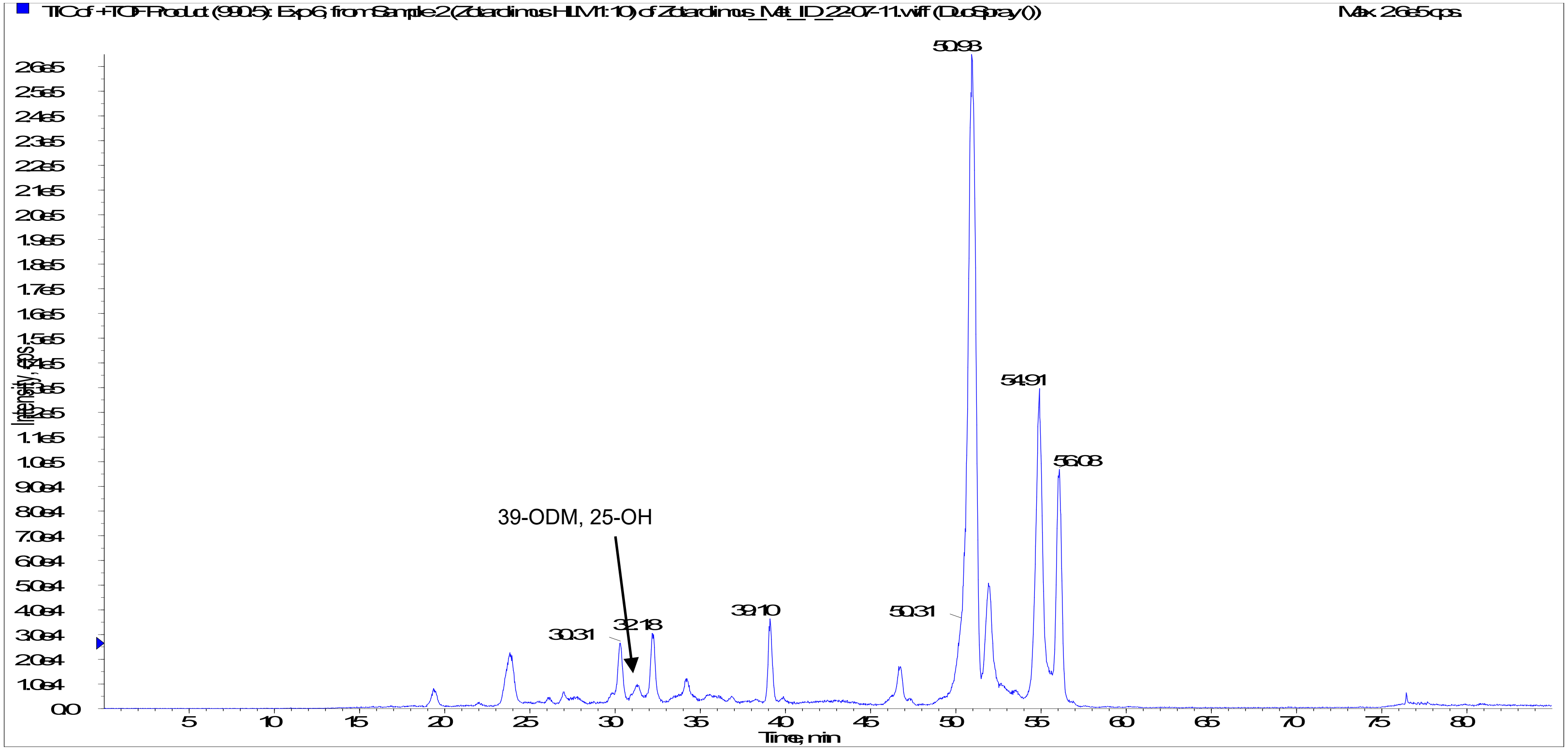

39-O-Desmethyl, 25-Hydroxy Zotarolimus m/z = 990.54101 Extracted Ion Chromatogram (EIC)

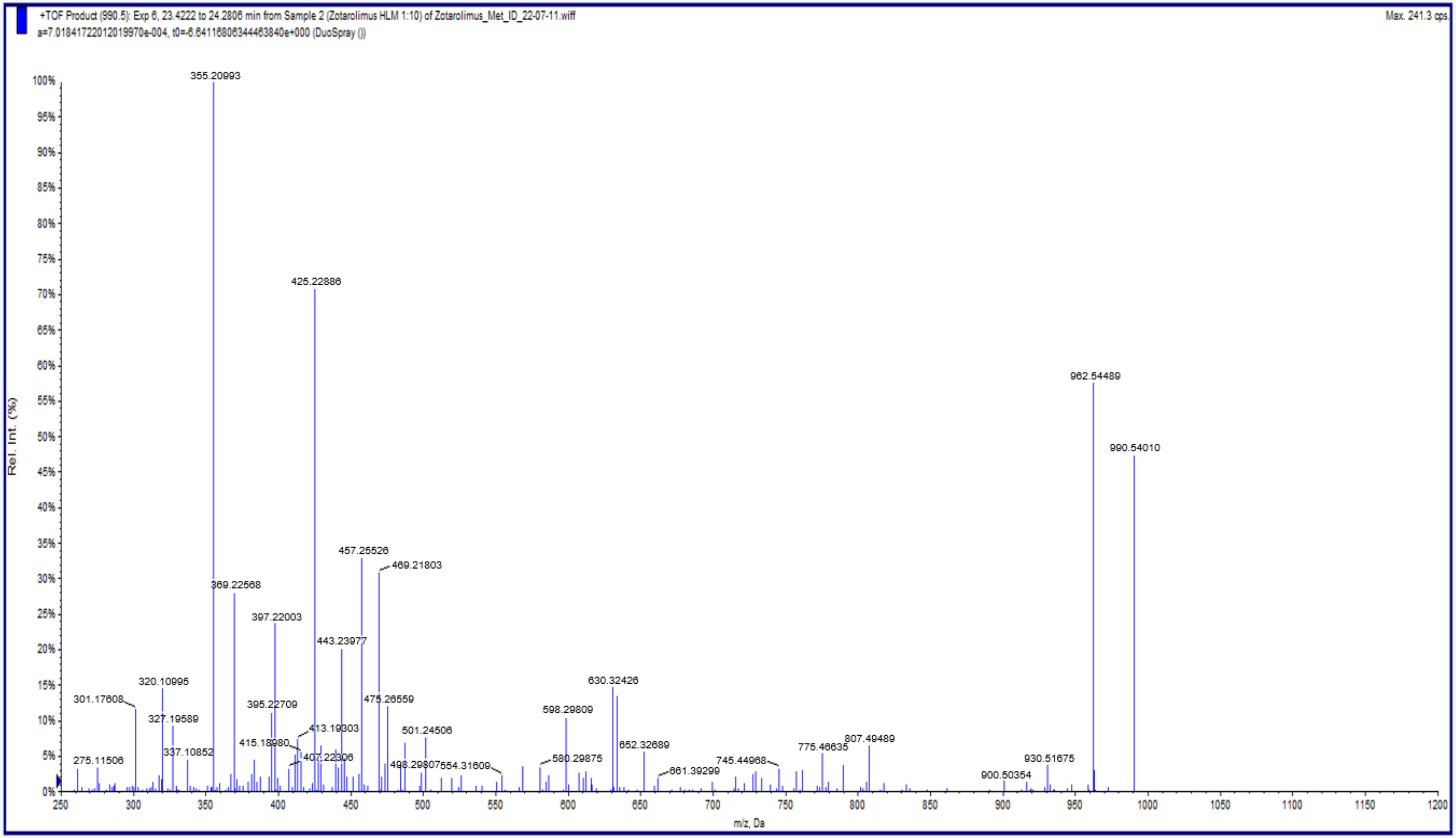

39-O-Desmethyl, 25-Hydroxy Zotarolimus Fragmentation Pattern

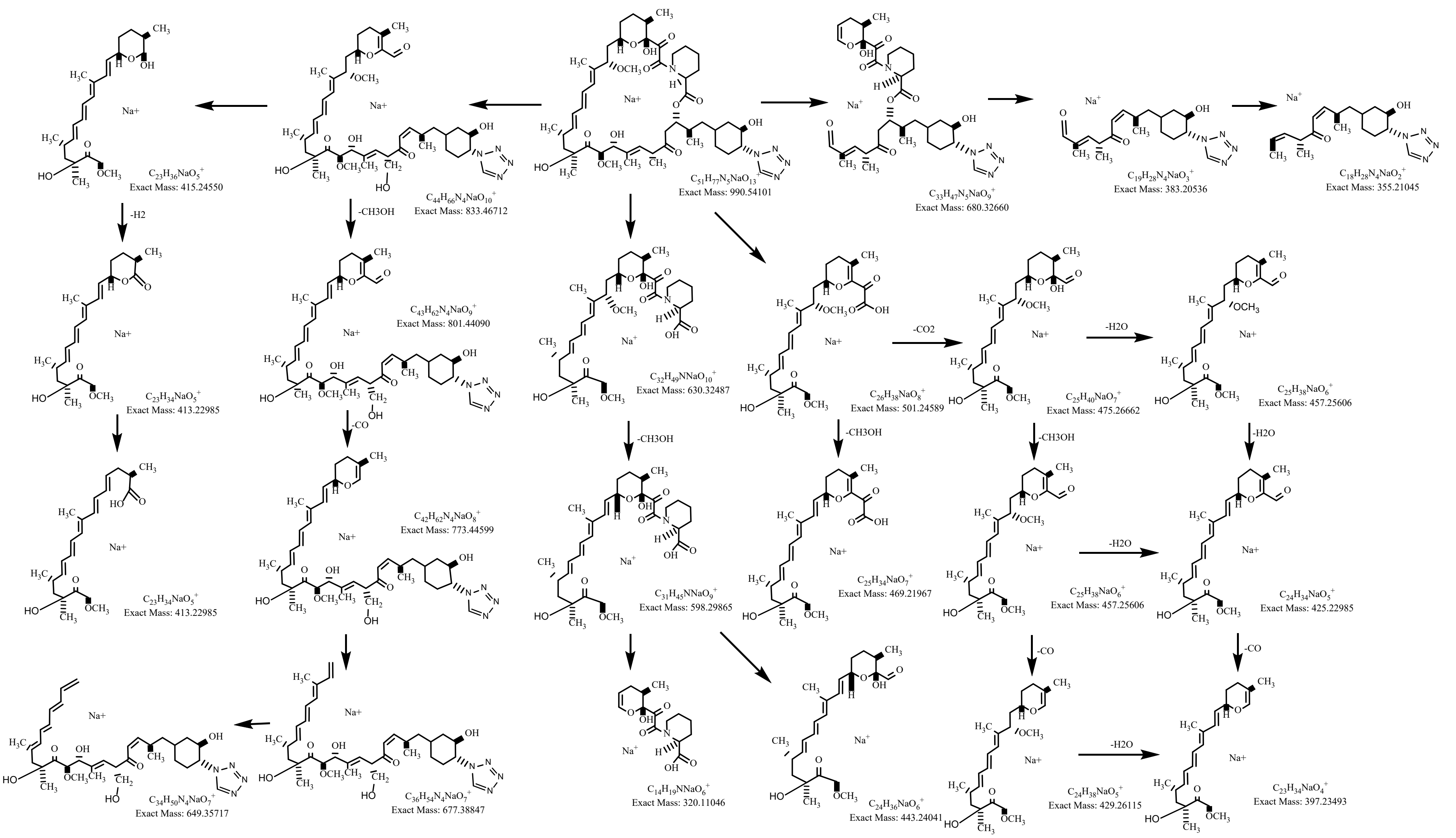

Δppm of 39-O-Desmethyl, 25-Hydroxy Zotarolimus

Fragment No.	Theoretical mass	Measured mass	Δppm
1	320.11046	320.11003	1.3
2	355.21045	355.21022	0.6
3	369.22610	369.22568	1.1
4	383.20536	383.20489	1.2
5	425.22985	425.22933	1.2
6	443.24041	443.24006	0.8
7	457.25606	457.25584	0.5
8	469.21967	469.21919	1.0
9	475.26662	475.26630	0.7
10	501.24589	501.24554	0.7
11	598.29865	598.29833	0.5
12	630.32487	630.32444	0.7
13	661.39356	661.39288	1.0
14	757.45107	757.45044	0.8
15	990.54101	990.54027	0.7

Characteristic Fragments of 39-O-desmethyl, 25-OH Zotarolimus (Water Loss)

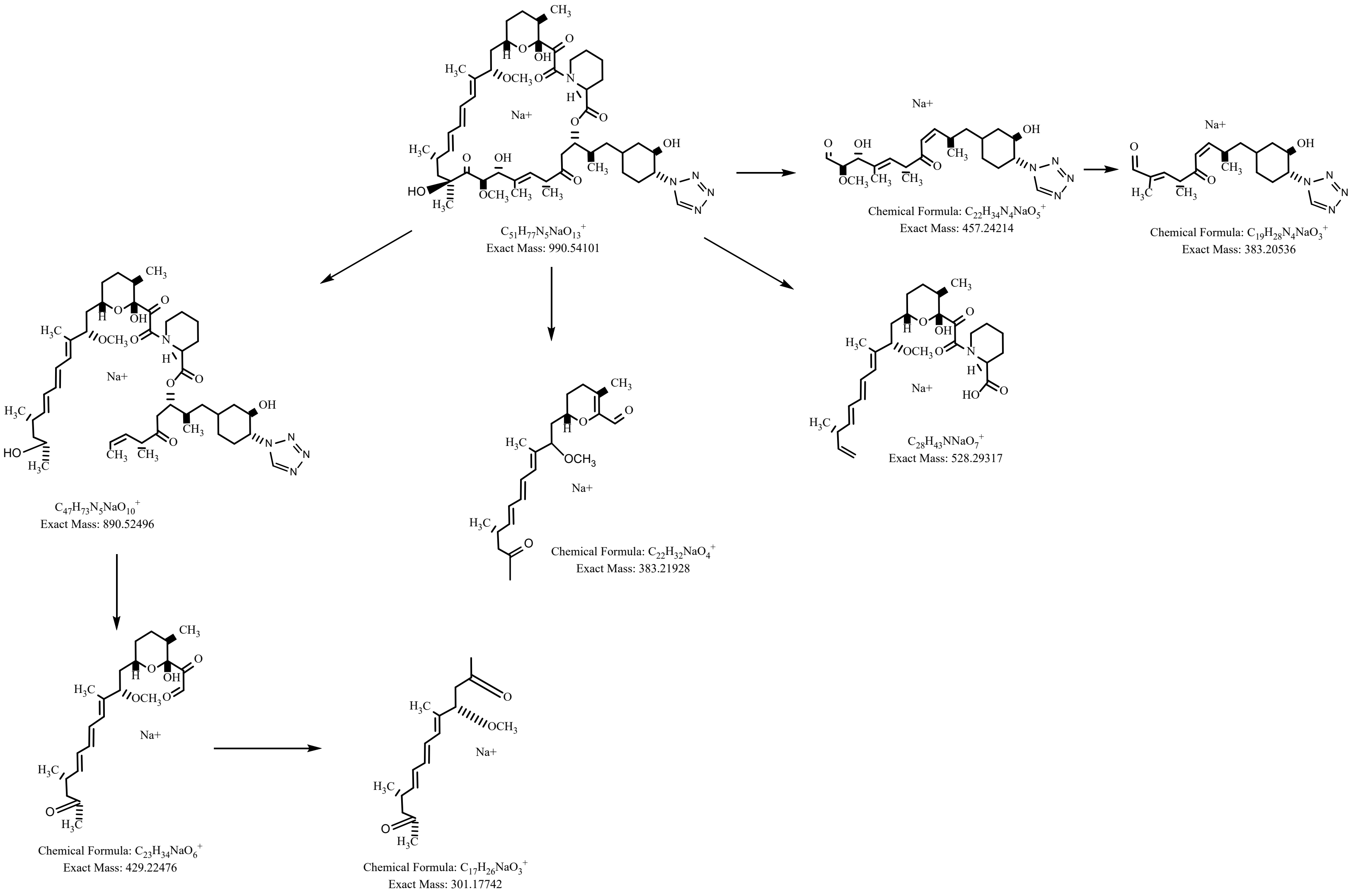

Structural Confirmation of 39-O-Desmethyl, 25-Hydroxy Zotarolimus

Zotarolimus Fragments	39-O-desmethyl, 25-hydroxy Zotarolimus Fragments	Comments	
320.11046	320.11003	no hydroxylation, no desmethylation	Rules out OH-pipiredine, 11, 12, and 14-OH
369.22610	355.21022	desmethylation	Confirms 39-O-desmethyl/ Rules out 47-, 48-, and 49-OH
NA	383.21883	NA	Rules out 16-O-desmethyl
409.23493	425.22933	hydroxylation	11, 12, 14, 23, 24, 45, or 46-OH present
427.2455	443.24006	hydroxylation	11, 12, 14, 23, 24, 45, or 46-OH present/ Rules out 27-O-desmethyl
459.27171	475.26630	hydroxylation	11, 12, 14, 23, 24,45, or 45/46-OH present/ Rules out 16- or 27-O-desmethyl
485.25097	501.24554	hydroxylation	11, 12, 14, 23, 24,45, or 45/46-OH present/ Rules out 16- or 27-O-desmethyl
582.30374	598.29833	hydroxylation	11, 12, 14, 23, 24, 45, or 46-OH present/ 16- or 27-O-desmethyl
614.32995	630.32444	hydroxylation	11, 12, 14, 23, 24, 45, or 46-OH present/ 16- or 27-O-desmethyl
659.41429	661.39288	hydroxylation	Rules out 11, 12, 14-OH, and OH-piperidine
755.47181	757.45044	hydroxylation, desmethylation	11, 12, 14, 23, 24,45, or 45/46-OH present/ 16- 27-. or 39-O-desmethyl
988.56174	990.54027	demethylation	
Characteristic Fragments	301.17742<br>383.21883<br>429.22476<br>528.29317		Characteristic Fragments: 301, 383, 429, and 528
Determinant patterns			water loss instead of methanol loss

39-O-Desmethyl, 11-Hydroxy Zotarolimus, m/z = 990.54101

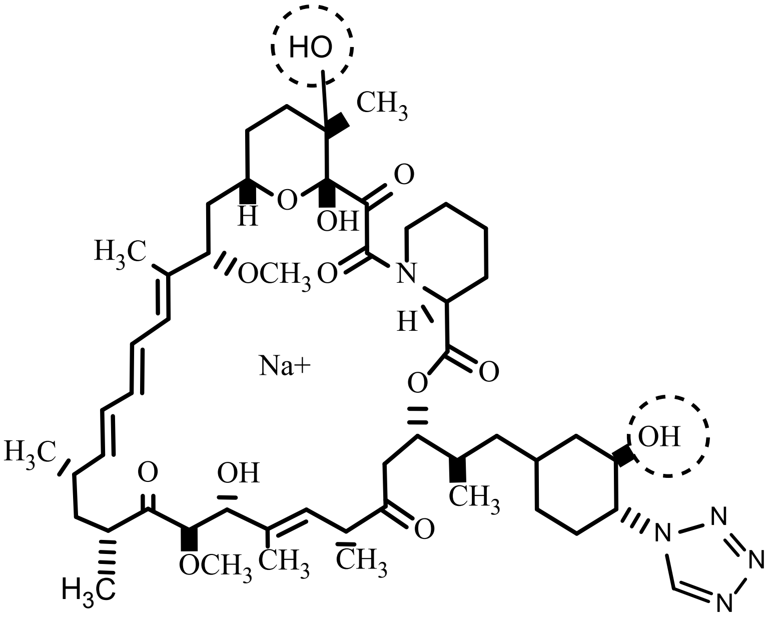

$C_{51}H_{77}N_5NaO_{13}{}^{+}$
Exact Mass: 990.54101

39-O-Desmethyl, 11-Hydroxy Zotarolimus Metabolites, m/z = 990.54101

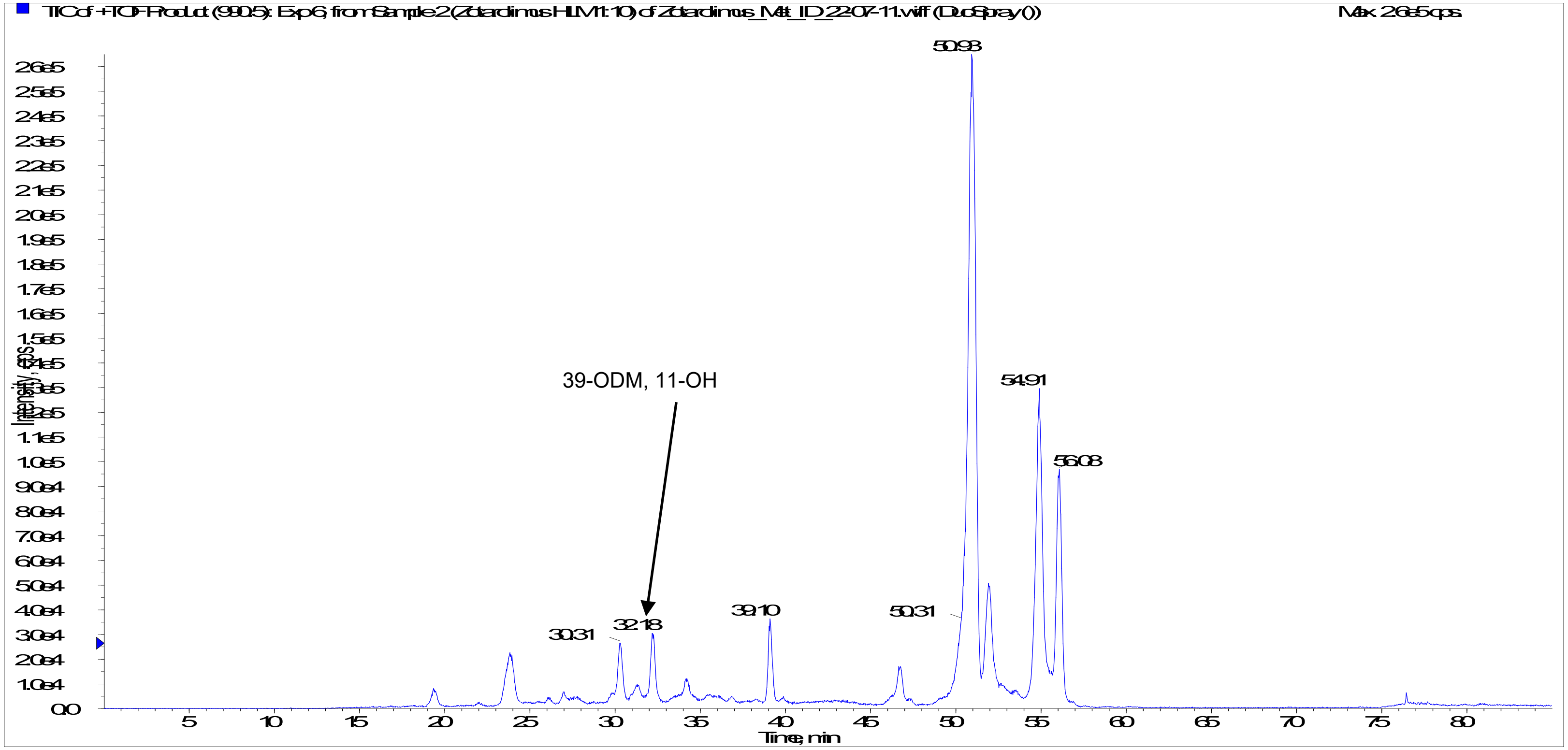

39-O-Desmethyl, 11-Hydroxy Zotarolimus
Extracted Ion Chromatogram (EIC)

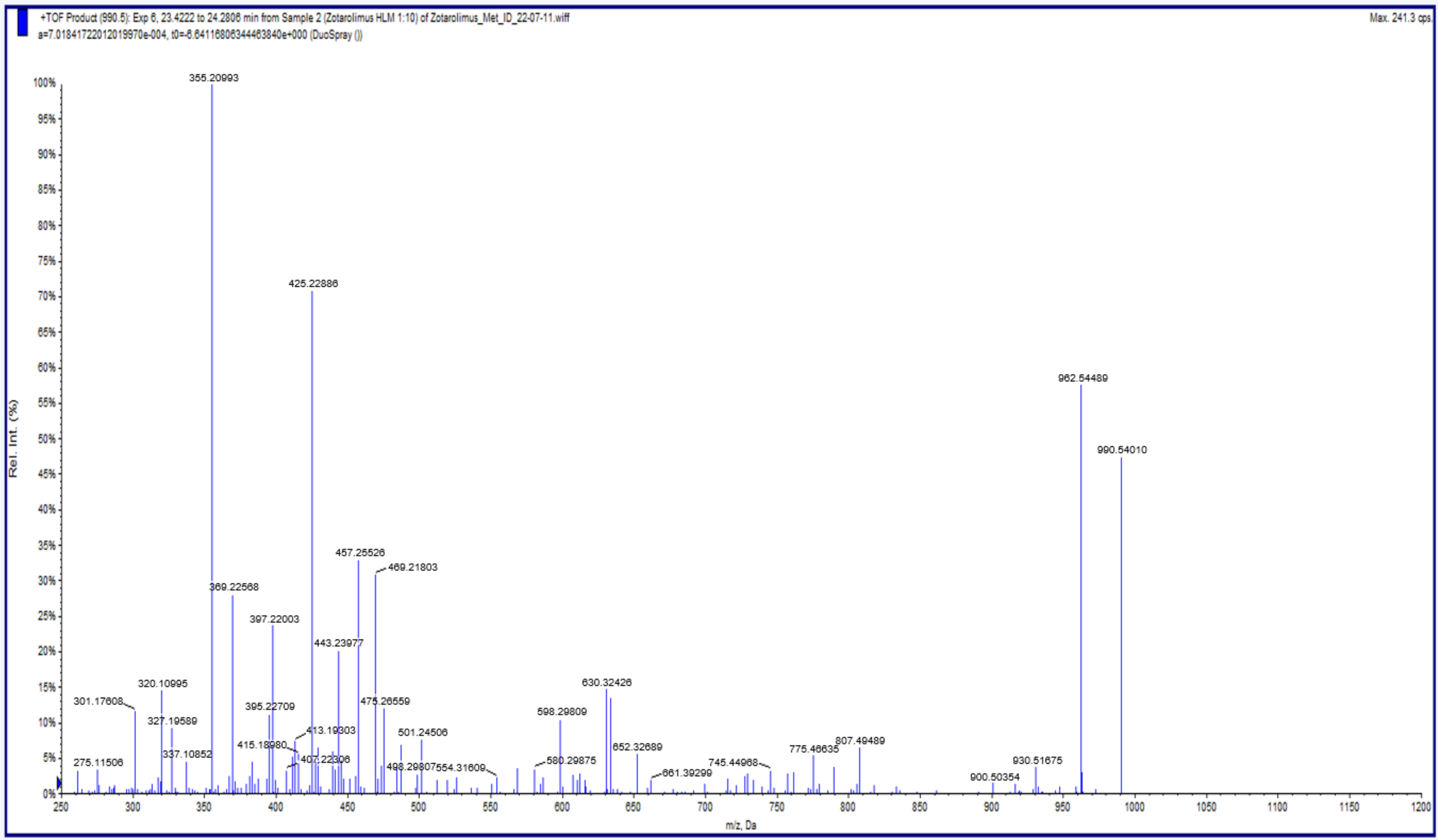

Δppm of 39-O-Desmethyl, 11-Hydroxy Zotarolimus

Fragment No.	Theoretical mass	Measured mass	Δppm
1	336.10537	336.10479	1.7
2	355.21045	355.20958	2.4
3	369.24002	369.2393	1.9
4	383.20536	383.20488	1.3
5	401.26623	401.26583	1.0
6	425.22985	425.22901	2.0
7	443.24041	443.2399	1.2
8	469.21967	469.21905	1.3
9	501.24589	501.24539	1.0
10	733.45107	733.45009	1.3
11	761.44599	761.44409	2.5
12	789.47729	789.47650	1.0
13	807.48785	807.48665	1.5
14	990.54101	990.54001	1.0

39-O-Desmethyl, 11-Hydroxy Zotarolimus Fragmentation Pattern

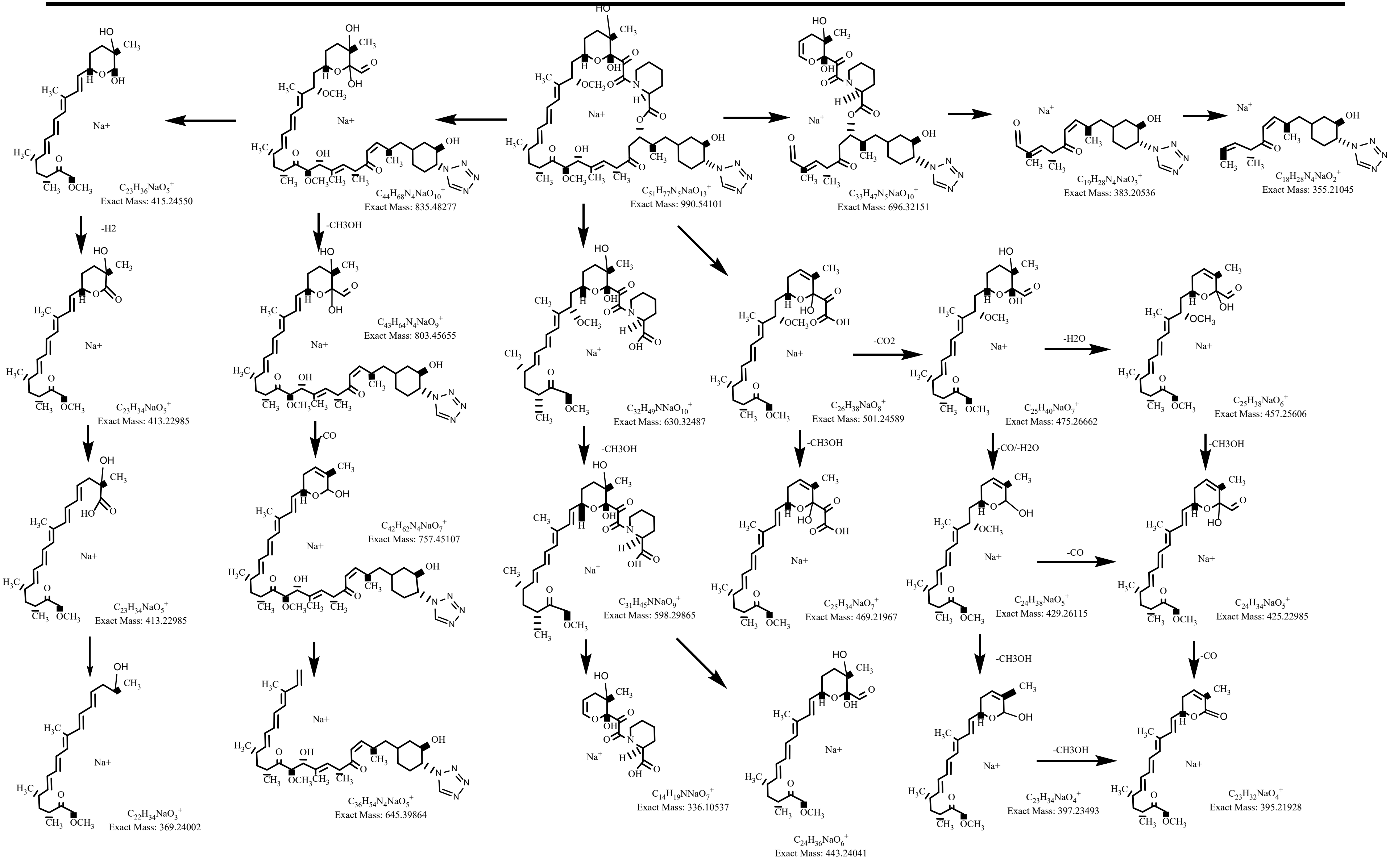

39-O-Desmethyl, 11-Hydroxy Zotarolimus Characteristic Fragmentation Pattern

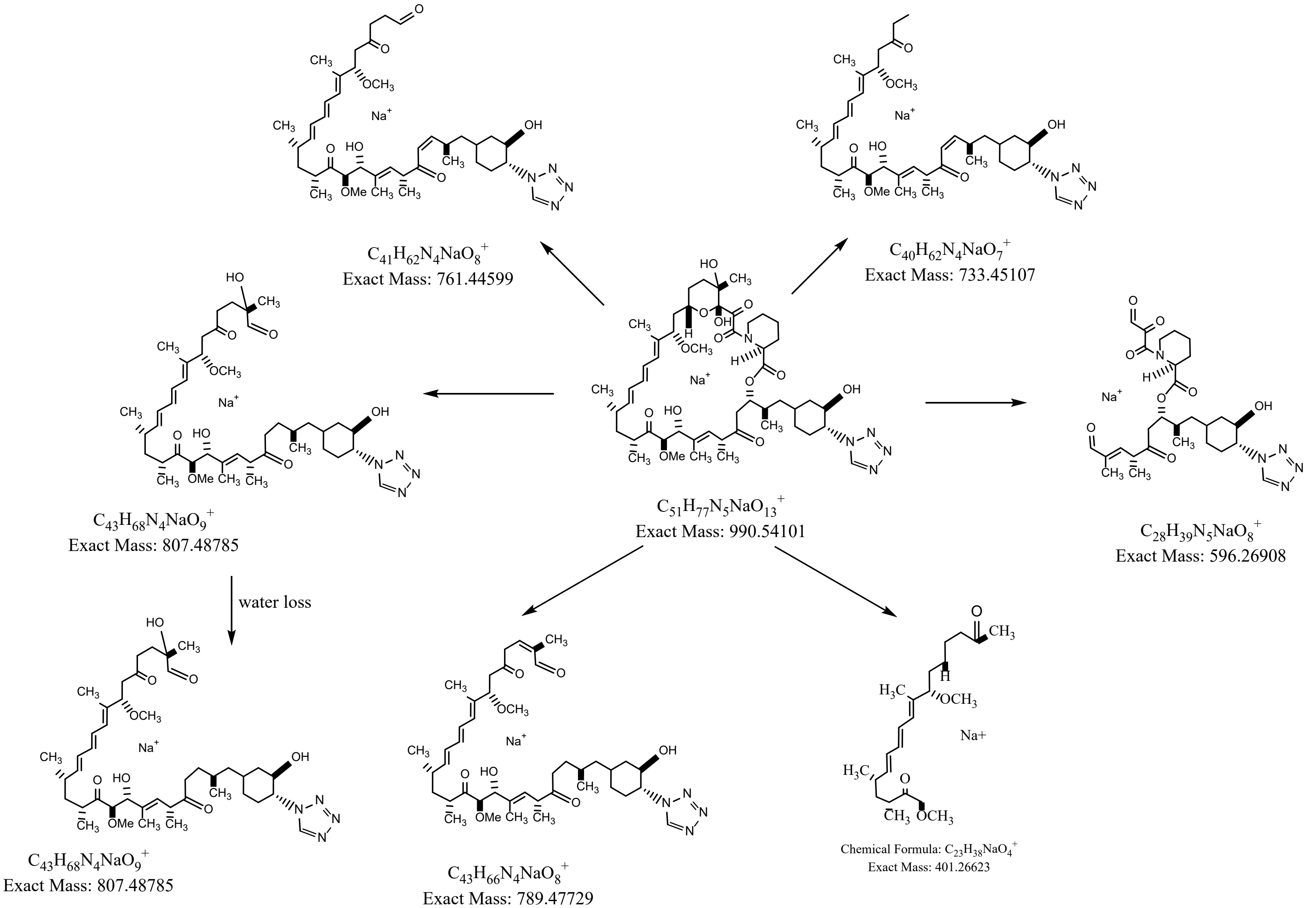

Structural Confirmation of 39-O-Desmethyl, 11-Hydroxy Zotarolimus

Zotarolimus Fragments	39-O-desmethyl, 11-hydroxy-Zotarolimus Fragments	Comments	
320.11046	336.10479	hydroxylation	11, 12, 14, or piperidine-OH present
NA	369.2393	Specific fragment	confirms 11-OH
397.22101	383.20488	desmethylation	confirms 39-O-desmethyl/ Rules out 47, 48, and 49-OH
409.23493	425.22901	hydroxylation	11, 12, 14, 23, 24, 25, 45, or 46-OH present/ 16- or 27-O-desmethyl present
427.2455	443.2399	hydroxylation	11, 12, 14, 23, 24, 25, 45, or 46-OH present/ 16- or 27-O-desmethyl present
453.22476	469.21905	hydroxylation	11, 12, 14, 23, 24, 25, 45, or 46-OH present/ 16- or 27-O-desmethyl present
485.25097	501.24539	hydroxylation	11, 12, 14, 23, 24, 25, 45, or 46-OH present/ Rules out 16- or 27-O-desmethyl
614.32995	630.32444	hydroxylation	11, 12, 14, 23, 24, 25, 45, or 46-OH present/ Rules out 16- or 27-O-desmethyl
659.41429	645.39787	desmethylation	Rules out 23, 24, 25, 45, 46, 47, 487, 49-OH/ 27- or 39-ODM present
988.56174	990.54001	hydroxylation, desmethylation	
Characteristic Fragments			369/ 383/ 401/ 596/ 733/ 761/ 789
Determinant patterns			

16-O-Desmethyl, Hydroxy-Piperidine Zotarolimus (I), m/z = 990.54101

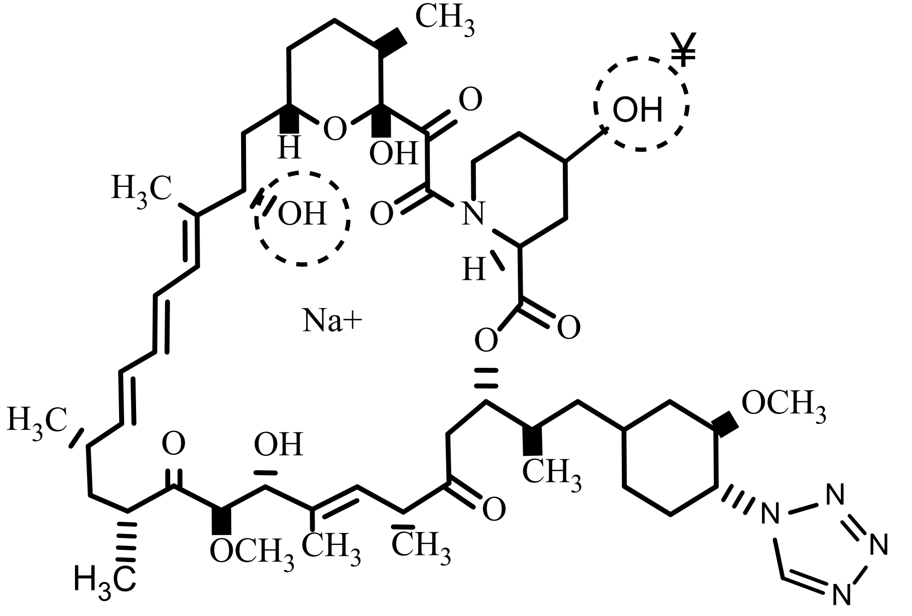

$C_{51}H_{77}N_5NaO_{13}^+$
Exact Mass: 990.54101

¥ Only one of five possible hydroxyl group in piperidine ring is shown.

16-O-Desmethyl, Hydroxy-Piperidine Zotarolimus (I) Metabolites, m/z = 990.54101

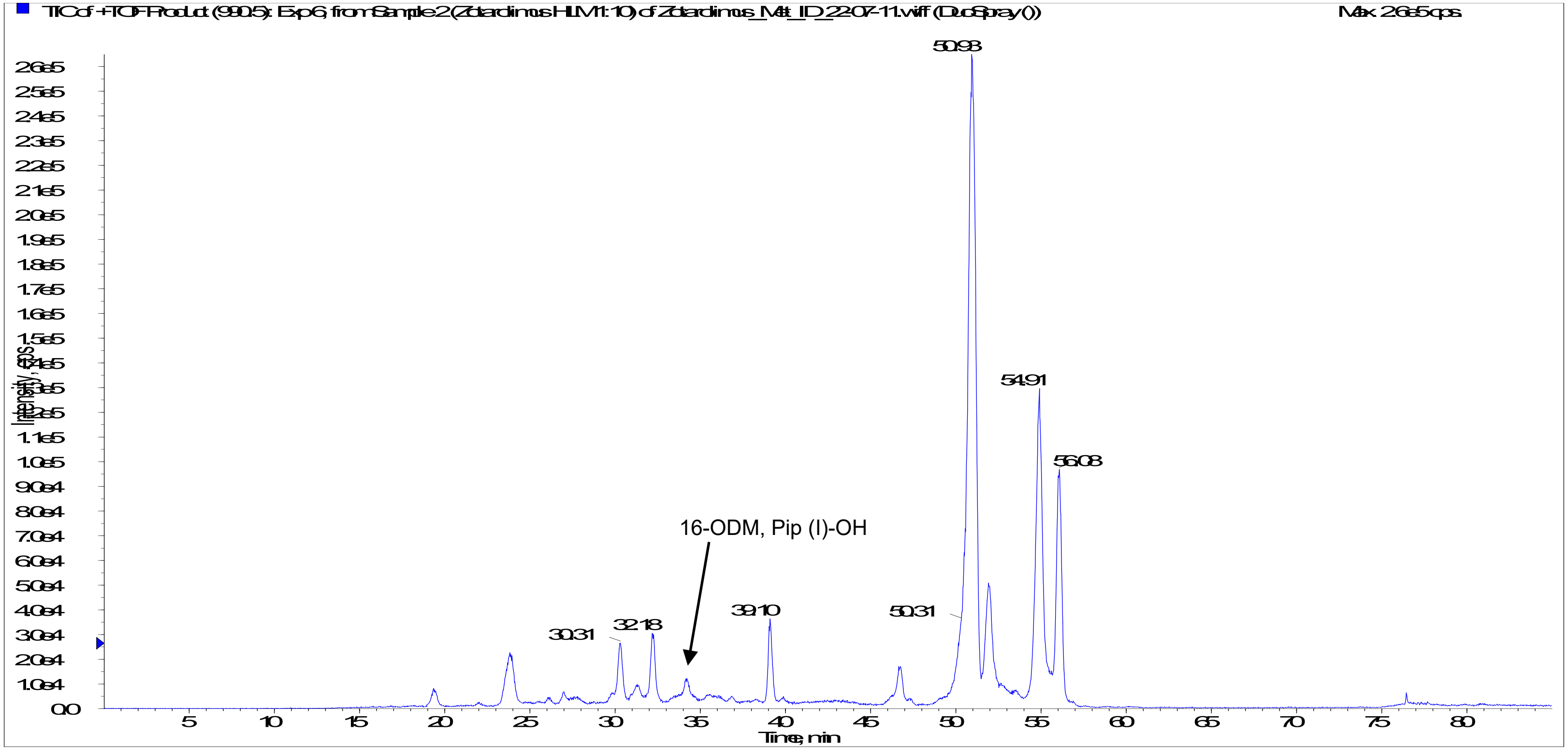

16-O-Desmethyl, Hydroxy-Piperidine Zotarolimus (I)
Extracted Ion Chromatogram (EIC)

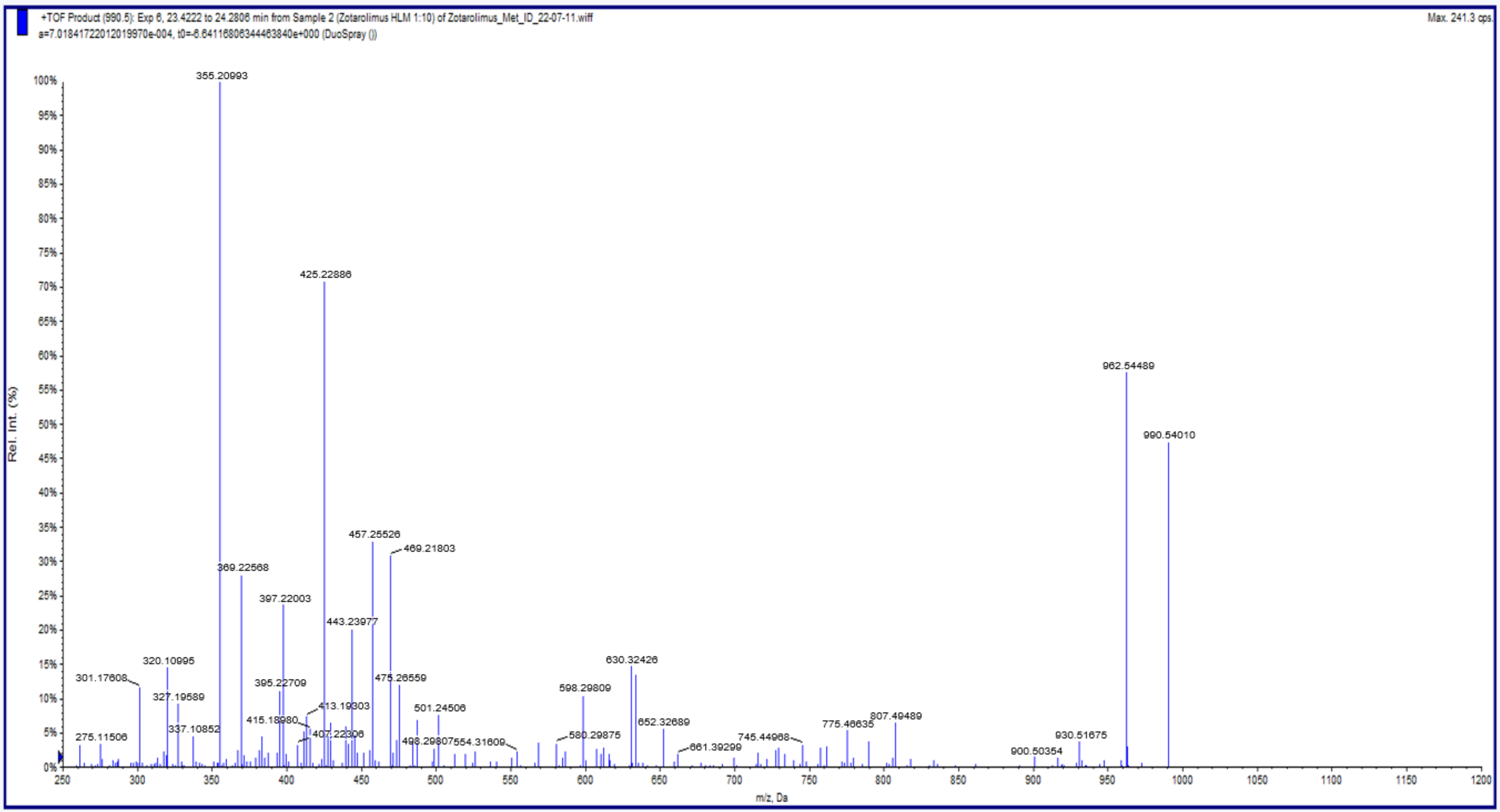

16-O-Desmethyl, Hydroxy-Piperidine Zotarolimus (I) Fragmentation Pattern

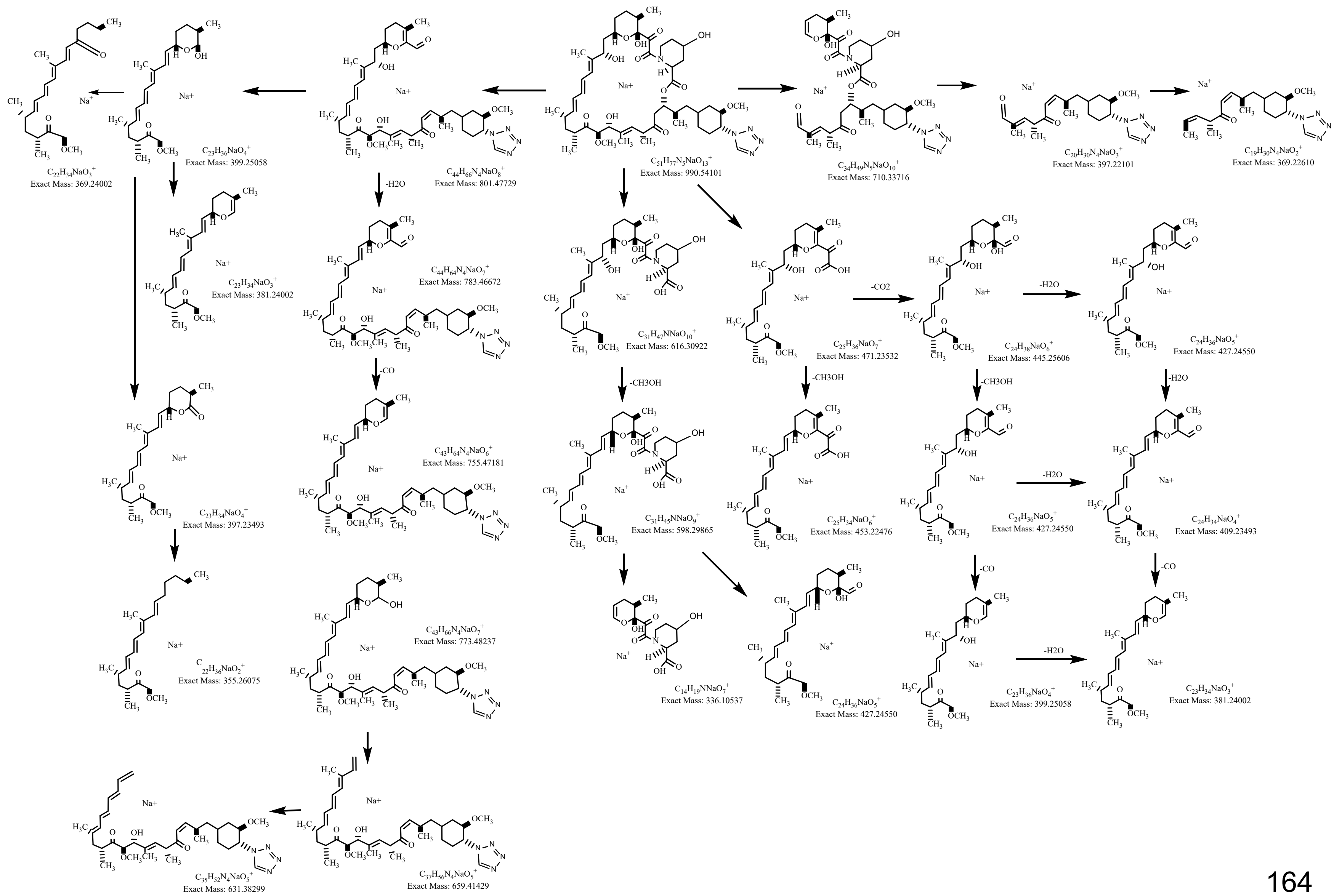

Δppm of 16-O-Desmethyl, Hydroxy-piperidine Zotarolimus (I)

Theoretical mass	Measured mass	Δppm
336.10537	336.10439	2.9
355.26075	355.25973	2.9
381.24002	381.23887	3.0
397.23493	397.23448	1.1
409.23493	409.23415	1.9
427.24550	427.24506	1.0
453.22476	453.22423	1.2
471.23532	471.23403	2.7
598.29865	598.29813	0.9
616.30922	616.30817	1.7
631.38299	631.38206	1.5
990.54101	990.54006	1.0

Structural Confirmation of 16-O-Desmethyl, Hydroxy-Piperidine Zotarolimus (I)

Zotarolimus Fragments	16-O-desmethyl, hydroxy-piperidine Zotarolimus Fragments	Comments	
320.11046	336.10439	hydroxylation	11, 12, 14, or piperidine-OH present
381.24002	381.23887	no hydroxylation, no desmethylation	11, 12, 14, 23, 24, 25, 45, or 46-OH present/ confirms 16-O-desmethyl
397.23493	397.23448	no hydroxylation, no desmethylation	Rules out 47, 48, and 49-OH/ Rules out 39-O-desmethyl
409.23493	409.23415	no hydroxylation, no desmethylation	11, 12, 14, 23, 24, 25, 45, or 46-OH present/ confirms 16-O-desmethyl
441.26115	427.24506	demethylation	11, 12, 14, 23, 24, 25, 45, or 46-OH present/ 16- or 27-O-desmethyl present
453.22476	453.22423	no hydroxylation, no desmethylation	11, 12, 14, 23, 24,45, or 46-OH present/ confirms 16-O-desmethyl
485.25097	471.23403	hydroxylation	11, 12, 14, 23, 24, 25, 45, or 46-OH present/ 16- or 27-O-desmethyl present
582.30374	598.29813	hydroxylation	11, 12, 14, 23, 24, 45, 46, or Piperidine-OH present/ confirms 16-O-desmethyl
614.32995	616.30817	hydroxylation, desmethylation	11, 12, 14, 23, 24,45, 46, or Piperidine-OH present/16- or 27-O-desmethyl
659.41429	ND	hydroxylation	
631.38299	631.38206	no hydroxylation, no desmethylation	23, 24, 25, 45, 46, 47, 48 , or 49-OH present/ Rules out 39-O-desmethyl
988.56174	990.54027	demethylation	
Characteristic Fragments			336
Determinant patterns			water loss instead of methanol loss at position C16

27-O-Desmethyl, 45/46-Hydroxy Zotarolimus (I), m/z = 990.54101

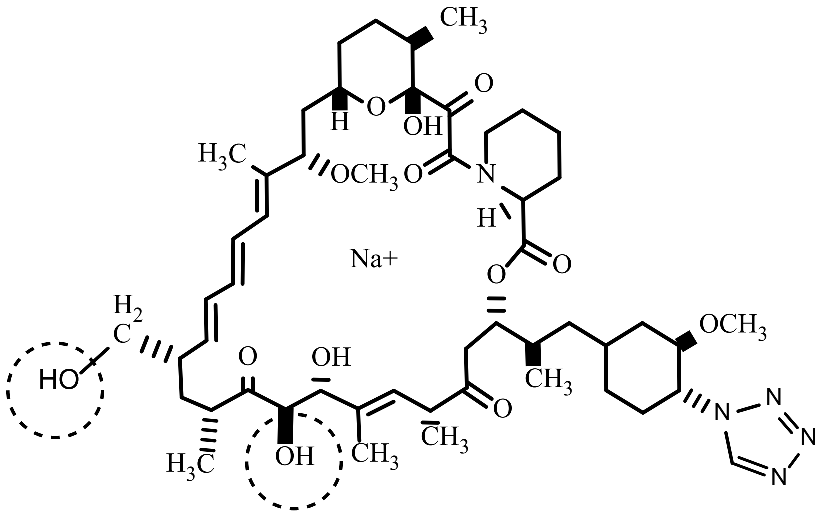

$C_{51}H_{77}N_5NaO_{13}^{+}$
Exact Mass: 990.54101

27-O-desmethyl, 45-hydroxy sirolimus

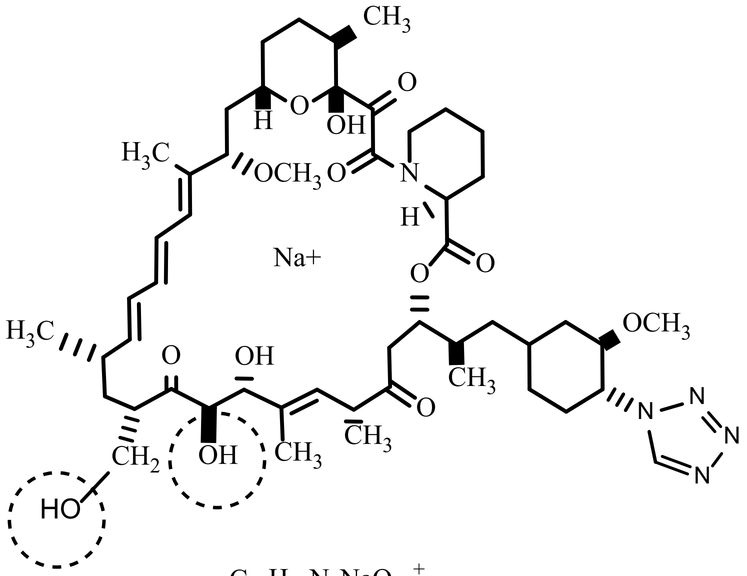

$C_{51}H_{75}N_5NaO_{13}^{+}$
Exact Mass: 988.52536

27-O-desmethyl, 46-hydroxy sirolimus

27-O-Desmethyl, 45/46-Hydroxy Zotarolimus Metabolites, m/z = 990.54101

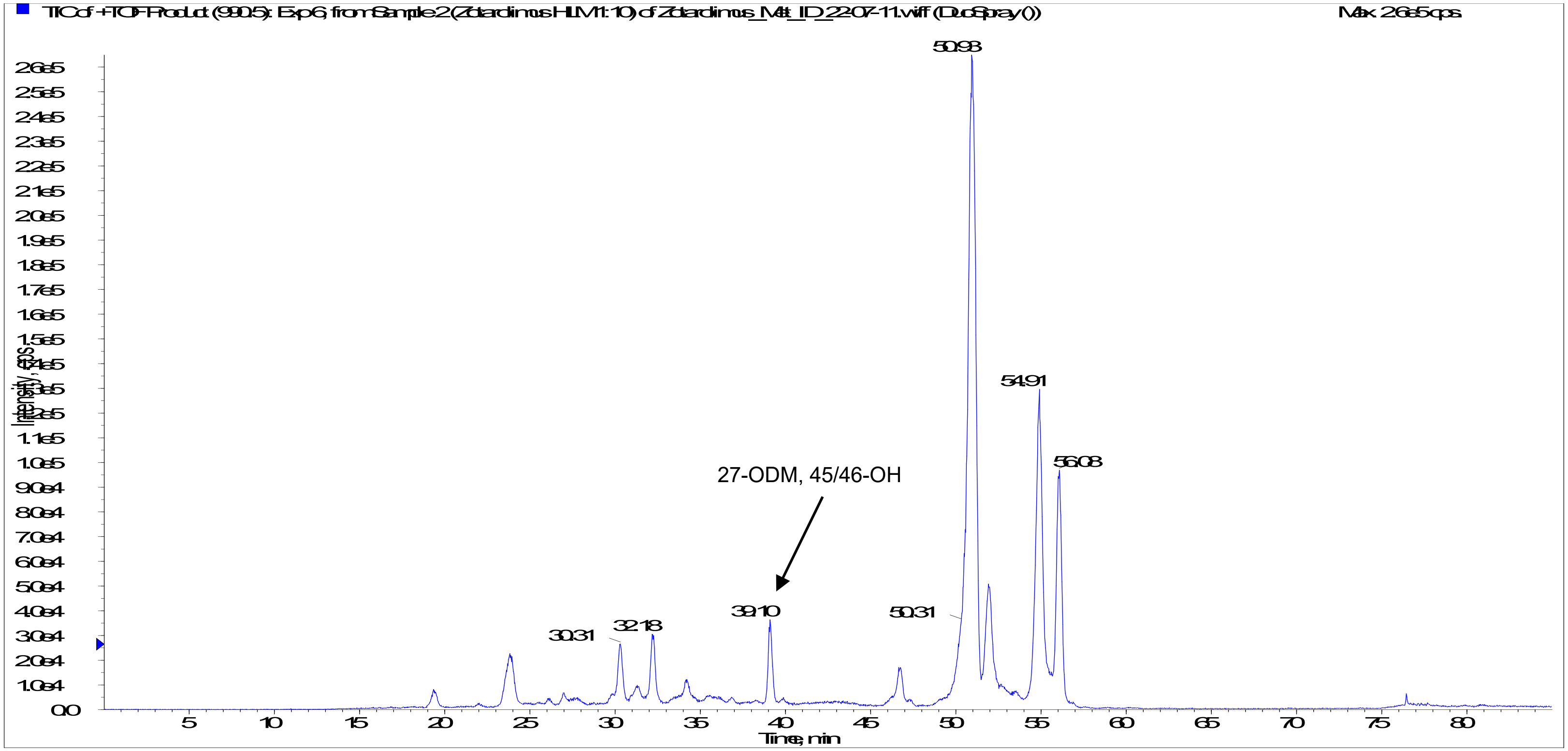

27-O-Desmethyl, 45/46-Hydroxy Zotarolimus
Extracted Ion Chromatogram (EIC)

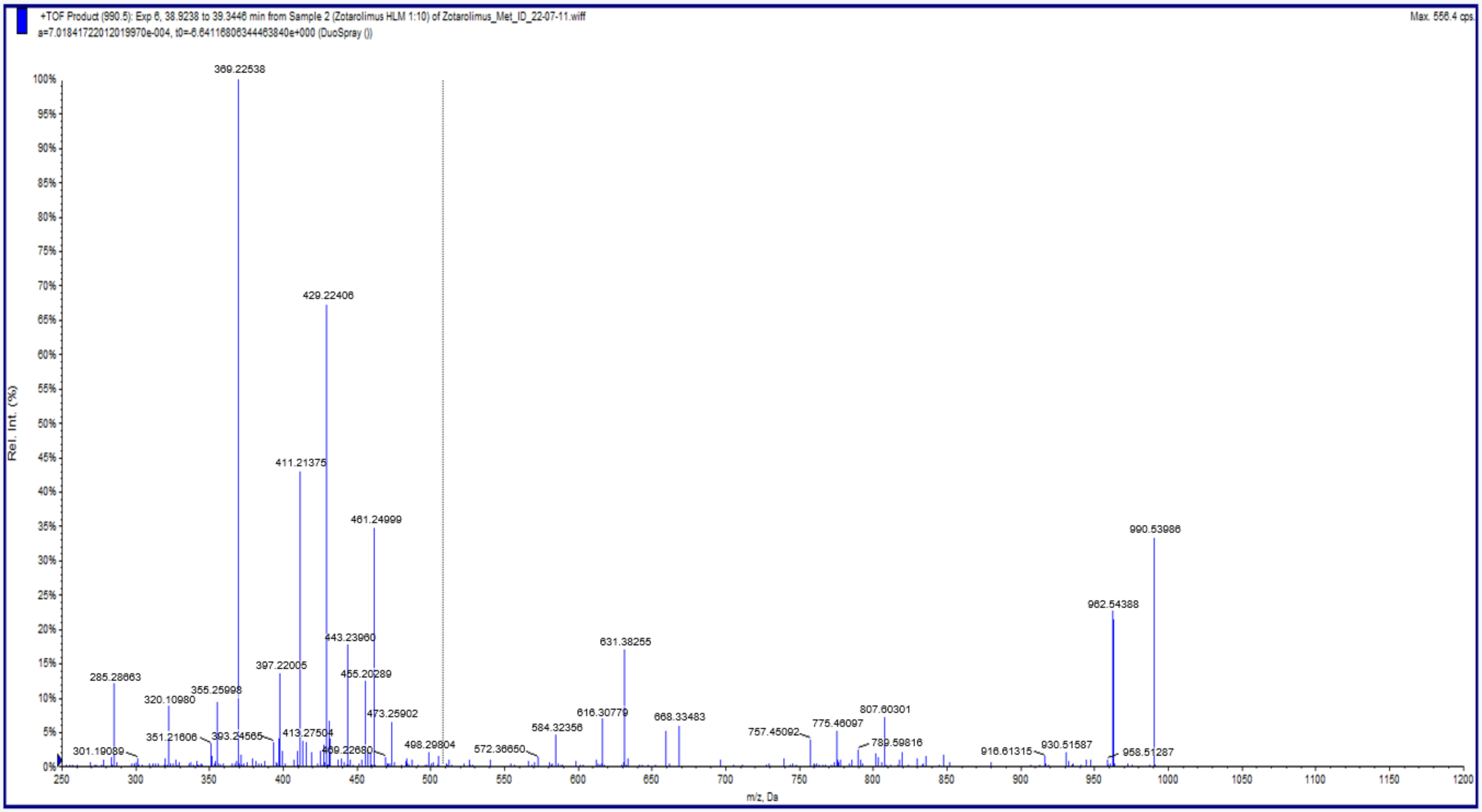

Δppm of 16-O-Desmethyl, 45/46-Hydroxy Zotarolimus

Fragment No.	Theoretical mass	Measured mass	Δppm
1	320.11046	320.1098	2.1
2	355.26075	355.25998	2.2
3	369.2261	369.22538	2.0
4	397.22101	397.22005	2.4
5	411.21420	411.21375	1.1
6	429.22476	429.22406	1.6
7	443.24041	443.2396	1.8
8	455.20402	455.20289	2.5
9	461.25097	461.24999	2.1
10	598.29865	598.29769	1.6
11	616.30922	616.30779	2.3
12	631.38299	631.38255	0.7
13	990.54101	990.53986	1.2

27-O-Desmethyl, 45-Hydroxy Zotarolimus Metabolites Fragmentation Pattern

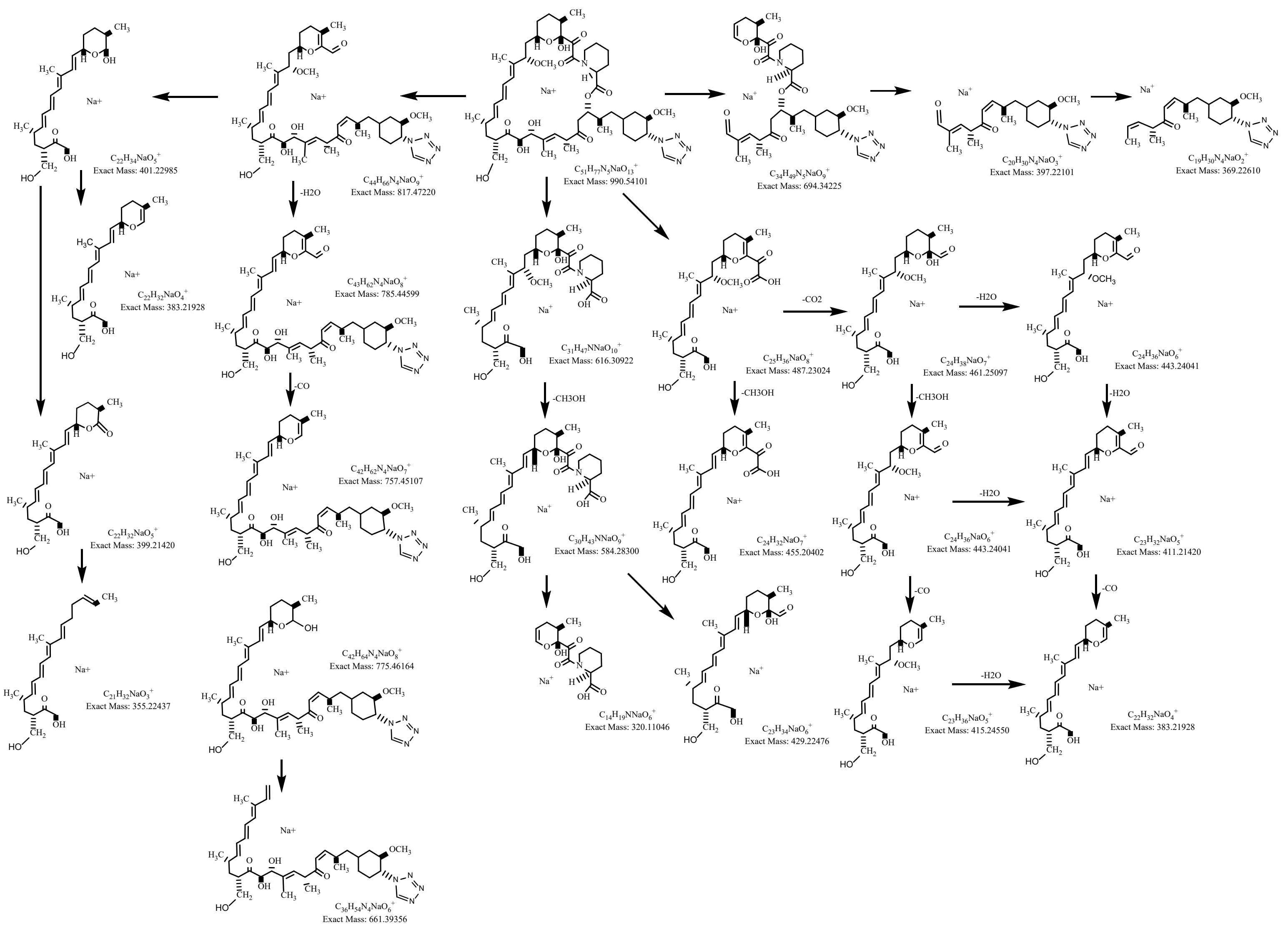

Structural Confirmation of 16-O-Desmethyl, 45/46-Hydroxy Zotarolimus

Zotarolimus Fragments	16-O-desmethyl, 45/46-hydroxy-Zotarolimus Fragments	Comments	
320.11046	320.1098	no hydroxylation, no desmethylation	Rules out 11, 12, 14, or piperidine-OH
369.2261	369.22538	no hydroxylation, no desmethylation	Rules out 47, 48, or 49-OH/ Rules out 39-O-desmethyl
397.22101	397.22005	no hydroxylation, no desmethylation	Rules out 47, 48, or 49-OH/ Rules out 39-O-desmethyl
409.23493	411.21375	hydroxylation, desmethylation	11, 12, 14, 23, 24, 25, 45, or 46-OH present/ confirms 27-O-desmethyl
427.2455	429.22406	hydroxylation, desmethylation	11, 12, 14, 23, 24, 25, 45, or 46-OH present/ confirms 27-O-desmethyl
413.26623	415.24499	hydroxylation, desmethylation (low intensity)	11, 12, 14, 23, 24, 25, 45, or 46-OH present/ 16- or 27-O-desmethyl present
485.25097	487.25023	hydroxylation, desmethylation (low intensity)	11, 12, 14, 23, 24, 25, 45, or 46-OH present/ 16- or 27-O-desmethyl present
582.30374	584.30336	hydroxylation, desmethylation	11, 12, 14, 23, 24, 25, 45, 46, or Piperidine-OH present/ confirms 27-O-desmethyl present
453.22476	455.20289	hydroxylation, desmethylation	11, 12, 14, 23, 24, 25, 45, or 46-OH present/ confirms 27-O-desmethyl
459.27171	461.24999	hydroxylation, desmethylation	11, 12, 14, 23, 24, 25, 45, 46, or Piperidine-OH present/ 16- or 27-O-desmethyl present
614.32995	616.30779	hydroxylation, desmethylation	11, 12, 14, 23, 24, 25, 45, 46, or Piperidine-OH present/ 16- or 27-O-desmethyl present
659.41429	661.3935	hydroxylation, desmethylation	23, 24, 25, 45, 46, 47, 48, or 49-OH present/ 27- or 39-O-desmethyl present
988.56174	990.53986	hydroxylation, desmethylation	
Characteristic Fragments			351, 397
Determinant patterns			Methanol loss<br>383- 32 = 351<br>429- 32 = 397

Didesmethyl Zotarolimus Metabolites, (m/z = 960.53044)

Didesmethyl Zotarolimus metabolites

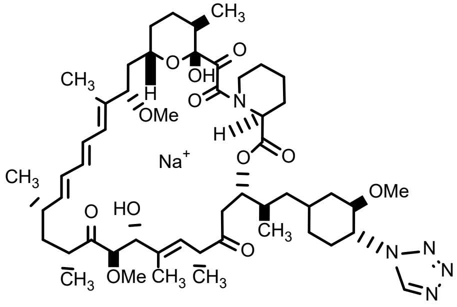

$C_{52}H_{79}N_5NaO_{12}^+$
Exact Mass: 988.56174

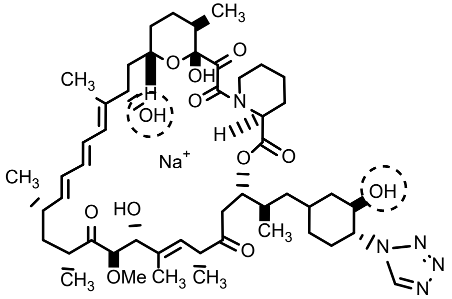

16,39-O-didesmethyl zotarolimus
$C_{50}H_{75}N_5NaO_{12}^+$
Exact Mass: 960.53044

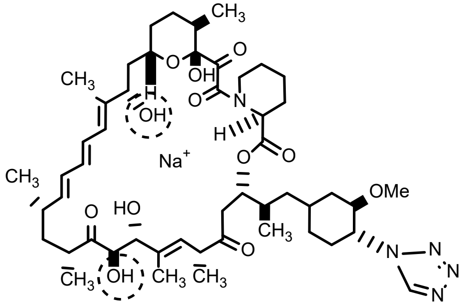

16, 27-O-didesmethyl zotarolimus

$C_{50}H_{73}N_5NaO_{12}^+$
Exact Mass: 958.51479

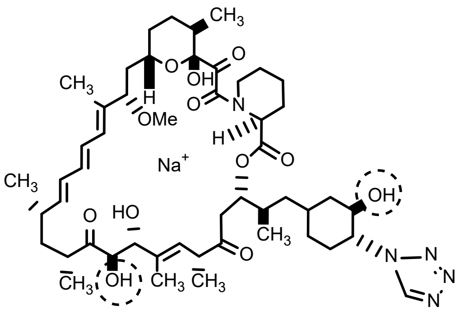

27, 39-O-didesmethyl zotarolimus

$C_{50}H_{75}N_5NaO_{12}^+$
Exact Mass: 960.53044

16, 39-O-Didesmethyl Zotarolimus Metabolites
Extracted Ion Chromatogram (EIC), m/z = 960.53044

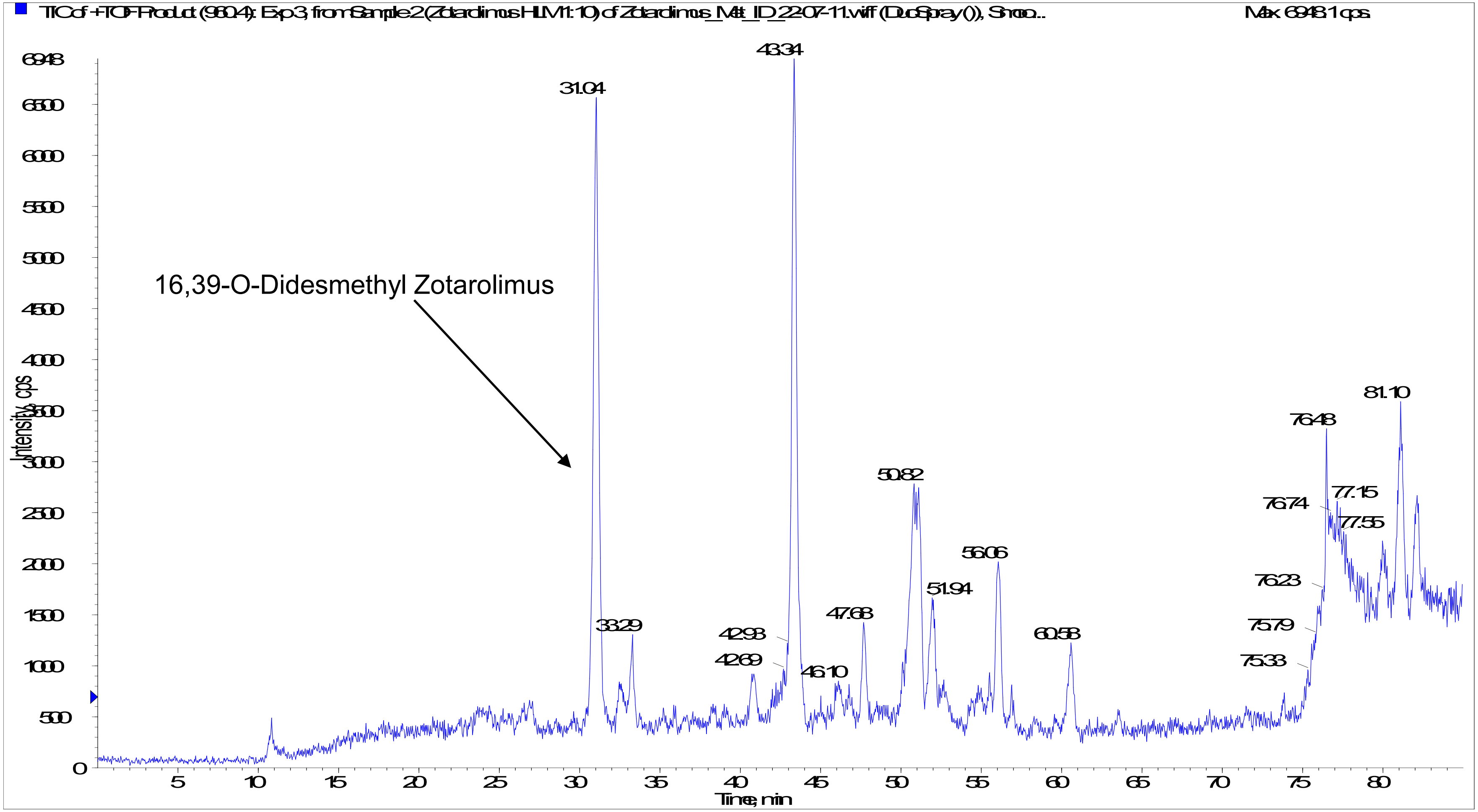

16, 39-O-didesmethyl Zotarolimus, m/z = 1020.55157

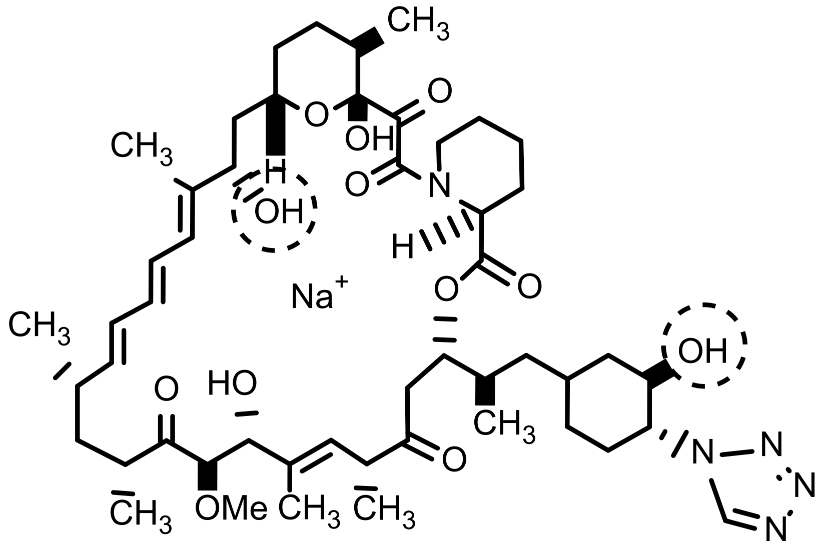

16,39-O-didesmethyl zotarolimus

$C_{50}H_{75}N_5NaO_{12}^+$

Exact Mass: 960.53044

16, 39-O-Didesmethyl Zotarolimus

Mass Spectrum (TOF Fragmentation)

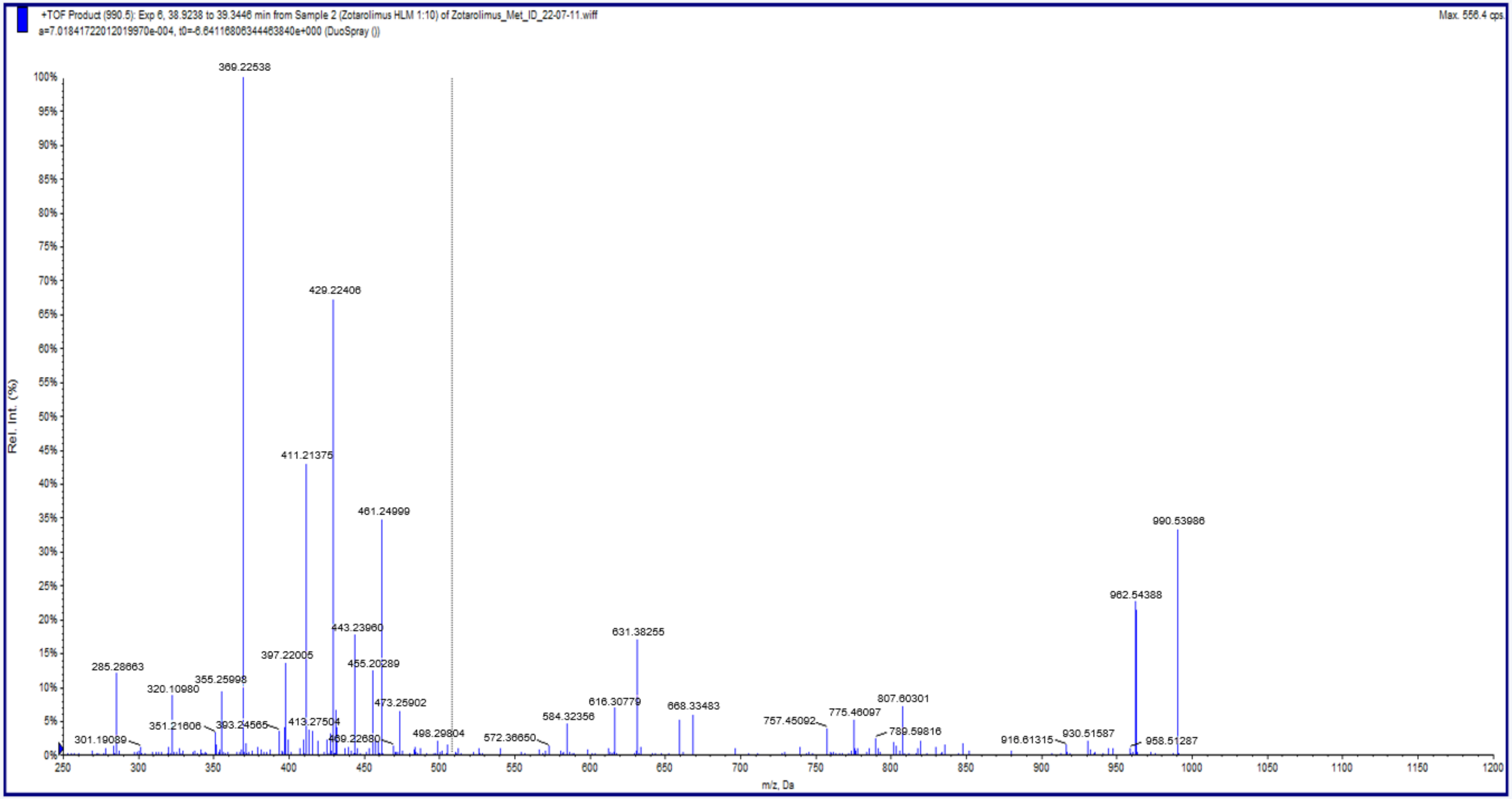

Δppm of 16, 39-O-Didesmethyl Zotarolimus

Fragment No.	Theoretical mass	Measured mass	Δppm
1	320.11046	320.11003	1.3
2	355.21045	355.21007	1.1
3	381.24002	381.23965	1.0
4	397.22101	397.22066	0.9
5	409.23493	409.23466	0.7
6	427.24550	427.24489	1.4
7	441.26115	441.26088	0.6
8	453.22476	453.22409	1.5
9	582.30374	582.30297	1.3
10	600.31430	600.31387	0.7
11	645.39864	645.39803	0.9
12	680.32660	680.32599	0.9
13	741.45616	741.45587	0.4
14	769.45107	769.45006	1.3
15	960.53044	960.52965	0.8

16, 39-O-Didesmethyl Zotarolimus Fragmentation Pattern

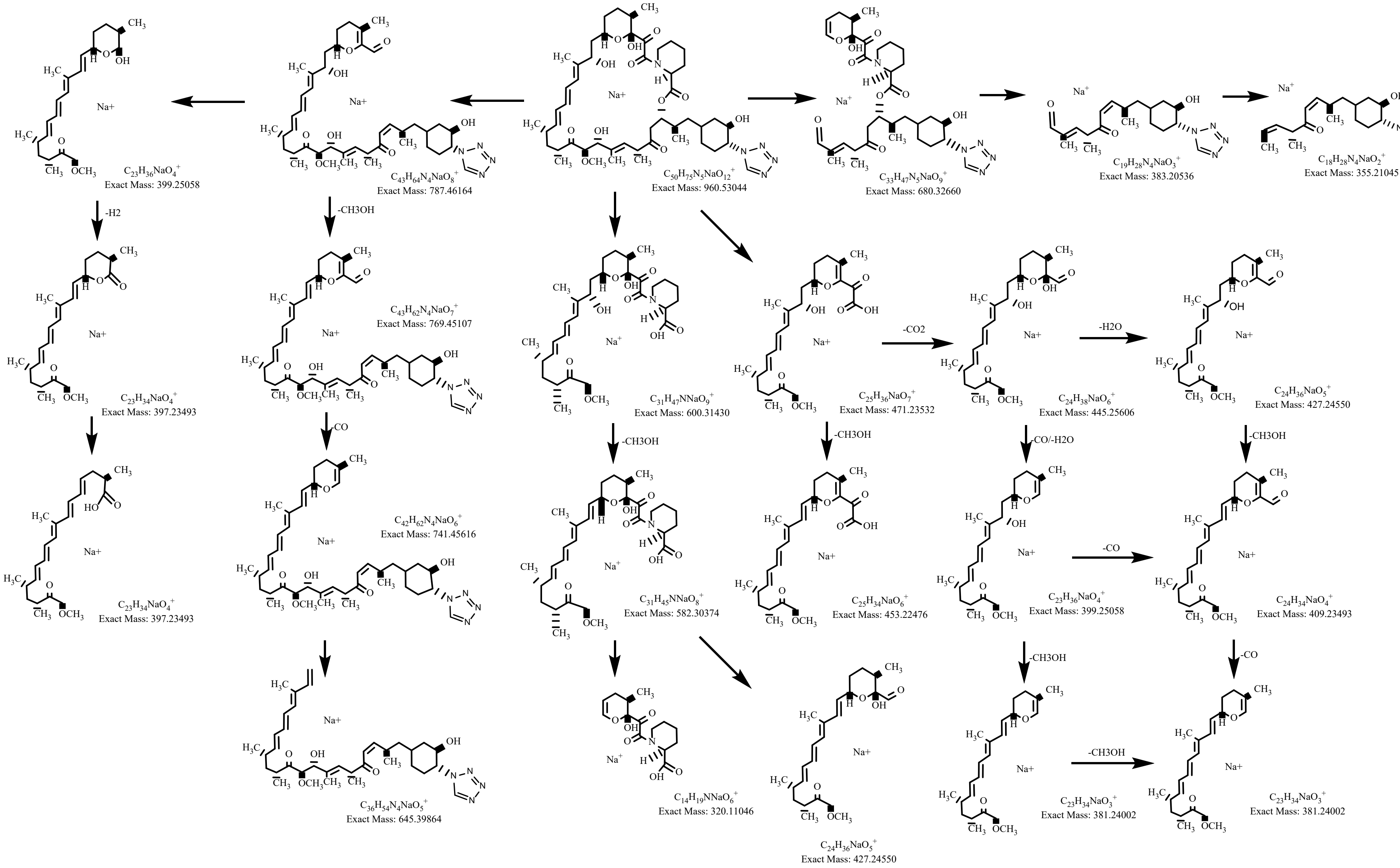

16, 39-O-Didesmethyl Zotarolimus Characteristic Fragmentation Pattern

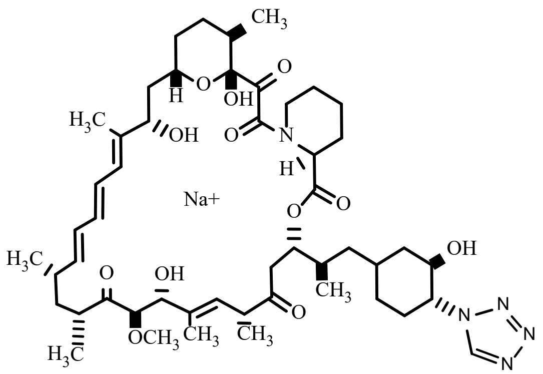

Chemical Formula: $C_{50}H_{75}N_5NaO_{12}^{+}$
Exact Mass: 960.53044

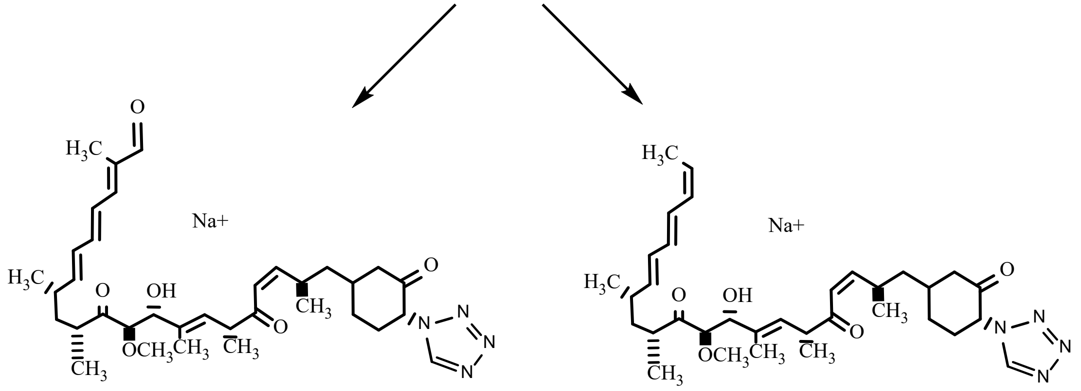

$C_{35}H_{50}N_4NaO_6^{+}$
Exact Mass: 645.36226

$C_{34}H_{50}N_4NaO_5^{+}$
Exact Mass: 617.36734

Structural Confirmation of 16, 39-O-Didesmethyl Zotarolimus

Zotarolimus Fragments	16,39-O-Didesmethyl Zotarolimus Fragments	Comments	
320.11046	320.11023	no demethylation	Not conclusive
369.22610	355.21045	Demethylation	Confirms 39-O-desmethyl
381.24002	381.23965	no demethylation	Confirms 16-O-desmethyl
397.23493	397.22066	no demethylation	Confirms 16-O-desmethyl
409.23493	409.23466	no demethylation	confirms 16-O-desmethyl
441.26115	427.24489	Demethylation	16, or 27-O-desmethyl present
453.22476	453.22409	no demethylation	Rules out out 27-O-desmethyl
582.30374	582.30297	no demethylation	Rules out out 27-O-desmethyl
614.32995	600.31387	Demethylation	16, and 27-O-desmethyl present
659.41429	645.39803	Demethylation	27, or 39-O-desmethyl present
694.34225	680.32599	Demethylation	Confirms 39-O-desmethyl
755.47181	741.45587	Demethylation	16, 27, or 39-O-desmethyl present
783.46672	769.45006	Demethylation	16, 27, or 39-O-desmethyl present
960.53044	960.52965	no demethylation	
Characteristic Fragments	355, 381, 397, 409, 617, and 645		
Determinant patterns			

27, 39-O-didesmethyl Zotarolimus, m/z = 1020.55157

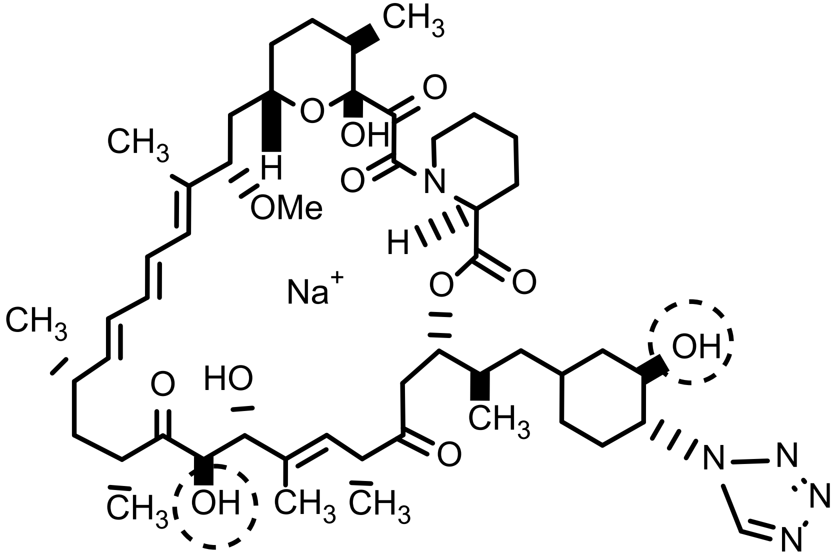

27, 39-O-didesmethyl zotarolimus

$C_{50}H_{75}N_5NaO_{12}^+$

Exact Mass: 960.53044

27, 39-O-Didesmethyl Zotarolimus Metabolites
Extracted Ion Chromatogram (EIC), m/z = 960.53044

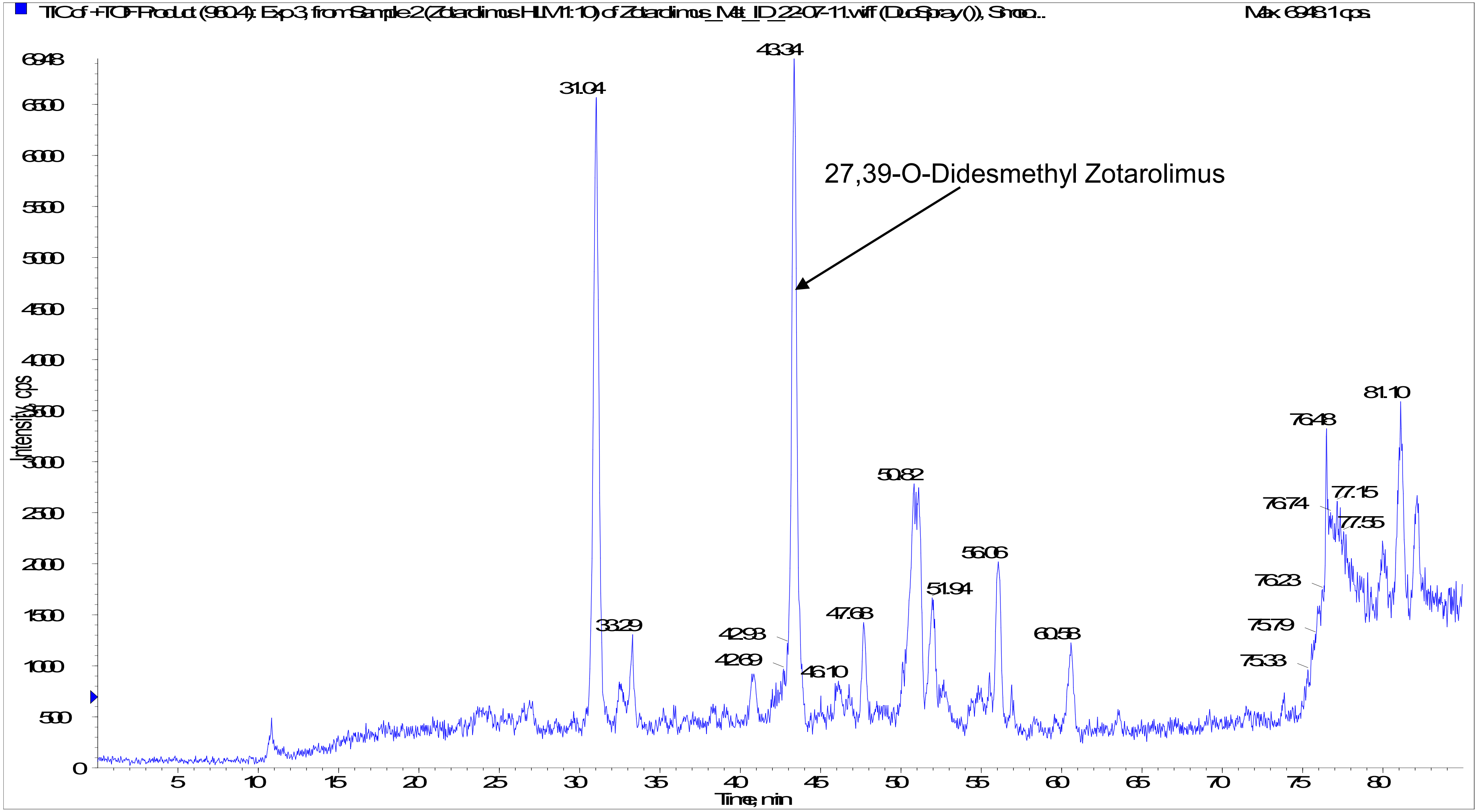

27, 39-O-Didesmethyl Zotarolimus

Mass Spectrum (TOF Fragmentation)

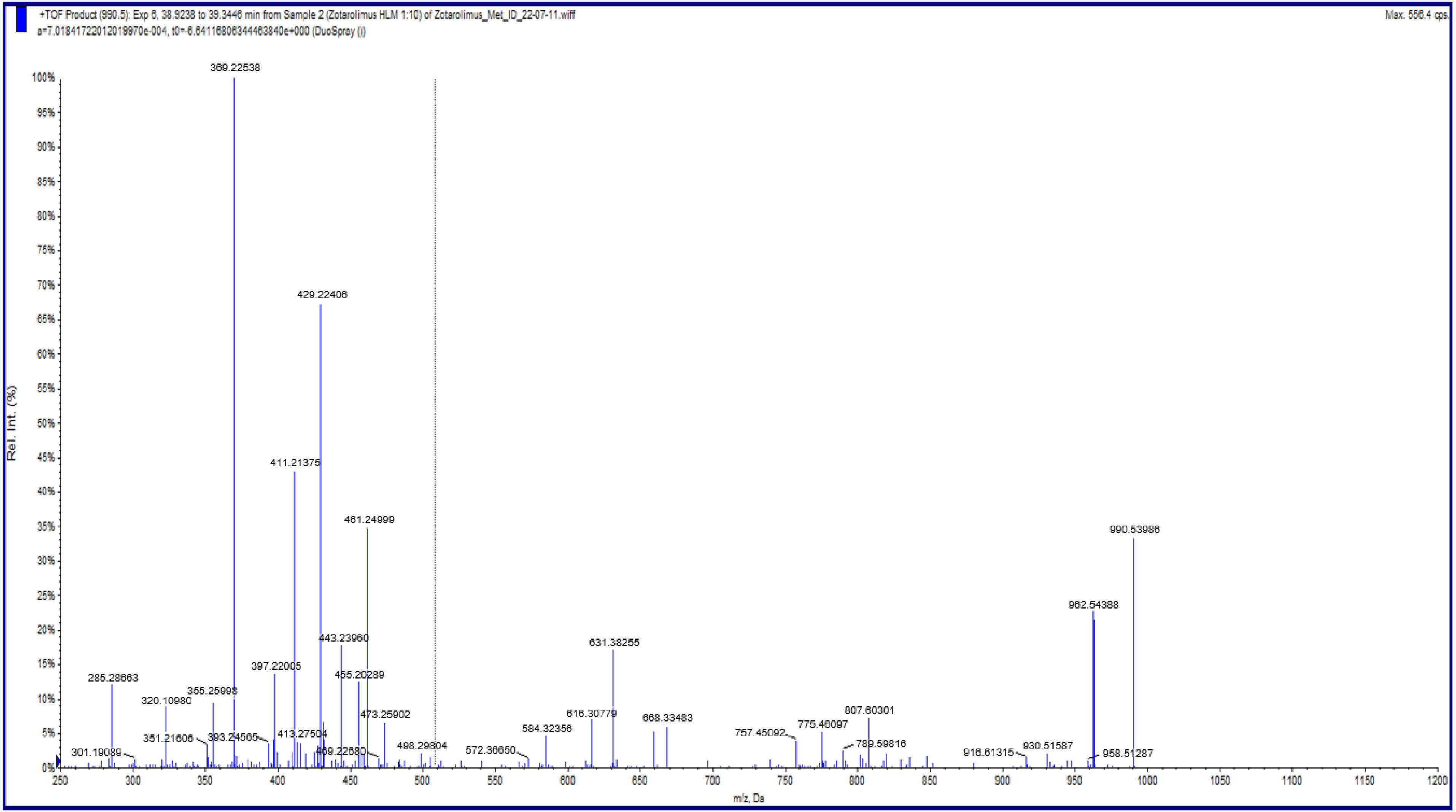

Δppm of 27, 39-O-Didesmethyl Zotarolimus

Fragment No.	Theoretical mass	Measured mass	Δppm
1	320.11046	320.10993	1.7
2	355.21045	355.20999	1.3
3	367.22437	367.22396	1.1
4	383.21928	383.21879	1.3
5	395.21928	395.21897	0.8
6	413.22985	413.22936	1.2
7	427.24550	427.24499	1.2
8	439.20911	439.20855	1.3
9	568.28809	568.28763	0.8
10	600.31430	600.31357	1.2
11	631.38299	631.38236	1.0
13	727.44051	727.44003	0.7
14	755.43542	755.43487	0.7
15	960.53044	960.52938	1.1

27, 39-O-Didesmethyl Zotarolimus Fragmentation Pattern

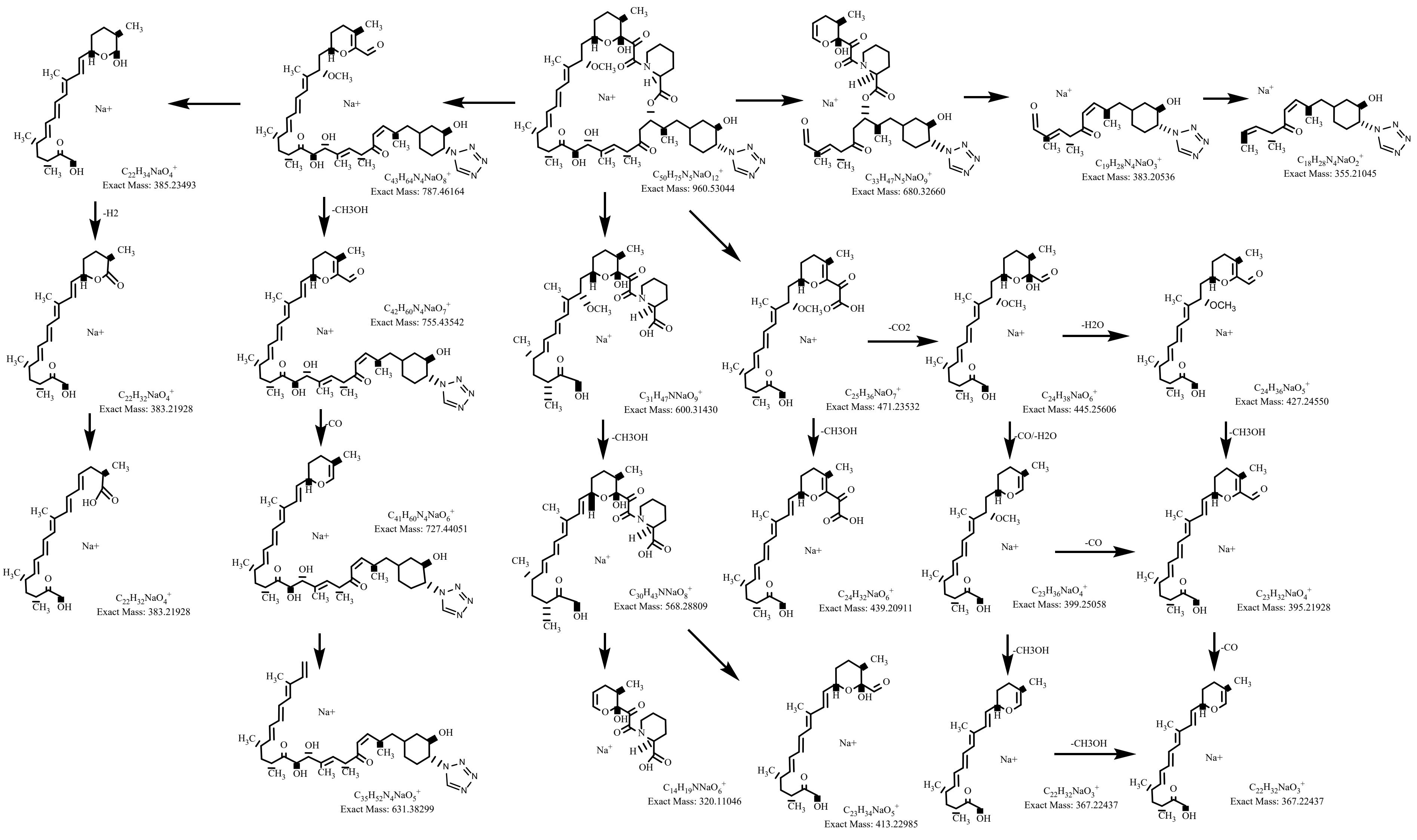

Structural Confirmation of 27, 39-O-Didesmethyl Zotarolimus

Zotarolimus Fragments	27,39-O-Didesmethyl Zotarolimus Fragments	Comments	
320.11046	320.10993	no demethylation	Not conclusive
369.22610	355.20999	Demethylation	Confirms 39-O-desmethyl
381.24002	367.22396	Demethylation	Confirms 27-O-desmethyl
409.23493	395.21897	Demethylation	Confirms 27-O-desmethyl
427.24550	413.22936	Demethylation	Confirms 27-O-desmethyl
441.26115	427.24499	Demethylation	16, or 27-O-desmethyl present
453.22476	439.20855	Demethylation	Confirms 27-O-desmethyl
485.25097	471.23532	Demethylation	16, or 27-O-desmethyl present
614.32995	600.31357	Demethylation	16, and 27-O-desmethyl present
659.41429	631.38236	Demethylation	Confirms 27,39-O-didesmethyl
694.34225	680.32599	very low	NA
755.47181	727.44003	Demethylation	16, 27, or 39-O-desmethyl present
783.46672	755.43487	Demethylation	16, 27, or 39-O-desmethyl present
960.53044	960.52938	no demethylation	
Characteristic Fragments	355, 367, 395, 413, 439		
Determinant patterns			No water loss possible for fragments 367, 383, 385, 395, 413, 427, 439, 471, 568, and 600

16, 27-O-didesmethyl Zotarolimus, m/z = 1020.55157

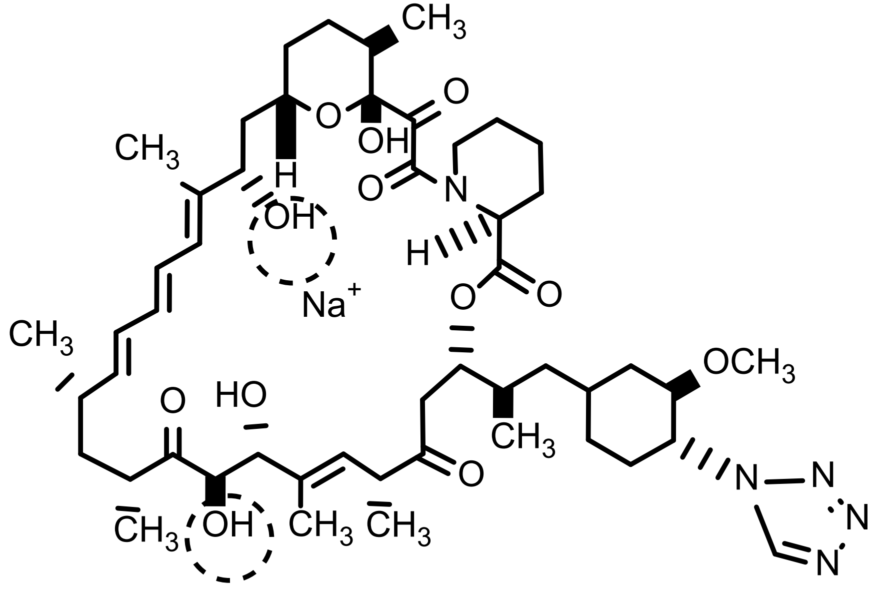

16, 27-O-didesmethyl zotarolimus

$C_{50}H_{75}N_5NaO_{12}^+$

Exact Mass: 960.53044

16, 27-O-Didesmethyl Zotarolimus Metabolites
Extracted Ion Chromatogram (EIC), m/z = 960.53044

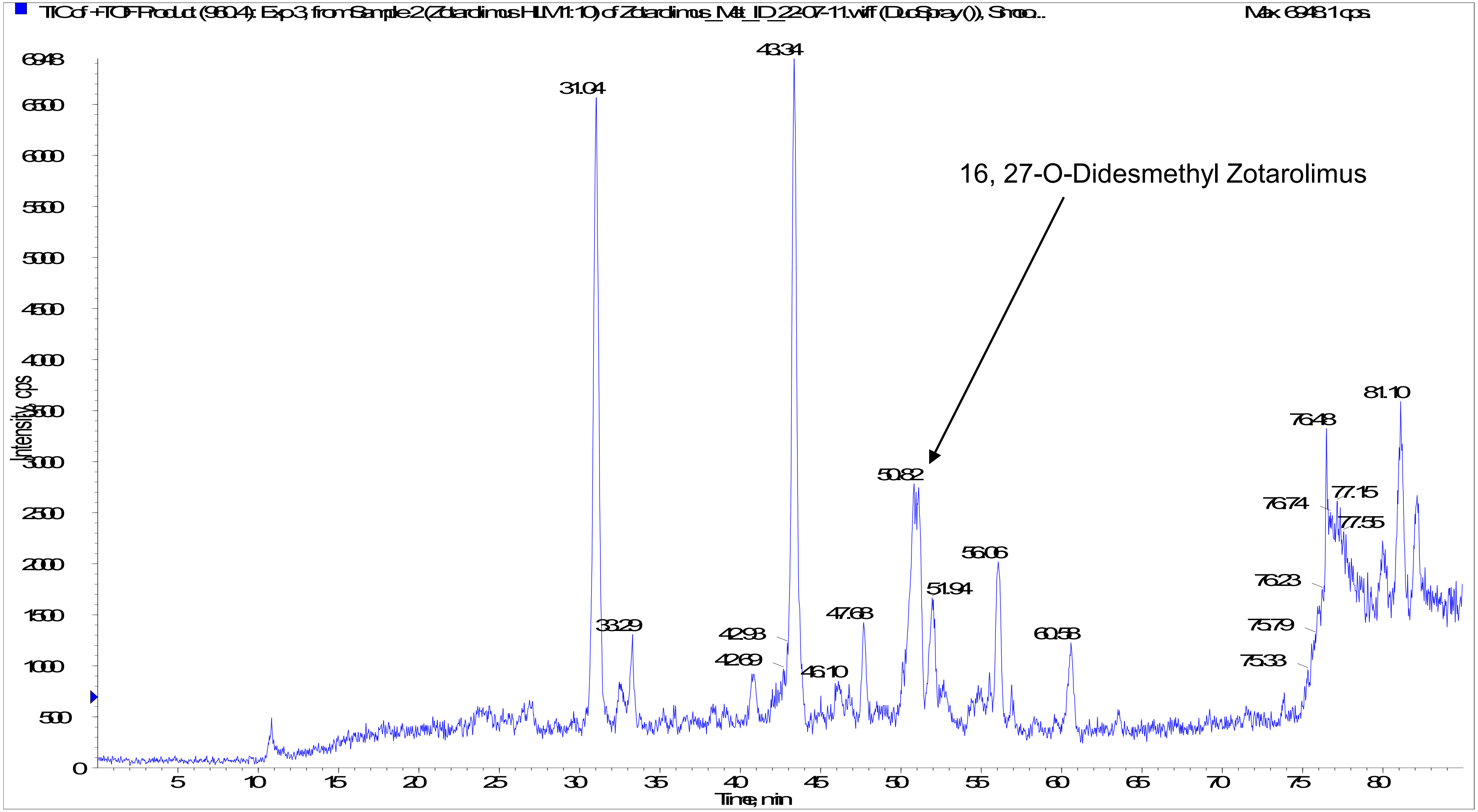

16, 27-O-Didesmethyl Zotarolimus

Mass Spectrum (TOF Fragmentation)

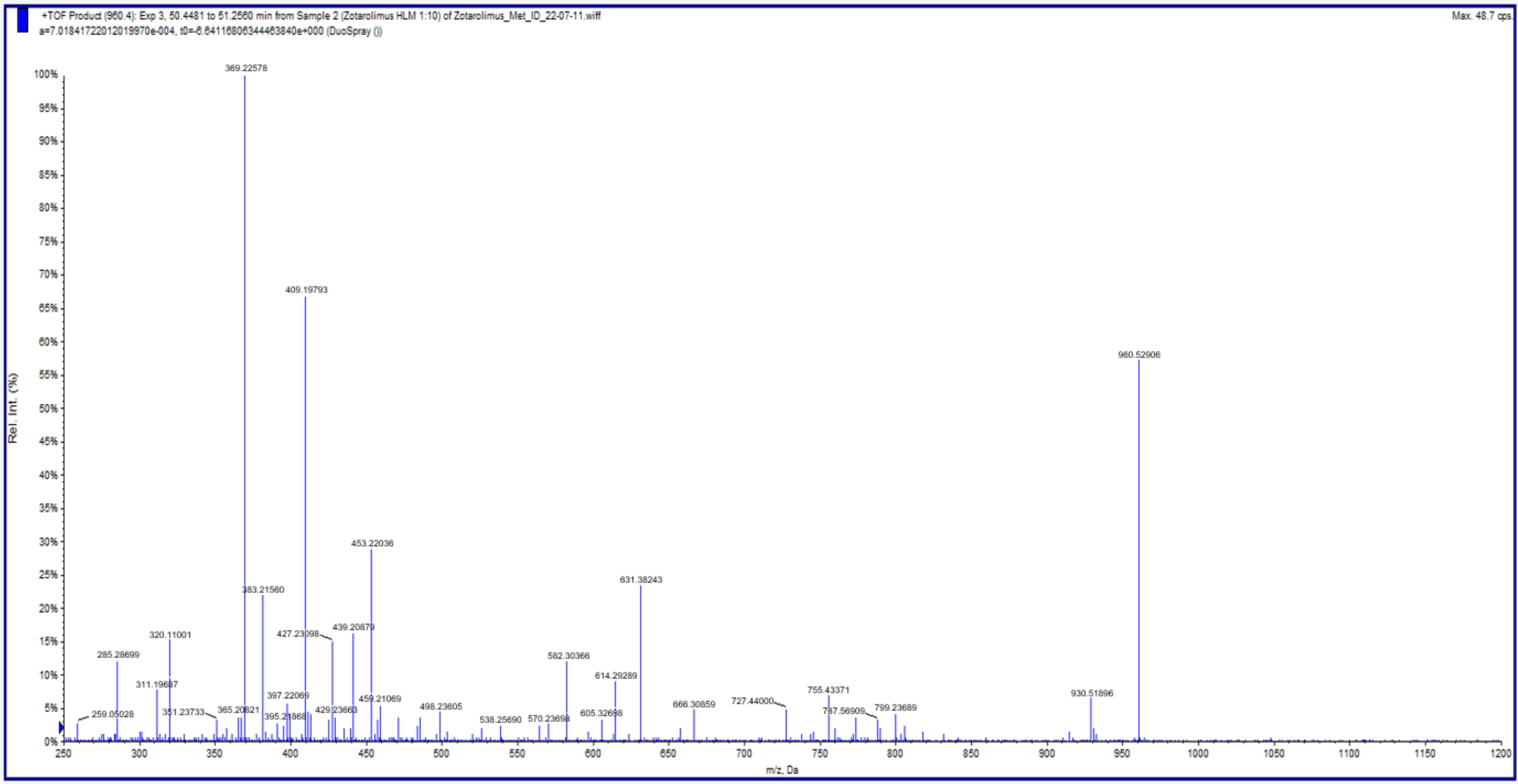

Δppm of 16, 27-O-Didesmethyl Zotarolimus

Fragment No.	Theoretical mass	Measured mass	Δppm
1	320.11046	320.11001	1.4
2	369.22610	369.22578	0.9
3	397.22101	397.22069	0.8
4	383.21928	383.21860	1.8
5	395.21928	395.21868	1.5
6	409.19855	409.19793	1.5
7	427.23158	427.23098	1.4
8	439.20911	439.20879	0.7
9	568.28809	568.28703	1.9
10	600.31430	ND	NA
11	631.38299	631.38243	0.9
13	727.44051	727.44000	0.7
15	960.53044	960.52906	1.4

16, 27-O-Didesmethyl Zotarolimus Fragmentation Pattern

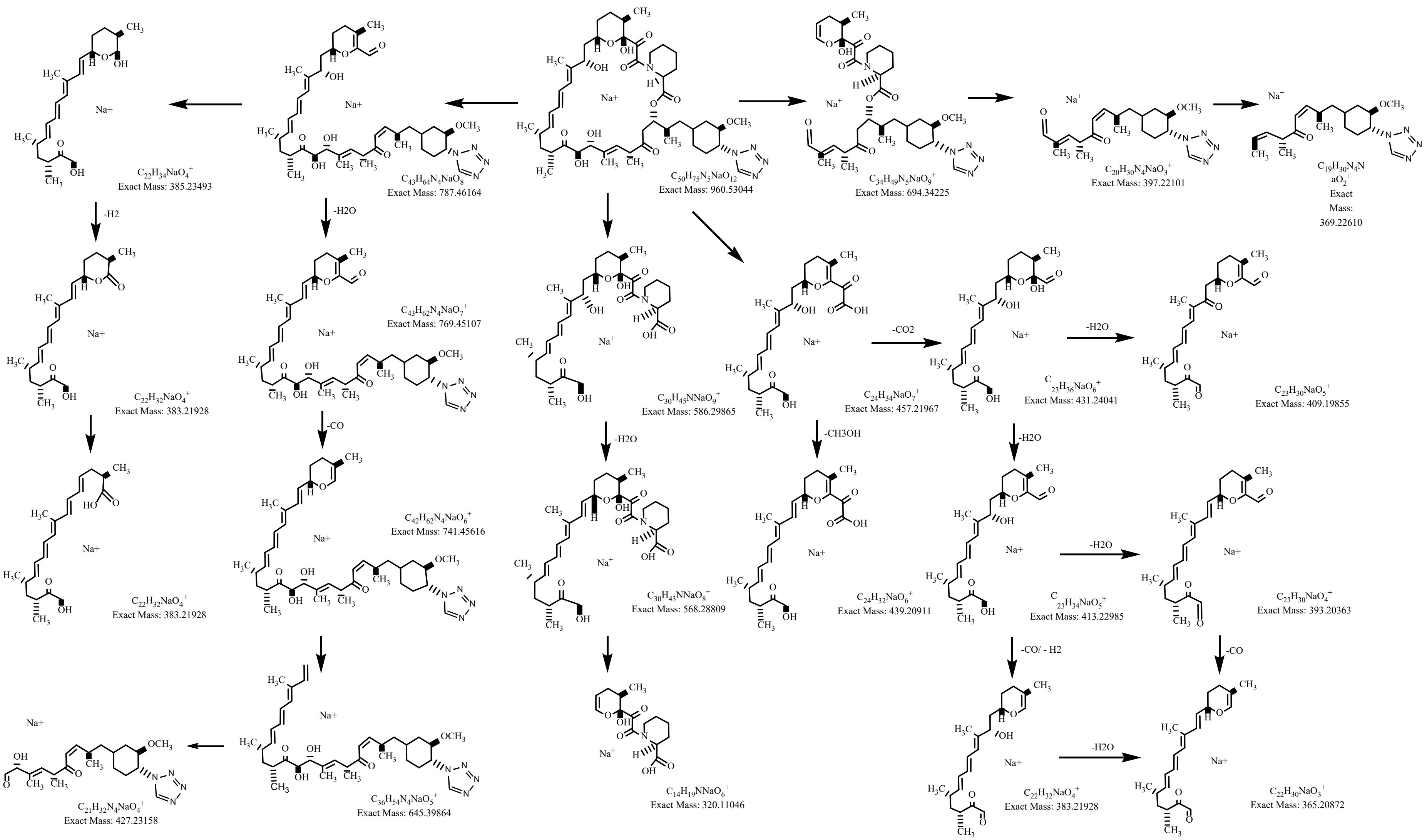

16, 27-O-Didesmethyl Zotarolimus Characteristic Fragmentation Pattern

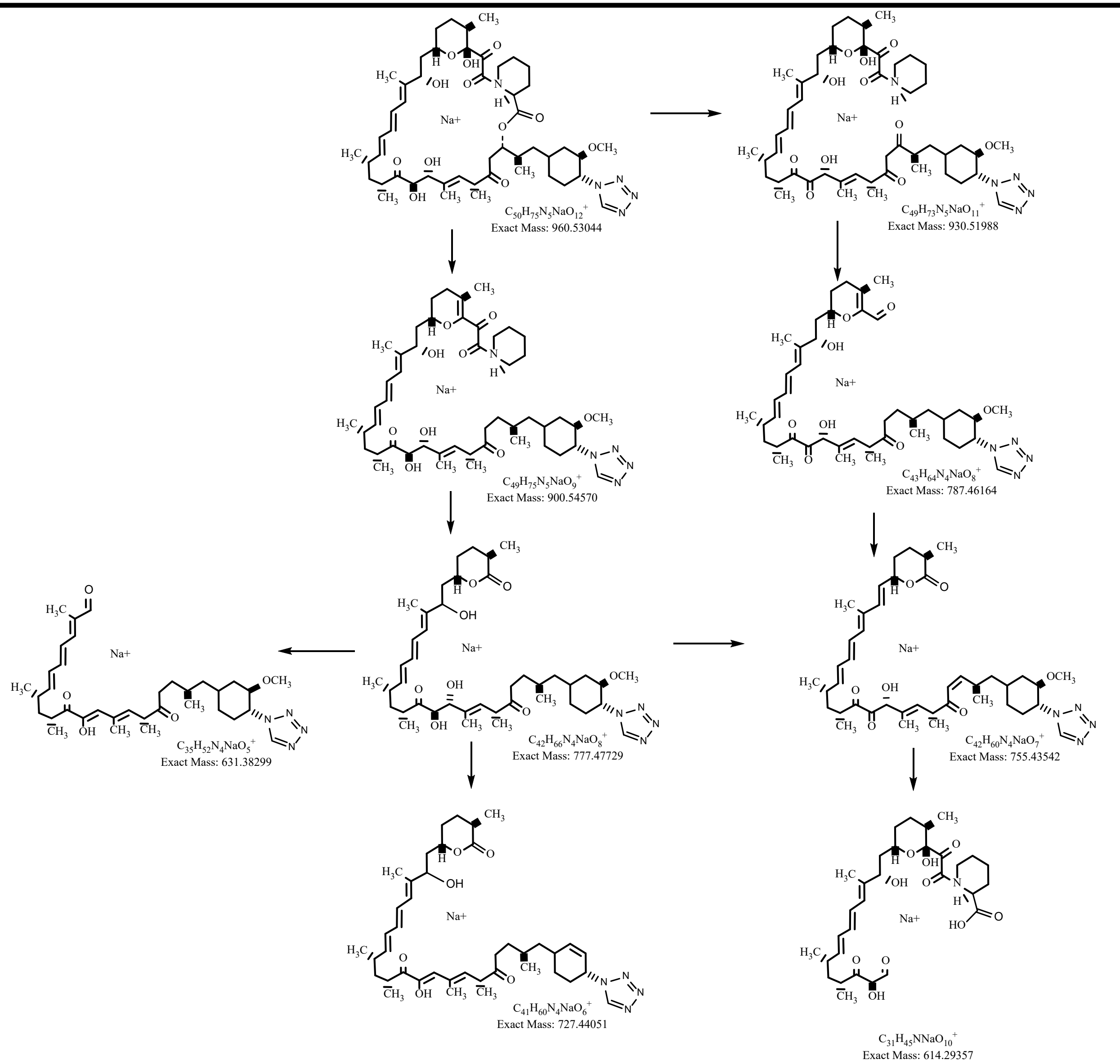

Structural Confirmation of 16, 27-O-Didesmethyl Zotarolimus

Zotarolimus Fragments	16,27-O-Didesmethyl Zotarolimus Fragments	Comments	
320.11046	320.10993	no demethylation	Not conclusive
397.22101	369.22578	Demethylation	Rules out 39-O-desmethyl
381.24002	367.22344	Demethylation	Confirms 27-O-desmethyl
409.23493	395.21868	Demethylation	Confirms 27-O-desmethyl
427.24550	413.22901	Demethylation	Confirms 27-O-desmethyl
441.26115	(413.22985) ND	ND	NA
453.22476	439.20879	Demethylation	Confirms 27-O-desmethyl
485.25097	457.21885	Demethylation (low intensity)	Confirms 16 and 27-O-desmethyl
614.32995	568.28703	Demethylation + water loss (very low intensity)	Confirms 16 and 27-O-desmethyl
659.41429	645.39864 (ND)	ND	NA
694.34225	694.34225 (ND)	ND	NA
755.47181	741.45616 (ND)	ND	NA
783.46672	769.45107 (ND)	ND	NA
960.53044	960.52906	no demethylation	
Characteristic Fragments	383, 409, 439, 614, and 631		
Determinant patterns			No water loss possible for fragments 383, 395, and 439

Dihydroxy Zotarolimus, m/z = 1020.55157

11, 24-Dihydroxy Zotarolimus, m/z = 1020.55157

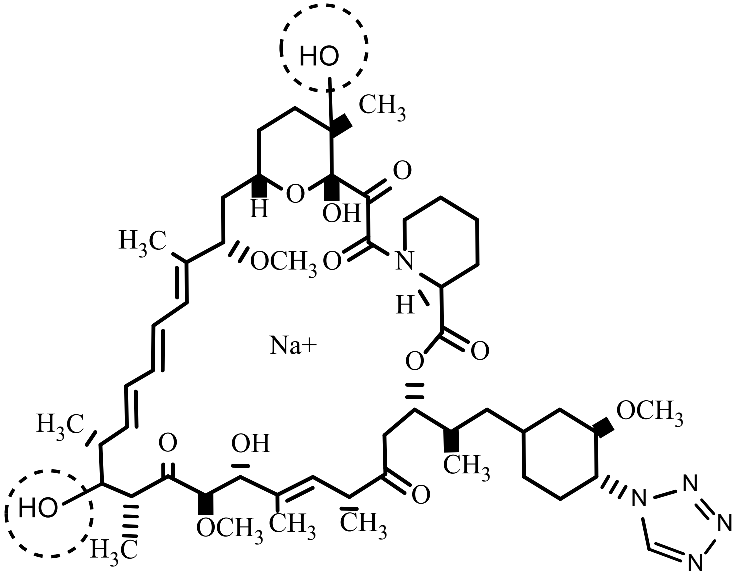

Chemical Formula: $C_{52}H_{79}N_5NaO_{14}^{+}$
Exact Mass: 1020.55157

11, 24-dihydroxy zotarolimus

Dihydroxy Zotarolimus Metabolites
Extracted Ion Chromatogram (EIC), m/z = 1020.55157

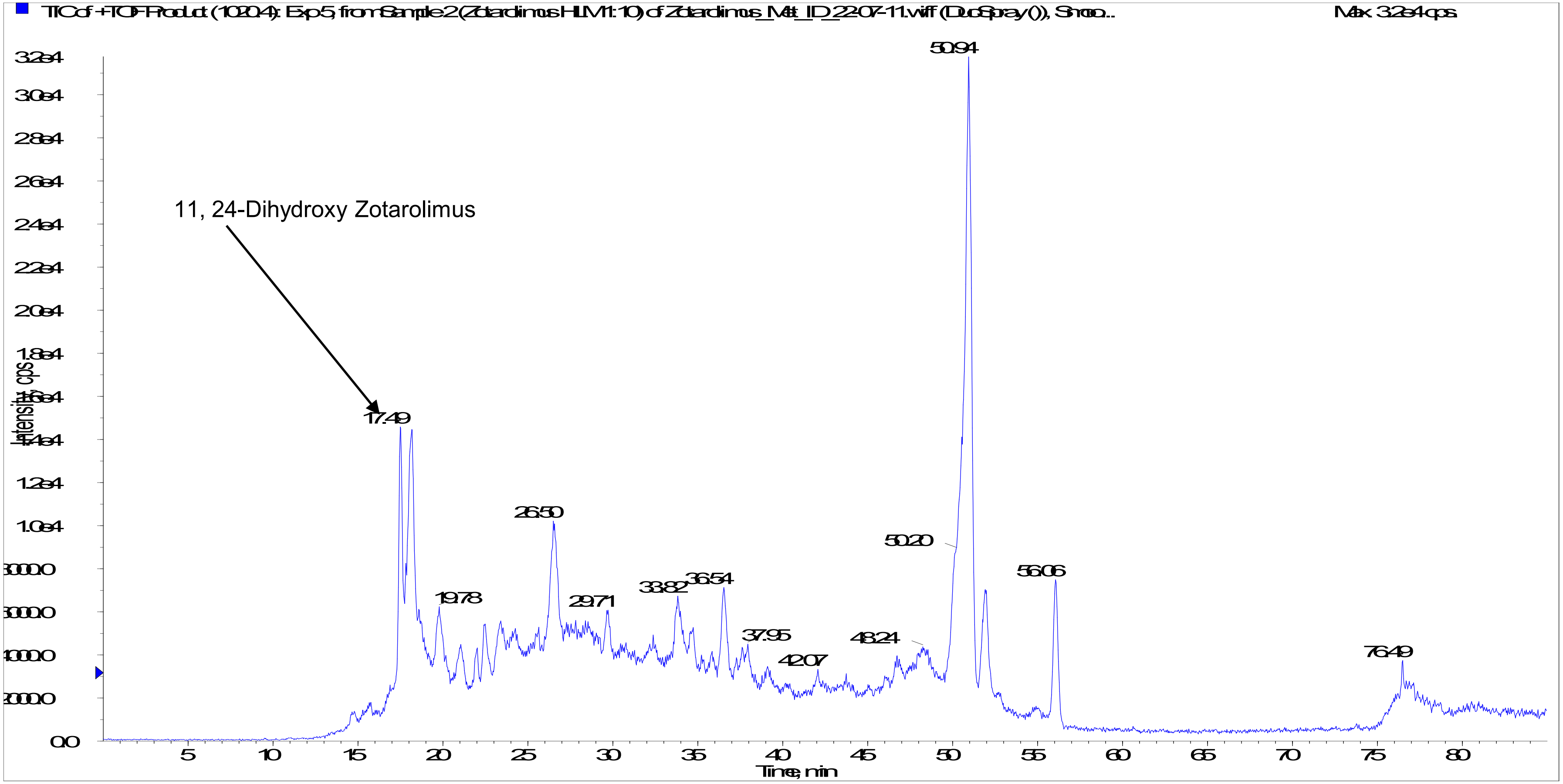

11, 24-Dihydroxy Zotarolimus

Mass Spectrum (TOF Fragmentation)

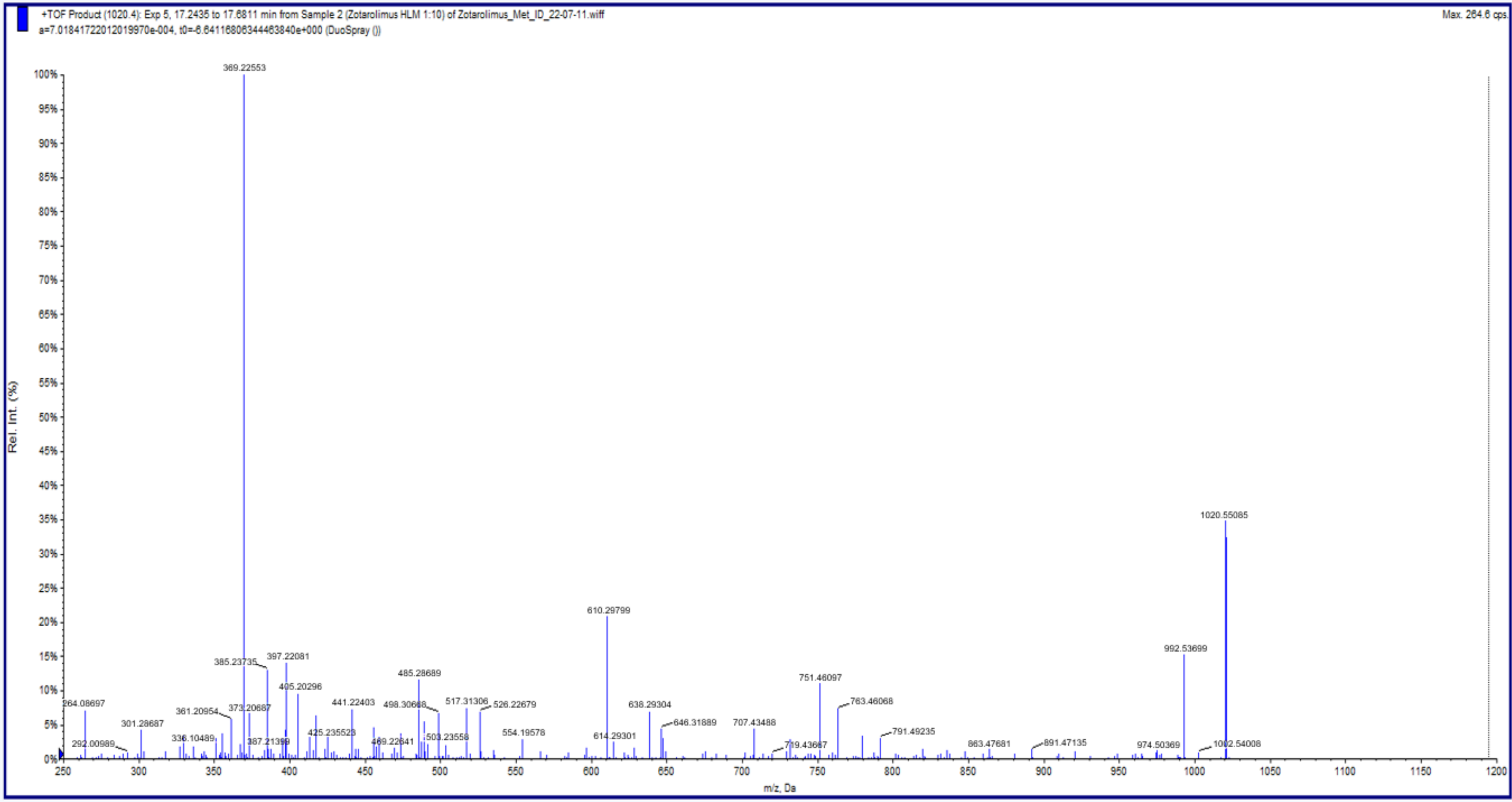

Δppm of 11, 24-Dihydroxy Zotarolimus

Fragment No.	Theoretical mass	Measured mass	Δppm
1	336.10537	336.10489	1.4
2	369.22610	369.22553	1.5
3	397.22101	397.22081	0.5
4	405.20363	405.20296	1.7
5	441.22476	441.22403	1.7
6	485.28736	485.28689	1.0
7	517.31357	517.31306	1.0
8	610.29865	610.29799	1.1
9	638.29357	638.29304	0.8
10	707.43542	707.43488	0.8
11	751.46164	751.46097	0.9
12	763.46164	763.46068	1.3
13	791.45655	791.45590	0.8
14	1002.54101	1002.54008	0.9
15	1020.55157	1020.55085	0.7

11, 24-Dihydroxy Zotarolimus Fragmentation Pattern

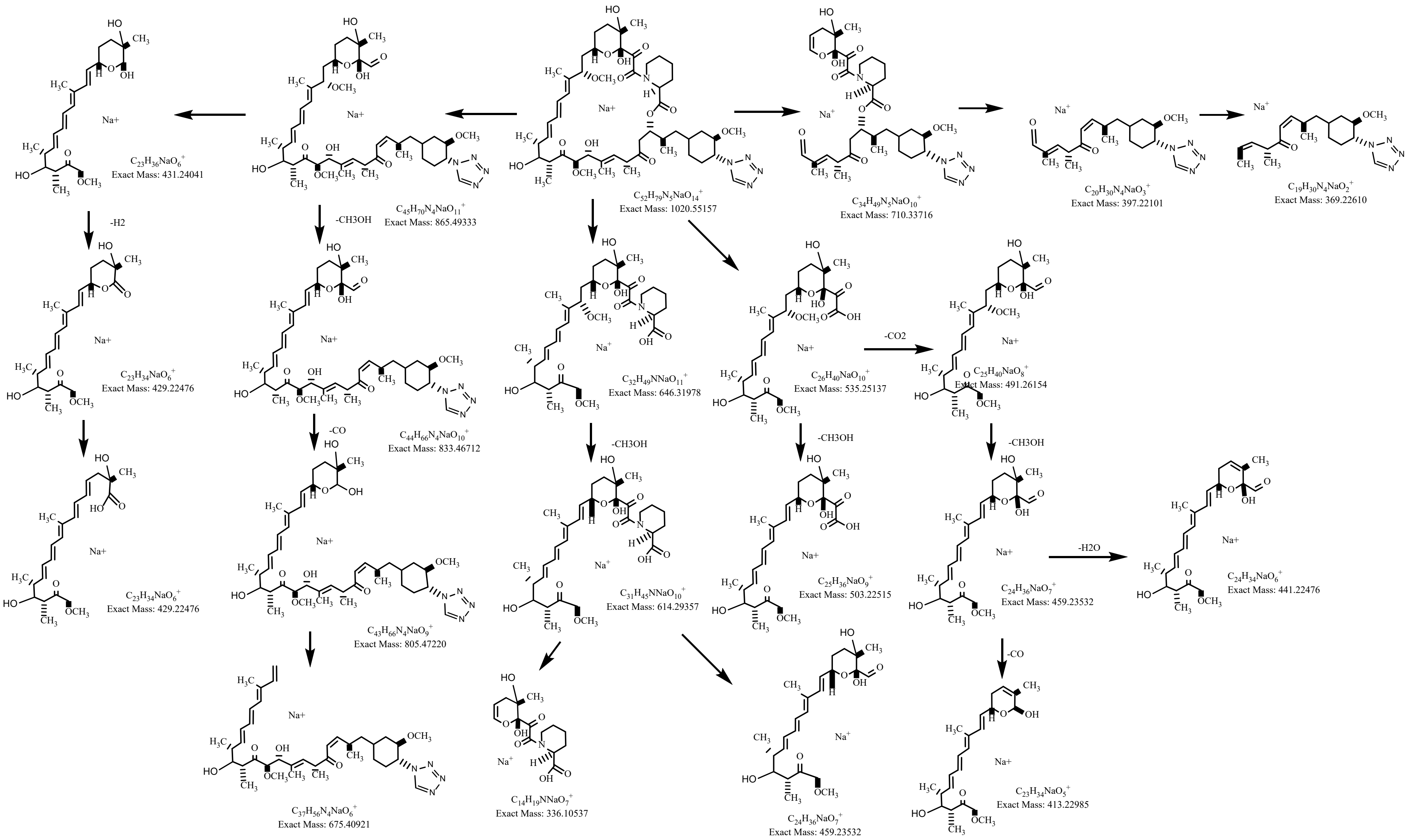

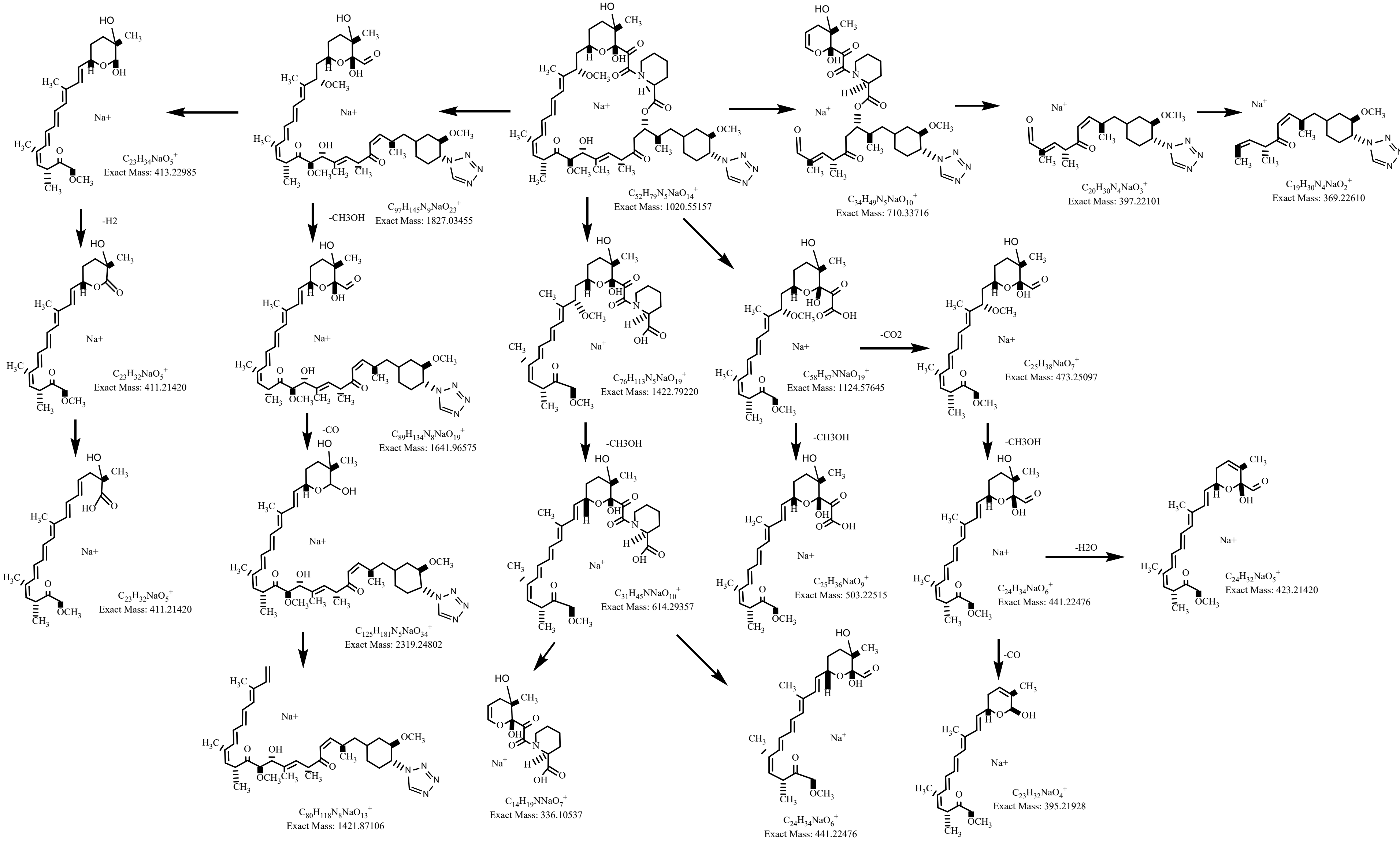
$C_{52}H_{79}N_5NaO_{14}^+$
Exact Mass: 1020.55157
$C_{97}H_{145}N_9NaO_{23}^+$
Exact Mass: 1827.03455
$C_{23}H_{34}NaO_5^+$
Exact Mass: 413.22985
$C_{34}H_{49}N_5NaO_{10}^+$
Exact Mass: 710.33716
$C_{20}H_{30}N_4NaO_3^+$
Exact Mass: 397.22101
$C_{19}H_{30}N_4NaO_2^+$
Exact Mass: 369.22610
-H2
$C_{23}H_{32}NaO_5^+$
Exact Mass: 411.21420
-CH3OH
$C_{89}H_{134}N_8NaO_{19}^+$
Exact Mass: 1641.96575
$C_{76}H_{113}N_5NaO_{19}^+$
Exact Mass: 1422.79220
$C_{58}H_{87}NNaO_{19}^+$
Exact Mass: 1124.57645
-CO2
$C_{25}H_{38}NaO_7^+$
Exact Mass: 473.25097
-CO
$C_{23}H_{32}NaO_5^+$
Exact Mass: 411.21420
$C_{125}H_{181}N_5NaO_{34}^+$
Exact Mass: 2319.24802
-CH3OH
$C_{31}H_{45}NNaO_{10}^+$
Exact Mass: 614.29357
-CH3OH
$C_{25}H_{36}NaO_9^+$
Exact Mass: 503.22515
-CH3OH
$C_{24}H_{34}NaO_6^+$
Exact Mass: 441.22476
-H2O
$C_{24}H_{32}NaO_5^+$
Exact Mass: 423.21420
$C_{80}H_{118}N_8NaO_{13}^+$
Exact Mass: 1421.87106
$C_{14}H_{19}NNaO_7^+$
Exact Mass: 336.10537
$C_{24}H_{34}NaO_6^+$
Exact Mass: 441.22476
-CO
$C_{23}H_{32}NaO_4^+$
Exact Mass: 395.21928

11, 24-Dihydroxy Zotarolimus Characteristic Fragmentation Pattern

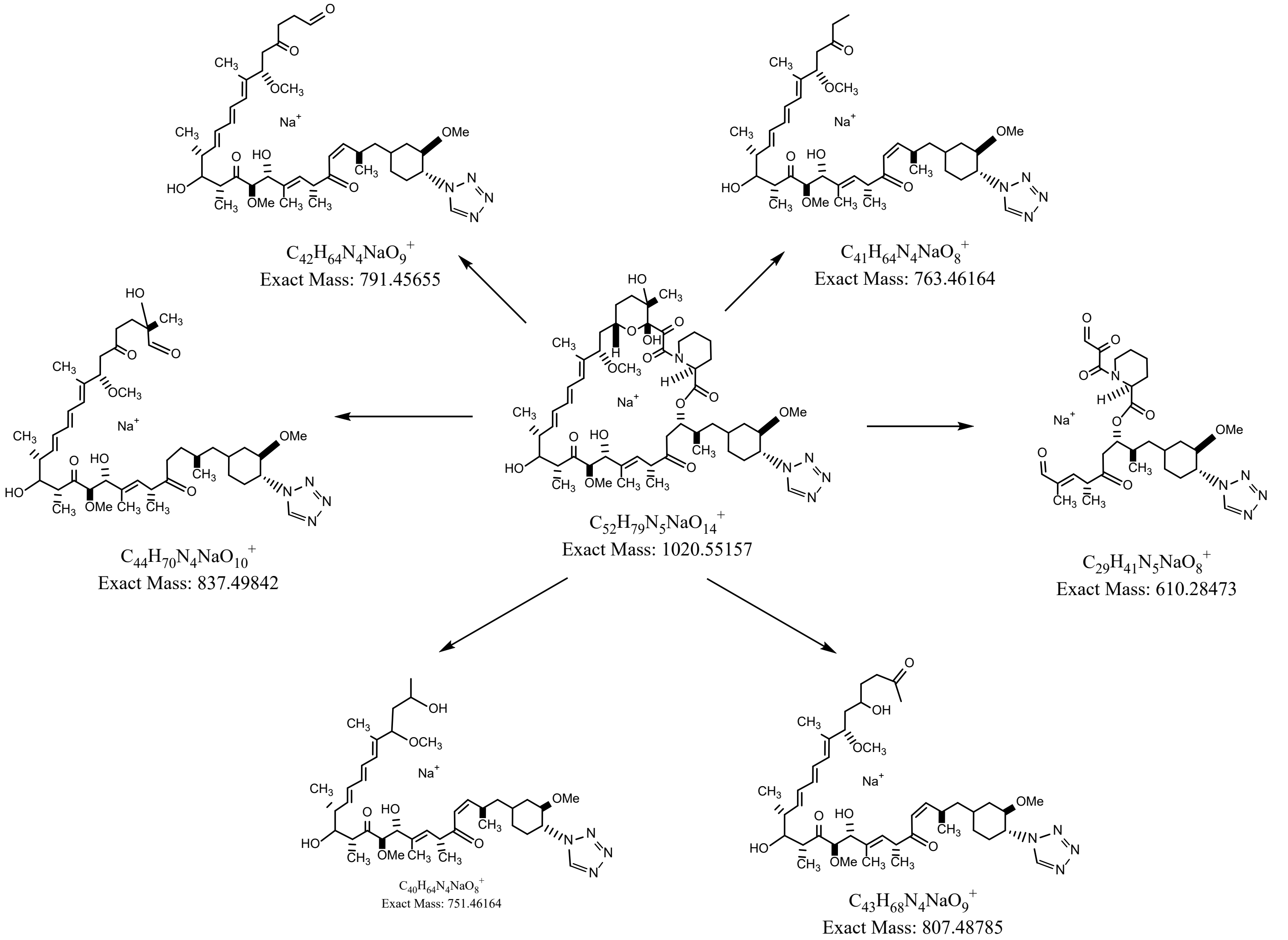

Structural Confirmation of 11, 24-dihydroxy Zotarolimus

Zotarolimus Fragments	11,24-dihydroxy Zotarolimus Fragments	Comments	
320.11046	336.10489	hydroxylation	11, 12, 14, or OH-piperidine present
369.22610	369.22553	no hydroxylation	Rules out 47, 48, and 49-OH
397.22101	397.220831	no hydroxylation	Rules out 47, 48, and 49-OH
409.23493	441.22403	double hydroxylation with water loss	11, 12, 14, 23, 24, 45, or 46-OH present
427.2455	405.20296	double hydroxylation with double water loss	11, 12, 14, 23, 24, 45, or 46-OH present
441.26115	(491.26154) ND	ND	NA
453.22476	503.22406	double hydroxylation	11, 12, 14, 23, 24, 45, or 46-OH present
485.25097	517.31306	double hydroxylation with water loss	11, 12, 14, 23, 24, 45, or 46-OH present
614.32995	646.31889	double hydroxylation	11, 12, 14, 23, 24, 45, or 46-OH present
659.41429	675.40865	hydroxylation	11, 12, 14, 23, 24, 45, or 46-OH present
694.34225	710.33716	ND	11, 12, 14, 23, 24, 45, or 46-OH present
755.47181	805.47220	very low	11, 12, 14, 23, 24, 45, or 46, 49-OH present
783.46672	833.46712	very low	16, 27, or 39-O-desmethyl present
988.56174	1020.55085	no demethylation	
Characteristic Fragments	610, 763, 791		
Determinant patterns			water loss due to 24-hydroxylation position

12, 24-Dihydroxy Zotarolimus, m/z = 1020.55157

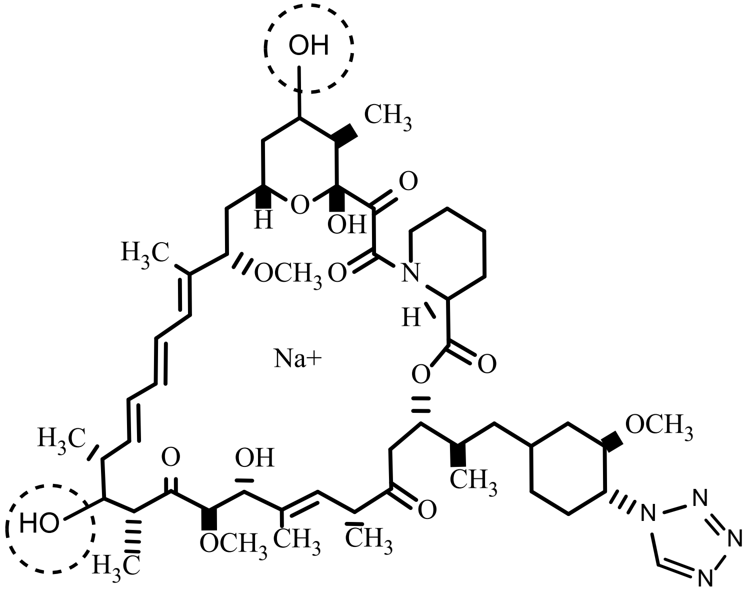

Chemical Formula: $C_{52}H_{79}N_5NaO_{14}^{+}$
Exact Mass: 1020.55157

12, 24-dihydroxy zotarolimus

Dihydroxy Zotarolimus Metabolites
Extracted Ion Chromatogram (EIC), m/z = 1020.55157

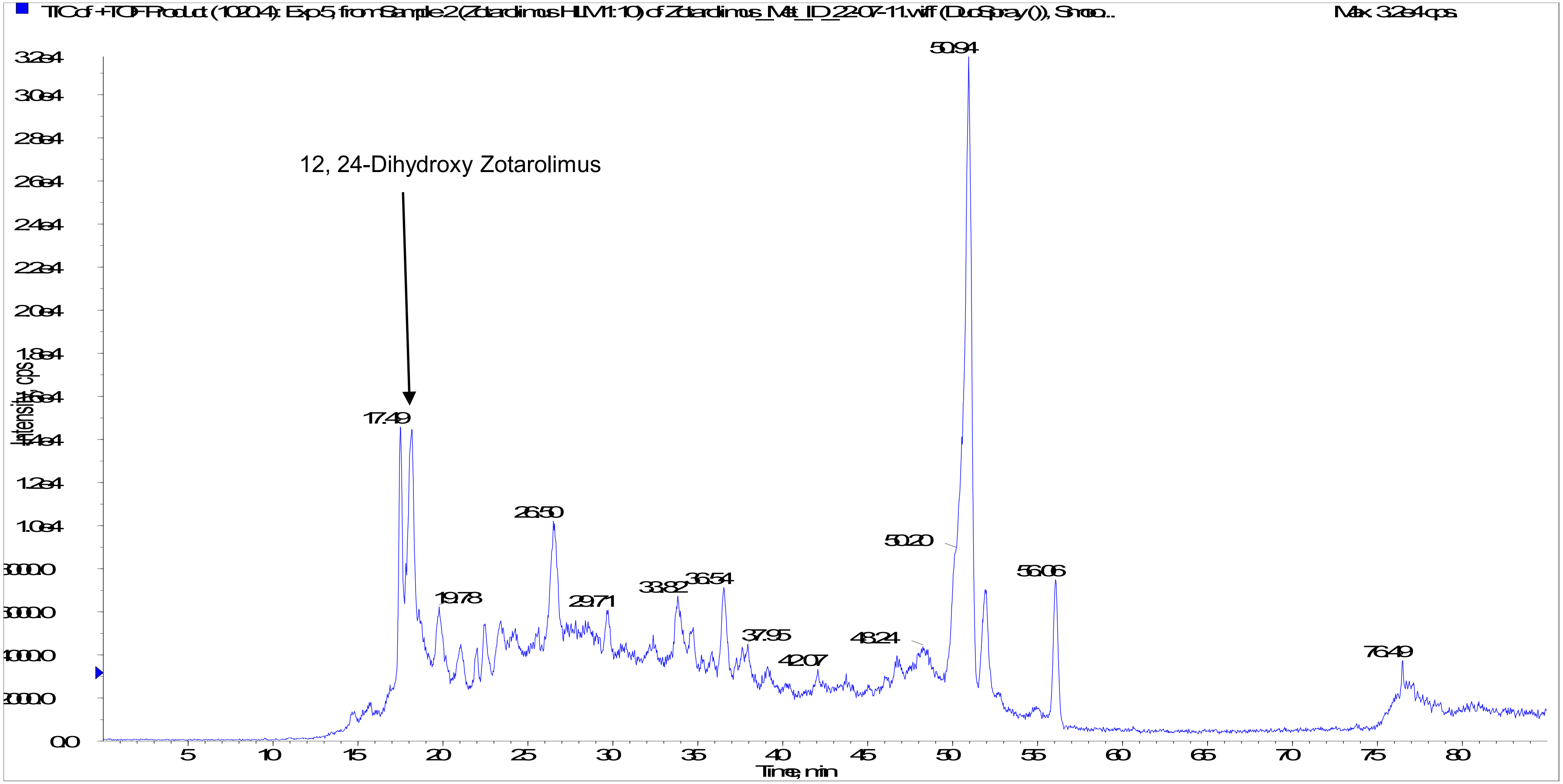

12, 24-Dihydroxy Zotarolimus

Mass Spectrum (TOF Fragmentation)

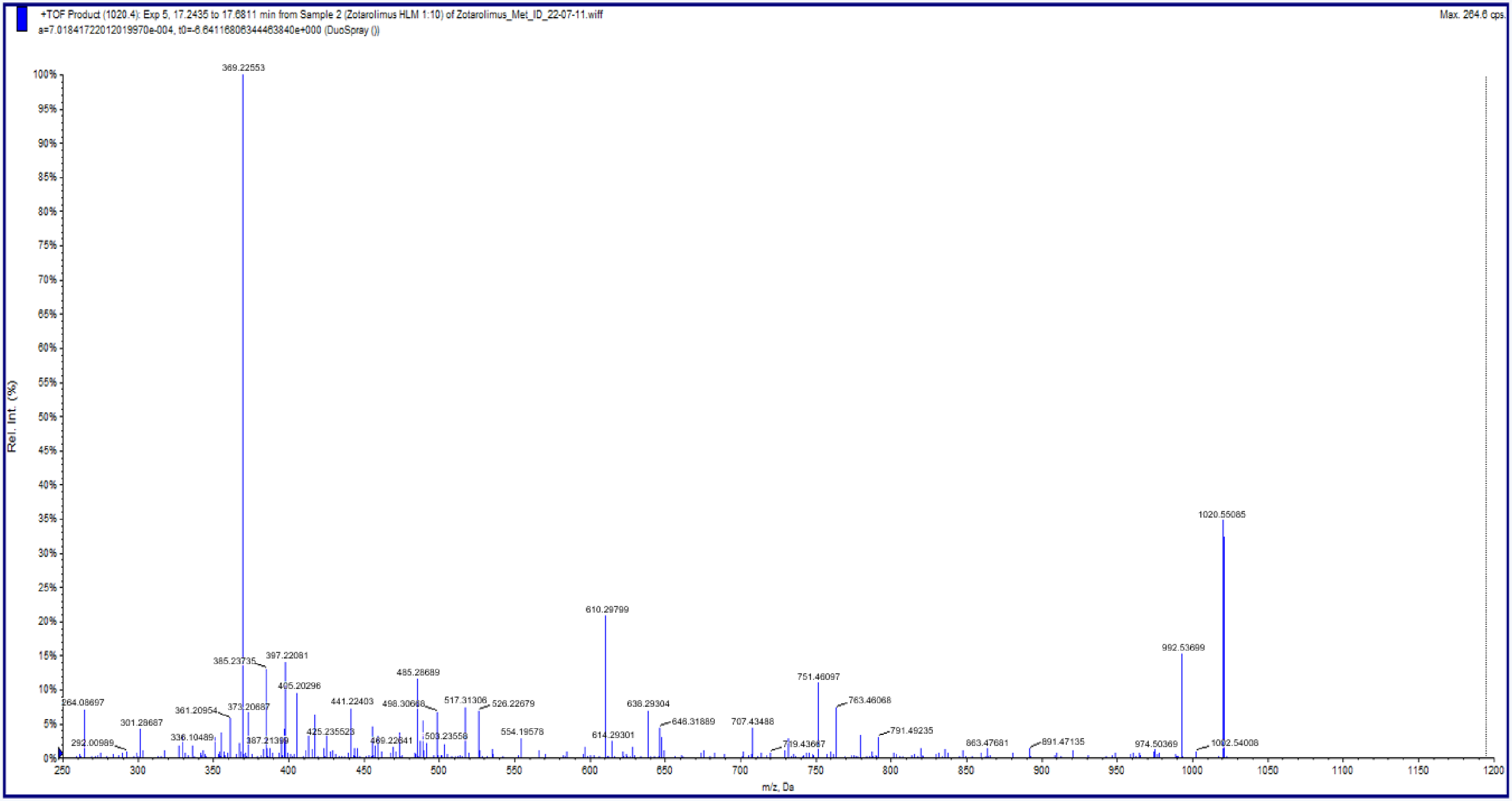

Δppm of 11, 24-Dihydroxy Zotarolimus

Fragment No.	Theoretical mass	Measured mass	Δppm
1	336.10537	336.10493	1.3
2	369.22610	369.22573	1.0
3	397.22101	397.22081	0.5
4	405.20363	405.20288	1.9
5	441.22476	441.22437	0.9
6	485.28736	485.28703	0.7
7	517.31357	517.31322	0.7
8	610.29865	610.29811	0.9
9	638.29357	638.29296	1.0
10	707.43542	707.43463	1.1
11	751.46164	751.46088	1.0
12	763.46164	763.46077	1.1
13	791.45655	791.45539	1.5
14	1002.54101	1002.54011	0.9
15	1020.55157	1020.55033	1.2

12, 24-Dihydroxy Zotarolimus Fragmentation Pattern

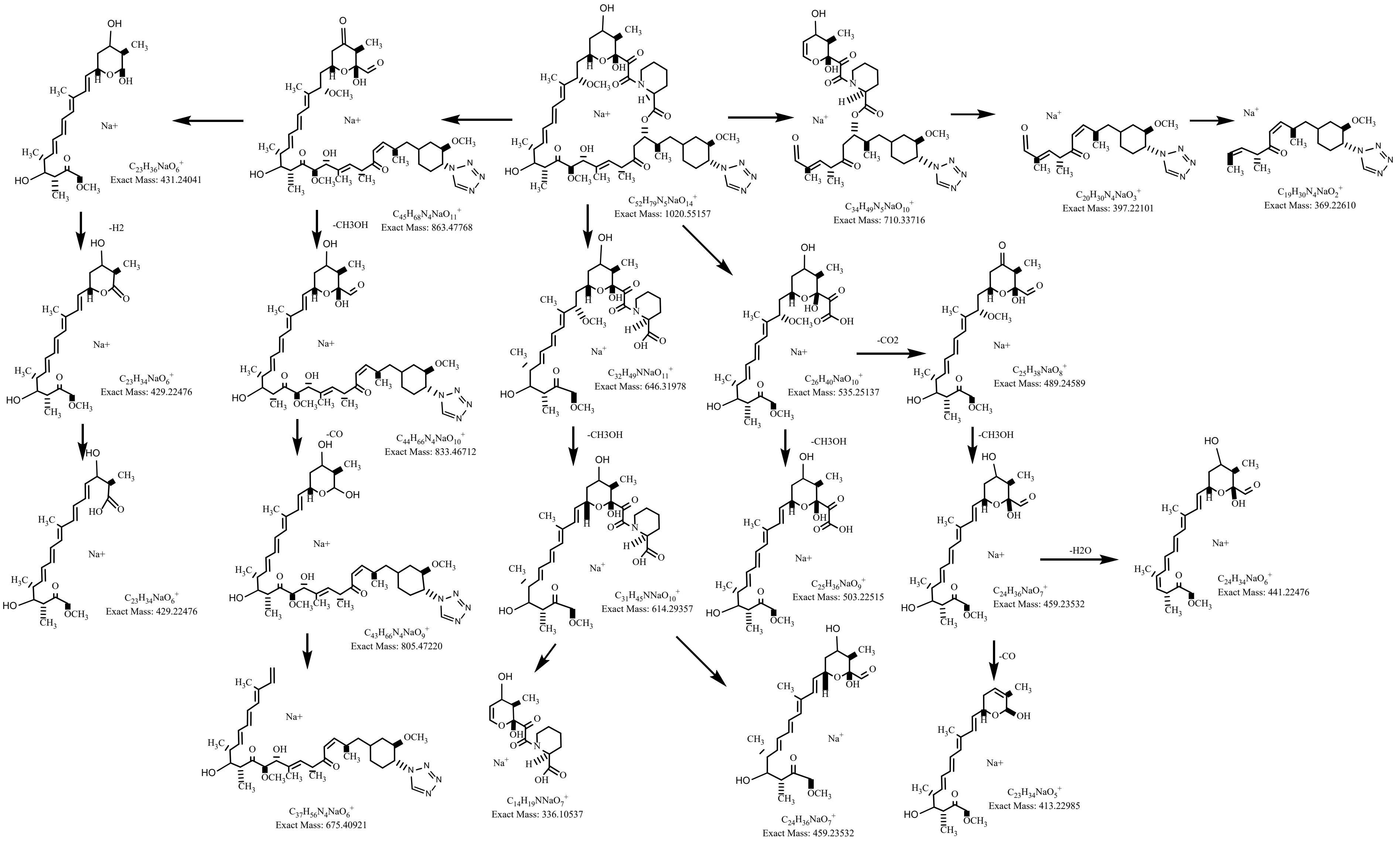

Water loss of 12, 24-Dihydroxy Zotarolimus Fragmentation Pattern

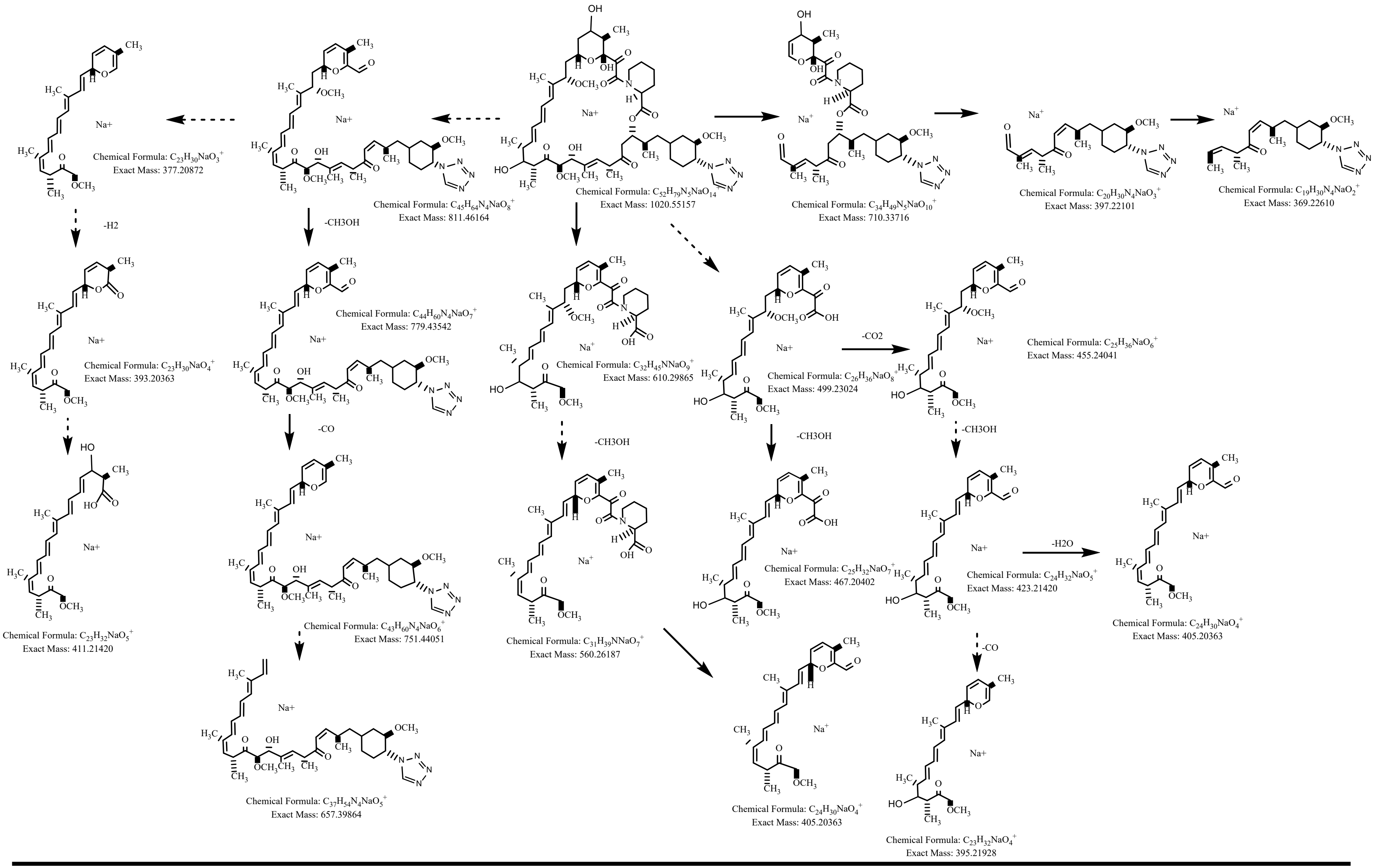

12, 24-Dihydroxy Zotarolimus Characteristic Fragmentation Pattern

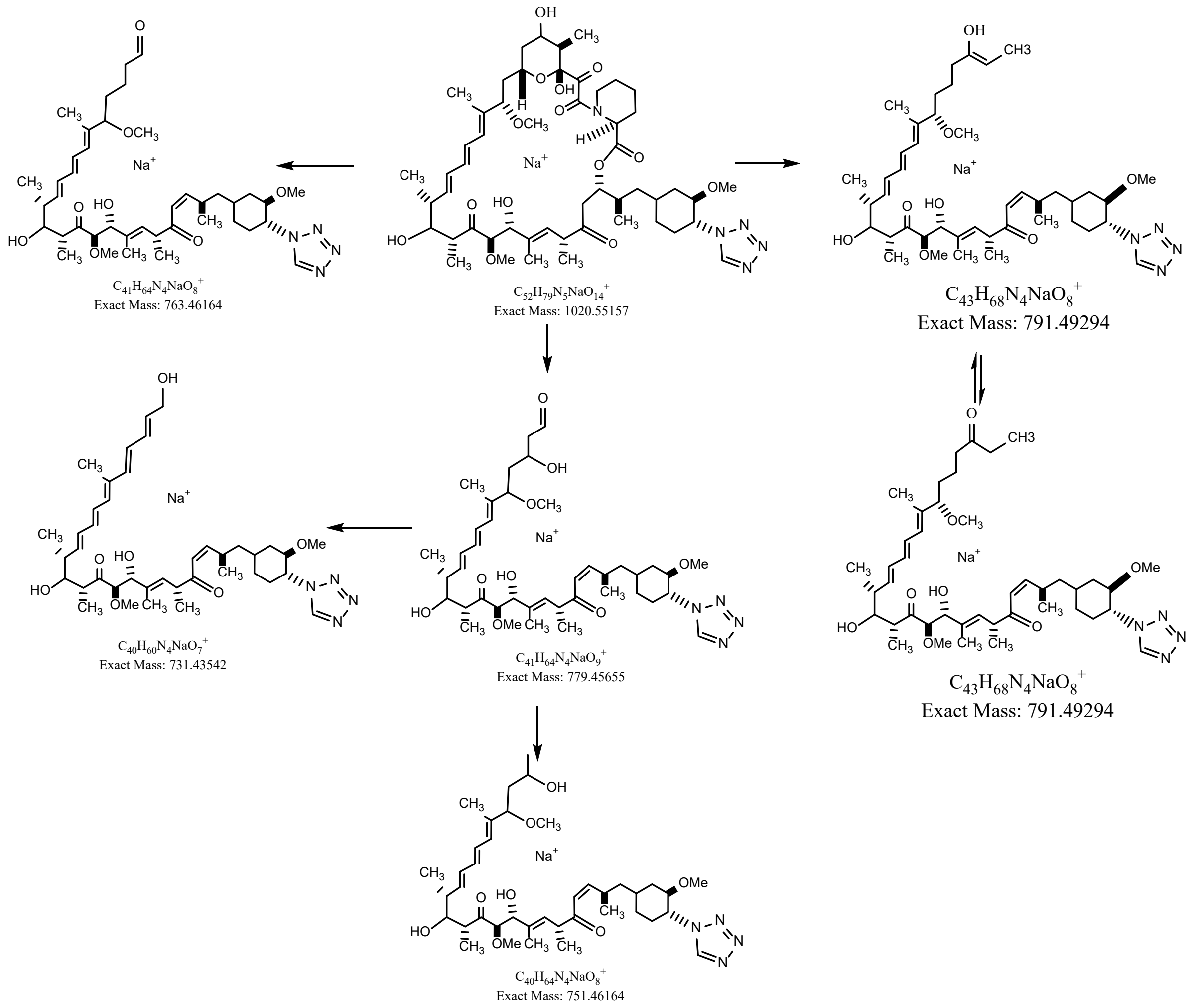

Structural Confirmation of 12, 24-dihydroxy Zotarolimus

Zotarolimus Fragments	12, 24-dihydroxy Zotarolimus Fragments	Comments	
320.11046	336.10493	hydroxylation	11, 12, 14, or OH-piperidine present
369.22610	369.22573	no hydroxylation	Rules out 47, 48, and 49-OH
397.22101	397.22081	no hydroxylation	Rules out 47, 48, and 49-OH
409.23493	441.22437	double hydroxylation with water loss	11, 12, 14, 23, 24, 45, or 46-OH present
427.2455	405.20296	double hydroxylation with double water loss	11, 12, 14, 23, 24, 45, or 46-OH present
441.26115	(491.26154) ND	ND	NA
453.22476	503.22406	double hydroxylation	11, 12, 14, 23, 24, 45, or 46-OH present
485.25097	517.31306	double hydroxylation with water loss	11, 12, 14, 23, 24, 45, or 46-OH present
614.32995	646.31889	double hydroxylation	11, 12, 14, 23, 24, 45, or 46-OH present
659.41429	675.40865	hydroxylation	11, 12, 14, 23, 24, 45, or 46-OH present
694.34225	710.33716	ND	11, 12, 14, 23, 24, 45, or 46-OH present
755.47181	805.47220	very low	11, 12, 14, 23, 24, 45, or 46, 49-OH present
783.46672	833.46712	very low	16, 27, or 39-O-desmethyl present
988.56174	1020.55085	no demethylation	
Characteristic Fragments	731, 763, 779, 791		
Determinant patterns			double water loss only if 12-OH position presents:<br>491- 2x (18) = 455<br>646 - 2x (18) = 610<br>459 - 3 x (18) = 405

11, 47/48-Dihydroxy Zotarolimus, m/z = 1020.55157

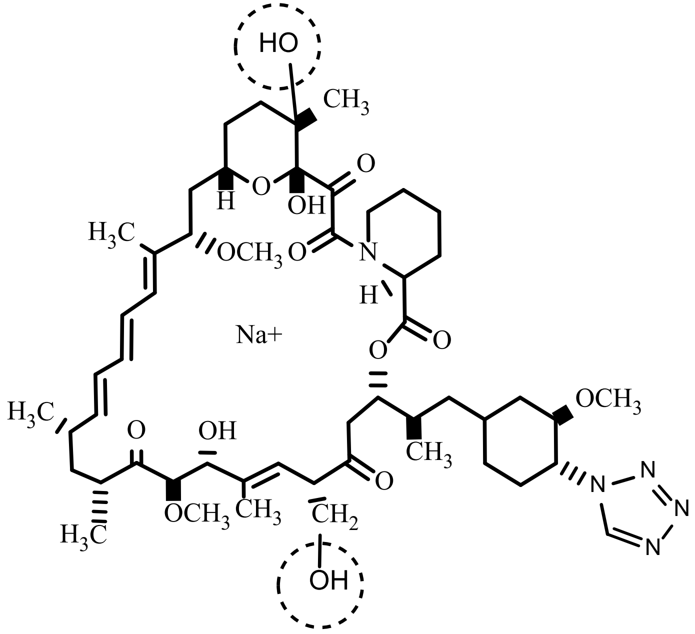

Chemical Formula: $C_{52}H_{79}N_5NaO_{14}^{+}$
Exact Mass: 1020.55157

11, 47-dihydroxy zotarolimus

Dihydroxy Zotarolimus Metabolites
Extracted Ion Chromatogram (EIC), m/z = 1020.55157

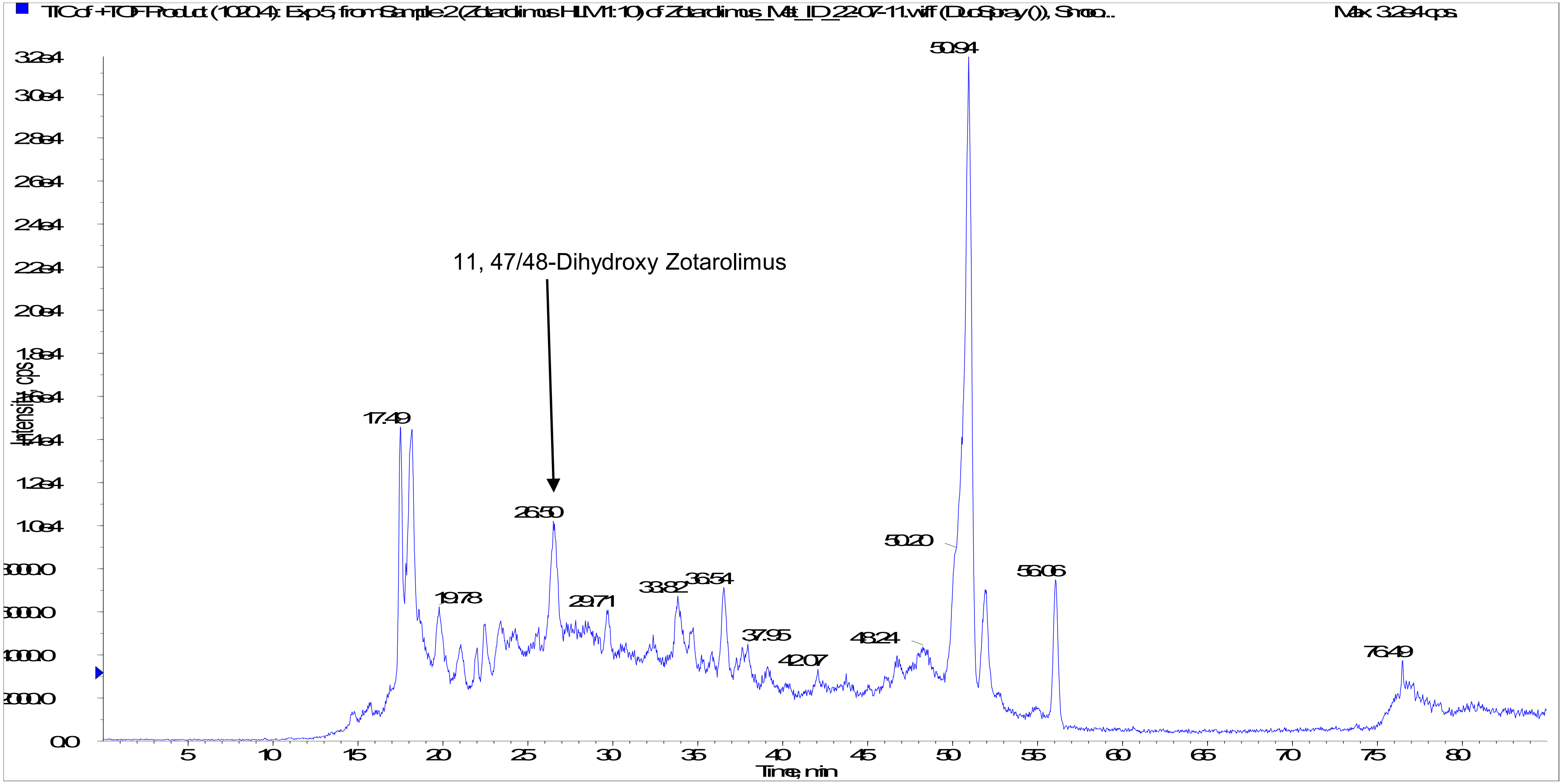

11, 47/48-Dihydroxy Zotarolimus

Mass Spectrum (TOF Fragmentation)

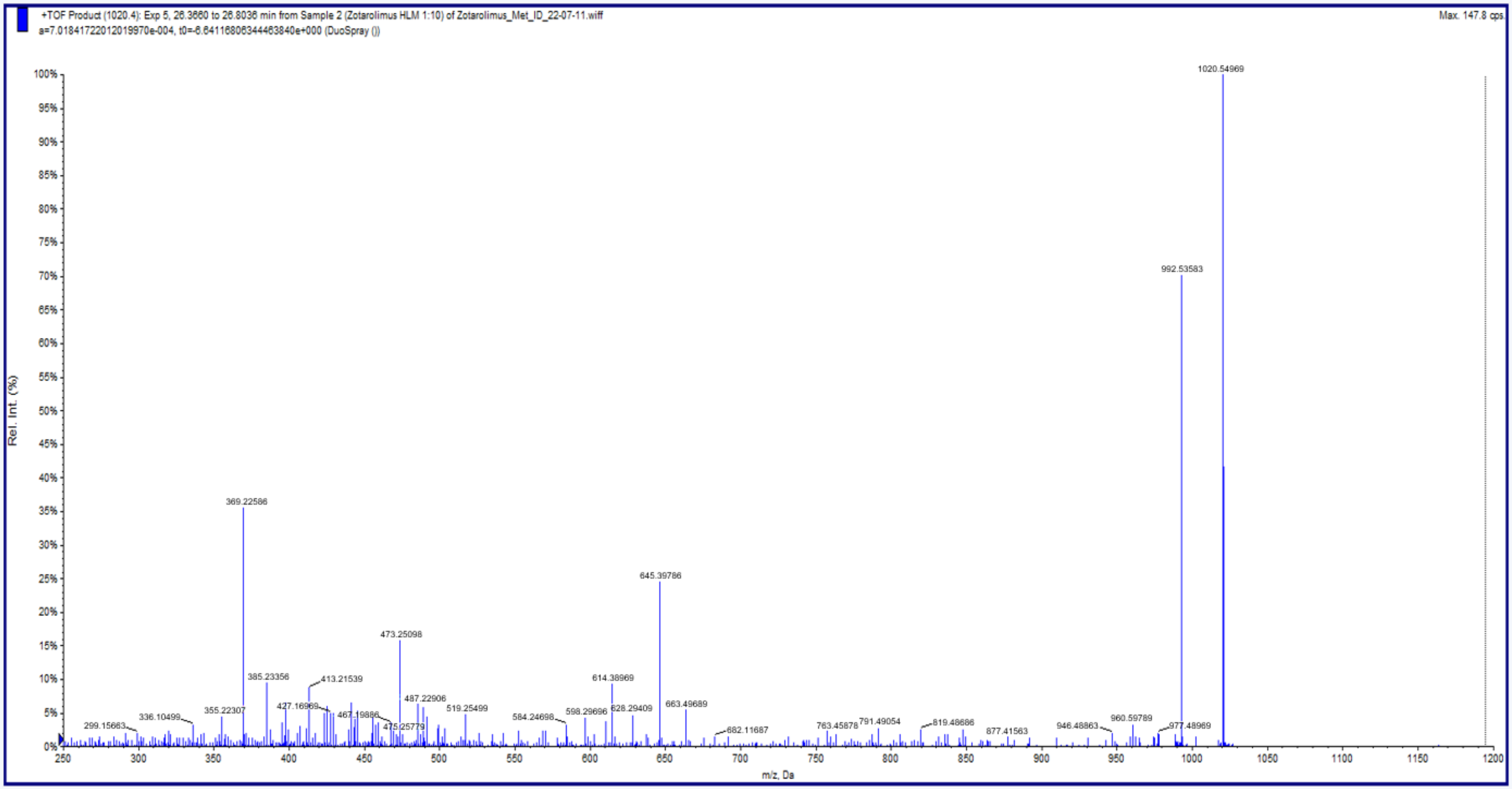

Δppm of 11, 47/48-Dihydroxy Zotarolimus

Fragment No.	Theoretical mass	Measured mass	Δppm
1	336.10537	336.10499	1.1
2	355.22437	355.22307	3.7
3	369.2261	369.22586	0.7
4	385.23493	385.23356	3.6
5	413.21593	413.21539	1.3
6	425.22985	425.22885	2.4
7	443.24041	443.2396	1.8
8	457.25606	457.25537	1.5
9	473.25097	473.25098	0.0
10	487.23024	487.22906	2.4
11	519.25645	519.25499	2.8
12	598.29865	598.29696	2.8
13	628.2953	628.29409	1.9
14	763.46164	763.45878	3.7
15	791.49294	791.49054	3.0
16	819.48785	819.48686	1.2
17	837.49842	837.49736	1.3
18	1002.54101	1002.53869	2.3
19	1020.55157	1020.54969	1.8

11, 48-Dihydroxy Zotarolimus Fragmentation Pattern

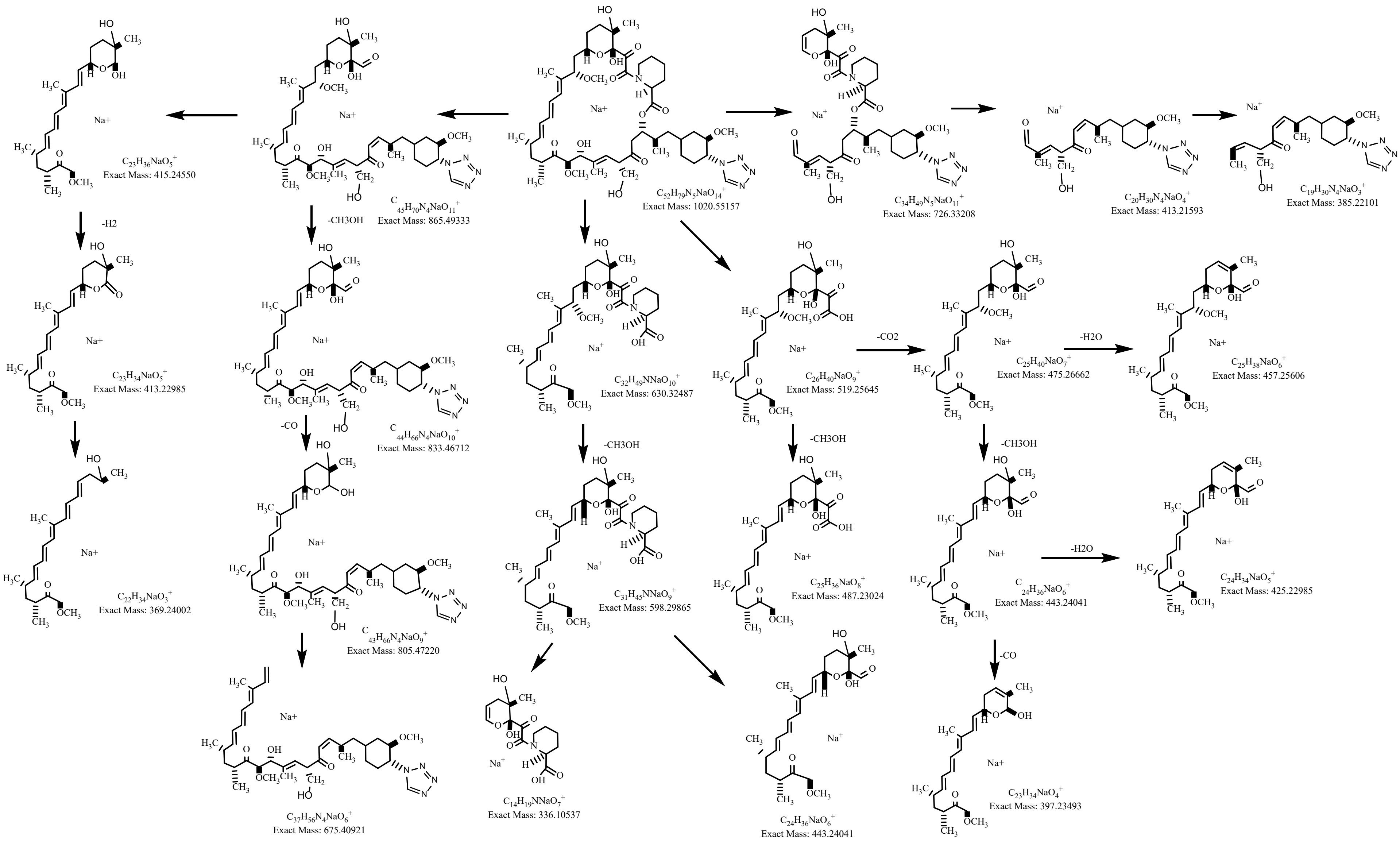

11, 48-Dihydroxy Zotarolimus Characteristic Fragmentation Pattern

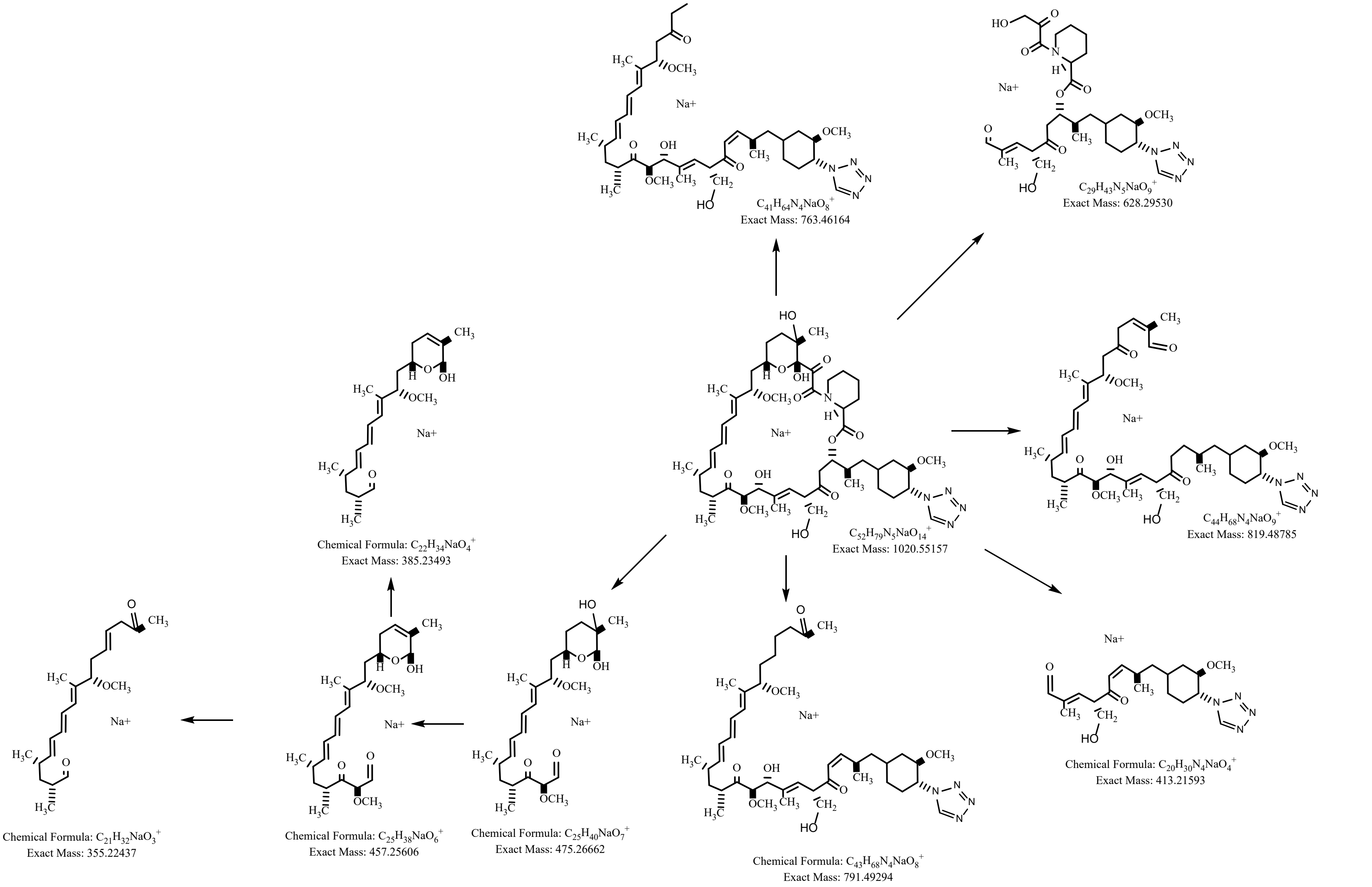

Structural Confirmation of 11, 47/48-dihydroxy Zotarolimus

Zotarolimus Fragments	11,47/48-dihydroxy Zotarolimus Fragments	Comments	
320.11046	336.10499	hydroxylation	11, 12, 14, or OH-piperidine present
397.22101	413.21539	hydroxylation	confirms 48 or 49-OH
409.23493	425.22885	hydroxylation	11, 12, 14, 23, 24, 45, or 46-OH present
427.2455	443.23960	hydroxylation	11, 12, 14, 23, 24, 45, or 46-OH present
441.26115	457.25537	hydroxylation	11, 12, 14, 23, 24, 45, or 46-OH present
453.22476	487.22906	hydroxylation	11, 12, 14, 23, 24, 45, or 46-OH present
503.26154	519.25499	hydroxylation	11, 12, 14, 23, 24, 45, or 46-OH present
614.32995	630.32487	ND	11, 12, 14, 23, 24, 45, or 46-OH present
659.41429	675.40823	hydroxylation	23, 24, 45, 46, 47, 48 or 49-OH present
694.34225	726.33208	hydroxylation (low intensity)	11, 12, 14, or OH-piperidine present
755.47181	805.47178	hydroxylation (low intensity)	11, 12, 14, 23, 24, 45, 46, 47, 48, OR 49-OH present
783.46672	833.46686	hydroxylation (low intensity)	11, 12, 14, 23, 24, 45, 46, 47, 48, OR 49-OH present
988.56174	1020.55085	hydroxylation	
Characteristic Fragments	355, 385, 413, 473, 763, 791, 819		
Determinant patterns			no additional water loss at 11-OH position possible<br>No methanol loss possible at 47 or 48 positions

Representative negative and positive control samples: total ion chromatograms (TIC) measured on a Sciex API4000 triple quadrupole mass spectrometer

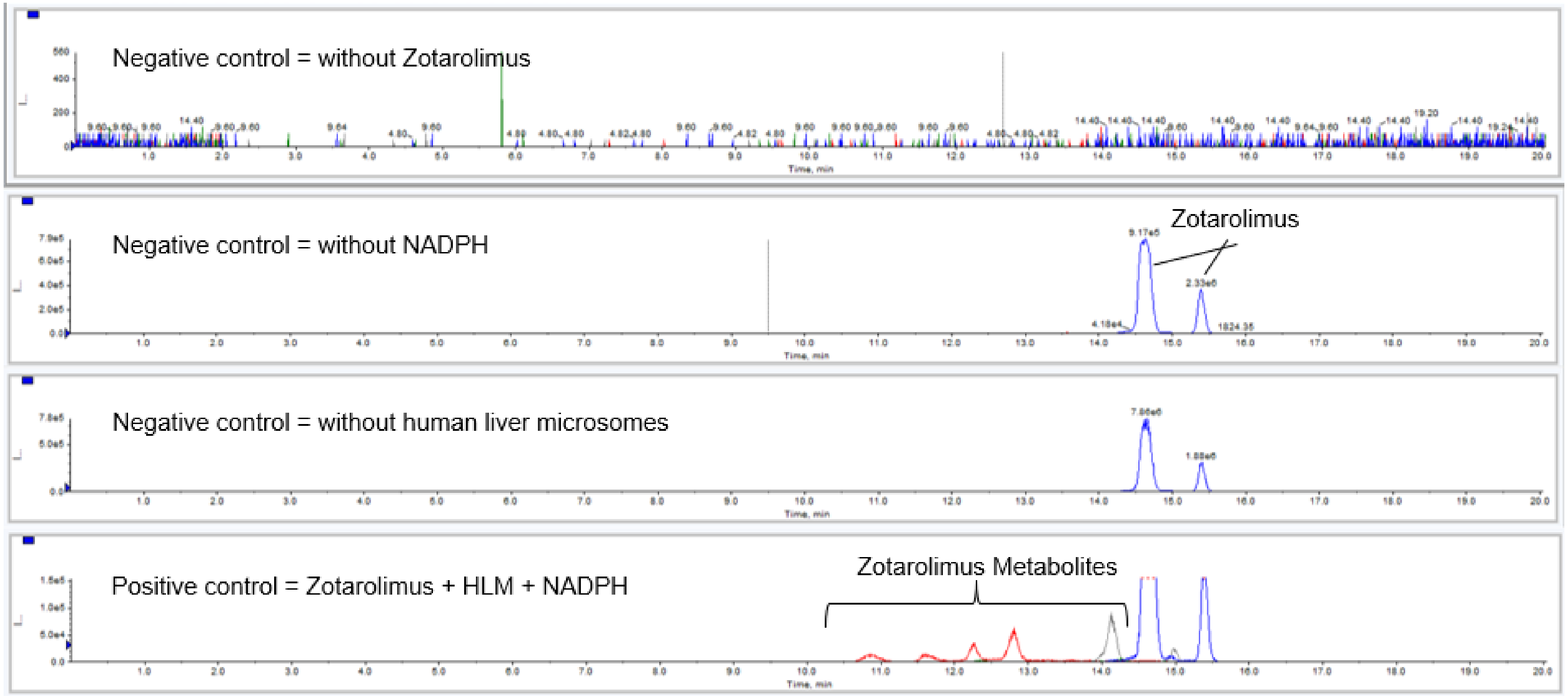

Representative negative and positive control samples: total ion chromatograms (TIC) measured on the Sciex 5600+ triple TOF high-resolution mass spectrometer

Positive control
Zotarolimus + HLM + NADPH

Without HLM

Without Zotarolimus

Without NADPH

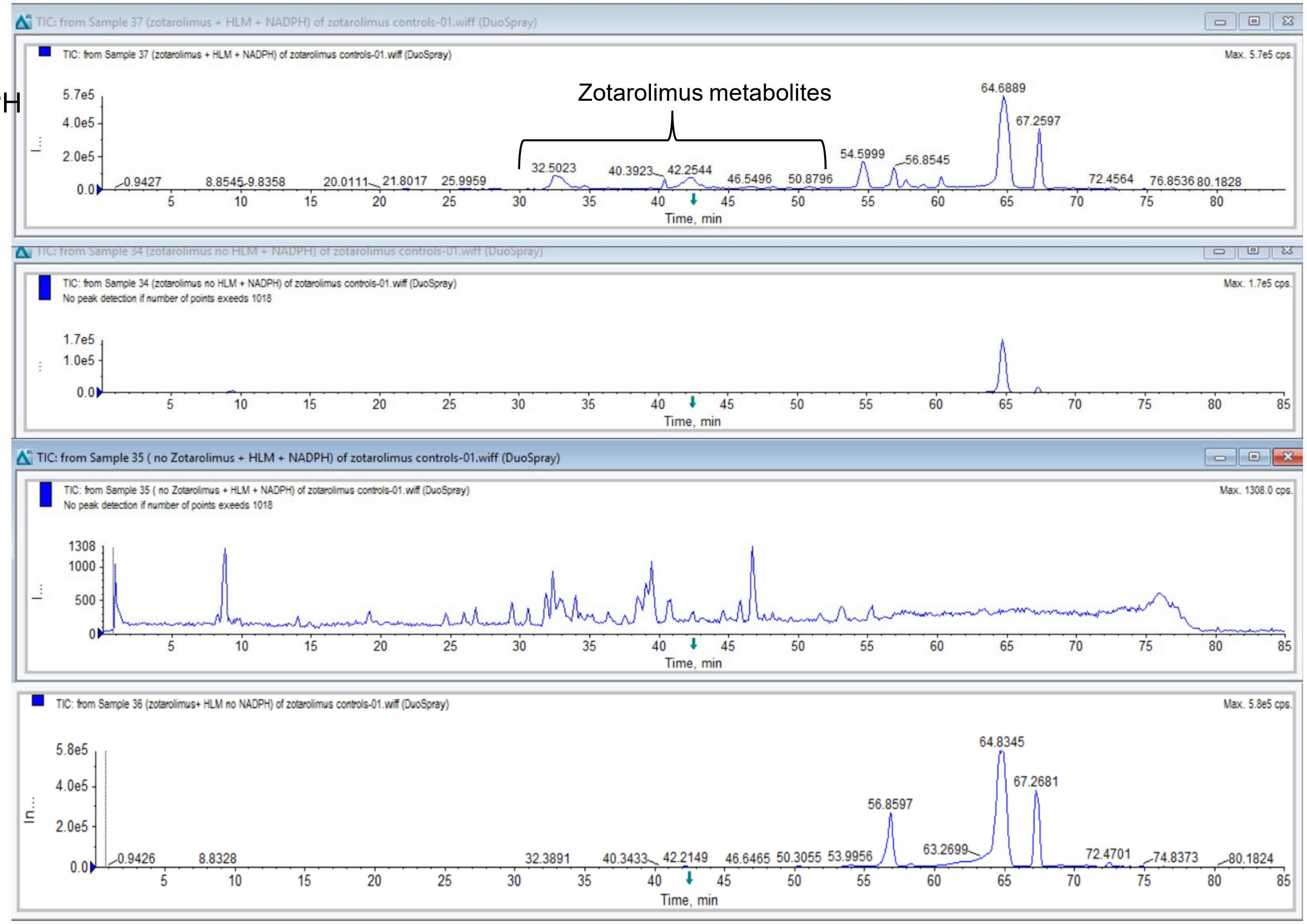

Supplement: Supplementary file 1 [file metabolites-13-01093-s001.zip › metabolites-2633405-supplementary.pdf]
